# Anti-EGFR Rechallenge in Patients With Refractory ctDNA *RAS/BRAF* wt Metastatic Colorectal Cancer: A Nonrandomized Controlled Trial

**DOI:** 10.1001/jamanetworkopen.2024.5635

**Published:** 2024-04-09

**Authors:** Davide Ciardiello, Erika Martinelli, Teresa Troiani, Gianluca Mauri, Daniele Rossini, Giulia Martini, Stefania Napolitano, Vincenzo Famiglietti, Sara Del Tufo, Gianluca Masi, Daniele Santini, Antonio Avallone, Filippo Pietrantonio, Sara Lonardi, Massimo Di Maio, Maria Giulia Zampino, Nicola Fazio, Alberto Bardelli, Salvatore Siena, Chiara Cremolini, Andrea Sartore-Bianchi, Fortunato Ciardiello

**Affiliations:** 1 Division of Gastrointestinal Medical Oncology and Neuroendocrine Tumors, European Institute of Oncology, IEO, IRCCS, Milan, Italy; 2 Department of Precision Medicine, Università degli Studi della Campania Luigi Vanvitelli, Naples, Italy; 3 Department of Oncology and Hemato-Oncology, Università degli Studi di Milano, Milano, Italy; 4 Department of Hematology, Oncology and Molecular Medicine, Grande Ospedale Metropolitano Niguarda, Milano, Italy; 5 IFOM ETS–The AIRC Institute of Molecular Oncology, Milan, Italy; 6 Department of Translational Research and New Technologies in Medicine and Surgery, University of Pisa, Pisa, Italy; 7 Division of Medical Oncology, Azienda Ospedaliero-Universitaria Pisana, Pisa, Italy; 8 Medical Oncology Department, La Sapienza University of Rome, Rome, Italy; 9 Experimental Clinical Abdominal Oncology Unit, Istituto Nazionale Tumori–IRCCS–Fondazione G. Pascale, Napoli, Italy; 10 Medical Oncology Department, Fondazione IRCCS Istituto Nazionale dei Tumori, Milan, Italy; 11 Istituto Oncologico Veneto IOV-IRCCS, Padova, Italy; 12 Department of Oncology, University of Turin, Molinette Hospital, Turin, Italy; 13 Department of Oncology, Università degli Studi di Torino, Turin, Italy

## Abstract

**Importance:**

The available evidence regarding anti–epidermal growth factor receptor (EGFR) inhibitor rechallenge in patients with refractory circulating tumor DNA (ctDNA) *RAS/BRAF* wild-type (wt) metastatic colorectal cancer (mCRC) is derived from small retrospective and prospective studies.

**Objective:**

To evaluate the efficacy of anti-EGFR rechallenge in patients with refractory ctDNA *RAS/BRAF* wt mCRC.

**Design, Setting, and Participants:**

This nonrandomized controlled trial used a pooled analysis of individual patient data from patients with *RAS/BRAF* wt ctDNA mCRC enrolled in 4 Italian trials (CAVE, VELO, CRICKET, and CHRONOS) and treated with anti-EGFR rechallenge between 2015 and 2022 (median [IQR] follow-up, 28.1 [25.8-35.0] months).

**Intervention:**

Patients received anti-EGFR rechallenge therapy, including cetuximab plus avelumab, trifluridine-tipiracil plus panitumumab, irinotecan plus cetuximab, or panitumumab monotherapy.

**Main Outcomes and Measures:**

Overall survival (OS), progression-free survival (PFS), overall response rate (ORR), and disease control rate (DCR) were calculated. Exploratory subgroup analysis evaluating several clinical variables was performed. Safety was reported.

**Results:**

Overall, 114 patients with *RAS/BRAF* wt ctDNA mCRC (median [IQR] age, 61 [29-88] years; 66 men [57.9%]) who received anti-EGFR rechallenge as experimental therapy (48 received cetuximab plus avelumab, 26 received trifluridine-tipiracil plus panitumumab, 13 received irinotecan plus cetuximab, and 27 received panitumumab monotherapy) were included in the current analysis. Eighty-three patients (72.8%) had received 2 previous lines of therapy, and 31 patients (27.2%) had received 3 or more previous lines of therapy. The ORR was 17.5% (20 patients), and the DCR was 72.3% (82 patients). The median PFS was 4.0 months (95% CI, 3.2-4.7 months), and the median OS was 13.1 months (95% CI, 9.5-16.7 months). The subgroup of patients without liver involvement had better clinical outcomes. The median PFS was 5.7 months (95% CI, 4.8-6.7 months) in patients without liver metastasis compared with 3.6 months (95% CI, 3.3-3.9 months) in patients with liver metastasis (hazard ratio, 0.56; 95% CI, 0.37-0.83; *P* = .004). The median OS was 17.7 months (95% CI, 13-22.4 months) in patients without liver metastasis compared with 11.5 months (95% CI, 9.3-13.9 months) in patients with liver metastasis (hazard ratio, 0.63; 95% CI, 0.41-0.97; *P* = .04). Treatments showed manageable toxic effects.

**Conclusions and Relevance:**

These findings suggest that anti-EGFR rechallenge therapy has promising antitumor activity in patients with refractory ctDNA *RAS/BRAF* wt mCRC. Within the limitation of a subgroup analysis, the absence of liver metastases was associated with significant improved survival.

**Trial Registration:**

ClinicalTrials.gov Identifiers: NCT02296203; NCT04561336; NCT03227926; NCT05468892

## Key Points

**Question:**

Is rechallenge with anti–epidermal growth factor receptor (EGFR) inhibitors a therapeutic option in patients with refractory circulating tumor DNA (ctDNA) *RAS/BRAF* wild-type (wt) colorectal cancer (CRC)?

**Findings:**

This nonrandomized controlled trial used a pooled analysis of individual patient data from 114 patients with ctDNA *RAS/BRAF* wt metastatic CRC receiving anti-EGFR rechallenge therapy in 4 prospective, phase 2 Italian trials. The treatment strategy was associated with tumor shrinkage, a high rate of disease control, and promising progression-free and overall survival, with a significant improvement in patients without liver involvement.

**Meaning:**

These findings suggest that rechallenge with anti-EGFR inhibitors has promising antitumor activity in patients with refractory ctDNA *RAS/BRAF* wt metastatic CRC.

## Teaser

This nonrandomized controlled trial evaluates anti–epidermal growth factor receptor (EGFR) rechallenge therapy in patients in Italy with circulating tumor DNA (ctDNA) *RAS/BRAF* wild-type (wt) metastatic colorectal cancer.

## Introduction

Treatment with anti–epidermal growth factor receptor (EGFR) monoclonal antibodies (mAbs) in combination with chemotherapy is a standard of care as first-line treatment for patients with *RAS/BRAF* wild-type (wt) metastatic colorectal cancer (mCRC).^1^ Despite an initial antitumor activity with high overall response rate (ORR), disease progression almost inevitably occurs as a result of cancer cells acquiring resistance.^2,3^

It is well known that the most frequent resistance alterations are associated with the EGFR signaling cascade, such as the EGFR extracellular domain (ECD) or alterations in downstream effectors like *KRAS/NRAS* and *BRAF*. Nevertheless, the spectrum of the molecular alterations may change over time depending on the treatment received. By use of serial liquid biopsy of circulating tumor DNA (ctDNA) status, it has been shown that *RAS/BRAF/EGFR ECD* alteration cancer cell clones have a half-life of approximately 4 months.^4,5^ Therefore, after disease progression, during a so-called anti-EGFR therapeutic holiday, resistant clones might decay, thereby potentially restoring sensitivity to EGFR blockade.^3,4,5^ Consequently, over the last decade, different groups have investigated the role of anti-EGFR rechallenge therapy in patients with refractory *RAS* wt mCRC.^6,7,8,9,10,11,12,13,14,15,16^ The main clinical criteria for patient selection in these trials were receiving an anti-EGFR–based therapy, experiencing a clinical benefit followed by progressive disease, and then receiving a subsequent EGFR-free treatment. In an unselected population with refractory disease, the combination of chemotherapy with anti-EGFR rechallenge was associated with heterogenous responses, with an ORR ranging from 0% to 54%.

Translational retrospective analyses of these studies showed that patients with *RAS/BRAF* wt ctDNA showed the highest clinical benefit from this treatment.^9,10,11,13,14,15^ To date, the CHRONOS (Rechallenge With Panitumumab Driven by *RAS* Dynamic of Resistance) trial, in which patients with *RAS/BRAF* wt mCRC with received panitumumab rechallenge, is the only study that prospectively used liquid biopsy for patient selection.^12^

Despite the strong rationale, the quality of the available evidence on the role of anti-EGFR rechallenge in ctDNA *RAS/BRAF* wt tumors is poor, because it has been mainly derived via post hoc analysis performed with limited numbers of patients. Consequently, caution is required when interpreting these results, and further validation is needed. Moreover, in a molecularly selected population, the identification of other variables potentially associated with clinical activity represents an unmet need.

To fill this gap, we conducted a pooled analysis of individual patient data (IPD) from 4 prospective phase 2 trials. Only patients with ctDNA *RAS/BRAF* wt tumors confirmed by liquid biopsy at baseline were included. Finally, exploratory subgroup analyses were performed to identify further potential biomarkers.

## Methods

### Study Population

In this nonrandomized controlled trial, we conducted a pooled analysis of IPD from 4 multicenter, phase 2 studies: CRICKET (Cetuximab Rechallenge in Irinotecan-pretreated mCRC, *KRAS*, *NRAS* and *BRAF* Wild-type Treated in 1st Line With Anti-EGFR Therapy), CAVE (Avelumab Plus Cetuximab in Pre-treated *RAS* Wild Type Metastatic Colorectal Cancer), CHRONOS, and VELO (Phase II Randomized Study Evaluating the Efficacy of Panitumumab [Vectibix] and Trifluridine-Tipiracil [Lonsurf] in Pretreated *RAS* Wild Type Metastatic Colorectal Cancer Patients) trials. Patients who received rechallenge with EGFR inhibitors and exhibited *RAS/BRAF* wt ctDNA tumors at baseline were included.

The 4 studies were conducted in accordance with the principles of the Declaration of Helsinki^17^ and were approved by the ethical committees of all participating centers. Patients provided written informed consent for trial participation. This study followed the Transparent Reporting of Evaluations With Nonrandomized Designs (TREND) reporting guideline.

The CRICKET study was a single-group phase 2 trial that investigated rechallenge with cetuximab plus irinotecan as third-line treatment in patients with *RAS/BRAF* wt mCRC.^9^ The study provided the first prospective evidence of rechallenge therapy in refractory mCRC. Post hoc analysis showed that confirmed responses were observed only in patients without resistance alterations detected at liquid biopsy analysis. The CAVE trial was a single-group, multicenter, phase 2 study that evaluated rechallenge with cetuximab plus the anti–programmed cell death ligand 1 mAbs avelumab in patients with heavily pretreated *RAS* wt mCRC.^11^ The study met its primary end point, and an increase in survival of more than 3 months compared with historical controls was reported. The CHRONOS study was the first trial that prospectively selected patients amenable for rechallenge therapy with panitumumab on the basis of the results of liquid biopsy using highly sensitive digital droplet polymerase chain reaction (ddPCR) to detect *RAS/BRAF/EGFR ECD* alterations in the plasma.^12^ The trial was positive, with an ORR of 30%. The VELO trial was a randomized phase 2 study that compared rechallenge with panitumumab plus trifluridine-tipiracil vs trifluridine-tipiracil as third-line treatment in *RAS* wt mCRC.^13,14^ The trial reached its primary end point, demonstrating a significant increase in progression-free survival (PFS) of the experimental group compared with the standard of care. The full study protocols with inclusion and exclusion criteria have been previously published^9,11,12,13,14^ and are shown in Supplement 1.

**Figure 1.**
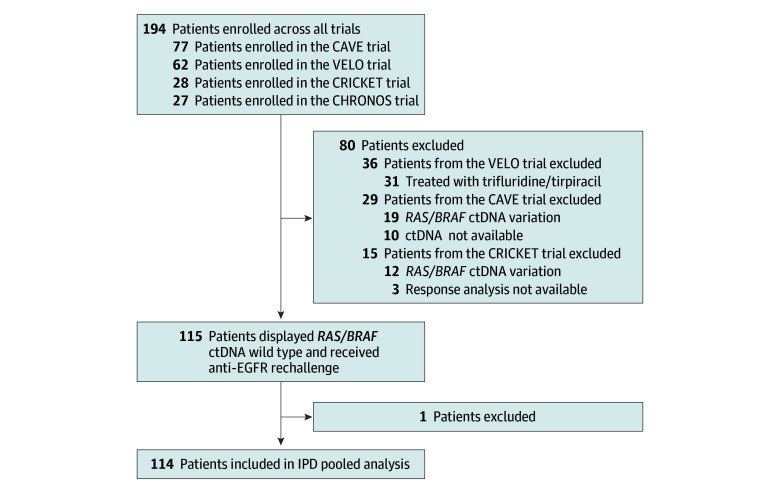
Patient Enrollment Flowchart CAVE indicates Avelumab Plus Cetuximab in Pre-treated *RAS* Wild Type Metastatic Colorectal Cancer; CHRONOS, Rechallenge With Panitumumab Driven by *RAS* Dynamic of Resistance; CRICKET, Cetuximab Rechallenge in Irinotecan-pretreated mCRC, *KRAS*, *NRAS* and *BRAF* Wild-type Treated in 1st Line With Anti-EGFR Therapy; ctDNA, circulating tumor DNA; IPD, individual patient data; VELO, Phase II Randomized Study Evaluating the Efficacy of Panitumumab (Vectibix) and Trifluridine-Tipiracil (Lonsurf) in Pretreated *RAS* Wild Type Metastatic Colorectal Cancer Patients.

The procedure for patient selection is displayed in Figure 1. Among the 194 patients enrolled in the 4 trials, 80 patients did not meet the inclusion criteria (ie, having received an anti-EGFR rechallenge treatment with baseline *RAS/BRAF* alteration ctDNA at liquid biopsy analysis) and were excluded. In the group of patients not eligible for the current analysis, 15 were from the CRICKET trial (3 were not evaluable for tumor response, and 12 showed *RAS/BRAF* alteration ctDNA), 29 from the CAVE trial (10 did not have available baseline plasma samples, and 19 displayed *RAS/BRAF* alteration ctDNA), and 36 from the VELO trial (31 did not receive anti-EGFR rechallenge, and 5 exhibited *RAS/BRAF* alteration ctDNA). To facilitate data gathering and analysis, a study data set including key information from the 4 trials was set up. The following data were extrapolated from each trial: age, sex, Eastern Cooperative Oncology Group performance status, primary tumor sidedness, resection of primary tumor, microsatellite status, number of previous lines of treatment, type of anti-EGFR treatment received as first-line or second-line therapy, number of metastatic sites, metastasis location (liver, lung, peritoneum, and lymph nodes), carcinoembryonic antigen levels, treatment efficacy (ORR, PFS, and overall survival [OS]), and toxic effects.

### Efficacy and Safety of the Treatments

The ORR was defined as the percentage of patients who achieved complete or partial response during treatment according to RECIST version 1.1.^18^ PFS was defined as the time from the initiation of study treatment to disease progression or death. OS was determined as the time from the beginning of the experimental therapy to death. In the absence of events, PFS and OS were censored at the time of last follow-up.

Tumor measurements were done at baseline and were repeated every 8 weeks in the CRICKET, CHRONOS, and VELO studies. In the CAVE trial, tumor measurements were performed at baseline, and every 8 weeks for the first 40 weeks and subsequently every 12 weeks. Adverse events were graded according to the National Cancer Institute’s Common Terminology Criteria for Adverse Events version 4.0 for CRICKET, version 4.03 for CAVE and CHRONOS, and version 5.0 for VELO studies.^19^

### Molecular Analysis

In the CRICKET study, baseline plasma samples were available for 25 patients and were analyzed using the next-generation sequencing Ion AmpliSeq Cancer Hotspot Panel (Thermo Fisher).^7^ For the CAVE trial, pretreatment plasma samples were collected for 67 patients and were analyzed using a reverse transcriptase–PCR test (IdyllaTM Biocartis platform).^11,20^ In the CHRONOS trial, baseline liquid biopsy analysis was performed during the screening procedures. Overall, ctDNA from 52 patients was assessed using a ddPCR-based assay (Bio-Rad) for the identification of the most frequent *KRAS*, *BRAF*, and *EGFR ECD* alterations. Finally, for the 31 patients enrolled in the VELO trial who received rechallenge therapy, ctDNA was retrospectively evaluated using the IdyllaTM Biocartis assay.^13,14,20^

### Statistical Analysis

Descriptive statistics were used to summarize clinical and pathological outcomes. The Kaplan-Meier method was used to estimate PFS and OS. The subgroup exploratory analyses were conducted by using the Cox hazard ratio (HR) regression model. Comparison of treatment efficacy in the patient subgroups with or without liver metastases was performed with the log-rank test for PFS and OS. Statistical analyses were performed using SPSS statistical software version 23.0 (IBM). The threshold for statistical significance was set at 2-sided *P* < .05.

## Results

### Patient Characteristics

Overall, 114 patients (median [IQR] age, 61 [29-88] years; 66 men [57.9%]) met the specified criteria and were included in the pooled data set: 13 patients from the CRICKET trial received irinotecan plus cetuximab, 48 patients from the CAVE trial received cetuximab plus avelumab, 27 patients from the CHRONOS trial received panitumumab monotherapy, and 26 patients from the VELO trial received trifluridine-tipiracil plus panitumumab. Baseline clinical characteristics of the study population are detailed in Table 1. Eighty-three patients (72.8%) received 2 previous lines of therapy, and 31 (27.2%) patients received 3 or more anticancer treatments. In total, 36 patients (31.6%) had 3 or more different metastatic sites. Liver was the most frequent metastatic site (72 patients [63.2%]), followed by lung (63 patients [55.3%]), lymph nodes (47 patients [41.2%]), and peritoneum (26 patients [22.8%]). Increased carcinoembryonic antigen levels (>5 ng/mL; to convert to micrograms per liter, multiply by 1.0) were reported in 76 patients (66.7%). The type of previous anti-EGFR treatment was balanced in the study population; 57 patients (50.0%) received cetuximab, 56 patients (49.0%) received panitumumab, and 1 patient (0.9%) received both drugs.

**Table 1.** Baseline Characteristics of Patients With Metastatic Colorectal Cancer Receiving Anti-EGFR Challenge Therapy in 4 Italian Trials

Characteristic	Patients, No. (%)				
	CRICKET (n = 13)	CAVE (n = 48)	VELO (n = 26)	CHRONOS (n = 27)	Pooled analysis (N = 114)
Sex					
Female	4 (30.8)	23 (47.9)	10 (38.5)	11 (40.7)	48 (42.1)
Male	9 (69.2)	25 (52.1)	16 (61.5)	16 (59.3)	66 (57.9)
Age, median (IQR), y	68 (45-86)	60 (30-88)	63 (39-81)	59 (29-78)	61 (29-88)
Eastern Cooperative Oncology Group performance status					
0	10 (76.9)	34 (70.8)	18 (69.2)	14 (51.9)	76 (66.7)
1	2 (15.4)	14 (29.2)	8 (30.8)	12 (44.4)	36 (31.6)
2	1 (7.7)	0	0	1 (3.7)	2 (1.8)
Tumor sidedness					
Right rectum	3 (23.1)	1 (2.1)	1 (3.8)	5 (18.5)	10 (8.8)
Left rectum	10 (76.9)	47 (97.9)	25 (96.2)	22 (81.5)	104 (91.2)
Resection of primary tumor					
No	1 (7.7)	16 (33.3)	4 (15.4)	2 (7.2)	23 (20.2)
Yes	12 (92.3)	32 (66.7)	22 (84.6)	25 (92.6)	91 (79.8)
Microsatellite status					
Microsatellite stable	0	44 (91.7)	12 (46.2)	27 (100.0)	83 (72.8)
Microsatellite instable	0	2 (4.2)	1 (3.8)	0	3 (2.6)
NA	13 (100.0)	2 (4.2)	13 (50)	0	28 (24.6)
No. of previous lines of treatment					
2	13 (100.0)	33 (68.8)	26 (100.0)	11 (40.7)	83 (72.8)
≥3	0	15 (31.3)	0	16 (59.3)	31 (27.2)
Previous anti-EGFR treatment					
Panitumumab	0	25 (52.1)	17 (65.4)	15 (55.6)	57 (50.0)
Cetuximab	13 (100.0)	23 (47.9)	9 (34.6)	11 (40.7)	56 (49.1)
Both	0	0	0	1 (3.7)	1 (0.9)
No. of metastatic sites					
≤2	8 (61.5)	32 (66.7)	20 (76.9)	18 (66.7)	78 (68.4)
>2	5 (38.5)	16 (33.3)	6 (23.1)	9 (33.3)	36 (31.6)
Liver metastasis					
No	3 (23.1)	18 (37.5)	10 (38.5)	11 (40.7)	42 (36.8)
Yes	10 (76.9)	30 (62.5)	16 (61.5)	16 (59.3)	72 (63.2)
Lung metastasis					
No	8 (61.5)	16 (33.3)	13 (50.0)	14 (51.9)	51 (44.7)
Yes	5 (38.5)	32 (66.7)	13 (50.0)	13 (48.1)	63 (55.3)
Peritoneal metastasis					
No	9 (69.2)	36 (75.0)	21 (80.8)	22 (81.5)	88 (77.2)
Yes	4 (30.8)	12 (25.0)	5 (19.2)	5 (18.5)	26 (22.8)
Lymph node metastasis					
No	7 (53.8)	29 (60.4)	21 (80.8)	10 (37.0)	67 (58.8)
Yes	6 (46.2)	19 (39.6)	5 (19.2)	17 (63.0)	47 (41.2)
Carcinoembryonic antigen level					
<5 ng/mL	1 (7.7)	7 (14.6)	3 (11.5)	5 (18.5)	16 (14.0)
≥5 ng/mL	11 (84.6)	24 (50.0)	20 (76.9)	21 (77.8)	76 (66.7)
NA	1 (7.7)	17 (35.4)	3 (11.5)	1 (3.7)	22 (19.3)

Abbreviations: CAVE, Avelumab Plus Cetuximab in Pre-treated *RAS* Wild Type Metastatic Colorectal Cancer; CHRONOS, Rechallenge With Panitumumab Driven by *RAS* Dynamic of Resistance; CRICKET, Cetuximab Rechallenge in Irinotecan-pretreated mCRC, *KRAS*, *NRAS* and *BRAF* Wild-type Treated in 1st Line With Anti-EGFR Therapy; EGFR, epidermal growth factor receptor; NA, not available; VELO, Phase II Randomized Study Evaluating the Efficacy of Panitumumab (Vectibix) and Trifluridine-Tipiracil (Lonsurf) in Pretreated *RAS* Wild Type Metastatic Colorectal Cancer Patients.

SI conversion factor: To convert carcinoembryonic antigen to micrograms per liter, multiply by 1.0.

### Efficacy and Safety Analysis

**Figure 2.**
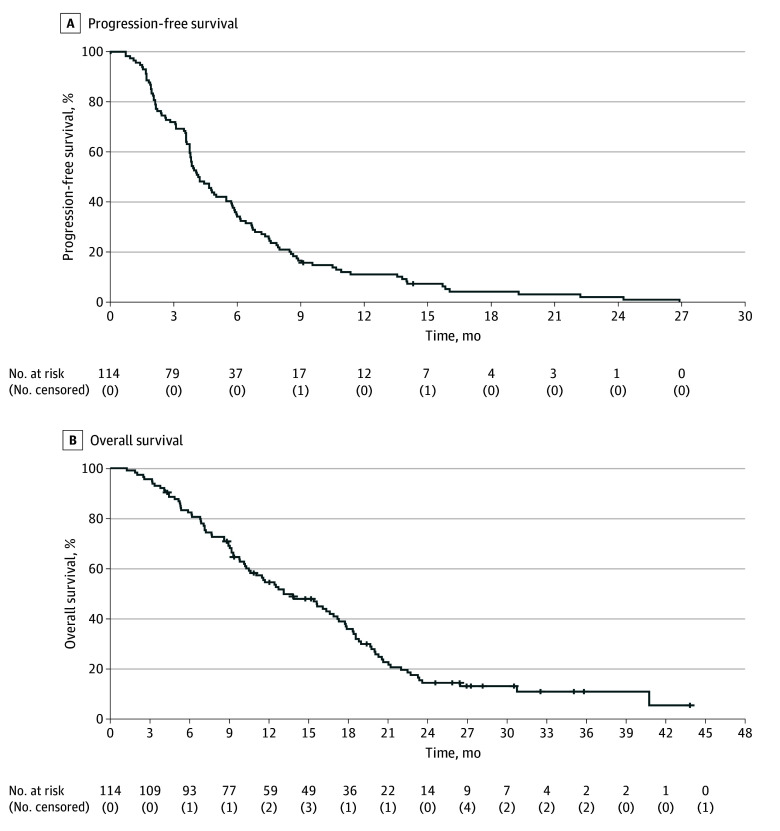
Kaplan-Meier Survival Curves for All Patients Graphs show progression-free survival (A) and overall survival (B). The median progression-free survival was 4.0 months (95% CI, 3.2-4.7 months), and the median OS was 13.1 months (95% CI, 9.5-16.7 months). Numbers in parentheses denote censored patients.

The median (IQR) follow-up was 28.1 (25.8-35.0) months. The ORR in the pooled population was 17.5% (20 patients), with 1 patient who achieved complete response and 19 patients who achieved partial response (Table 2). Stable disease was observed in 65 patients (57.0%). The DCR was 72.3% (82 patients). The median PFS was 4.0 months (95% CI, 3.2-4.7 months), and the median OS was 13.1 months (95% CI, 9.5-16.7 months) in the study population overall (Figure 2). Of note, a subset of patients experienced prolonged disease control upon anti-EGFR rechallenge therapy, with a 6-month PFS rate of 32.5% that led to an 18-month OS rate of 36.0% (eTable 1 in Supplement 2).

**Table 2.** Tumor Response of Patients With Metastatic Colorectal Cancer Receiving Anti–Epidermal Growth Factor Receptor Challenge Therapy in 4 Italian Trials

Study	Patients, No. (%)					
	Complete response	Partial response	Stable disease	Progressive disease	Overall response rate	Disease control rate
CAVE (n = 48)	1 (2.1)	3 (6.25)	31 (64.5)	13 (27.1)	4 (8.3)	35 (73.0)
VELO (n = 26)	0	3 (11.5)	18 (69.2)	5 (19.2)	3 (11.5)	21 (81.0)
CRICKET (n = 13)	0	5 (38.5)	5 (38.5)	3 (23.1)	5 (38.5)	10 (77.0)
CHRONOS (n = 27)	0	8 (30.0)	8 (30.0)	11 (41.0)	8 (30.0)	16 (59.3)
Pooled analysis (N = 114)	1 (0.9)	19 (16.7)	65 (57.0)	32 (28.0)	20 (17.5)	82 (72.3)

Abbreviations: CAVE, Avelumab Plus Cetuximab in Pre-treated *RAS* Wild Type Metastatic Colorectal Cancer; CHRONOS, Rechallenge With Panitumumab Driven by *RAS* Dynamic of Resistance; CRICKET, Cetuximab Rechallenge in Irinotecan-pretreated mCRC, *KRAS*, *NRAS* and *BRAF* Wild-type Treated in 1st Line With Anti-EGFR Therapy; VELO, Phase II Randomized Study Evaluating the Efficacy of Panitumumab (Vectibix) and Trifluridine-Tipiracil (Lonsurf) in Pretreated *RAS* Wild Type Metastatic Colorectal Cancer Patients.

**Figure 3.**
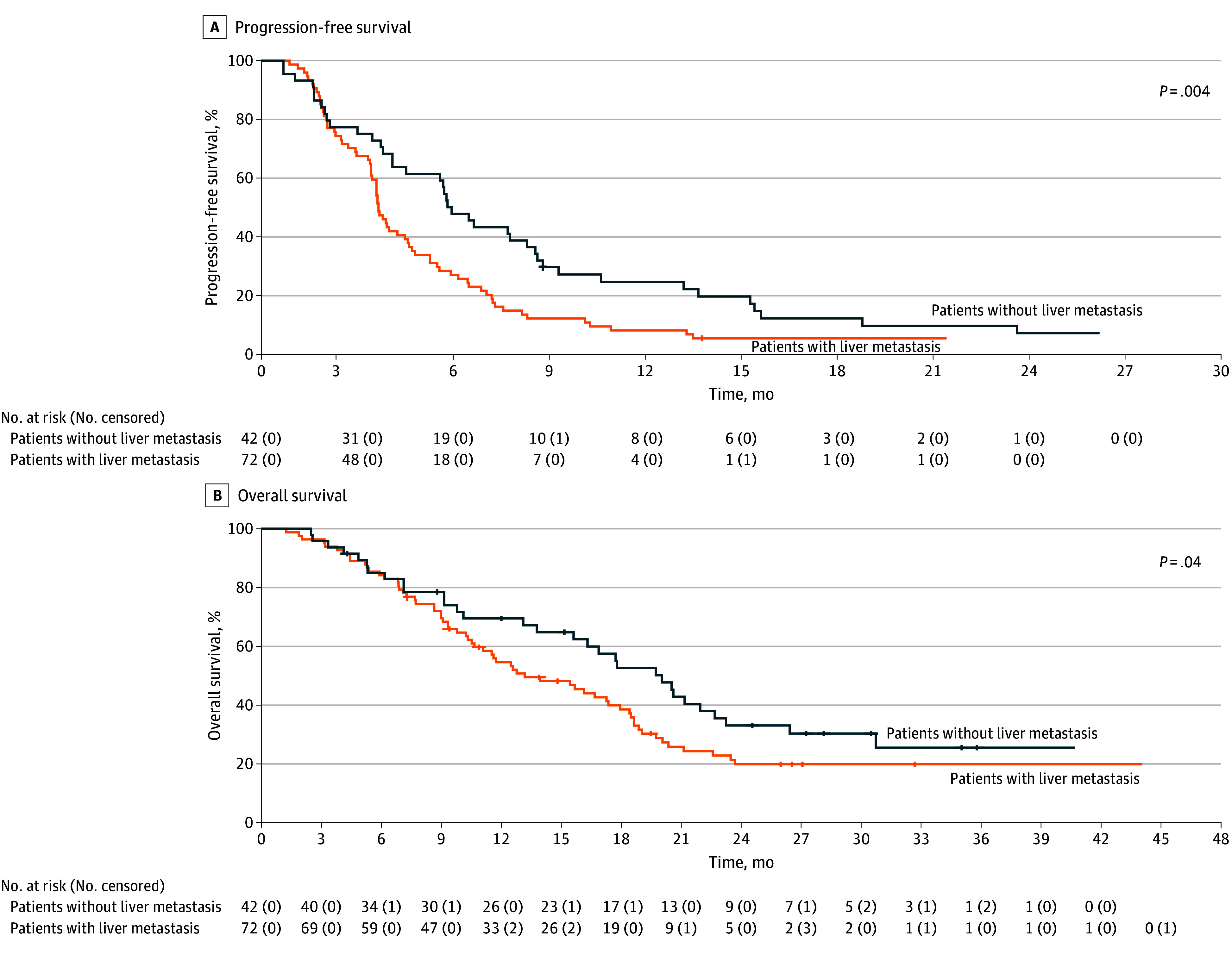
Kaplan-Meier Survival Curves According to the Presence of Liver Metastasis Graphs show progression-free survival (A) and overall survival (B). The median progression-free survival was 5.7 months (95% CI, 4.8-6.7 months) for patients without liver metastasis and 3.6 months (95% CI, 3.3-3.9 months) for patients with liver metastasis (hazard ratio, 0.56; 95% CI, 0.37-0.83). The median overall survival was 17.7 months (95% CI, 13.0-22.4 months) for patients without liver metastasis and 11.5 months (95% CI, 9.3-13.9 months) for patients with liver metastasis (hazard ratio, 0.63; 95% CI, 0.41-0.97). Numbers in parentheses denote censored patients.

Subsequently, we conducted an exploratory subgroup analysis to evaluate the association of several clinical variables (including performance status, number of previous line of treatments, tumor burden, resection of primary tumor, primary tumor sidedness, location of metastatic sites, and carcinoembryonic antigen) with treatment outcomes (eFigure 1 and eFigure 2 in Supplement 2). The absence of liver metastases was the only variable associated with improved PFS and OS. In the subgroup of patients without liver metastasis, the median PFS was 5.7 months (95% CI, 4.8-6.7 months) compared with 3.6 months (95% CI, 3.3-3.9 months) in patients with liver metastases (HR, 0.56; 95% CI, 0.37-0.83; *P* = .004) (Figure 3A). The median OS was 17.7 months (95% CI, 13-22.4 months) in patients without liver metastases compared with 11.5 months (95%, CI 9.3-13.9 months) in patients with liver metastases (HR, 0.63; 95% CI, 0.41-0.97; *P* = .04) (Figure 3B). Finally, in patients without liver involvement, the 12-month PFS rate was 21.0%, whereas the 30-month OS rate was 21.6% (eTable 2 in Supplement 2).

The safety profiles of the pooled analysis of the 4 clinical trials were in line with previous findings and were manageable (eTable 3 in Supplement 2). Skin rash (grade 3-4, 24 patients [21%]) and diarrhea (grade 3-4, 9 patients [8%]) were the most frequent adverse events related to the use of anti-EGFR mAbs. Grade 3 to 4 neutropenia was observed in 19 patients (17%), only among the 39 patients who received a backbone chemotherapy.

## Discussion

Over the last 2 decades, the identification of the molecular factors underlying disease and the clinical development of more effective treatments have led to substantial improvement in the treatment of patients with mCRC.^21^ Now, after progression to first-line and second-line therapies, more than one-half of patients maintain good performance status and are amenable to receiving further therapies.^22,23^

In the continuum of care for mCRC, even in later lines of treatment, fluoropyrimidine-based therapies and the blockade of angiogenesis are the main therapeutic choices.^24,25,26,27^ The use of the antiangiogenic drugs regorafenib and fruquintinib, or the chemotherapy compound trifluridine-tipiracil, is associated with a modest but significant clinical benefit compared with placebo.^24,26,27^ To date, SUNLIGHT is the only randomized phase 3 trial that demonstrated an improvement in both PFS and OS over an active treatment in patients with chemorefractory mCRC.^25^

In this scenario, novel and more effective therapeutic options are required. To answer to this unmet need, in this nonrandomized controlled trial, we conducted a pooled analysis of IPD from patients enrolled in 4 prospective Italian phase 2 studies that investigated different anti-EGFR rechallenge strategies.^9,11,12,13,14^ Because the presence of *RAS/BRAF* alterations at baseline liquid biopsy is associated with unresponsiveness to anti-EGFR mAbs, only patients with plasma ctDNA *RAS/BRAF* wt mCRC were included in this analysis. Here we provide evidence based on the largest data set available that anti-EGFR rechallenge therapy exerts antitumor activity.

The expected survival for patients with refractory mCRC who received trifluridine-tipiracil, regorafenib, or fruquintinib as single agents is approximately 7 to 9 months, whereas patients who were treated with trifluridine-tipiracil plus bevacizumab as third-line therapy had a median OS of 10.8 months.^22,24,26,27^ Furthermore, the available options display mainly a cytostatic activity (ORR, 1%-6%).^24,25,26,27^ With all the limitations for indirect cross-trial comparisons, in this study we report an OS longer than 12 months, with approximately one-third of the patients experiencing long survival (approximately 18 months).

In the study population, anti-EGFR rechallenge therapy was administered as third or later line of treatment. Interestingly, no difference in terms of survival was observed between patients according to the number of previous lines of therapies. Moreover, in this heavily pretreated population, anti-EGFR rechallenge achieved an ORR of 17.5% and a DCR of 72.3%. Thus, in a potential real clinical scenario, ctDNA-associated rechallenge with EGFR inhibitors could be considered following progression to trifluridine-tipiracil plus bevacizumab or an option as third-line treatment if tumor shrinkage is required. Further evidence regarding the optimal timing of anti-EGFR rechallenge therapy in the continuum of care of refractory mCRC will be provided by the results of the currently ongoing PARERE trial.^28^ Overall, more than 200 patients with ctDNA *RAS/BRAF* wt tumors will be randomly assigned to receive either panitumumab as third-line treatment followed by regorafenib at disease progression or the reverse sequence.

To better elucidate the role of potential factors involved in each treatment’s efficacy, we conducted an exploratory analysis investigating. The absence of liver metastasis was associated with a significantly longer median PFS and OS. Remarkably, 1 of 5 patients was still progression free at 12 months and alive beyond 30 months. Of course, owing to the nature of this subgroup analysis, these data require further confirmation by larger prospective trials. If confirmed, prospective translational and so-called multi-omics studies (ie, studies using data types derived from different research areas, such as genomics, epigenomics, transcriptomics, proteomics and metabolomics) are required to identify the subset of patients with liver metastases who could respond to anti-EGFR treatment.

### Limitations

Our study has several limitations that are intrinsic to its design. First, this pooled analysis has included patients who were enrolled in 4 phase 2 studies administering different therapies, which could have influenced clinical outcomes. In this respect, no evidence is currently available regarding the best anti-EGFR rechallenge regimen. To address this question, our group is currently conducting the CAVE-2 trial, a randomized phase 2 trial, that compares rechallenge with cetuximab plus avelumab vs cetuximab as single agent in patients with refractory plasma ctDNA *RAS/BRAF* wt microsatellite stable mCRC.^29^ Second, distinct liquid biopsy tests with different sensitivity thresholds and different panels were used for ctDNA analysis in the 4 trials. In the CHRONOS trial, a highly sensitive ddPCR was used, and only patients with 0 *RAS/BRAF/EGFR* ECD alteration in ctDNA were included.^12^ For the CAVE and VELO trials, the IdyllaTM Biocartis platform was used.^20^ Nevertheless, the issue of what is the real impact of a very low alteration allele fraction on anti-EGFR drug response is still debated.^30,31^ Third, alterations other than *RAS/BRAF*, such as *EGFR ECD*, *MAP2K1*, and *ERBB2* alterations or amplification, could constitute mechanisms of cancer cell resistance to EGFR blockade.^32,33,34,35,36^ Future trials using larger next-generation sequencing panels for patient selection will contribute to answer this question.^37^ Fourth, because of the single-group design of the CRICKET, CAVE, and CHRONOS trials and the reduced number of patients included in the control group of the VELO study, no direct comparison with other therapeutic options could be performed. Fifth, we were not able to evaluate the impact of the burden of hepatic disease (number and dimension of liver metastases) on treatment efficacy. These results should be considered as exploratory and hypothesis generating.

## Conclusions

In this pooled analysis of IPD from 4 phase 2 trials, anti-EGFR rechallenge therapy showed a promising antitumor activity in patients with refractory *RAS/BRAF* wt tumors as confirmed by liquid biopsy. Within the limitation of a subgroup analysis, the absence of liver metastases was associated with significantly improved survival. Further randomized studies are currently ongoing to confirm these results.

## Notes

Supplement 1.
Trial Protocols

Supplement 2. **eFigure 1.** Forest Plot of Progression Free Survival in Different Subgroup **eFigure 2.** Forest Plot of Overall Survival (OS) in Different Subgroup **eTable 1.** Progression Free and Overall Survival Rate in the Study Population **eTable 2.** Progression Free and Overall Survival Rate According to the Presence of Liver Metastasis **eTable 3.** Adverse Events

Supplement 3.
Data Sharing Statement
