# Error in Author Affiliation

**DOI:** 10.1001/jamanetworkopen.2024.18437

**Published:** 2024-05-22

In the Original Investigation titled “Anti-EGFR Rechallenge in Patients With Refractory ctDNA *RAS/BRAF* wt Metastatic Colorectal Cancer: A Nonrandomized Controlled Trial,”^1^ published April 9, 2024, coauthor Nicola Fazio’s affiliation was incorrect. It should have been Division of Gastrointestinal Medical Oncology and Neuroendocrine Tumors, European Institute of Oncology, IEO, IRCCS, Milan, Italy. This article has been corrected.^1^
