# Supplementary material for: Anti-EGFR Rechallenge in Patients With Refractory ctDNA RAS/BRAF wt Metastatic Colorectal Cancer: A Nonrandomized Controlled Trial
Source: JAMA Netw Open. 2024 Apr 9;7(4):e245635. doi: 10.1001/jamanetworkopen.2024.5635 (PMC11004834; doi:10.1001/jamanetworkopen.2024.5635)
Supplement: Supplement 1. — Trial Protocols [file jamanetwopen-e245635-s001.pdf]

Clinical Trial Protocol MS 100070-0028

Title: CAVE (*C*etuximab-*AVE*lumab) mCRC: A single arm phase IIclinical study of the combination of avelumab plus cetuximab in pre-treated RAS wild type metastatic colorectal cancer patients.

Short Trial Name: CAVE mCRC

EudraCT Number 2017-004392-32

Coordinating Investigator: Fortunato Ciardiello

Sponsor: Dipartimento di internistica clinica e sperimentale “Flaviano Magrassi”Università degli studi della Campania “Luigi Vanvitelli”

Table of Contents

Table of Contents
Table of Tables
Table of Figures
Listof Abbreviations
1 Synopsis
2 Sponsor, Investigators, and Trial Administrative Structure
2.1 Investigational Sites
3 Background Information
3.1 Metastatic colorectal cancer
3.2 Avelumab
3.3 Cetuximab
3.4 Epidermal growth factor receptor
3.5 Cetuximab general safety information
4 Trial Objectives
4.1 Primary objective
4.2 Secondary objectives
4.3 Exploratory objectives
4.4 Exploratory endpoints
5 Investigational Plan
5.2 Overall Trial Design and Plan
5.2.1 Trial Endpoints
5.2.2 Secondary Endpoints and Exploratory Endpoints
5.3 Trial Medication Administration and Schedule
5.3.1 Avelumab
5.3.2 Cetuximab
5.4 Dose Modification and Adverse Drug Reactions Requiring Treatment Discontinuation
5.4.1 Dose Modification for Avelumab
5.4.1.2 Adverse Drug Reactions Requiring Avelumab Discontinuation or Modification
5.4.1.3 Infusion-Related Reactions
5.4.1.4 Severe Hypersensitivity Reactions and Flu-Like Symtoms
5.4.1.5 Tumor Lysis Syndrome
5.4.1.6 Immune-Related Adverse Events
5.4.2 Dose Modification and Discontinuation for Cetuximab

5.4.2.1 Adverse Drug Reactions Requiring Cetuximab Discontinuation or Modification

5.4.2.2 Skin Toxicity

5.4.2.3 Allergic/Hypersensitivity Reactions

5.4.2.4 Infusion-Related Reactions

5.4.2.5 Other Considerations

5.5 Selection of Trial Population

5.5.1 Inclusion and Exclusion Criteria

5.6 Criteria for Subject Withdrawal

5.6.1 Criteria for Withdrawal from Trial Treatment

5.6.2 Withdrawal from the Trial

5.7 Premature Discontinuation of the Trial

5.8 Definition of End of Trial

6 Investigational Medicinal Product and Other Drugs Used in the Trial

6.1 Description of Investigational Medicinal Product

6.1.1 Avelumab

6.1.2 Cetuximab

6.2 Dosage and Administration

6.2.1 Avelumab Dosage and Administration

6.2.2 Cetuximab Dosage and Administration

6.3 Other Drugs to be Used in the Trial

6.4 Concomitant Medications and Therapies

6.4.1 Permitted Medicines

6.4.2 Nonpermitted Medicines

6.4.3 Other Considerations

6.5 Investigational Medicinal Products Formulation, Packaging and Storage

6.5.1 Avelumab

6.5.2 Cetuximab

6.6 Investigational Medicinal Product Accountability

6.7 Assessment of Investigational Medicinal Product Compliance

6.8 Treatment of Overdose

6.9 Medical Care of Subjects After End of Treatment

7 Trial Procedures and Assessments

7.1 Screening and Baseline Procedures and Assessments

7.2 Treatment Period

7.3	End of Treatment
7.4	Safety Follow-up
7.5	Long-term Follow-up
7.6	Demographic and Other Baseline Characteristics
7.6.1	Demographic Data
7.6.2	Diagnosis of mCRC
7.6.3	Medical History
7.6.4	Vital Signs and Physical Examination
7.6.5	CT or MRI Scans for Tumor Assessment at Baseline
7.6.6	Cardiac Assessments
7.6.7	Ophthalmologic Assessment
7.6.8	Clinical Laboratory Tests
7.7	Assessment of Efficacy
7.8	Assessment of Safety
7.9	Adverse Events
7.9.1	Adverse Event Definitions
7.9.2	Methods of Recording and Assessing Adverse Events
7.9.3	Definition of the Adverse Event Reporting Period
7.9.4	Procedure for Reporting Serious Adverse Events
7.9.5	Safety Reporting to Health Authorities, Independent Ethics Committees/Institutional Review Boards and Investigators
7.9.6	Monitoring of Subjects with Adverse Events
7.10	Pregnancy and In Utero Drug Exposure
7.11	Clinical laboratory assessments
7.12	Vital Signs, Physical Examination and Other Assessment
8.1	Primary Endpoint
8.2	Secondary Endpoints
8.2.1	Progression-Free Survival
8.2.2	Overall response rate (ORR)
8.2.3	Safety Endpoints
8.3	Description of Statistical Analyses
8.3.1	General Considerations
8.3.2	Analysis of Primary Endpoint
8.3.3	Analysis of Secondary Endpoints
9	Ethical and Regulatory Aspects
9.1	Responsibilities of the Investigator
9.2	Subject Information and Informed Consent

9.3	Subject Identification and Privacy
9.4	Emergency Medical Support and Subject Card
9.5	Clinical Trial Insurance and Compensation to Subjects
9.6	Independent Ethics Committee or Institutional Review Board
9.7	Health Authorities
10	Trial Management
10.1	Case Report Form Handling
10.2	Source Data and Subject Files
10.3	Investigator Site File and Archiving
10.4	Monitoring, Quality Assurance, and Inspection by Health Authorities
10.5	Changes to the Clinical Trial Protocol
10.6	Clinical Trial Report and Publication Policy
10.6.1	Clinical Trial Report
10.6.2	Publication
11	References
12	Appendices
Appendix I	Schedules of Assessments
Appendix II	Eastern Cooperative Oncology Group Performance Status

Table of Tables

Table 1	Most Frequently Reported Treatment-related TEAEs During Dose Expansion
Table 2	Prevalence of EGFR expression in common tumour types
Table 3	Treatment Modification for Symptoms of Infusion-Related Reactions Caused by Avelumab
Table 4	Management of Immune-mediated Adverse Reactions
Table 5	Treatment adjustment for Cetuximab caused Allergic/Hypersensitivity Reaction
Table 6	Required Full Laboratory Safety Tests
Table 7	Screening/Baseline, Treatment Phase and Follow-up procedures

Table of Figure

Figure 1 **Assessment and Initial Management of Tumor Lysis Syndrome**

Figure 2 **Treatment adjustment in case of Grade 3 skin toxicity considered to be related to Cetuximab**

List of abbreviations

1. Synopsis

**Title**	CAVE (Cetuximab-AVElumab) mCRC: A single arm phase II clinical study of the combination of avelumab plus cetuximab in pre-treated RAS wild type metastatic colorectal cancer patients.
**Short name trial**	CAVE mCRC
**EudraCTnumber**	2017-004392-32
**Sponsor**	Dipartimento di internistica clinica e sperimentale "Flaviano Magrassi"<br>Università degli studi della Campania "Luigi Vanvitelli"
**Phase**	PhaseII
**Trial Centers/Countries**	The study will be conducted in nine centers in Italy<br><br>1. Oncologia Medica, Università degli Studi della Campania "L. Vanvitelli", PI: Prof. Fortunato Ciardiello<br>2. Oncologia Medica, Istituto Nazionale per lo Studio e la Cura dei Tumori "Fondazione Giovanni Pascale" – IRCCS, Napoli, PI: Dr Antonio Avallone.<br>3. Oncologia Medica, Azienda Ospedaliera Universitaria, Università di Pisa. PI: Prof Alfredo Falcone<br>4. Oncologia Medica, Ospedale Casa Sollievo della Sofferenza - San Giovanni Rotondo (FG). PI: Dr Evaristo Maiello<br>5. Oncologia Medica, Nuovo Ospedale Garibaldi, Nesima, Catania. PI: Dr Roberto Bordonaro<br>6. Oncologia Medica,Campus Biomedico , Roma. PI: Prof Daniele Santini<br>7. Oncologia Medica, ASL Pescara. PI: Dr Carlo Garufi<br>8. Oncologia Medica,Istituto Nazionale dei Tumori di Milano. PI: Prof Filippo De Braud<br>9. Oncologia Medica, IRCCS Santa Maria Nuova. PI: Dr Carmine Pinto<br>10. Onco-Ematologia, Azienda Ospedaliera di Rilievo Nazionale "S.G. Moscati" Avellino. PI: Dr Gridelli Cesare
**Planned Trial period**<br>**(first enrollment-last subject out)**	36 months
**Trial objectives**	The primary objective of the study is to evaluate the efficacy (OS) of avelumab and cetuximab combinedin pre-treated RAS wild type metastatic colorectal cancer patients

	Secondaryobjectivewill be:<br>• To demonstrate superiority with regard to the objective response rate (ORR) of avelumab and cetuximab combined in pre-treated RAS wild type metastatic colorectal cancer patients.<br>• To demonstrate superiority with regard to progression free survival (PFS) of avelumab and cetuximab combined in pre-treated RAS wild type metastatic colorectal cancer patients.<br>• To determine the safety and tolerability of avelumab and cetuximab combinedin pre-treated RAS wild type metastatic colorectal cancer patients.
**Trial design and plan**	This isa non-profit phase II, open-label, single-arm study of cetuximab plus avelumabin patients with RAS WT mCRC treated in first line with chemotherapy in combination with an anti-EGFR drug that have had a clinical benefit (complete or partial response)from treatment.<br>Tumor measurements by computed tomography (CT) scan or magnetic resonance imaging (MRI) will be performed every 8 weeks from the beginning of treatment to determine response to treatment. Response will be evaluated using the Response Evaluation Criteria in Solid Tumors version 1.1 (RECIST 1.1).<br>Treatment will continue until disease progression, significant clinical deterioration, unacceptable toxicity, any criterion for withdrawal from the trial or trial drug is fulfilled. Treatment may continue past the initial determination of disease progression per RECIST 1.1 if the subject's performance status has remained stable, and if in the opinion of the Investigator, the subject will benefit from continued treatment and if other criteria are fulfilled as outlined in the protocol.
**Plannednumber of subjects**	75patients
**Schedule of visits and assessments**	**Screening/Baseline Assessments (day -28 from starting treatment)**<br><u>Screening procedures will include the following:</u><br>- Signing of the informed consent<br>- Collection of tumor tissue when available. Tumor tissue can be archival or resulting from a screening biopsy of the subject if no archival tissue is available (biopsies are only to be obtained from safely accessible tumor tissue/sites).<br>- Recording of the demographic information, complete medical history, and baseline medical condition<br>- A physical examination including vital signs, body weight, and height, 12-lead electrocardiogram (ECG), and a determination of the Eastern Cooperative Oncology Group Performance Status (ECOG PS)

	- Oftalmological assessment<br>- AE and concomitant medication assessments<br>- Safety laboratory assessments including free T4 and TSH<br>- Tumor evaluation by CT scan or MRI (a bone scan should be done at Screening as clinically indicated)<br>- Serum β-human chorionic gonadotropin (β-HCG) pregnancy test for females of childbearing potential<br>- Blood samples for hepatitis B virus (HBV) and hepatitis C virus (HCV) testing (local laboratory)<br><br>Treatment phase<br>Treatment phase begins the day of first infusion and ends when a decision is made to stop the trial drugs by the Investigator or when consent is withdrawn by the subject.<br>Visits will take place every week (-1/+1 days)<br><br>The main assessments are as follows:<br>• Tumor responses will be assessed every 8 weeks from starting treatment, per RECIST 1.1 while on trial.<br>• Vital signs will be collected prior to each trial drugs administration. Administration of trial drugs will take place only after relevant results have been checked by a medically qualified person.<br>• Blood chemistry and hematology assessments: must be performed at baseline, every two weeks prior to each avelumabplus cetuximab dose, at end of treatment visit and at 30 days post-treatment safety follow-up.<br>• Urine pregnancy test for women of childbearing potential must be performed at baseline and least every month during treatment.<br>• Free T4 and TSH must be performed at baseline and at least every 8 weeks during treatment and at end of treatment or 30 days post-treatment safety follow-up (if not performed in the previous 8 weeks).<br>• AEs and concomitant medications will be documented at each visit<br>Avelumab treatment will be administered by IV infusion once everytwo weeks whereas cetuximab treatment will be administrated by IV infusion once everyweekuntil disease progression, significant clinical deterioration (clinical progression), discontinuation for unacceptable toxicity, or withdrawal of consent.<br><br>Note: Treatment may continue past the initial determination of disease progression per RECIST 1.1 if the subject's ECOG PS has remained stable, and if in the opinion of the Investigator, the subject will benefit from continued treatment and if other

	criteria are fulfilled as outlined in the protocol, that is, no new symptoms or worsening of existing symptoms and no decrease in performance score.<br><br>Extended safety follow-up<br>• Given the potential risk for delayed immune-related toxicities, safety follow-up must be performed up to 90 days after the last dose of avelumab administration.<br>• The extended safety follow-up beyond 30 days after last avelumab administration may be performed either via a site visit or via a telephone call with subsequent site visit requested in case any concerns noted during the telephone call.<br><br>Discontinuation visit<br>Any subject who experiences an AE that mandates discontinuation of trial treatment should have a Discontinuation visit within 7 days of the decision to discontinue trial treatment.<br><br>Follow-up phase<br>The Follow-up phase starts when the decision has been made to stop trial drug treatment.<br>Subjects will have<br>- an End-of-Treatment visit at 28 days (± 5 days) after the last administration of trial treatment or before the start of any other antineoplastic therapy, and<br>- a Safety Follow-up visit 12 weeks (± 2 weeks) after the last administration of trial treatment.<br><br>After the End-of-Treatment visit only treatment related AEs have to be documented until the Safety Follow-up visit, defined as 12 weeks (± 2 weeks) after the last trial treatment administration.<br><br>Subjects with a serious AE (SAE) ongoing at the Safety follow-up visit must be followed up by the Investigator until stabilization or until outcome is known, unless the subject is documented as “lost to follow-up.”<br><br>Subjects who discontinue the trial treatment for reasons other than disease progression according to RECIST 1.1 will be followed up every 6 weeks (± 5 days) for radiographic assessment until disease progression, lost to follow-up, or withdrawal of informed consent.

	After the End-of-Treatment visit, subjects will be followed quarterly (that is, every 3 months ± 1 week) for survival (including assessment of any further tumor therapy). The survival follow-up will continue a maximum of 2 years after the last subject receives the last dose of avelumab and cetuximab. Subject-reported outcomes questionnaires will be assessed at the Early Discontinuation/End-of-Treatment visit.
**Diagnosis and main inclusion and exclusion criteria**	**Inclusion Criteria**<br>For inclusion in the trial, all of the following inclusion criteria must be fulfilled:<br>1. Signed written informed consent before any trial-related procedure is undertaken that is not part of the standard patient management<br>2. Male or female subjects aged ≥ 18 years<br>3.Histologically proven diagnosis of colorectal adenocarcinoma.<br>4.Diagnosis of metastatic disease<br>5. RAS (NRAS and KRAS exon 2,3 and 4) wild-type in tissue at initial diagnosis.<br>6. Efficacy of a first line therapy containing an anti-EGFR agent (panitumumab or cetuximab) with a major response achieved (complete or partial response).<br>7. A second line therapy.<br>8. More than 4 months from last dose of anti-EGFR agent administered in first line treatment before randomization.<br>9.Measurable disease according to RECIST criteria v1.1<br>10 ECOG PS of 0 to 1 at trial entry<br>11. Estimated life expectancy of more than 12 weeks<br>12. Adequate hematological function defined by white blood cell (WBC) count ≥ 2.5 × 10^9/L with absolute neutrophil count (ANC) ≥ 1.5 × 10^9/L, lymphocyte count ≥ 0.5 × 10^9/L, platelet count ≥ 100 × 10^9/L, and hemoglobin ≥ 9 g/dL (may have been transfused)<br>13. Adequate hepatic function defined by a total bilirubin level ≤ 1.5 × the upper limit of normal (ULN) range and AST and alanine aminotransferase (ALT) levels ≤ 2.5 × ULN for all subjects or AST and ALT levels ≤ 5 x ULN (for subjects with documented metastatic disease to the liver).<br>14. Adequate renal function defined by an estimated creatinine clearance > 30 mL/min according to the Cockcroft-Gault formula (or local institutional standard method)<br>15. Effective contraception for both male and female subjects if the risk of conception exists (Note: The effects of the trial drug on the developing human fetus are unknown; thus, women of childbearing potential and men must agree to use effective contraception, defined as 2 barrier methods, or 1 barrier method with a spermicide, an intrauterine device, or use of oral female contraceptive. Should a woman become

	pregnant or suspect she is pregnant while she or her partner is participating in this trial, the treating physician should be informed immediately.)<br>Highly effective contraception for both male and female subjects throughout the study and for at least 30 days after last avelumab treatment administration if the risk of conception exists.<br>16. No prior immunotherapy<br><br>**Exclusion Criteria**<br>Subjects are not eligible for this trial if they fulfill any of the following exclusion criteria:<br>1.Any contraindication to cetuximab and/or avelumab.<br>2.Past or current history of malignancies other than colorectal carcinoma, except for curatively treated basal and squamous cell carcinoma of the skin or in situ carcinoma of the cervix.<br>3.Pregnancy<br>4.Breastfeeding<br>5.Participation in a clinical study or experimental drug treatment within 30 days.<br>6.Subjects receiving immunosuppressive agents (such as steroids) for any reason should be tapered off these drugs before initiation of the trial treatment, with the exception of:<br>-subjects with adrenal insufficiency, who may continue corticosteroids at physiologic replacement dose, equivalent to ≤ 10 mg prednisone daily<br>-intranasal, inhaled, topical steroids,<br>-local steroid injection (e.g., intra-articular injection)<br>-Systemic corticosteroids at physiologic doses ≤ 10 mg/day of prednisone or equivalent<br>-Steroids as premedication for hypersensitivity reactions (e.g., CT scan premedication)<br>7.All subjects with brain metastases, except those meeting the following criteria:<br>-Brain metastases have been treated locally, and<br>-No ongoing neurological symptoms that are related to the brain localization of the disease (sequelae that are a consequence of the treatment of the brain metastases are acceptable)<br>8.Prior organ transplantation, including allogeneic stem-cell transplantation<br>9.Significant acute or chronic infections including, among others:<br>-Known history of testing positive test for human immunodeficiency virus (HIV) or known acquired immunodeficiency syndrome<br>-Hepatitis B virus (HBV) or hepatitis C virus (HCV) infection at screening (positive HBV surface antigen or HCV RNA if anti-

	HCV antibody screening test positive)<br>10.Active autoimmune disease that might deteriorate when receiving an immunostimulatory agent:<br>-Subjects with diabetes type I, vitiligo, psoriasis, hypo- or hyperthyroid disease not requiring immunosuppressive treatment are eligible<br>-Subjects requiring hormone replacement with corticosteroids are eligible if the steroids are administered only for the purpose of hormonal replacement and at doses ≤ 10 mg or equivalent prednisone per day.<br>-Administration of steroids through a route known to result in a minimal systemic exposure (topical, intranasal, intro-ocular, or inhalation) are acceptable.<br>-Active infection requiring systemic therapy.<br>11.Previous or ongoing administration of systemic steroids for the management of an acute allergic phenomenon is acceptable as long as it is anticipated that the administration of steroids will be completed in 14 days, or that the daily dose after 14 days will be ≤ 10 mg per day of equivalent prednisone.<br>12.Known severe hypersensitivity to investigational product or any component in its formulations, including known severe hypersensitivity reactions to monoclonal antibodies (NCI CTCAE v4.03 Grade ≥ 3), any history of anaphylaxis, or uncontrolled asthma (that is, 3 or more feauters of partially controlled asthma).<br>13.History of hypersensitivity to Polysorbate 80 that led to unacceptable toxicity requiring treatment cessation<br>14. Persisting toxicity related to prior therapy of Grade > 1 NCI-CTCAE v 4.03.<br>15. Known alcohol or drug abuse.<br>16.Clinically significant (that is active) cardioavscular disease: cerebral vascular accident/stroke (<6 months prior to enrollment), myocardial infarction (<6 months prior to enrollment), unstable angina, comgestive heart failure (New York Heart Association Classification Class≥II), or serious uncontrolled cardiac arrhytmia requiring medication<br>17.Other severe acute or chronic medical conditions including immune colitis, inflammatory bowel disease, immune pneumonitis, pulmonary fibrosis or psychiatric conditions including recent (within the past year) or active suicidal ideation or behavior; or laboratory abnormalities that may increase the risk associated with study participation or study treatment administration or may interfere with the interpretation of study results and, in the judgment of the investigator, would make the patient inappropriate for entry into this study.<br>18. Any psychiatric condition that would prohibit the understanding or rendering of informed consent.

	19.Vaccination within 4 weeks of the first dose of avelumab and cetuximab and while on treatment is prohibited except for administration of inactivated vaccine (i.e. inactivated influenza vaccine)<br>20. Legal incapacity or limited legal capacity.
**Investigational Medicinal Product: dose/mode of administration/dosing schedule**	Avelumab will be administered as a 1-hour IV infusion at 10 mg/kg once every 2-week treatment cycle.<br>Cetuximab will be administered at 1st dose at 400 mg/m^2 by i.v.infusionover 120 minutes, after avelumab infusion.<br>The 2nd dose and subsequent doses will be peroformed at 250 mg/ m^2 by i.v.infusionover 60 minutes, every week and after avelumab infusion every two weeks.<br>NB: Dilution is not required, but is possible in NaCl 0.9% only, via infusion pump or gravity drip.<br><br><u>Special Precautions for Administration:</u><br>• Premedication: In order to mitigate infusion related reactions, a premedication with an antihistamine and with paracetamol (acetaminophen) 30 to 60 minutes prior to the infusions of avelumab and cetuximab is mandatory (for example, 25 50 mg diphenhydramine and 500 650 mg paracetamol IV or oral). This may be modified based on local treatment standards and guidelines, as appropriate.<br>• Setting: Avelumaband cetuximab should be administered in a setting that allows for immediate access to an intensive care unit or equivalent environment and administration of therapy for anaphylaxis, such as the ability to implement immediate resuscitation measures. Steroids (dexamethasone 10 mg), epinephrine (1:1,000 dilution), allergy medications (IV antihistamines), bronchodilators, or equivalents, and oxygen should be available for immediate access and must be in place for use in the treatment of potential infusion-related reactions.<br>• Observation period: Following avelumab infusions, patients must be observed for 30 minutes post infusion for potential infusion related reactions.<br><br>The dose of avelumaband cetuximab will be calculated based on the weight and body surface, respectively, of the subject determined on the day prior to or the day of each drug administration.<br>Infusion of avelumab will be stopped in case of Grade ≥ 2 infusion-related, allergic, or hypersensitivity reactions (according to NCI-CTCAE v 4.03).<br><br>If the subject experiences an infusion-related reaction of Grade 2, the infusion rate of the subsequent administration of

	avelumab or cetuximab will be reduced by 50%. If a subject experiences a Grade 3 or 4 infusion-related reaction at any time, the subject must discontinue avelumab or cetuximab.<br><br>If the subject has a second infusion-related reaction Grade ≥ 2 on the slower infusion rate, the infusion should be stopped and the subject should be removed from treatment.
**Planned treatment duration per subject**	Subjects will receive trial treatment until progressive disease (PD) per RECIST 1.1, significant clinical deterioration (clinical progression), unacceptable toxicity, withdrawal of consent, or if any criterion for withdrawal from the trial or trial treatment is fulfilled. Treatment may continue past the initial determination of disease progression per RECIST 1.1 if the subject's ECOG PS has remained stable, and if in the opinion of the Investigator, the subject will benefit from continued treatment and if other criteria are fulfilled as outlined in the protocol, that is, no new symptoms or worsening of existing symptoms and no decrease in performance score.<br><br>Subjects receiving avelumab plus cetuximab who have experienced a CR should be treated for a maximum of 24 months after confirmation, at the discretion of the Investigator.If the Investigator believes that a subject may benefit from treatment beyond 24 months, it may be permissible after discussion with the Sponsor. In case a subject with a confirmed CR relapses after stopping treatment, but prior to the end of the trial, 1 re-initiation of treatment is allowed at the discretion of the Investigator and agreement of the Medical Monitor. In order to be eligible for re-treatment, the subject must not have experienced any toxicity that led to treatment discontinuation of the initial therapy. Subjects who re-initiate treatment will stay on trial and will be treated and monitored according to the protocol.
**Primaryendpoint**	The primary endpoint for the trial is OS time, defined as the interval from enrollment to death for every cause.
**Secondary/exploratoryendpoints**	Secondaryendpointswill be:<br>• The overall response rate (ORR) according to RECIST 1.1<br>• Progression free survival (PFS) according to RECIST 1.1<br>• The safety profile of the trial drugs as measured by the incidence of AEs, SAEs, clinical laboratory assessments, vital signs, physical examination, ECG parameters, and ECOG PS.
**Exploratoryendpoints**	Exploratoryendpoints are<br><br>• Duration of response of cetuximab plus avelumabaccording to RECIST 1.1<br>• Quantification PD-L1 expression levels in tumor cells

	and cells of the tumor microenvironment at baseline with their relation to selected clinical response parameters<br>• EGFR expression levels in tumor cells as candidate predictive biomarker with their relation to selected clinical response parameters<br>• Molecular, cellular, and soluble markers in peripheral blood and/or tumor tissue that may be relevant to the mechanism of action of, or response/resistance to avelumab and cetuximab
**Statistical methods (includes sample size calculation)**	To determine the potential efficacy on OS of the combination of avelumab and cetuximab we have considered the median OS that is obtained in third line in mCRC patients treated with standard third line therapy (CORRECT, RECOURSE). The current study aims to demonstrate a median OS of 11.0months (alternative hypothesis) by experimental combination for comparison with historical median OS 8.0(null hypothesis) with standard third line treatment, which correspond to an improvement of OS at 6 months from 35% to 46%. It was estimated that we would need to enroll 66 patients to achieve with a 1-sided 5% level test in this single stage, single arm trial. The accrual period will be of 18 months and the total duration of the stud will be of 36 months. Considering a potential drop-out of approximately 15% of patients a total of 75 patients will be recruited.

2. Sponsor, Investigators, and Trial Administrative Structure

The Sponsor of thisclinical trial with avelumaband cetuximab is the Dipartimento di internistica clinica e sperimentale “Flaviano Magrassi” Università degli studi della Campania “Luigi Vanvitelli”

2.1 Investigational Sites

The trial will be conducted in Italy in 9 Centers.

3. Background Information

3.1 Metastatic colorectal cancer

Colorectal cancer (CRC) is considered the third most commonly diagnosed cancer in males and the second in females worldwide, with an estimated 1.4 million new cases in 2012. In the same year, CRC was responsible for 693900 deaths, making it the fourth leading cause of cancer-related death in men and the third in women(1). Although the advances in screening and medical treatments have led a trend in reduction of both incidence and mortality, almost 20% of patients present metastases at the time of diagnosis, and approximately 35% of patients will subsequently develop a metastatic disease(2). The prognosis of patients with metastatic colorectal cancer (mCRC) has improved over the last 20 years, thanks to the introduction of active chemotherapy drugs and target therapies, such as fluropyrimidines, oxaliplatin, irinotecan, TAS-102, and of targeted drugs, such as bevacizumab, cetuximab, panitumumab, aflibercept, ramucirumab and regorafenib that led to an increase in median overall survival (OS) from 6 mo, with the only best supportive care (BSC), to approximately 30 mo(3,4).

The Epidermal Growth Factor Receptor (EGFR) targeted therapy with the monoclonal antibodies cetuximab or panitumumab represents a major step forward in the treatment of RAS wild type (WT) metastatic colorectal cancer (mCRC), given the relevant efficacy in terms of progression-free survival (PFS), overall survival (OS), response rate (RR), as well as quality of life (QoL), observed in several phase III clinical trials among different lines of treatment. However, the clinical benefit observed with these agents is limited to only a subset of patients and responses are often transient due to the development of various mechanisms of resistance. Several studies have provided new insights into molecular basis of EGFR inhibitors resistance and have identified mutations in KRAS, NRAS, BRAF and EGFR extracellular domain (ECD) as well as the amplification of ERBB2 and MET, as biomarkers of both primary and/or acquired resistance to these drugs. Unraveling the biology underlying the complex mechanisms of resistance have been useful for developing rational combination therapies in order to revert or overcome resistance. Rechallenge with an alternative anti-EGFRmonoclonal antibody (MoAb) after failure with an agent of the same family has been proposed as strategy to overcome drug resistance. However, panitumumab, as single agent, has demonstrated to provide minimal benefit in patients with KRAS WT mCRC who have experienced progression to cetuximab as prior therapy (5,6). The hypotheses that pre-existing sensitive subclones may emerge after treatment breaks with anti-EGFRmoAb has led the design of several clinical trials prospectively evaluating the rechallenge with anti-EGFRmoAbs in the third-line setting after a response to afirst-line therapy with anti-EGFR drugs (7). The immune system has a crucial role in modulating response to monoclonal antibody therapy in cancer, with novel agents inducing potent cytotoxicity and combinations with immune checkpoint inhibitors worth exploring in the anti-EGFR resistance setting. Avelumab is a fully human anti-PD-L1 IgG1 monoclonal antibody. By inhibiting PD-L1 interactions, avelumab is thought to enable the activation of T-cells and the adaptive immune system. By retaining a native Fc-region, avelumab is thought to potentially engage the innate immune system and induce antibody-dependent cell-

mediated cytotoxicity (ADCC) (8). In preclinical model, Cetuximab stimulates tumor antigen presentation through the formation of immune complexes,wich enhances the induction of tumor specific T cells. In an *in vitro* study using colon cancer cell lines, cetuximab promoted dendritic cell (DC) opsonization of tumor cells, and associated DC maturation with increased expression of MHC class II molecules, CD40, CD80 and CD 86. DCs incubated with tumor cells and cetuximab more effectively primed tumor-specific T cells than DCs that were incubated with tumor cells alone(9). Additionally, cetuximab facilitates NK cell-mediated antibody-dependent cellular cytotoxicity (ADCC) and complement-dependent cytoxicity(CDC), wich may further enhance tumor cell killing (10, 11).Cetuximab in combination with avelumab could be a valid therapeutic option for mCRCRAS WT patients that achieved a major response in the first line of therapy to panitumumab or cetuximab as third line rechallenge treatment.

3.2Avelumab

The Investigational Medicinal Product (IMP) for the present trial is avelumab (*avelumab is the proposed International Nonproprietary Name for the anti-PD-L1 monoclonal antibody MSB0010718C), a fully human monoclonal antibody of the immunoglobulin (Ig) G1 isotype. This anti-PD-L1 therapeutic antibody concept is being developed in oncological settings by Merck KGaA, Darmstadt, Germany, and by its subsidiary, EMD Serono R&D, Billerica, MA, USA.

Avelumab selectively binds to PD-L1 and competitively blocks its interaction with PD-1. Compared with anti-PD-1 antibodies that target T-cells, avelumab targets tumor cells, and therefore is expected to have fewer side effects, including a lower risk of autoimmune-related safety issues, as blockade of PD-L1 leaves the PD-L2 – PD-1 pathway intact to promote peripheral self-tolerance (22). For complete details of the in vitro and nonclinical studies, please refer to the Investigator’s Brochure.Avelumab is currently in clinical development with 2 ongoing Phase I studies in subjects with solid tumors and a Phase II trial in subjects with Merkel cell carcinoma:

- Trial EMR100070-001 is “a Phase I, open-label, multiple-ascending dose trial to investigate the safety, tolerability, pharmacokinetics, biological, and clinical activity of avelumab in subjects with metastatic or locally advanced solid tumors.” The most frequently reported treatment-related emergent adverse events (TEAEs) observed in subjects during the dose-expansion portion of the trial are presented in Table 1.

Table 1. Most Frequently Reported Treatment-related TEAEs During Dose Expansion

Treatment-emergent Adverse Events Grande ≤2 a Preferred Term (MedDRA)	Subjects (Safety Population, N = 480) N (%)	Treatment-emergent Adverse Events Grade ≥3 a Preferred Term (MedDRA)	Subjects (Safety Population, N = 480) N (%)
Fatigue	97 (20.2%)	Fatigue	5 (1.0%)
Nausea	62 (12.9%)	Anemia	5 (1.0%)
Infusion-related reaction	47 (9.8%)	Infusion-related reaction	4 (0.8%)
Chills	33 (6.9%)	Lipase increase	4 (0.8%)
Diarrhea	33 (6.9%)	GGT increase	4 (0.8%)
Decreased appetite	30 (6.3%)		
Pyrexia	27(5.6%)		
Influenza like illness	25 (5.2%)		
Arthralgia	24 (5.0%)		

MedDRA = Medical Dictionary for Regulatory Activities. a Only treatment-emergent adverse eventsstarted during the on-treatment period are summarized.

Serious Adverse Events

Overall, 176 of the 480 subjects (36.7%) treated during the dose expansion had serious TEAEs. Of these, 22 (4.6%) subjects reported dyspnea, which was the most frequent serious TEAE in this group, followed by 19 subjects (4.0%) reporting disease progression, 12 subjects (2.5%) reporting pleural effusion, 11 subjects (2.3%) reporting pneumonia, and 7 subjects (1.5%) reporting anemia. All other serious TEAEs were each reported in less than 1.5% of subjects. Of the serious TEAEs considered treatment-related by the Investigator (31subjects; 6.5%), the following were reported for 2 or more subjects: infusion-related reaction (4 subjects, 0.8%), pneumonitis (3 subjects, 0.6%), and disease progression, dyspnea, and hypercalcemia (each in 2subjects, 0.4%).

Deaths and withdrew from trial

In total, 134 subjects (27.9%) treated during the dose expansion died up to the cut-off date. Of these, the majority of deaths (101 deaths; 21.0%) were due to disease progression. A further 8deaths (1.7%) were due to TEAEs unrelated to trial treatment, 4 deaths (0.8%) were due to TEAEs related to trial treatment, and the reason for 8 deaths (1.7%) was labeled as other.

The reason for 13 deaths (2.7%) was unknown at the time of the data cut-off. Of the 134 subjects who died, 53subjects (11.0%) died within 30 days of the last administration of trial treatment. Among these deaths, 39 (8.1%) were due to disease progression, 7 (1.5%) were due to TEAEs unrelated to trial treatment, 4(0.8%) were due to TEAEs related to trial treatment, and 3(0.6%) were due to otherreasons. No death of unknown reason was reported in the 30-dayperiod. A total of 80 subjects (16.7%) treated during the dose expansion withdrew permanently from trial treatment due to one or more TEAE. In 25 (6.6%) of these subjects, the TEAEs leading to treatment discontinuation were considered related to trial treatment by the Investigator. These TEAEs were infusion-related reaction (6 withdrawals; 1.6%), GGT increased (3 withdrawals, 0.8%), dyspnea (3 withdrawals; 0.8%), and radiation pneumonitis, aspartate aminotransferase (AST) increased, hepatocellular injury, blood creatine phosphokinase increased, blood pressure increased, pneumonitis, anaphylactic reaction, food allergy, adrenal insufficiency, anemia, hypercalcemia, hyperglycemia, arthralgia, arthritis, myositis, pain, abdominal pain lower, chest discomfort, cramps and ache on back and all over body (not yet coded), encephalopathy, syncope, and flushing (1 withdrawal each; 0.3%). Most of the events of infusion-related reaction and anaphylactic reaction that led to permanent discontinuation of trial treatment (as described above) occurred before implementation of mandatory premedication on 28 January 2014.

Immunorelated (Ir) Adverse Events

A cumulative review revealed 56 cases of potential immune-related AEs of out of 480 subjects (11.7%) treated in the dose expansion part of trial EMR 100070-001 and 4 cases out of 50 subjects (8.0%) treated in the dose escalation part of trial EMR 100070-001.

Of 69 potential irAEs reported, 13 were SAEs (18.8%) and 56 were non-serious AEs (81.1%). In the majority of the cases, there was a plausible temporal association between the event onset and the drug administration. Of these 69 events, 46 events (66.7%) were assessed as treatment- related by the Investigator and 23 events (33.3%) were assessed as not treatment-related by the Investigator. Twenty-six events were assessed as Grade 1, 29 events as Grade 2, 11 events as

Grade 3, 2 events as Grade 4, and 1 event (pneumonitis) as Grade 5 (Please note: two more events of autoimmune hepatitis had a fatal outcome; however, they were assessed as Grade 3 with a consequent fatal liver failure). Based on the irAE cases that have been observed, all trial Investigators have been trained to be made aware of the frequency and severity of the observed events and to proactively administer steroid treatment for any suspicion of irAEs. Of note, irAEs are considered as an identified risk by the Sponsor.

Two suspected unexpected serious adverse reactions (SUSARs; anaphylactic reaction and infusion-related reaction) involving 2 subjects were reported in December 2013 and triggered a cumulative review of serious and non-serious cases of infusion-related reactions / hypersensitivity across the avelumab program. Following evaluation of safety signals, infusion-related reactions / hypersensitivity have been classified as a newly identified risk (previously classified as a potential risk) and a mandatory premedication regimen of a histamine H1 receptor (H1) blockers plus acetaminophen was implemented for all trial subjects as of 28 January 2014.

As of 05 November 2014, 49 (10.2%) of the 480 subjects in the expansion cohorts experienced at least 1 episode of an infusion-related reaction when receiving avelumab monotherapy. Most of the events were Grade 1 (8 subjects, 1.7%) or Grade 2 (36 subjects, 7.5%) in intensity, and Grade 3 (3 subjects, 0.6%) or Grade 4 events (2 subjects, 0.4%) were less frequent. No Grade 5 events were reported. Most of the infusion-related reaction events had an onset after the first (30 subjects, 6.3%) or second (16 subjects, 3.3%) avelumab infusion. In 8 subjects (1.7%), avelumab treatment was discontinued because of infusion-related reaction events.In addition, 1 subject (2.0%) in the dose escalation cohort reported an infusion-related reaction event (Grade 2).In addition to the aforementioned 49 subjects, 1 case of Grade 4 cardiac arrest occurred 1.5 hours after the third infusion of avelumab (10 mg/kg). The subject died due to an anoxic brain injury 7 days later; no autopsy was performed.Starting from 29 January 2014, the Sponsor has implemented a mandatory premedication with H1 blockers plus acetaminophen for all subjects who are to receive avelumab. This premedication procedure was applied to 28 and 440 subjects in the dose escalation and the pooled treatment expansion cohort, respectively. Under this premedication procedure, 33 of 440 subjects (7.5%) in the expansion cohort experienced infusion-related reaction events, with 6 subjects (1.4%) having Grade 1, 26 subjects (5.9%) having Grade 2, and 1 subject (0.2%) having Grade 3 events. No infusion-related reaction events were reported in the 28 subjects in the dose escalation cohort.Guidelines for the management of infusion-related reactions and severe hypersensitivity reaction according to the National Cancer Institute (NCI) are found in Section**5.4**. A complete guideline for the emergency treatment of anaphylactic reactions according to the Working Group of the Resuscitation Council (United Kingdom) can be found at https://www.resus.org.uk/pages/reaction.pdf.

Further information about the events described below is available in the current version of the Investigator's Brochure.

- Trial EMR100070-002 is "a Phase I trial to investigate the tolerability, safety, pharmacokinetics, biological, and clinical activity of avelumab in Japanese subjects with metastatic or locally advanced solid tumors, with expansion part in Asian subjects with gastric cancer."

- Trial EMR100070-003 is "a Phase II, open-label, multicenter trial to investigate the clinical activity and safety of avelumab in subjects with Merkel cell carcinoma."

3.3 Cetuximab

Cetuximab (ERBITUX®) has been approved and is available in the United States, European Union, Switzerland and over fifty countries worldwide. Cetuximab is a targeted therapeutic agent, a chimeric IgG1 monoclonal antibody that specifically binds to the EGFR with high affinity, internalising the receptor and preventing the ligands EGF and TGF-α from interacting with the receptors and thus effectively blocking ligand-induced EGFR phosphorylation (22). In addition, cetuximab has been found to potentiate the effects of chemotherapy and radiotherapy in experimental systems (23, 24).The dose of cetuximab (initial dose 400 mg/m^2 and subsequent weekly doses of 250 mg/m^2) has been found to be generally safe and effective in several studies in major tumor types expressing the EGFR. These included colorectal cancer, squamous cell carcinoma of the head and neck and non-small cell lung cancer, with cetuximab given either in combination with chemotherapy and/or radiotherapy or as monotherapy. The main side effects of cetuximab monotherapy are hypersensitivity and acne-like skin reactions.

3.4 Epidermal growth factor receptor

The EGFR is a transmembrane glycoprotein, which is commonly expressed, in many normal human tissues and solid human tumors (Table 2). It was one of several growth factors and their receptors, which were found to be encoded by proto-oncogenes. It is a member of the tyrosine kinase family of growth factor receptors, and is over-expressed in many human tumor types. The EGFR, when situated in the transmembrane position, has an extracellular domain, which provides a ligand-binding site for epidermal growth factor (EGF) and transforming growth factor alpha (TGFα). The intracellular domain of EGFR is activated upon ligand binding, which triggers the EGF-mediated tyrosine kinase signal transduction pathway and cascades many cellular operations concerning cell growth and division (25).

Analyses performed in vitro, using cell lines with a high degree of EGFR expression have shown a proliferation of cells in culture, probably due to activation via an autocrine pathway. In contrast, EGFR antagonists, which block the ligand-binding site, have been developed in order to inhibit proliferation of EGFR-expressing cells (26-28).

Table 2. Prevalence of EGFR expression in common tumour types

Tumour Type	EGFR Expression
Head and Neck	90 - 100%
Colon	75 – 89%
Prostate	Up to 100%
Pancreatic	Up to 95%
Breast	Up to 91%
Renal	Up to 90%
Cervix	Up to 82%
Non-Small Cell Lung Carcinoma	Up to 80%
Ovarian	Up to 77%
Bladder	Up to 72%
Primary Glioblastoma	Up to 63%

3.5 Cetuximab general safety information

Adverse event data are available for 3339 patients treated with cetuximab alone or in combination with chemotherapy and/or radiation therapy from investigational trials across all indications conducted by ImClone, BMS, Merck KGaA, Investigator sponsored Trials (IST), Cooperative Groups and the National Cancer Institute (NCI). As most of the trials were conducted under different settings and in combination with various cytostatic therapies, adverse reaction rates cannot be validly pooled and quoted as mean rates. Nevertheless, they constitute a basis for identifying approximate adverse event rates associated with the administration of Cetuximab.
Skin reactions may develop in more than 80% of patients and mainly present as acne-like rash and/or, less frequently, as pruritus, dry skin, desquamation, hypertrichosis, or nail disorders (e.g. paronychia). The majority of skin reactions develop within the first three weeks of therapy. They generally resolve, without sequelae, over time following cessation of treatment if the recommended adjustments in dose regimen are followed (29).Approximately 15% of the skin reactions are severe, including single cases of skin necrosis. In the event of Grade 3 or 4 skin reactions the patients should be referred for dermatological advice.
The incidence of radiation dermatitis of any grade was comparable between the treatment groups in a phase 3 SCCHN trial in patients receiving either cetuximab in combination with RT (86%) or RT alone (90%) (30)

Other side effects observed in patients receiving cetuximab monotherapy include asthenia, dyspnoea, mucositis, nausea, pain, fever and headache.
Mild or moderate infusion-related reactions may occur (≥ 1/10) comprising symptoms such as fever, chills, nausea, vomiting, headache, dizziness, or dyspnoea that occur in a close temporal relationship mainly to the first cetuximab infusion (29). They can be managed by slowing the infusion rate of cetuximab and by the continued use of pre- medications for subsequent doses in addition to the mandatory use for the first infusion.

Severe infusion-related reactions may occur (≥ 1/100, < 1/10), in rare cases with fatal outcome. They usually develop during or within 1 hour of the initial cetuximab infusion and may include symptoms such as rapid onset of airway obstruction (bronchospasm, stridor, hoarseness, difficulty in speaking), urticaria, hypotension, or loss of consciousness; in rare cases, angina pectoris, myocardial infarction or cardiac arrest have been observed. Severe infusion reactions (grade 3 or 4) require immediate interruption of the cetuximab infusion and permanent discontinuation from further treatment (29).
A large multinational study of cetuximab plus irinotecan in irinotecan-resistant metastatic colorectal cancer (MABEL) investigated, in a post-hoc analysis, whether the type of prophylactic pre-medication had an impact on the incidence of infusion-related reactions including allergic/hypersensitivity reactions. The incidence of infusion-related reactions was lower in patients who received anti-histamines and corticosteroids as prophylactic medication (9.6%, n=700) compared to patients who received anti-histamines but not corticosteroids (25.6%, n=422). A similar trend was seen in the analysis of the grade 3/4 infusion-related reactions (1% vs. 4.7%). These data suggest that the addition of corticosteroids to antihistamines as prophylactic pre-medication seems to reduce the incidence of infusion-related reactions such as allergic/-hypersensitivity reactions (31).

Progressively decreasing serum magnesium levels have been observed leading to severe hypomagnesaemia in some patients. Hypomagnesaemia is reversible following discontinuation of cetuximab. Depending on severity, other electrolyte disturbances, mainly hypocalcaemia or hypokalaemia, have also been observed. Determination of serum electrolyte levels is recommended

prior to and periodically during cetuximab treatment. Electrolyte repletion is recommended, as appropriate (29).

4. Trial Objectives

4.1 Primary

The primary objective of the study is to evaluate the efficacy (OS) of avelumab and cetuximab combined in pre-treated RAS wild type metastatic colorectal cancer patients

4.2 Secondary objectives

Secondary objectives are as follows:

- To demonstrate superiority with regard to the objective response rate (ORR) of avelumab and cetuximab combined in pre-treated RAS wild type metastatic colorectal cancer patients.
- To demonstrate superiority with regard to progression free survival (PFS) of avelumab and cetuximab combined in pre-treated RAS wild type metastatic colorectal cancer patients.
- To determine the safety and tolerability of avelumab and cetuximab combinedin pre-treated RAS wild type metastatic colorectal cancer patients

4.3 Exploratory objectives

Exploratory objectives are as follows:

- To determine duration of response of cetuximab plus avelumab
- To evaluate PD-L1 expression levels in tumor cells and cells of the tumor microenvironment (for example, infiltrating lymphocytes) as candidate predictive biomarker with their relation to selected clinical response parameters
- To characterize the immunogenicity of cetuximab plus avelumab
- To evaluate EGFR expression levels in tumor cells as candidate predictive biomarker with their relation to selected clinical response parameters
- To explore molecular, cellular, and soluble markers in peripheral blood and/or tumor tissue that may be relevant to the mechanism of action of, or response/resistance to avelumab and cetuximab

4.4 Exploratory endpoints

Exploratory endpoints are

- Duration of response of cetuximab plus avelumab according to RECIST 1.1
- Quantification of PD-L1 expression levels in tumor cells and cells of the tumor microenvironment at baseline with their relation to selected clinical response parameters
- EGFR expression levels in tumor cells as candidate predictive biomarker with their relation to selected clinical response parameters

- Molecular, cellular, and soluble markers in peripheral blood and/or tumor tissue that may be relevant to the mechanism of action of, or response/resistance to avelumab and cetuximab

The exploratory endpoint analyses will be performed on the ITT analysis set.

Duration of response will be analyzed descriptively by treatment arm. The Kaplan-Meier estimate of median time along with its 95% CI, as well as estimates of the survival function at 3, 6, and 12 months will be calculated for duration of response.

5. Investigational Plan

5.1 Overall Trial Design and Plan

This is a non-profit phase II, open-label, single-arm study of cetuximab plus avelumabin patients with RAS WT mCRC treated in first line with chemotherapy in combination with an anti-EGFR drug that have had a clinical benefit (complete or partial response) from treatment.

Approximately 75 subjects are planned to be enrolledin the Phase II to receive avelumab at a dose of 10 mg/kg once every 2 weeks plus cetuximab at a starting dose of 400 mg/m2 by i.v.infusion over 120 minutes at first dose and at the dose of 250 mg/ m2 by i.v.infusion over 60 minutes for subsequent infusions every week.

At the screening, when available, tumor tissue will be collected in order to analize the PD-L1 and EGFR expression by IHC (Appendix I, table 8)
During the Screening, and at progression the following collections will be performed:

- Blood and plasma samples will be collected from all subjects prior to infusion on Day1 (Week 1; Baseline samples for soluble factors may also be collected at Screening, instead of on Day 1 prior to dosing) and at progression (Appendix I, table 8).

Subjects will return to the clinic at regular intervals for assessments. Tumor measurements by computed tomography (CT) scan or magnetic resonance imaging (MRI) will be performed every 8 weeks to determine response to treatment. Response will be evaluated using the RECIST 1.1.

Treatment will continue until

- disease progression
- significant clinical deterioration
- unacceptable toxicity, or
- any criterion for withdrawal from the trial or trial drug is fulfilled (see Section 5.6).

Treatment may continue past the initial determination of disease progression according to RECIST 1.1 if the subject’s performance status has remained stable, and if in the opinion of the Investigator, the subject will benefit from continued treatment and if other criteria are fulfilled as outlined in the protocol, that is, no new symptoms or worsening of existing symptoms and no decrease in performance score.

Subjects who have experienced a complete response (CR) should be treated for a maximum of 24 months after confirmation, at the discretion of the Investigator. If the Investigator believes that a subject may benefit from treatment beyond 24 months, it may be permissible. In case a subject with a confirmed CR relapses after stopping treatment, but prior to the end of the trial, 1 re-initiation of treatment is allowed at the discretion of the Investigator. In order to be eligible for re-treatment, the subject must not have experienced any toxicity that led to treatment discontinuation of the initial avelumab plus cetuximab therapy. Subjects who re-initiate treatment will stay on trial and will be treated and monitored according to the protocol and the "until progression" schedule in the Schedule of Assessments (see Appendix I).

Subjects will attend clinic visits at regular intervals to receive trial treatment and for efficacy and safety assessments (see Section 7.2).

5.2 Trial Endpoints

5.2.1 Primary Endpoints

The primary endpoint for the trial is OS time, defined as the interval from enrollment to death for every cause.

5.2.2 Secondary Endpoints and Exploratory Endpoints

Secondary endpoints will be:

- The overall response rate (ORR) according to RECIST 1.1
- Progression free survival (PFS) according to RECIST 1.1
- Safety endpoints include AEs, assessed throughout the trial and evaluated using the NCI-CTCAE version 4.03 (CTCAE v 4.03), clinical laboratory assessments, vital signs, and electrocardiogram (ECG) parameters.

5.3 Trial Medication Administration and Schedule

The trial Schedule of Assessments is illustrated in Appendix I.

5.3.1 Avelumab

Subjects will receive IV infusion of avelumab (10 mg/kg over 1 hour) once every 2 weeks.

<u>Precautions for Administration:</u>

• Premedication: In order to mitigate infusion related reactions, a premedication with an antihistamine and with paracetamol (acetaminophen) 30 to 60 minutes prior to the first 4 infusions of avelumab is mandatory (for example, 25 50 mg diphenhydramine and 500 650 mg paracetamol IV or oral). Premedication should be administered for subsequent avelumab infusions based upon clinical judgment and presence/severity of prior infusion reactions. This may be modified based on local treatment standards and guidelines, as appropriate.

• Setting: Avelumab should be administered in a setting that allows for immediate access to an intensive care unit or equivalent environment and administration of therapy for anaphylaxis, such as the ability to implement immediate resuscitation measures. Steroids (dexamethasone 10 mg), epinephrine (1:1,000 dilution), allergy medications (IV antihistamines), bronchodilators, or equivalents, and oxygen should be available for immediate access.

• Observation period: Following avelumab infusions, patients must be observed for 30 minutes post infusion for potential infusion related reactions.

The formulation and packaging information of avelumab is provided in Sections 6.1.1 and 6.6, respectively.

5.3.2 Cetuximab

Cetuximab will be administered at 1st dose at 400 mg/m^2 by i.v.infusionover 120 minutes, directly afteravelumab.

The 2nd dose and subsequent doses will be performed at 250 mg/ m^2 by i.v.infusionover60 minutes, every week andafter by avelumab every two weeks.

NB: Dilution is not required, but is possible in NaCl 0.9% only, via infusion pump or gravity drip.

5.4 Dose Modification and Adverse Drug Reactions RequiringTreatment Discontinuation

5.4.1 Dose Modification for Avelumab

The dose of avelumab will be calculated based on the weight of the subject determined on the day prior to or the day of each drug administration.

Each subject will stay on the avelumab assigned dose of 10 mg/kg unless treatment needs to be stopped. There are to be no dose reductions.

Dosing modifications (changes in infusion rate) and dose delays are described in Sections 5.4 and 6.4.

5.4.1.2 Adverse Drug Reactions Requiring Avelumab Discontinuation or Modifications

The following adverse drug reactions (ADRs, see Section 7.9) require permanent treatment discontinuation of avelumab:

- Any Grade 4 ADRs require treatment discontinuation with avelumab except for single laboratory values out of normal range that are unlikely related to trial treatment as assessed by the Investigator, do not have any clinical correlate, and resolve within 7 days with adequate medical management.
- Any Grade 3 ADRs require treatment discontinuation with avelumab except for any of the following:

- Transient (≤ 6 hours) Grade 3 flu-like symptoms or fever, which is controlled with medical management
- Transient (≤ 24 hours) Grade 3 fatigue, local reactions, headache, nausea, or emesis that resolves to Grade ≤ 1
- Single laboratory values out of normal range (excluding Grade ≥ 3 liver function test increase) that are unlikely related to trial treatment according to the Investigator, do not have any clinical correlate, and resolve to Grade ≤ 1 within 7 days with adequate medical management
- Tumor flare phenomenon defined as local pain, irritation, or rash localized at sites of known or suspected tumor
- Change in Eastern Cooperative Oncology Group Performance Status (ECOG PS) to ≥ 3 that resolves to 2 within 14 days (infusions should not be given on the following cycle, if the ECOG PS is ≥ 3 on the day of trial drug administration)

Any Grade 2 ADR should be managed as follows:

- If a Grade 2 ADR resolves to Grade ≤ 1 by the last day of the current cycle, treatment may continue.
- If a Grade 2 ADR does not resolve to Grade ≤ 1 by the last day of the current cycle, infusions should not be given on the following cycle. If at the end of the following cycle the

event has not resolved to Grade 1, the subject should permanently discontinue treatment with aavelumab ADR (except for hormone insufficiencies, that can be managed by replacement therapy; for these hormone insufficiencies, up to 2 subsequent doses may be omitted).

- Upon the second occurrence of the same Grade 2 ADR (except for hormone insufficiencies that can be managed by replacement therapy) in the same subject, treatment with avelumab has to be permanently discontinued.

5.4.1.3 Infusion-Related Reactions

Infusion-related reactions, hypersensitivity reactions (Grades 1 to 4), tumor lysis syndrome, and irAEs should be handled according to guidelines in Section6.3.

A. **Symptoms**
- Fever
- Chills
- Rigors
- Diaphoresis
- Headache

B. **Management according to Table 3**

Table 3.Treatment Modification for Symptoms of Infusion-Related Reactions Caused by Avelumab

NCI-CTCAE Grade	Treatment Modification for Avelumab
**Grade 1 – mild**<br>Mild transient reaction; infusion interruption not indicated; intervention not indicated.	Decrease the avelumab infusion rate by 50% and monitor closely for any worsening.
**Grade 2 – moderate**<br>Therapy or infusion interruption indicated but responds promptly to symptomatic treatment (for example, antihistamines, NSAIDs, narcotics, IV fluids); prophylactic medications indicated for ≤ 24 h.	Temporarily discontinue avelumab infusion.<br>Resume infusion at 50% of previous rate once infusion-related reaction has resolved or decreased to at least Grade 1 in severity, and monitor closely for any worsening.
**Grade 3 or Grade 4 – severe or life-threatening**<br>Grade 3: Prolonged (for example, not rapidly responsive to symptomatic medication and/or brief interruption of infusion); recurrence of symptoms following initial improvement; hospitalization indicated for clinical sequelae.<br>Grade 4: Life-threatening consequences; urgent intervention indicated.	Stop avelumab infusion immediately and disconnect infusion tubing from the subject.<br>Subjects have to be withdrawn immediately from study avelumab and must not receive any further avelumab treatment.
- If avelumab infusion rate has been decreased by 50% or interrupted due to an infusion reaction, it must remain decreased for the next scheduled infusion. If no infusion reaction is observed in the next scheduled infusion, the infusion rate may be returned to baseline at the subsequent infusions based on investigator’s medical judgment.- If hypersensitivity reaction occurs, the subject must be treated according to the best available medical practice.	

IV=intravenous, NCI-CTCAE=National Cancer Institute-Common Terminology Criteria for Adverse Event, NSAIDs=nonsteroidal anti-inflammatory drugs.

5.4.1.4 Severe Hypersensitivity Reactions and Flu-Like Symtoms

If hypersensitivity reaction occurs, the subject must be treated according to the best available medical practice. A complete guideline for the emergency treatment of anaphylactic reactions according to the Working Group of the Resuscitation Council (United Kingdom) can be found at https://www.resus.org.uk/pages/reaction.pdf. Subjects should be instructed to report any delayed reactions to the Investigator immediately.

A. Symptoms

- Impaired airway
- Decreased oxygen saturation (<92%)
- Confusion
- Lethargy
- Hypotension
- Pale/clammy skin
- Cyanosis

B. Management

- Epinephrine injection and dexamethasone infusion
- Subject should be placed on monitor immediately
- Alert ICU for possible transfer if required

For prophylaxis of flu-like symptoms, 25 mg of indomethacin or comparable nonsteroidal anti-inflammatory drug (NSAID) dose (for example, ibuprofen 600 mg, naproxen sodium 500mg) may be administered 2 hours before and 8 hours after the start of each dose of avelumab IV infusion. Alternative treatments for fever (for example, paracetamol) may be given to subjects at the discretion of the Investigator.

5.4.1.5 TumorLysis Syndrome

Since avelumab can induce ADCC, there is a potential risk of tumor lysis syndrome. Should this occur, subjects should be treated per the local guidelines and the management algorithm published by Howard et al (36) (Figure 1)

Figure 1. Assessment and Initial Management of Tumor Lysis Syndrome

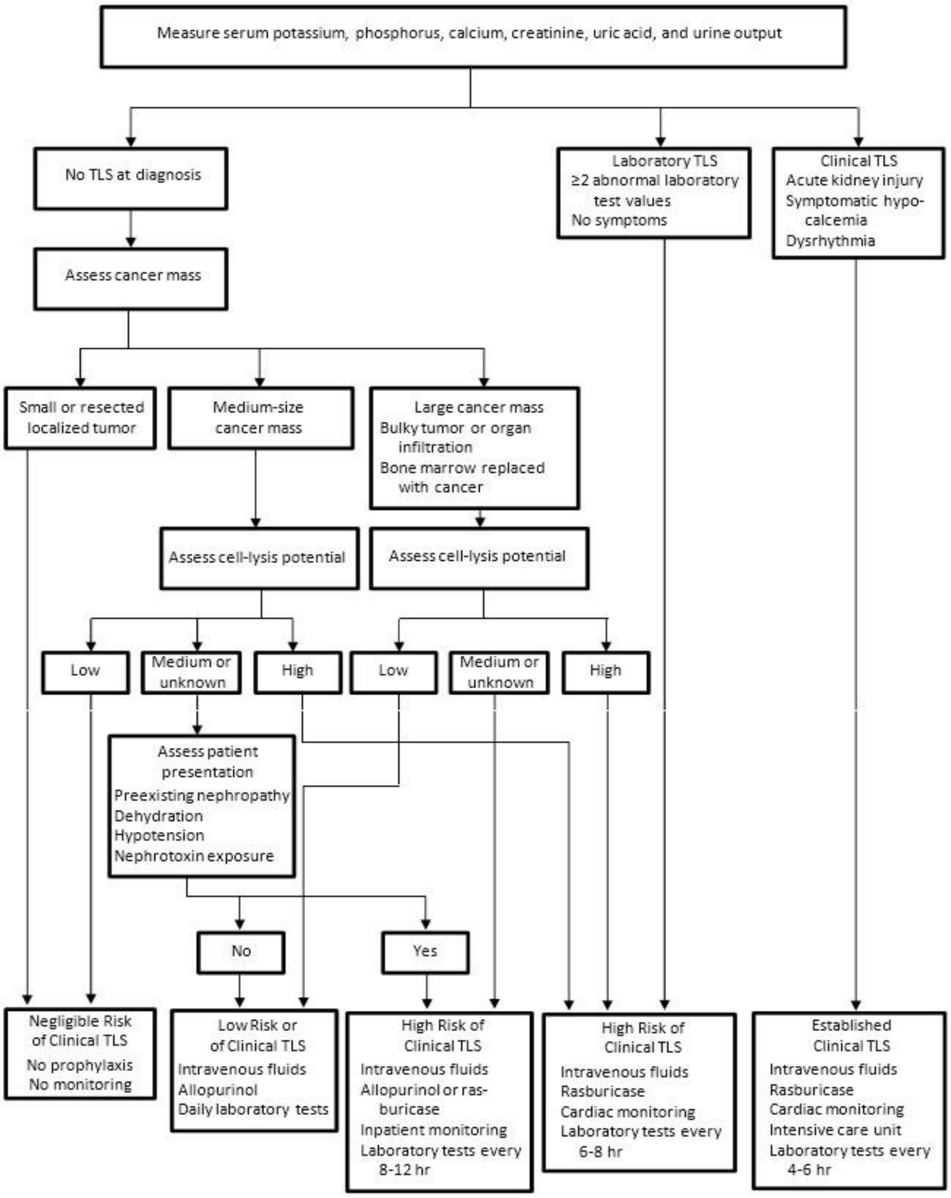

TLS=tumor lysis syndrome.

5.4.1.6 Immune-Related Adverse Events

Since inhibition of PD-L1 stimulates the immune system, irAEsmayoccur. Treatment of irAEs is mainly dependent upon severity (NCI-CTCAE grade):

- Grade 1 to 2: treat symptomatically or with moderate dose steroids, more frequent monitoring
- Grade 1 to 2 (persistent): manage similar to high grade AE (Grade 3 to 4)
- Grade 3 to 4: treat with high dose corticosteroids

Treatment of irAEs should follow guidelines set forth in the following Table 4

Table 4. Management of Immune-mediated Adverse Reactions

Gastrointestinal irAEs		
**Severity of Diarrhea/Colitis (NCI-CTCAE v4)**	**Initial Management**	**Follow-up Management**
**Grade 1**<br>Diarrhea: < 4 stools/day over Baseline<br>Colitis: asymptomatic	Continue avelumab therapy<br>Symptomatic treatment (e.g. loperamide)	Close monitoring for worsening symptoms<br>Educate subject to report worsening immediately<br>If worsens:<br>Treat as Grade 2, 3 or 4.
**Grade 2**<br>Diarrhea: 4 to 6 stools per day over Baseline; IV fluids indicated < 24 hours; not interfering with ADL<br>Colitis: abdominal pain; blood in stool	Withhold avelumab therapy<br>Symptomatic treatment	If improves to Grade ≤ 1:<br>Resume avelumab therapy<br><br>If persists> 5-7 days or recurs:<br>Treat as Grade 3 or 4.
**Grade 3 to 4**<br>Diarrhea (Grade 3): ≥ 7 stools per day over Baseline; incontinence; IV fluids ≥ 24 h; interfering with ADL<br>Colitis (Grade 3): severe abdominal pain, medical intervention indicated, peritoneal signs<br>Grade 4: life-threatening, perforation	Withhold avelumab for Grade 3.<br>Permanently discontinue avelumab for Grade 4 or recurrent Grade 3.<br><br>1.0 to 2.0 mg/kg/day prednisone IV or equivalent<br>Add prophylactic antibiotics for opportunistic infections<br>Consider lower endoscopy	If improves:<br>Continue steroids until Grade ≤ 1, then taper over at least 1 month; resume avelumab therapy following steroids taper (for initial Grade 3).<br><br>If worsens, persists > 3 to 5 days, or recurs after improvement:<br>Add infliximab 5mg/kg (if no contraindication). Note: infliximab should not be used in cases of perforation or sepsis.

Dermatological irAEs		
**Grade of Rash (NCI-CTCAE v4)**	**Initial Management**	**Follow-up Management**
**Grade 1 to 2**<br>Covering ≤ 30% body surface area	Continue avelumab therapy Symptomatic therapy (for example, antihistamines, topical steroids)	If persists > 1 to 2 weeks or recurs:<br>Withhold avelumab therapy<br>Consider skin biopsy<br><br>Consider 0.5-1.0 mg/kg/day prednisone or equivalent. Once improving, taper steroids over at least 1 month, consider prophylactic antibiotics for opportunistic infections, and resume avelumab therapy following steroids taper.<br>If worsens:<br>Treat as Grade 3 to 4.
**Grade 3 to 4**<br>Grade 3: Covering > 30% body surface area;<br>Grade 4: Life threatening	Withhold avelumab for Grade 3.<br>Permanently discontinue for Grade 4 or recurrent Grade 3.<br>Consider skin biopsy	If improves to Grade ≤ 1:<br>Taper steroids over at least 1 month; resume avelumab therapy following steroids taper (for initial Grade 3).

consequences	Dermatology consult<br>1.0 to 2.0 mg/kg/day prednisone or equivalent<br>Add prophylactic antibiotics for opportunistic infections	
	**Pulmonary irAEs**	
**Grade of Pneumonitis (NCI-CTCAE v4)**	**Initial Management**	**Follow-up Management**
**Grade 1**<br>Radiographic changes only	Consider withholding avelumab therapy<br>Monitor for symptoms every 2 to 3 days<br>Consider Pulmonary and Infectious Disease consults	Re-assess at least every 3 weeks<br>If worsens:<br>Treat as Grade 2 or Grade 3 to 4.
**Grade 2**<br>Mild to moderate new symptoms	Withhold avelumab therapy<br>Pulmonary and Infectious Disease consults<br>Monitor symptoms daily; consider hospitalization<br>1.0 to 2.0 mg/kg/day prednisone or equivalent<br>Add prophylactic antibiotics for opportunistic infections<br>Consider bronchoscopy, lung biopsy	Re-assess every 1 to 3 days<br>If improves:<br>When symptoms return to Grade ≤ 1, taper steroids over at least 1 month, and then resume avelumab therapy following steroids taper<br>If not improving after 2 weeks or worsening:<br>Treat as Grade 3 to 4.
**Grade 3 to 4**<br>Grade 3: Severe new symptoms; New/worsening hypoxia;<br>Grade 4: Life-threatening	Permanently discontinue avelumab therapy.<br>Hospitalize.<br>Pulmonary and Infectious Disease consults.<br>1.0 to 2.0 mg/kg/day prednisone or equivalent<br>Add prophylactic antibiotics for opportunistic infections<br>Consider bronchoscopy, lung biopsy	If improves to Grade ≤ 1:<br>Taper steroids over at least 1 month<br>If not improving after 48 hours or worsening:<br>Add additional immunosuppression (for example, infliximab, cyclophosphamide, IV immunoglobulin, or mycophenolatemofetil)
	**Hepatic irAEs**	
**Grade of Liver Test Elevation (NCI-CTCAE v4)**	**Initial Management**	**Follow-up Management**
**Grade 1**<br>Grade 1 AST or ALT > ULN to 3.0 x ULN and/or Total bilirubin > ULN to 1.5 x ULN	Continue avelumab therapy	Continue liver function monitoring<br>If worsens:<br>Treat as Grade 2 or 3 to 4.
**Grade 2**<br>AST or ALT > 3.0 to ≤ 5 x ULN and/or total bilirubin > 1.5 to ≤ 3 x ULN	Withhold avelumab therapy<br>Increase frequency of monitoring to every 3 days.	If returns to Grade ≤ 1:<br>Resume routine monitoring; resume avelumab therapy.<br>If elevation persists > 5 to 7 days or worsens:

		Treat as Grade 3 to 4.
**Grade 3 to 4**<br>AST or ALT > 5 x ULN and/or total bilirubin > 3 x ULN	Permanently discontinue avelumab therapy<br>Increase frequency of monitoring to every 1 to 2 days<br>1.0 to 2.0 mg/kg/day prednisone or equivalent<br>Add prophylactic antibiotics for opportunistic infections<br>Consult gastroenterologist/ hepatologist<br>Consider obtaining MRI/CT scan of liver and liver biopsy if clinically warranted	If returns to Grade ≤ 1:<br>Taper steroids over at least 1 month<br>If does not improve in > 3 to 5 days, worsens or rebounds:<br>Add mycophenolatemofetil 1 gram (g) twice daily<br>If no response within an additional 3 to 5 days, consider other immunosuppressants per local guidelines.

Renal irAEs

Grade of Creatinine Increased (NCI-CTCAE v4)	Initial Management	Follow-up Management
**Grade 1**<br>Creatinine increased > ULN to 1.5 x ULN	Continue avelumab therapy	Continue renal function monitoring<br>If worsens:<br>Treat as Grade 2 to 3 or 4.
**Grade 2 to 3**<br>Creatinine increased > 1.5 and ≤ 6 x ULN	Withhold avelumab therapy<br>Increase frequency of monitoring to every 3 days<br>1.0 to 2.0 mg/kg/day prednisone or equivalent.<br>Add prophylactic antibiotics for opportunistic infections<br>Consider renal biopsy	If returns to Grade ≤1:<br>Taper steroids over at least 1 month, and resume avelumab therapy following steroids taper.<br>If worsens:<br>Treat as Grade 4.
**Grade 4**<br>Creatinine increased > 6 x ULN	Permanently discontinue avelumab therapy<br>Monitor creatinine daily<br>1.0 to 2.0 mg/kg/day prednisone or equivalent.<br>Add prophylactic antibiotics for opportunistic infections<br>Consider renal biopsy<br>Nephrology consult	If returns to Grade ≤1:<br>Taper steroids over at least 1 month.

Cardiac irAEs

Myocarditis	Initial Management	Follow-up Management
New onset of cardiac signs or symptoms and / or new laboratory cardiac biomarker elevations (e.g. troponin, CK-MB, BNP) or cardiac imaging abnormalities suggestive of	Withhold avelumab therapy.<br>Hospitalize.<br>In the presence of life threatening cardiac decompensation, consider transfer to a facility experienced in advanced heart failure and arrhythmia management.	If symptoms improve and immune-mediated etiology is ruled out, re-start avelumab therapy.<br><br>If symptoms do not improve/worsen, viral myocarditis is

myocarditis.	Cardiology consult to establish etiology and rule-out immune-mediated myocarditis.<br>Guideline based supportive treatment as per cardiology consult.*<br><br>Consider myocardial biopsy if recommended per cardiology consult.	excluded, and immune-mediated etiology is suspected or confirmed following cardiology consult, manage as immune-mediated myocarditis.
Immune-mediated myocarditis	Permanently discontinue avelumab.<br>Guideline based supportive treatment as appropriate as per cardiology consult.*<br>1.0 to 2.0 mg/kg/day prednisone or equivalent<br>Add prophylactic antibiotics for opportunistic infections.	Once improving, taper steroids over at least 1 month.<br><br>If no improvement or worsening, consider additional immunosuppressants (e.g. azathioprine, cyclosporine A).
*Local guidelines, or eg. ESC or AHA guidelines<br>ESC guidelines website: https://www.escardio.org/Guidelines/Clinical-Practice-Guidelines<br>AHA guidelines website: http://professional.heart.org/professional/GuidelinesStatements/searchresults.jsp?q=&y=&t=1001		
**Endocrine irAEs**		
**Endocrine Disorder**	**Initial Management**	**Follow-up Management**
**Grade 1 or Grade 2 endocrinopathies (hypothyroidism, hyperthyroidism, adrenal insufficiency, type I diabetes mellitus)**	Continue avelumab therapy<br>Endocrinology consult if needed<br><br>Start thyroid hormone replacement therapy (for hypothyroidism), anti-thyroid treatment (for hyperthyroidism), corticosteroids (for adrenal insufficiency) or insulin (for Type I diabetes mellitus) as appropriate.<br><br>Rule-out secondary endocrinopathies (i.e. hypopituitarism / hypophysitis)	Continue hormone replacement/suppression and monitoring of endocrine function as appropriate.
**Grade 3 or Grade 4 endocrinopathies (hypothyroidism, hyperthyroidism, adrenal insufficiency, type I diabetes mellitus)**	Withhold avelumab therapy<br>Consider hospitalization<br>Endocrinology consult<br><br>Start thyroid hormone replacement therapy (for hypothyroidism), anti-thyroid treatment (for hyperthyroidism), corticosteroids (for adrenal insufficiency) or insulin (for type I diabetes mellitus) as appropriate.<br><br>Rule-out secondary endocrinopathies (i.e. hypopituitarism / hypophysitis)	Resume avelumab once symptoms and/or laboratory tests improve to Grade ≤ 1 (with or without hormone replacement/suppression).<br><br>Continue hormone replacement/suppression and monitoring of endocrine function as appropriate.
**Hypopituitarism/Hypophysitis (secondary endocrinopathies)**	If secondary thyroid and/or adrenal insufficiency is confirmed (i.e. subnormal serum FT4 with inappropriately low TSH and/or low serum cortisol with inappropriately low ACTH) :<br>• Refer to endocrinologist for dynamic testing as indicated and measurement of other hormones (FSH, LH, GH/IGF-1,	Resume avelumab once symptoms and hormone tests improve to Grade ≤ 1 (with or without hormone replacement).<br><br>In addition, for hypophysitis with abnormal MRI, resume avelumab only once shrinkage of the pituitary

	PRL, testosterone in men, estrogens in women)<br>• Hormone replacement/suppressive therapy as appropriate<br>• Perform pituitary MRI and visual field examination as indicated<br><br>**If hypophysitis confirmed:**<br>• Continue avelumab if mild symptoms with normal MRI. Repeat the MRI in 1 month<br>• Withhold avelumab if moderate, severe or life-threatening symptoms of hypophysitis and/or abnormal MRI. Consider hospitalization. Initiate corticosteroids (1 to 2 mg/kg/day prednisone or equivalent) followed by corticosteroids taper during at least 1 month.<br>• Add prophylactic antibiotics for opportunistic infections.	gland on MRI/CT scan is documented.<br><br>Continue hormone replacement/suppression therapy as appropriate.
**Other irAEs (not described above)**		
**Grade of other irAEs (NCI-CTCAE v4)**	**Initial Management**	**Follow-up Management**
**Grade 2 or Grade 3 clinical signs or symptoms suggestive of a potential irAE**	Withhold avelumab therapy pending clinical investigation	If irAE is ruled out, manage as appropriate according to the diagnosis and consider re-starting avelumab therapy<br>If irAE is confirmed, treat as Grade 2 or 3 irAE.
**Grade 2 irAE or first occurrence of Grade 3 irAE**	Withhold avelumab therapy<br>1.0 to 2.0 mg/kg/day prednisone or equivalent<br>Add prophylactic antibiotics for opportunistic infections<br>Specialty consult as appropriate	If improves to Grade ≤ 1:<br>Taper steroids over at least 1 month and resume avelumab therapy following steroids taper.
**Recurrence of same Grade 3 irAEs**	Permanently discontinue avelumab therapy<br>1.0 to 2.0 mg/kg/day<br>prednisone or equivalent<br>Add prophylactic antibiotics for opportunistic infections<br>Specialty consult as appropriate	If improves to Grade ≤ 1:<br>Taper steroids over at least 1 month.
**Grade 4**	Permanently discontinue avelumab therapy<br>1.0 to 2.0 mg/kg/day<br>prednisone or equivalent and/or other immunosuppressant as needed<br>Add prophylactic antibiotics for opportunistic infections<br>Specialty consult.	If improves to Grade ≤ 1:<br>Taper steroids over at least 1 month
**Requirement for 10 mg per day or greater prednisone or equivalent for more than 12 weeks for**	Permanently discontinue avelumab therapy<br>Specialty consult	

**reasons other than hormonal replacement for adrenal insufficiency**<br><br>**Persistent Grade 2 or 3 irAE lasting 12 weeks or longer**		

Abbreviations: ACTH=adrenocorticotropic hormone; ADL=activities of daily living; ALT=alanine aminotransferase; AST=aspartate aminotransferase; BNP=B-type natriuretic peptide; CK-MB=creatine kinase MB; CT= computed tomography; FSH=follicle-stimulating hormone; GH=growth hormone; IGF-1=insulin-like growth factor 1; irAE=immune related adverse event; IV=intravenous; LH=luteinizing hormone; MRI=magnetic resonance imaging; NCI CTCAE=National Cancer Institute Common Terminology Criteria for Adverse Events; PRL=prolactin;T4=thyroxine; TSH=thyroid stimulating hormone; ULN=upper limit of normal.

5.4.2 Dose Modification and Discontinuation for Cetuximab

The dose of Cetuximab will be calculated based on the Body Surface Area of the subject determined on the day prior to or the day of each drug administration. Each subject will stay on the Cetuximab assigned dose unless treatment modification needs to be performed. Dose modifications and dose delays are described in the following Section.

5.4.2.1 Adverse Drug Reaction Requiring Cetuximab Discontinuation or Modifications

5.4.2.1.2 Skin Toxicity

The most common AEs associated with Cetuximab administration are skin reactions, particularly acne-like rash. Skin reactions to Cetuximab may be considered as being in 3 phases. The first, early phase (1-4 weeks) is a moist acneiformphase which is usually responsive to tetracyclines and/or retinoids. Limiting sun exposure may be useful. Drying agents may be of value. The second phase is a dry skin phase, seldom troublesome, which usually responds well to emollients or petroleum jelly.

If started, drying agents should be discontinued. The third phase also included skin fissuring and nail changes. Skin fissures are best treated with cyanoacrylate or other adhesives and strong tape until healed.

If a patient experiences a Grade 3 skin toxicity (as defined in the US National Cancer Institute's - Common Toxicity Criteria - NCI-CTC- Version 3.0), Cetuximab therapy may be deferred for up to two consecutive infusions without changing the dose level. The investigator should also consider concomitant treatment with topical and/or oral antibiotics. Topical corticosteroids are not recommended. If the toxicity resolves to Grade 2 or less by the following treatment period, the treatment may resume.

With the second and third occurrences of a Grade 3 skin toxicity, Cetuximab therapy may again be deferred for up to two consecutive weeks with concomitant dose reductions to 200 mg/m^2 and 150 mg/m^2, respectively. Cetuximab dose reductions are permanent. Patients should discontinue Cetuximab if more than two consecutive infusions are withheld or a fourth occurrence of a Grade 3 skin toxicity occurs despite an appropriate dose reduction (Figure 2).

Figure 2. Treatment adjustment in case of Grade 3 skin toxicity considered to be related to Cetuximab.

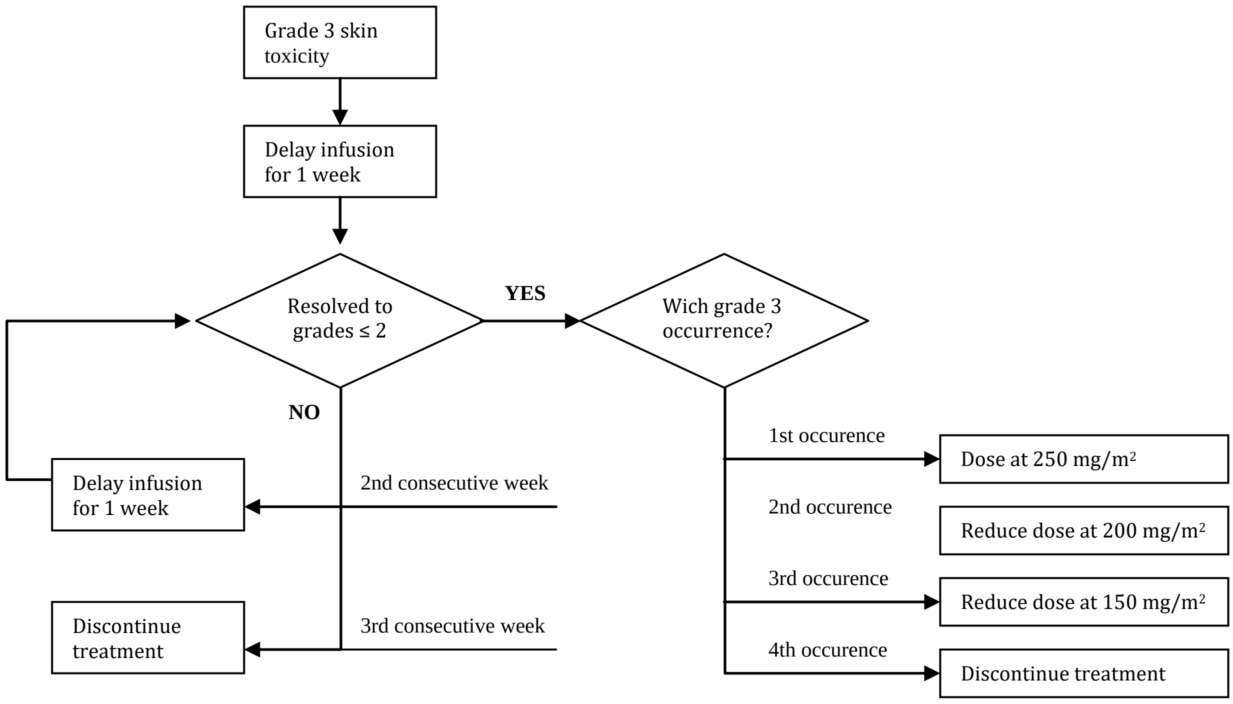

5.4.2.1.3 Allergic/Hypersensitivity Reactions

Allergic/hypersensitivity reactions may occur during or following the administration of Cetuximab. Patients must therefore be pretreated with an appropriate antihistamine and acetaminophen before infusions. If should an allergic/hypersensitivity or infusion reaction to Cetuximab occur, then the patient must be treated according to the best available medical practices. Grade 3 or 4 allergic/hypersensitivity reactions require immediate interruption of the Cetuximab infusion, appropriate medical measures and permanent discontinuation of treatment. Patients should be carefully monitored until the complete resolution of all signs and symptoms (Table 5).

Table 5. Treatment adjustment for Cetuximab caused Allergic/Hypersensitivity Reaction

CTC Grade	Treatment
**Grade 1**<br>Transient flushing or rash, drug fever <38ºC	Decrease the Cetuximab infusion rate by 50% and monitor closely for any worsening
**Grade 2**<br>Rash; flushing; urticaria; dyspnea; drugfever ≥ 38°C	Stop Cetuximab infusion<br>Administer bronchodilators, oxygen etc. as medically indicated<br>Resume infusion at 50% of previous rate once allergic/hypersensitivity reaction has resolved or decreased to grade 1 in severity, and monitor closely for any worsening
**Grade 3 or Grade 4**<br><br>Grade3:<br>Symptomatic bronchospasm, with or without urticaria;<br>parenteral medication(s)indicated;	 Stop Cetuximab infusion immediately and disconnect infusion tubing from the patient<br>Administer epinephrine, bronchodilators, antihistamines, glucocorticoids, intravenous fluids, vasopressor agents, oxygen etc., as

allergy-related edema/angioedema; hypotension<br><br>Grade 4: Anaphylaxis	medically indicated<br><br>Patients have to be withdrawn immediately from treatment and must not receive any further Cetuximab treatment

Once a Cetuximab infusion rate has been decreased due to an allergic/hypersensitivity reaction, it will remain decreased for all subsequent infusions. If the patient has a second allergic/hypersensitivity reaction with the slower infusion rate, the infusion should be stopped, and the patient should continue on chemotherapy alone. The patient must not receive any further cetuximab treatment.

If a subject experiences a Grade 3 or 4-allergic/hypersensitivity reaction at any time, Cetuximab should be discontinued.

5.4.2.1.4 Infusion-Related Reactions

Infusion reactions of Grade 1 and 2 severity may be treated with appropriate antihistamines, corticosteroid and by slowing the Cetuximab infusion rate to deliver the volume in 4 hours (but no longer). Infusion reactions of Grade 3 or higher should be managed with appropriate agents, including volume expanders, epinephrine, glucocorticoids and other agents as appropriate. Cetuximab should be immediately and permanentlydiscontinued.

5.4.2.1.5 Other Considerations

A. **Interstitial Pneumonitis**: severe interstitial pneumonitis has been described in subjects treated with the EGFR-pathway targeting therapy gefitinib. To date, no increased risk of interstitial pneumonitis has been identified with Cetuximab. Nevertheless, all subjects must have adequate chest imaging prior to commencing Cetuximab therapy in the study, as a safety precaution in order to document the baseline pulmonary condition. If there are respiratory symptoms at study entry, lung function tests and further diagnostic procedures must also be undertaken in order to diagnose pre-existing pulmonary fibrosis or interstitial pneumonitis. Furthermore, subjects will be regularly questioned about pulmonary symptoms during the study. Should pulmonary symptoms appear or worsen during or after Cetuximab treatment, a detailed description is required and investigators should use their discretion in ordering such diagnostic procedures as are necessary to elicit an accurate diagnosis.

B. **Electrolyte Disturbances**: progressively decreasing serum magnesium levels have been observed leading to severe hypomagnesemia in some patients. Hypomagnesaemia is reversible following discontinuation of cetuximab. Depending on severity, other electrolyte disturbances, mainly hypocalcaemia or hypokalaemia, have also been observed. Determination of serum electrolyte levels is recommended prior to and periodically during Erbitux ®treatment. Electrolyterepletionisrecommended, as appropriate.

C. **Other Reasons for Cetuximab Discontinuation**: if a patient develops an intercurrent illness (i.e., infection) that, in the opinion of the investigator mandates interruption of Cetuximab therapy, that intercurrent illness must resolve within a time frame such that no more than two, weekly, infusions are withheld. After the interruption of treatment, the subject will continue with a Cetuximab dose of 250 mg/m^2 every week at subsequent visits or the last dose before the interruption if there have been previous dose reductions

5.5Selection of Trial Population

Only persons meeting all inclusion criteria and no exclusion criteria may be enrolled into the trial as subjects. Prior to performing any trial assessments not part of the subject's routine medical care, the Investigator will ensure that the subject or the subject's legal representative has provided written informed consent following the procedure described in Section 9.2.

5.5.1 Inclusion and Exclusion Criteria

Inclusion Criteria

For inclusion in the trial, all of the following inclusion criteria must be fulfilled:

1. Signed written informed consent before any trial-related procedure is undertaken that is not part of the standard patient management
2. Male or female subjects aged ≥ 18 years
3.Histologically proven diagnosis of colorectal adenocarcinoma.
4.Diagnosis of metastatic disease
5. RAS (NRAS and KRAS exon 2,3 and 4) wild-type in tissue at initial diagnosis.
6. Efficacy of a first line therapy containing an anti-EGFR agent (panitumumab or cetuximab) with a major response achieved (complete or partial response).
7. A second line therapy.
8. More than 4 months from last dose of anti-EGFR agent administered in first line treatment before randomization.
9.Measurable disease according to RECIST criteria v1.1
10 ECOG PS of 0 to 1 at trial entry
11. Estimated life expectancy of more than 12 weeks
12. Adequate hematological function defined by white blood cell (WBC) count ≥ 2.5 × 10^9/L with absolute neutrophil count (ANC) ≥ 1.5 × 10^9/L, lymphocyte count ≥ 0.5 × 10^9/L, platelet count ≥ 100 × 10^9/L, and hemoglobin ≥ 9 g/dL (may have been transfused)
13. Adequate hepatic function defined by a total bilirubin level ≤ 1.5 × the upper limit of normal (ULN) range and AST and alanine aminotransferase (ALT) levels ≤ 2.5 × ULN for all subjects or AST and ALT levels ≤ 5 x ULN (for subjects with documented metastatic disease to the liver).
14. Adequate renal function defined by an estimated creatinine clearance > 30 mL/min according to the Cockcroft-Gault formula (or local institutional standard method)
15. Effective contraception for both male and female subjects if the risk of conception exists (Note: The effects of the trial drug on the developing human fetus are unknown; thus, women of childbearing potential and men must agree to use effective contraception, defined as 2 barrier methods, or 1 barrier method with a spermicide, an intrauterine device, or use of oral female contraceptive. Should a woman become pregnant or suspect she is pregnant while she or her partner is participating in this trial, the treating physician should be informed immediately.)
Highly effective contraception for both male and female subjects throughout the study and for at least 30 days after last avelumab treatment administration if the risk of conception exists.
16. No prior immunotherapy

Exclusion Criteria

Subjects are not eligible for this trial if they fulfill any of the following exclusion criteria:

1.Any contraindication to cetuximab and/or avelumab.
2.Past or current history of malignancies other than colorectal carcinoma, except for curatively treated basal and squamous cell carcinoma of the skin or in situ carcinoma of the cervix.

3.Pregnancy
4.Breastfeeding
5.Participation in a clinical study or experimental drug treatment within 30 days.
6.Subjects receiving immunosuppressive agents (such as steroids) for any reason should be tapered off these drugs before initiation of the trial treatment, with the exception of:
-subjects with adrenal insufficiency, who may continue corticosteroids at physiologic replacement dose, equivalent to ≤ 10 mg prednisone daily
-intranasal, inhaled, topical steroids,
-local steroid injection (e.g., intra-articular injection)
-Systemic corticosteroids at physiologic doses ≤ 10 mg/day of prednisone or equivalent
-Steroids as premedication for hypersensitivity reactions (e.g., CT scan premedication)
7.All subjects with brain metastases, except those meeting the following criteria:
-Brain metastases have been treated locally, and
-No ongoing neurological symptoms that are related to the brain localization of the disease (sequelae that are a consequence of the treatment of the brain metastases are acceptable)
8.Prior organ transplantation, including allogeneic stem-cell transplantation
9.Significant acute or chronic infections including, among others:
-Known history of testing positive test for human immunodeficiency virus (HIV) or known acquired immunodeficiency syndrome
-Hepatitis B virus (HBV) or hepatitis C virus (HCV) infection at screening (positive HBV surface antigen or HCV RNA if anti-HCV antibody screening test positive)
10.Active autoimmune disease that might deteriorate when receiving an immunostimulatory agent:
-Subjects with diabetes type I, vitiligo, psoriasis, hypo- or hyperthyroid disease not requiring immunosuppressive treatment are eligible
-Subjects requiring hormone replacement with corticosteroids are eligible if the steroids are administered only for the purpose of hormonal replacement and at doses ≤ 10 mg or equivalent prednisone per day.
-Administration of steroids through a route known to result in a minimal systemic exposure (topical, intranasal, intro-ocular, or inhalation) are acceptable.
-Active infection requiring systemic therapy.
11.Previous or ongoing administration of systemic steroids for the management of an acute allergic phenomenon is acceptable as long as it is anticipated that the administration of steroids will be completed in 14 days, or that the daily dose after 14 days will be ≤ 10 mg per day of equivalent prednisone.
12.Known severe hypersensitivity to investigational product or any component in its formulations, including known severe hypersensitivity reactions to monoclonal antibodies (NCI CTCAE v4.03 Grade ≥ 3), any history of anaphylaxis, or uncontrolled asthma (that is, 3 or more feauters of partially controlled asthma).
13.History of hypersensitivity to Polysorbate 80 that led to unacceptable toxicity requiring treatment cessation
14.Persisting toxicity related to prior therapy of Grade > 1 NCI-CTCAE v 4.03.
15. Known alcohol or drug abuse.
16. Clinically significant (that is active) cardioavscular disease: cerebral vascular accident/stroke (<6 months prior to enrollment), myocardial infarction (<6 months prior to enrollment), unstable angina, comgestive heart failure (New York Heart Association Classification Class≥II), or serious uncontrolled cardiac arrhytmia requiring medication

17.Other severe acute or chronic medical conditions including immune colitis, inflammatory bowel disease, immune pneumonitis, pulmonary fibrosis or psychiatric conditions including recent (within the past year) or active suicidal ideation or behavior; or laboratory abnormalities that may increase the risk associated with study participation or study treatment administration or may interfere with the interpretation of study results and, in the judgment of the investigator, would make the patient inappropriate for entry into this study.
18. Any psychiatric condition that would prohibit the understanding or rendering of informed consent.
19. Vaccination within 4 weeks of the first dose of avelumab and cetuximab and while on treatment is prohibited except for administration of inactivated vaccine (i.e. inactivated influenza vaccine)
20. Legal incapacity or limited legal capacity.

5.6Criteria for Subject Withdrawal

5.6.1 Criteria for Withdrawal from Trial Treatment

Subjects will be withdrawn from trial treatment for any of the following reasons:

- PD per RECIST 1.1 (subjects receiving avelumab plus cetuximab treatment may continue past the initial determination of disease progression if the subject's ECOG PS has remained stable, and if in the opinion of the Investigator, the subject will benefit from continued treatment)
- Significant clinical deterioration (clinical progression), defined as new symptoms that are deemed by the Investigator to be clinically significant or significant worsening of existing symptoms
- Unacceptable toxicity
- Withdrawal of consent
- Occurrence of an exclusion criterion, which is clinically relevant and affects the subject's safety, if discontinuation is considered necessary by the Investigator and/or Sponsor
- Therapeutic failure requiring urgent additional drug (if applicable)
- Occurrence of any Grade ≥ 3 ADRs or repetitive Grade 2 ADRs as defined in Section 5.4
- Occurrence of AEs, resulting in the discontinuation of the trial drug being desired or considered necessary by the Investigator and / or the subject
- Occurrence of pregnancy
- Use of a nonpermitted concomitant drug, as defined in Section 6.4 if considered necessaryby the Investigator or Sponsor
- Noncompliance

5.6.2 Withdrawal from the Trial

Subjects are free to discontinue the trial at any time without giving their reasons.

A subject must be withdrawn in the event of any of the following:

- Withdrawal of the subject's consent
- Participation in any other therapeutic trial during the treatment duration of this trial; however, subjects will continue to be followed for survival

If a subject fails to attend scheduled trial assessments, the Investigator must determine the reasons and the circumstances as completely and accurately as possible.

In case of withdrawal from the trial, the assessments scheduled for the last visit (End-of-Treatment visit) should be performed (see Section 7.3), if possible, with focus on the most relevant assessments. In any case, the appropriate End-of-Treatment electronic case report form (eCRF) visit must be completed. In case of withdrawal, subjects will be asked to continue safety and survival follow-up, which includes the collection of data on survival, subject-reported outcomes and subsequent anticancer therapy.
If a subject is withdrawn prior to progression for any reason, the subject will not be replaced.

5.7 Premature Discontinuation of the Trial

The whole trial may be discontinued prematurely in the event of any of the following:

- New information leading to unfavorable risk-benefit judgment of the trial drug, for example, due to
- evidence of inefficacy of the trial drug,
- occurrence of significant previously unknown adverse reactions or unexpectedly high

intensity or incidence of known adverse reactions, or

- otherunfavorablesafetyfindings.

(Note: Evidence of inefficacy may arise from this trial or from other trials; unfavorable safety findings may arise from clinical or non-clinical examinations, for example, toxicology.)

- Sponsor’s decision that continuation of the trial is unjustifiable for medical or ethical reasons
- Poor enrollment of subjects making completion of the trial within an acceptable time frame

Unlikely

- Discontinuation of development of the Sponsor’s trial drug

Health Authorities and Independent Ethics Committees (IECs)/Institutional Review Boards (IRBs) will be informed about the discontinuation of the trial in accordance with applicable regulations.
The whole trial may be terminated or suspended upon request of Health Authorities.

5.8 Definition of End of Trial

If the trial is not terminated for a reason given in Section 5.8, the survival follow-up will continue until 2 years after the last subject receives the last dose of avelumab plus cetuximab.

6 Investigational Medicinal Product and Other Drugs Used in the Trial

In this trial, the investigational drugs areavelumaband cetuximab.

6.1Description of Investigational Medicinal Product

6.1.1 Avelumab

Avelumab is a sterile, clear, and colorless solution intended for IV administration. It is presented at a concentration of 20 mg/mL in single-use glass vials closed with a rubber stopper and sealed with an aluminum polypropylene flip-off seal.

6.1.2 Cetuximab

Cetuximab (Erbitux®): concentrate for solution is provided in vials of 5 mg/ml (European registered number EU/1/04/281/005, further information on: http://www.emea.europa.eu/)

6.2 Dosage and Administration

6.2.1 Avelumab Dosage and Administration

Subjects will receive an IV infusion of avelumab at a dose of 10 mg/kg (over the duration of 1 hour) following pretreatment with H1 blockers and acetaminophen 30 to 60 minutes prior to each avelumab infusion, once every 2 weeks (refer to Appendix I). Premedication with an antihistamine and with paracetamol (acetaminophen) approximately 30 to 60 minutes prior to each dose of avelumab is mandatory (for example, 25 50 mg diphenhydramine and 500-650 mg paracetamol [acetaminophen] IV or oral equivalent). Modifications of the infusion rate due to infusion-related reactions are described in Section 5.4. The dose of avelumab will be calculated based on the weight of the subject determined on the day prior to or the day of each drug administration. Complete blood count and core chemistry samples must be drawn and results reviewed within 48 hours prior to dose administration. Subjects will receive avelumab once every 2 weeks until the criteria in Sections 5.7 through 5.9 are met.

Treatment may continue past the initial determination of disease progression per RECIST 1.1 as long the following criteria are met:

- Investigator-assessed clinical benefit, without any rapid disease progression
- Tolerance of trial drug
- Stable ECOG PS
- Treatment beyond progression will not delay an imminent intervention to prevent serious complications of disease progression (for example, central nervous system metastases).

The decision to continue treatment should be discussed with the Medical Monitor and documented in the trial records.
A radiographic assessment should be performed within 6 weeks of original PD to determine whether there has been a decrease in the tumor size, or continued PD. The assessment of clinical benefit should be balanced by clinical judgment as to whether the subject is clinically deteriorating and unlikely to receive any benefit from continued treatment with avelumab plus cetuximab.
If the Investigator feels that the subject continues to achieve clinical benefit by continuing treatment, the subject should remain on the trial and continue to receive monitoring according to the Schedule of Assessments (Appendix I).
For subjects who continue avelumab plus cetuximab trial therapy beyond progression, further progression is defined as an additional 10% increase in tumor burden volume from time of initial PD. This includes an increase in the sum of all target lesions and/or the development of new measurable lesions. Treatment should be discontinued permanently upon documentation of further disease progression.
New lesions are considered measureable at the time of initial progression if the longest diameter is at least 10 mm (except for pathological lymph nodes, which must have a short axis of at least 15 mm). Any new lesion considered nonmeasureable at the time of initial progression may become measureable and therefore included in the tumor burden volume if the longest diameter increases to at least 10 mm (except for pathological lymph nodes, which must have a short axis of at least 15 mm).
Additionally, subjects receiving avelumab who have experienced a CR should be treated for a maximum of 24 months after confirmation, at the discretion of the Investigator. If the Investigator believes that a subject may benefit from treatment beyond 24 months, it may be permissible after discussion with the Sponsor. In case a subject with a confirmed CR relapses after stopping treatment, but prior to the end of the trial, 1 re-initiation of treatment is allowed at the discretion

of the Investigator. In order to be eligible for re-treatment, the subject must not have experienced any toxicity that led to treatment discontinuation of the initial avelumab therapy. Subjects who re-initiate treatment will stay on trial and will be treated and monitored according to the protocol and the "until progression" schedule in the Schedule of Assessments (see Appendix I).

6.2.2 Cetuximab Dosage and Administration

Cetuximab will be administered at 1st dose at 400 mg/m^2 by i.v.infusionover 120 minutes, directly afteravelumab.

The 2nd dose and subsequent doses will be performed at 250 mg/ m^2 by i.v.infusionover60 minutes, every week andafter by avelumab every two weeks.

NB: Dilution is not required, but is possible in NaCl 0.9% only, via infusion pump or gravity drip.

6.3 Other Drugs to be Used in the Trial

Subjects will receive pretreatment with H1 blockers and acetaminophen 30 to 60 minutes prior to each avelumab and cetuximab infusion. Premedication with an antihistamine and with paracetamol (acetaminophen) approximately 30 to 60 minutes prior to each dose of avelumaband cetuximab is mandatory (for example, 25 50 mg diphenhydramine and 500-650 mg paracetamol [acetaminophen] IV or oral equivalent). This regimen may be modified based on local treatment standards and guidelines as appropriate.

Immediate access to an Intensive Care Unit (ICU) or equivalent environment and appropriate medical therapy (including epinephrine, corticosteroids, IV antihistamines, bronchodilators, and oxygen) must be available for use in the treatment of infusion-related reactions.

Infusion of avelumab or cetuximab will be stopped in case of Grade ≥ 2 infusion-related, allergic, or anaphylactoid reactions. Following drug infusions, subjects must be observed for 2 hours post infusion for potential infusion-related reactions.

As with all monoclonal antibody therapies, there is a risk of allergic reaction. Avelumaband cetuximab should be administered in a setting that allows for immediate access and administration of therapy for severe allergic/hypersensitivity reactions, such as the ability to implement immediate resuscitation measures. Steroids (dexamethasone 10 mg), epinephrine (1:1000 dilution), allergy medications (antihistamines), or equivalents should be available for immediate access.

If hypersensitivity reaction occurs, the subject must be treated according to the best available medical practice. Guidelines for management of infusion-related reactions and severe hypersensitivity and flu-like symptoms according to the NCI are found in Sections 5.4.

A complete guideline for the emergency treatment of anaphylactic reactions according to the Working Group of the Resuscitation Council (United Kingdom) can be found at https://www.resus.org.uk/pages/reaction.pdf. Subjects should be instructed to report any delayed reactions to the Investigator immediately.

6.4 Concomitant Medications and Therapies

6.4.1 Permitted Medicines

Any medications (other than those excluded by the clinical trial protocol) that are considered necessary for the subjects' welfare and will not interfere with the trial drug may be given at the Investigator's discretion.

Other drugs to be used for prophylaxis, treatment of hypersensitivity reactions, and treatment of fever or flu-like symptoms are described in Section 5.4.

The Investigator will record all concomitant medications taken by the subject during the trial, from the date of signature of informed consent, in the appropriate section of the eCRF.
Any additional concomitant therapy that becomes necessary during the trial and any change to concomitant drugs must be recorded in the corresponding section of the eCRF, noting the name, dose, duration, and indication of each drug.
Palliative bone-directed radiotherapy may be administered during the trial. The assessment of PD will be made according to RECIST 1.1 (40) and not based on the necessity for palliative bone directed-radiotherapy.

6.4.2 Nonpermitted Medicines

As stated for the exclusion criteria in Section 5.6.1, subjects must not have had concurrent anticancer treatment (for example, cytoreductive therapy, radiotherapy [with the exception of palliative bone-directed radiotherapy, or radiotherapy administered on superficial lesions], major surgery (excluding prior diagnostic biopsy), concurrent systemic therapy with steroids or other immunosuppressive agents, or use of any investigational drug within 28 days before starting treatment.
In addition, the following treatments must not be administered during the trial:

- Immunotherapy, immunosuppressive drugs (that is, chemotherapy or systemic corticosteroids except for short-term treatment of allergic reactions or for the treatment of irAEs), or other experimental pharmaceutical products.

- Short-term administration of systemic steroid (that is, for allergic reactions or the management of irAEs is allowed)

- Growth factors (granulocyte colony stimulating factor or granulocyte macrophage colony stimulating factor). Exception: Erythropoietin and darbepoietin alpha may be prescribed at the Investigator’s discretion
- Bisphosphonate or denosumab treatment is not allowed unless it has been initiated more than 14 days prior to receiving the first administration of study drugs
- Vaccination within 4 weeks of the first dose of study drugs and while on trial is prohibited except for administration of inactivated vaccines (for example,inactivated influenza vaccines)

If the administration of a nonpermitted concomitant drug becomes necessary during the trial, the subject will be withdrawn from trial treatment.
Medications other than those specifically excluded in this trial (see above) may be administered for the management of symptoms associated with the administration of avelumab or cetuximab as required. These might include analgesics, antinausea medications, antihistamines, diuretics, anti-anxiety medications, and medication for pain management, including narcotic agents. Any additional concomitant therapy that becomes necessary during the trial and any change to concomitant drugs must be recorded in the corresponding section of the eCRF, noting the name, dose, duration, and indication of each drug.

6.4.3Other Considerations

The following nondrug therapies must not be administered during the trial (or within 28 days before starting treatment):
- Major surgery (excluding prior diagnostic biopsy)

- Herbal remedies with immunostimulating properties (for example, mistletoe extract) or known to potentially interfere with major organ function (for example,hypericin)
- Subjects should not abuse alcohol or other drugs during the trial

6.5 Investigational Medicinal Products Formulation, Packaging and Storage

6.5.1 Avelumab

Avelumab is formulated as a 20.0 mg/mL solution and is supplied by Merck SeronoKGaAin single-use glass vials, stoppered with a rubber septum and sealed with an aluminum polypropylene flip-off seal.

Packaging and labeling will be in accordance with applicable local regulatory requirements and applicable GMP guidelines. Avelumab will be packed in boxes each containing 1 vial. The information on the trial drugwill be in accordance with approved submission documents.
Avelumab will be shipped in transport cool containers (2°C to 8°C) that are monitored with temperature control devices.

The contents of the avelumab vials are sterile and nonpyrogenic, and do not contain bacteriostatic preservatives. Any spills that occur should be cleaned up using the facility's standard cleanup procedures for biologic products.

Avelumab drug product must be stored at 2°C to 8°C until use, with a temperature log maintained daily. All medication boxes supplied to each trial site must be stored carefully, safely, and separately from other drugs.
Avelumab drug product stored at room temperature (23°C to 27°C) or at elevated temperatures (38°C to 42°C) for extended periods is subject to degradation. Avelumab must not be frozen. Rough shaking of avelumab must be avoided.

For application in this trial, avelumab drug product must be diluted with 0.9% saline solution (sodium chloride injection). Avelumab must not be used for any purpose other than the trial.
The administration of trial drug to subjects who have not been enrolled into the trial is not covered by the trial insurance. Any unused portion of the solution should be discarded in biohazard waste disposal with final disposal by accepted local and national standards of incineration.

6.5.2 Cetuximab

Cetuximab will be supplied for the study by Merck SeronoKGaA. Cetuximab will be packed in boxes (with the required details concerning vial number, batch number, retest date, study number) and will be sent to study sites.

All treatment boxes supplied to the study centers must be stored carefully, safely, and separately from other drugs. Cetuximab must be stored under refrigeration at +2ºC to +8ºC. Do not freeze Cetuximab. Rough shaking of Cetuximab must be avoided.
For application in this trial, cetuximab drug product not require dilution but it is possible only with 0.9% saline solution (sodium chloride injection) via infusion pump or gravity drip. Cetuximab must not be used for any purpose other than the trial.
The administration of trial drug to subjects who have not been enrolled into the trial is not covered by the trial insurance. Any unused portion of the solution should be discarded in

biohazard waste disposal with final disposal by accepted local and national standards of incineration.

6.6 Investigational Medicinal Product Accountability

The Investigator is responsible for ensuring accountability for trial drug (avelumab or cetuximab), including reconciliation of drugs and maintenance of drug records.
Upon receipt of trial drug, the Investigator (or designee) will check for accurate delivery and acknowledge receipt by signing (or initialing) and dating the documentation provided by the Sponsor and returning it to the Sponsor. A copy will be retained for the Investigator File.
The dispensing of the trial drug will be carefully recorded on the appropriate drug accountability forms provided by the Sponsor and an accurate accounting will be available for verification by the Sponsor'sl Monitor at each monitoring visit.
Trial drug accountability records will include:

- confirmation of trial drug delivery to the trial site;
- the inventory at the site of trial drug provided by the Sponsor and prepared at the site;
- the use of each dose by each subject;
- the return to the Sponsor or alternative disposition of unused trial drug; and
- dates, quantities, batch numbers, expiry dates and (for trial drug prepared at the site) formulation, as well as the subjects' trial numbers.

The Investigator should maintain records that adequately document

- that the subjects were provided the doses specified by the clinical trial protocol / amendment(s);and
- that all trial drugprovided by the Sponsor was fully reconciled.

Unused trial drug must not be discarded or used for any purpose other than the present trial. Any trial drug that has been dispensed to a subject must not be redispensed to a different subject.
The Sponsor's Monitor will periodically collect the trial drug accountability forms and will check all returns (both unused and used containers) before arranging for their return to the Sponsor or authorizing their destruction by the trial site.
At the conclusion or termination of this trial, trial site personnel and the Clinical Trial Monitor will conduct a final product supply inventory on the Investigational Drug Accountability Forms and all unused containers will be destroyed. Instructions for destruction of product will be provided to the site. The Clinical Trial Monitor will be supplied with a copy for filing of the Investigational Drug Accountability Forms.

This documentation must contain a record of clinical supplies used, unused,and destroyed and shall include information on:

- all administered units,
- all unused units,
- all destroyed units (during the trial),
- all destroyed units at the end of the trial,
- date of destruction(s),
- name and signature of the Investigator/pharmacist.

It must be ensured at each trial site that the trial drugis not used

- after the expiry date, and
- after the retest date unless the trial drugis reanalyzed and its retest date extended.

This is to be closely monitored by the Clinical Trial Monitor.

6.7 Assessment of Investigational Medicinal Product Compliance

In this trial, subjects will receive trial treatment at the investigational site. Well-trained medical staff will monitor and perform the trial drug administration. The information of each trial drug administration including the date, time, and dose of trial drug will be recorded on the eCRF. The Investigator will make sure that the information entered into the eCRF regarding drug administration is accurate for each subject. Any reason for noncompliance should be documented. Noncompliance is defined as a subject missing > 1 infusion of trial treatment for nonmedical reasons. If 1 infusion is missed and the interval between the subsequent infusion and the last administered treatment is longer than 4 weeks for nonmedical reasons, the criteria of insufficient compliance are met as well.

6.8 Treatment of Overdose

An overdose is defined as any dose ≥ 5% than the calculated dose for that particular administration as described in this clinical trial protocol. Any overdose must be recorded in the trial drug section of the eCRF. For monitoring purposes, any case of overdose, whether or not associated with an AE (serious or nonserious), must be reported to the Sponsor’s Global Drug Safety department in an expedited manner using the appropriate reporting form (see below). There are no known symptoms of avelumab or cetuximab overdose to date. The Investigator should use his or her clinical judgment when treating an overdose of the trial drug.

6.9 Medical Care of Subjects After End of Treatment

After a subject has stopped trial treatment, usual treatment will be administered, if required, in accordance with the trial site’s standard of care and generally accepted medical practice and depending on the subject’s individual medical needs. Upon withdrawal from trial treatment, subjects may receive whatever care they and their physicians agree upon. Subjects will be followed for survival and AEs.

7 Trial Procedures and Assessments

7.1 Screening and Baseline Procedures and Assessments

During the Screening period and before any trial-related investigations and assessments are started, subjects will be asked to sign the ICF. The Screening procedures and Baseline assessments will be completed within 28 days of signing the ICF before start of treatment. Failure to establish eligibility within 28 days would result in screening failure and the subject will be excluded from the trial; however, subjects can be re-entered in the trial based on the Investigator’s judgment within 6 weeks of signing the ICF. In this case, a new ICF will be required to be signed by the subject.

The subjects' information that will be documented during Screening includes the demographic information (birth date, sex, and race) and the complete medical history, including the history of mCRC previous and ongoing (concomitant) medications, and Baseline medical condition (the information of concomitant medications and AEs will be monitored throughout the trial treatment period). During Screening, subjects will undergo a physical examination, including recording body height and weight, vital signs, 12-lead ECG, and a determination of the ECOG PS.

The Screening laboratory examination includes hematology, hemostaseology, full serum chemistry (including core chemistry), and full urinalysis (dipstick plus microscopic evaluation). Adrenocorticotropic hormone (ACTH), ANA, ANCA, rheumatoid factor (RF), free thyroxine (T4), and thyroid-stimulating hormone (TSH) will also be assessed at Screening for all subjects. Additionally, HBV surface antigen and anti-HCV tests must be performed at screening to exclude hepatitis infection. If the anti-HCV antibody test is positive, infection should be confirmed by an HCV RNA test.

During Screening, a serum β-human chorionic gonadotropin (β-HCG) pregnancy test will be performed for females of childbearing potential and blood hepatitis B virus and hepatitis C virus will be performed (local laboratory) for all Screening subjects as these conditions are trial entry exclusion criteria.

Females who are postmenopausal (age-related amenorrhea ≥ 12 consecutive months and increased follicle-stimulating hormone [FSH] >40mIU/mL), or who have undergone hysterectomy or bilateral oophorectomy are exempt from pregnancy testing. If necessary to confirm postmenopausal status, FSH will be drawn at Screening.

The tumor evaluation (type and staging, etc) will be performed using CT scan or MRI (if MRI is used, CT of chest is mandatory) or any other established methods. A brain CT/MRI scan is required at Screening if not performed within 6weeks prior to start treatment. A bone scan should be done at Screening as clinically indicated. The blood samples for soluble factors, and immunogenicity will be collected before or on Day 1 before trial treatment.

7.2 Treatment Period

In this trial, the treatment will be given until PD, significant clinical deterioration (clinical progression), unacceptable toxicity, or any criterion for withdrawal from the trial or trial drug is fulfilled.

Treatment may continue past the initial determination of disease progression per RECIST 1.1 as long the following criteria are met:

- Investigator-assessed clinical benefit, without anyrapid disease progression
- Tolerance of trial drug
- Stable ECOG PS
- Treatment beyond progression will not delay an imminent intervention to prevent serious complications of disease progression (for example, central nervous system metastases).

The decision to continue treatment should be discussed with the Sponsor and documented in the trial records.

A radiographic assessment should be performed within 6 weeks of original PD to determine whether there has been a decrease in the tumor size, or continued PD. The assessment of clinical

benefit should be balanced by clinical judgment as to whether the subject is clinically deteriorating and unlikely to receive any benefit from continued treatment.

If the Investigator feels that the subject continues to achieve clinical benefit by continuing treatment, the subject should remain on the trial and continue to receive monitoring according to the Schedule of Assessments.

For subjects who continue trial therapy beyond progression, further progression is defined as an additional 10% increase in tumor burden volume from time of initial PD. This includes an increase in the sum of all target lesions and/or the development of new measurable lesions. Treatment should be discontinued permanently upon documentation of further disease progression.

New lesions are considered measureable at the time of initial progression if the longest diameter is at least 10 mm (except for pathological lymph nodes, which must have a short axis of at least 15mm). Any new lesion considered nonmeasureable at the time of initial progression may become measureable and therefore included in the tumor burden volume if the longest diameter increases to at least 10 mm (except for pathological lymph nodes, which must have a short axis of at least 15 mm).

Additionally, subjects who have experienced a CR should be treated for a maximum of 24months after confirmation, at the discretion of the Investigator. I f the Investigator believes that a subject may benefit from treatment beyond 24months, it may be permissible after discussion with the Sponsor. In case a subject with a confirmed CR relapses after stopping treatment, but prior to the end of the trial, 1re-initiation of treatment is allowed at the discretion of the Investigator. In order to be eligible for re-treatment, the subject must not have experienced any toxicity that led to treatment discontinuation of the initial therapy. Subjects who re-initiate treatment will stay on trial and will be treated and monitored according to the protocol and the “until progression” schedule in the Schedule of Assessments.

While on trial treatment, subjects will be asked to visit the trial site at each cycle. A time window of up to 1 days before or 1 day after the scheduled visit day (-1/+1 days) will be permitted for all trial procedures. In addition, the tumor evaluation has a tumor assessment visiting time window of 5 days prior to the scheduled day(-5 days).

Subjects will receive:

- avelumab by IV infusion following pretreatment with H1 blockers (diphenhydramine 25to 50mg IV, or equivalent), and acetaminophen 500 to 650 mg (oral or IV), once every 2weeks,

  AND

- cetuximab by IV infusion at a dose of 400 mg/m^2 on day 1, then 250 mg/m^2 weekly.

During the treatment period, the following assessments will be performed:

- AEs and concomitant medications will be documented at each trial visit.
- ECOG PS will be assessed at Day 1 (unless the Screening ECOG PS was performed within 3days prior to Day 1) and at each trial visit thereafter.
- Physical examinations will be performed at each visit.
- Vital signs and body weight will be assessed in each visit.

- The laboratory hematology, hemostaseology, full serum chemistry tests and basic urinalysis will be assessed every two weeks before avelumab and cetuximab administration. If the basic urinalysis is abnormal, a full urinalysis should be performed.

- A urine or serum β-HCG pregnancy test will be performed before each administration of the trial drug for females of childbearing potential. Results of the most recent pregnancy test should be available prior to the next dosing of trial drugs.

- Tumor evaluation for all subjects will be performed every 8 weeks for 40 weeks after starting treatment, and every 12 weeks thereafter regardless of any dose delays or treatment, with a tumor assessment visiting time window of 5 days prior to the scheduled tumor assessment day (-5 days).

- ACTH, ANA, ANCA, RF will be measured at Week 13, Week 25, and if clinically indicated for all subjects.

- FT4 and TSH will be measured at baseline and at least every 8 weeks during treatment

7.3 End of Treatment

Discontinuation visit

Any subject who experiences an AE that mandates discontinuation of trial treatment should have a Discontinuation visit as soon as possible after the decision to discontinue trial treatment (at least within 7 days).

For all these subjects, the Discontinuation visit will include the following:

- Subject-reported outcomes will be completed
- Documentation of AEs and concomitant medication
- Physical examination, including vital signs and body weight
- 12-lead ECGs
- Laboratory hematology, hemostaseology, full serum chemistry, and basic urinalysis
- ECOG PS

Once the Discontinuation visit has been performed, subjects must return for the End-of-Treatment visit within 28 days (± 5 days) after discontinuation.

End-of-Treatment visit

The End-of-Treatment visit is scheduled 4 weeks (28 days ± 5 days) after the last administration of trial treatment, but before any new therapy is started, if possible, whichever occurs earlier. The End-of-Treatment visit will comprise a full assessment for safety, immunogenicity, and tumor response as appropriate, andwill include the following:

- Subject-reported outcomes
- AEs, concomitant medications, and ECOG PS
- Physical examinationincluding vital signs andbody weight
- 12-lead ECGs
- Laboratory hematology, hemostaseology, full serum chemistry, and full urinalysis (dipstick plus microscopic evaluation)
- Urine or serum β-HCG pregnancy test (in females of childbearing potential)
- Tumor evaluation (only to be performed if no disease progression was documented previously)
- ACTH, ANA, ANCA, RF, T4, and TSH levels

- Blood sample for determination of soluble factors
- Blood samples for gene expression profiling

7.4 Safety Follow-up

Given the potential risk for delayed immune-related toxicities, safety follow-up must be performed up to 90 days after the last dose of avelumab administration.All subjects will have a subsequent visit scheduled 30 days after the last administration of trial treatment. The visit will include the following full assessment of safety parameters:

- After the End-of-Treatment visit only treatment related AEs have to be documented until the Safety Follow-up visit
- Concomitant medications will be documented, including further anticancer therapy
- Vital signs and body weight will be measured
- Physical examination will be performed
- ECOG PS will be assessed

Laboratory testing consisting of the following will be assessed:

- Hematology, hemostaseology, coreserum chemistry, and basic urinalysis
- ACTH, ANA, ANCA, RF, T4, and TSH levels
- A urine or serum β-HCG pregnancy test (in females of childbearing potential) will be conducted

The extended safety follow-up beyond 30 days after last avelumab administration may be performed either via a site visit or via a telephone call with subsequent site visit requested in case any concerns noted during the telephone call.

7.5 Long-term Follow-up

All SAEs ongoing at the Safety follow-up visit must be monitored and followed up by the Investigator until stabilization or until the outcome is known, unless the subject is documented as “lost to follow-up.” Any SAE assessed as related to the IMP must be reported whenever it occurs, irrespective of the time elapsed since the last administration of the IMP.
Subjects without PD according to RECIST 1.1 at the End-of-Treatment visit will be followed up for disease progression (CT/MRI scans every 8 weeks[± 5 days] using the same procedures and review as while on treatment) until disease progression, lost to follow-up, or withdrawal of informed consent.
After the End-of-Treatment visit, subjects will be followed quarterly (that is, every 3 months ±1week) for survival (including assessment of any further tumor therapy). The survival follow-up will continue until 2 years after the last subject receives the last dose of study drugs.

7.6 Demographic and Other Baseline Characteristics

The assessments and procedures described in this section must be performed during the Screening period.

7.6.1 Demographic Data

The following demographic data will be recorded:

- Subject identifier

- Date of birth
- Sex
- Race
- Ethnicit

7.6.2 Diagnosis of mCRC

The tumor disease information that will be documented and verified at the Screening visit for each subject includes:

- detailed history of the tumor, including histological/citological diagnosis, grading, and staging in accordance with the International Union Against Cancer Tumor Node Metastasis Classification of Malignant Tumors at diagnosis;
- all therapy used for prior treatment of the tumor (including surgery, radiotherapy and chemotherapy, immunotherapy);
- any other conditions that were treated with chemotherapy, radiation therapy, or immunotherapy;
- current cancer signs and symptoms and side effects from current and previous anticancer treatments; and
- currentcancerdisease status.

7.6.3 Medical History

In order to determine the subject's eligibility to the trial, a complete medical history of each subject will be collected and documented during Screening, which will include, but may not be limited to, the following:

- Past and concomitant nonmalignant diseases and treatments
- All medications (including herbal medications) taken and procedures carried out within 28days prior to Screening

For the trial entry, all of the subjects must fulfill all inclusion criteria described in Section 5.5, and none of the subjects should have any exclusion criteria from the list described in 5.5.

7.6.4 Vital Signs and Physical Examination

Vital signs including body temperature, respiratory rate, heart rate (after 5-minute rest), and arterial blood pressure (after 5-minute rest) will be recorded at trial entry. A physical examination (including, in general, appearance, dermatological, head/neck, pulmonary, cardiovascular, gastrointestinal, genitourinary, lymphatic, musculoskeletal system, extremities, eyes [inspection and vision control], nose, throat, and neurologic status) will be performed and the results documented. The ECOG PSwillbe documented during the Screening phase and at each scheduled visit. Body weight and height (Screening only) will be recorded.

7.6.5 CT or MRI Scans for Tumor Assessment at Baseline

Baseline imaging will be performed within 28 days prior the start of treatment in order to establish Baseline disease status of target and nontarget lesions according to RECIST 1.1. Acceptable modalities include CT scans (chest, abdomen, and pelvis), CT chest with contrast (or chest MRI in Germany) together with MRI of the abdomen and pelvis or positron emission tomography / CT scans. The use of IV contrast is preferred unless there is a history of allergy or other risk in the opinion of the Investigator (chest X-ray is not acceptable and other imaging modalities may be

performed at the discretion of the Investigator and as clinically indicated). Bone scans should be performed if clinically indicated. Baseline tumor burden should be determined as outlined in Section 7.7. A brain CT/MRI scan is required at Screening if one has not been performed within 8 weeks prior to starting treatment. In general, lesions detected at Screening/Baseline need to be followed using the same imaging methodology and preferably the same imaging equipment at subsequent tumor evaluation visits.

7.6.6 Cardiac Assessments

A 12-lead ECG will be recorded at Screening and at the Early Discontinuation/End-of Treatment visit after the subject has been in a supine position breathing quietly for 5 minutes. The ECG results will be used to evaluate the heart rate, atrial-ventricular conduction, QR and QT intervals, and possible arrhythmias. Left-ventricular function evaluation (echocardiogram or multigated acquisition scan) will be also performed

7.6.7 Ophthalmologic Assessment

An ophthalmologic assessment will be also performed prior to study treatment start and clinically indicated, including at least visual acuity and slit-lamp tests

7.6.8 Clinical Laboratory Tests

Blood samples will be collected at Screening for clinical laboratory parameter evaluations. These clinical laboratory test results will serve not only as the Baseline values for subsequent safety clinical laboratory evaluations during the trial, but will also help to make sure that each enrolled subject fulfills all the trial entry criteria and does not meet any of the trial exclusion criteria for laboratory parameters.

7.7 Assessment of Efficacy

Radiographic images and physical findings (physical assessments) will be used for the local determination of tumor response or progression according to RECIST 1.1.
For each subject, tumor response assessment will be performed by CT scan or MRI (if MRI is used, CT of chest is mandatory) imaging of the chest/abdomen/pelvis (plus other regions as specifically required) and other established assessments of tumor burden if CT/MRI imaging is insufficient for the individual subject.
All the scans performed at Baseline and other imaging performed as clinically required (other supportive imaging) need to be repeated at subsequent visits (except for brain scans, unless clinically indicated). In general, lesions detected at Baseline need to be followed using the same imaging methodology and preferably the same imaging equipment at subsequent tumor evaluation visits. A brain CT/MRI scan is required at Screening if not performed within 8 weeks prior the start of treatmnet. Brain CT/MRI scans should be performed after Screening, if clinically indicated by development of new specific symptoms. A bone scan should be done as clinically indicated at Screening and beyond. For each subject, the Investigator will designate 1 or more of the following measures of tumor status to follow for determining response: CT or MRI images of primary and/or metastatic tumor masses, physical examination findings, and the results of other assessments. All available images collected during the trial period will be considered. The most appropriate measures to evaluate the tumor status of a subject should be used. The measure(s) to be chosen for sequential evaluation during the trial must correspond to the measures used to

document the progressive tumor status that qualifies the subject for enrollment. The tumor response assessment will be assessed and listed according to the Schedule of Assessments.
Treatment decisions will be made by the Investigator based on the Investigator's assessment of tumor status. For efficacy determination, tumor responses to treatment will be assigned based on the evaluation of the response of target, nontarget, and new lesions according to RECIST 1.1 (all measurements should be recorded in metric notation, as described in RECIST 1.1).

To assess objective response, the tumor burden at Baseline will be estimated and used for comparison with subsequent measurements. At Baseline, tumor lesions will be categorized in target and nontarget lesions as described in RECIST 1.1. Results for these evaluations will be recorded with as much specificity as possible so that pre-and post-treatment results will provide the best opportunity for evaluating tumor response. Any CR or PR should be confirmed, preferably at the scheduled 8-week interval, but no sooner than 5 weeks after the initial documentation of CR or PR. Confirmation of PR can be confirmed at an assessment later than the next assessment after the initial documentation of PR. The Investigator may perform scans in addition to a scheduled trial scan for medical reasons or if the Investigator suspects PD.
Subjects who withdraw from the trial for clinical or symptomatic deterioration before objective documentation of PD will be requested to undergo appropriate imaging to confirm PD. Every effort should be made to confirm a clinical diagnosis of PD by imaging.

7.8 Assessment of Safety

The safety profile of the trial treatments will be assessed through the recording, reporting, and analyzing of Baseline medical conditions, AEs, physical examination findings, including vital signs, and laboratory tests.
Comprehensive assessment of any apparent toxicity experienced by the subject will be performed throughout the course of the trial, from the time of the subject's signature of informed consent.
Trial site personnel will report any AE, whether observed by the Investigator or reported by the subject. Given the intended mechanism of action of avelumab, particular attention will be given to AEs that may follow the enhanced T-cell activation, such as dermatitis, colitis, hepatitis, uveitis, or other immune-related reactions. Ophthalmologic examinations should be considered, when clinically indicated, for signs or symptoms of uveitis.. The safety assessments will be performed according to the Schedule of Assessments (refer to Appendix I)

7.9 Adverse Events

7.9.1 Adverse Event Definitions

An AE is any untoward medical occurrence in a subject or clinical investigation subject administered a pharmaceutical product, regardless of a causal relationship with this treatment. An AE can therefore be any unfavorable and unintended sign (including an abnormal laboratory finding), symptom, or disease temporally associated with the use of a medicinal product, whether or not considered related to the medicinal product.
In cases of surgical or diagnostic procedures, the condition / illness leading to such a procedure is considered as the AE rather than the procedure itself.
The Investigator is required to grade the severity/intensity of each AE.
Investigators will reference the NCI-CTCAE v 4.0. This is a descriptive terminology that can be used for AE reporting. A general grading (severity / intensity; hereafter referred to as severity) scale is provided at the beginning of the referenced document, and specific event grades are also

provided. If a particular AE severity is not specifically graded by the guidance document, the Investigator is to use the general NCI-CTCAE definitions of Grade 1 through Grade 5 following his or her best medical judgment.

The 5 general grades are:

Grade 1: Mild
Grade 2: Moderate
Grade 3: Severe
Grade 4: Life-threatening
Grade 5: Death

According to the Sponsor’s convention, any clinical AE with severity of Grade 4 or 5 must also be reported as an SAE; however, a laboratory abnormality of Grade 4, such as anemia or neutropenia, is considered serious only if the condition meets one of the serious criteria described below.
If death occurs, the primary cause of death (or event leading to death) should be recorded and reported as an SAE. “Fatal” will be recorded as the outcome of this respective event; death will not be recorded as a separate event. Only if no cause of death can be reported (for example,sudden death, unexplained death), the death per se might be reported as an SAE.
Investigators must also systematically assess the causal relationship of AEs to the trial treatment using the following definitions. Decisive factors for the assessment of causal relationship of an AE to trial treatment include, but may not be limited to, temporal relationship between the AE and treatment administration, known side effects of trial treatment, medical history, concomitant medication, course of the underlying disease, trial procedures.

Not related: Not reasonably related to the trial treatment. The AE could not medically (pharmacologically/clinically) be attributed to the trial treatment in this clinical trial protocol. A reasonable alternative explanation must be available.

Related: Reasonably related to the trial treatment. The AE could medically (pharmacologically/clinically) be attributed to the trial treatment.

Abnormal laboratory findings and other abnormal investigational findings (for example, on an ECG trace) should not be reported as AEs unless they are associated with clinical signs and symptoms, lead to treatment discontinuation, or are considered otherwise medically important by the Investigator. If a laboratory abnormality fulfills these criteria, the identified medical condition (for example, anemia, increased ALT) must be reported as the AE rather than the abnormal value itself.

Adverse Drug Reaction (ADR)
Adverse drug reactions are defined in this trial as any AEs suspected to be related to trial treatment by the Investigator and / or Sponsor.

Serious Adverse Events (SAE)
A SAE is any untoward medical occurrence that at any dose

- results in death,

- is life-threatening (NOTE: The term "life-threatening" refers to an event in which the subject is at risk of death at the time of the event; not an event that hypothetically might have caused death if it was more severe),
- requires inpatient hospitalization or prolongation of existing hospitalization,
- results in persistent or significant disability/incapacity,
- is a congenital anomaly/birth defect, or
- is otherwise considered as medically important.

Note: Important medical events that may not result in death, be life-threatening, or require hospitalization may be considered as SAEs when, based upon appropriate medical judgment, they may jeopardize the subject or may require medical or surgical intervention to prevent one of the outcomes listed above. Examples of such events include allergic bronchospasm requiring intensive treatment in an emergency room or at home, blood dyscrasias or convulsions that do not result in inpatient hospitalization, or the development of drug dependency or drug abuse.

For the purposes of reporting, any suspected transmission of an infectious agent via a trial drug is also considered a SAE, as described in Section 7.9.

Events that do not meet the definition of an SAE
Elective hospitalizations to administer, or to simplify trial treatment or trial procedures (for example, an overnight stay to facilitate chemotherapy and related hydration therapy application) are not considered as SAEs; however, all events leading to unplanned hospitalizations or unplanned prolongation of an elective hospitalization (for example, undesirable effects of any administered treatment) must be documented and reported as SAEs.

Events not to be considered as AEs/SAEs
Medical conditions present at the initial trial visit that do not worsen in severity or frequency during the trial are defined as Baseline Medical Conditions, and are NOT to be considered AEs.

AEs/SAEs observed in association with disease progression
Disease progression recorded in the course of efficacy assessments only, but without any adverse signs and symptoms should not be reported as AEs. However, if adverse signs or symptoms occur in association with disease progression then these should be recorded, and reported as SAEs if meeting any seriousness criteria.

Predefined AEs of special interest for safety monitoring
Any AE that is suspicious to be a potential irAE will be considered AEs of special interest (AESI).

7.9.2 Methods of Recording and Assessing Adverse Events

At each trial visit the subject will be queried on changes in his or her condition. During the reporting period of the trial any unfavorable changes in the subject's condition will be recorded as AEs, whether reported by the subject or observed by the Investigator.

Complete, accurate, and consistent data on all AEs experienced for the duration of the reporting period (defined below) will be reported on an ongoing basis in the appropriate section of the eCRF.

All SAEs must be additionally documented and reported using the appropriate report formas described in Section 7.9.

It is important that each AE report include a description of the event, its duration (onset and resolution dates and times to be completed when it is important to assess the time of AE onset relative to the recorded treatment administration time), its severity, its causal relationship with the trial treatment, any other potential causal factors, any treatment given or other action taken (including dose modification or discontinuation of the trial drug), and its outcome. In addition, serious cases should be identified and the appropriate seriousness criteria documented.

7.9.3 Definition of the Adverse Event Reporting Period

The AE reporting period for safety surveillance begins when the subject is initially included in the trial (date of first signature of informed consent) and continues through the trial's End-of-Treatment visit, defined as 28 days (± 5 days) after last trial drug administration. After the End-of-Treatment visit only treatment related AEs have to be documented until the Safety Follow-up visit, defined as 12 weeks (± 2 weeks) after the last trial treatment administration.
Any SAE suspected to be related to the trial treatment must be reported whenever it occurs, irrespective of the time elapsed since the last administration.
Given the potential risk for delayed immune-related toxicities, safety follow-up must be performed up to 90 days after the last dose of avelumab administration.
The extended safety follow-up beyond 30 days after last avelumab administration may be performed either via a site visit or via a telephone call with subsequent site visit requested in case any concerns noted during the telephone call.

7.9.4 Procedure for Reporting Serious Adverse Events

The Sponsor-Investigator primary responsibilities for safety reporting are to identify and follow-up on Serious Adverse Events (SAEs) experienced by participants in the study and to forward the information to the local regulatory authorities and Merck, as required by local regulations (for regulatory reporting) and as required by the ISS agreement (for reporting to Merck).

The following reportable events must be submitted to the Sponsorwithin 24 hours (or immediately for death or life-threatening events)* using the applicable safety report form provided. The Sponsor will assume responsibility for submitting the reportable event(s) to Merck within 2 business days or 3 calendar days (whichever comes first), as well as ensuring that any local reporting requirements are completed in parallel.

- Serious Adverse Events
- Exposure during Pregnancy or Breastfeeding (even if not associated with an adverse event)
- Occupational exposure (even if not associated with an adverse event)
- Potential drug-induced liver injury (Hy's Law cases): These events are considered important medical events and should be reported as SAEs.

Contact information for submission of reportable events toFortunato Ciardiello*:

Fax: [## ### ### ###]*
OR
E-mail: [xxxxxxxxxxxxxxx]*
Specifying:

- PROTOCOL Number and/or Title
- Merck assigned Study Number
- SUBJECT Number
- SITE Number/PI Name
- SAE/ONSET DATE

*Sponsor/Chief Investigator (CI): the lead Institution/Investigator responsible for the ISS who has entered into a contractual agreement with Merck – please update red text as applicable depending on reporting arrangements for your study.

In the event of any new SAE occurring during the reporting period, the Investigator must immediately (that is, within a maximum of 24 hours after becoming aware of the event) inform the Sponsor or designee in writing. All written reports should be transmitted using the SAE Report Form, which must be completed by the Investigator following specific completion instructions. In exceptional circumstances, an SAE (or follow-up information) may be reported by telephone. In these cases, a written report must be sent immediately thereafter by fax or e-mail.
Reporting procedures and timelines are the same for any new information on a previously reported SAE (= follow-up).

Names, addresses, telephone, and fax numbers for SAE reporting will be included in the trial specific SAE report form.
Relevant pages from the eCRF may be provided in parallel (for example, medical history, concomitant drugs). Additional documents may be provided by the Investigator, if available (for example, laboratory results, hospital report, autopsy report).

In all cases, the information provided on the SAE report form must be consistent with the data about the event recorded in the eCRF. The Investigator must respond to any request for follow-up information (for example, additional information, outcome final evaluation, other records where needed) or to any question the Sponsor or designee may have on the AE within the same timelines as those noted above for initial reports. This is necessary to ensure a prompt assessment of the event by the Sponsor or designee and (as applicable) to allow the Sponsor to meet strict regulatory timelines associated with expedited safety reporting obligations.

Requests for follow-up will usually be made by the responsible Clinical Trial Monitor, although in exceptional circumstances, the Global Drug Safety department may contact the Investigator directly to obtain further information or to discuss the event

7.9.5 Safety Reporting to Health Authorities, Independent Ethics Committees/Institutional Review Boards and Investigators

The Sponsor will send appropriate safety notifications to Health Authorities in accordance with applicable laws and regulations. The Investigator must comply with any applicable site-specific requirements related to the reporting of SAEs (and in particular deaths) involving his or her subjects to the IEC / IRB that approved the trial.

In accordance with ICH GCP guidelines, the Sponsor or designee will inform the Investigator of "findings that could adversely affect the safety of subjects, impact the conduct of the trial, or alter the IEC/ IRB approval / favorable opinion to continue the trial." In particular and in line with respective regulations, the Sponsor will inform the Investigator of AEs that are both serious and

unexpected and are considered to be related to the administered product (SUSARs). The Investigator should place copies of Safety reports in the Investigator Site File. National regulations with regard to Safety report notifications to Investigators will be taken into account.

When specifically required by regulations and guidelines, the Sponsor or the designee will provide appropriate Safety reports directly to the concerned lead IEC / IRB and will maintain records of these notifications. When direct reporting by the Sponsor is not clearly defined by national or site-specific regulations, the Investigator will be responsible for promptly notifying the concerned IEC / IRB of any Safety reports provided by the Sponsor or designee and of filing copies of all related correspondence in the Investigator Site File.

For trials covered by the European Directive 2001/20/EC, the Sponsor's responsibilities regarding the reporting of SAEs / SUSARs / Safety Issues will be carried out in accordance with that directive and with the related detailed guidance.

7.9.6 Monitoring of Subjects with Adverse Events

Adverse events are recorded and assessed continuously throughout the trial (see Section 7.9) and are assessed for final outcome at the End-of-Treatment visit. After the End-of-Treatment visit, only treatment related AEs have to be documented until the Safety Follow-up visit, defined as 30 days after the last trial treatment administration.

All SAEs ongoing at the Safety Follow-up visit must be monitored and followed up by the Investigator until stabilization or until the outcome is known, unless the subject is documented as "lost to follow-up." Reasonable attempts to obtain this information must be made and documented. It is also the responsibility of the Investigator to ensure that any necessary additional therapeutic measures and follow-up procedures are performed.

7.10 Pregnancy and In Utero Drug Exposure

Only pregnancies considered by the Investigator as related to trial treatment (for example, resulting from a drug interaction with a contraceptive medication) are considered to be AEs; however, all pregnancies with an estimated conception date during the period must be recorded by convention in the AE page / section of the eCRF. The same rule applies to pregnancies in female subjects and to pregnancies in female partners of male subjects. The Investigator must notify the Sponsor or designee in an expedited manner of any pregnancy using the Pregnancy Report Form, which must be transmitted according to the same process as described for SAE reporting in Section 7.9.4.

Investigators must actively follow up, document, and report on the outcome of all these pregnancies, even if the subjectiswithdrawn from the trial. The Investigator must notify the Sponsor or designee of these outcomes using the Pregnancy Report Form. If an abnormal outcome occurs, the SAE Report Form will be used if the subject sustains an event, and the Parent-Child / Fetus Adverse Event Report Form, if the child / fetus sustains an event. Any abnormal outcome must be reported in an expedited manner as described in Section 7.9.4, while normal outcomes must be reported within 45 days from delivery. In the event of a pregnancy in a subject occurring during the course of the trial, the subject must be discontinued from the trial drug immediately.

The Sponsor or designee must be notified without delay and the subject must be followed as mentioned above.

7.11 Clinical Laboratory Assessments

It is essential that the Sponsor or designee be provided with a list of laboratory normal ranges before shipment of trial drug. Any change in laboratory normal ranges during the trial will additionally be forwarded to the CRO and the Sponsor.
Blood samples will be taken from nonfasted subjects. All routine laboratory analyses will be performed at a laboratory facility local to the trial site and relevant results must be available and checked before administration of trial treatment. The report of the results must be retained as a part of the subject's medical record or source documents. Blood samples for the full safety tests listed in Table 6 will be taken from nonfasted subjects during the Screening phase (28 days prior starting treatment), during the treatment phase as specified in Table 6 and in Appendix I, at the End-of-Treatmentvisit, and at the Safety Follow-up visit.
The ACTH, ANA, ANCA, RF, T4, TSH, and urinalysis will only be assessed at the time points defined in Table 6 and Appendix I. If confirmation of a subject's postmenopausal status is necessary, a FSH level will also be performed at Screening, see Section 7.1.

Table 6. Required Full Laboratory Safety Tests

**Full Chemistry**	**Core Chemistry**[a]	**Hematology**
Albumin	Alkaline phosphatase	Absolute lymphocyte count
Alkaline phosphatase	ALT	ANC
ALT	AST	Hematocrit
Amylase	BUN/total urea	Hemoglobin
AST	Calcium	Platelet count
GGT	Chloride	RBC count
BUN/total urea	Creatinine	WBC count and differential count
Calcium	Glucose	Reticulocytes
Chloride	Phosphorus/Phosphates	MCH
Cholesterol	Magnesium	Mean corpuscular volume
Creatine kinase	Potassium	MCHC
Creatinine	Sodium	
CRP	Total bilirubin	
Glucose		**Hemostaseology**
LDH		aPTT
Lipase		Prothrombin time/INR
Phosphorus/Phosphates		
Magnesium		**Basic Urinalysis** (dipstick, including macroscopic appearance, bilirubin, blood, color, glucose, ketones, leukocyte esterase, nitrite, pH, protein, specific gravity, urobilinogen)<br><br>Full urinalysis(dipstick plus microscopic evaluation)to be performed only at the Screening and End-of-Treatment visits and a basic urinalysis prior to each administration of the trial drug.
Potassium		
Sodium		
Total bilirubin		
Total protein		
Uric acid		**TSH and T4**<br>To be assessed at the Screeningvisit,

		and every 8 weeks
Triglycerides		
		**ACTH, ANA, ANCA, RF**<br>To be assessed at the Screeningvisit, Week 13, Week 25, and at the End-of Treatment visit
**Hormone**		
FSH (yes / no if applicable)		

ACTH=adrenocorticotropic hormone, ALT=alanine aminotransferase, ANA=antinuclear antibody, ANC=absolute neutrophil count, ANCA=antineutrophil cytoplasmic antibody, aPTT=activated partial thromboplastin time, AST=aspartate aminotransferase, BUN=blood urea nitrogen, CRP=C-reactive protein, FSH=follicle-stimulating hormone, GGT=gamma-glutamyltransferase, INR=international normalized ratio, LDH=lactate dehydrogenase, MCH=mean corpuscular hemoglobin, MCHC=mean corpuscular hemoglobin concentration, RBC=red blood cell, RF=rheumatoid factor; TSH=thyroid-stimulating hormone, T4=free thyroxine, WBC=white blood cell.

If a subject has a clinically significant abnormal laboratory test value that is not present at Baseline, the test will be repeated weekly and the subject will be followed until the test value has returned to the normal range or the Investigator has determined that the abnormality is chronic or stable.

7.12 Vital Signs, Physical Examination and Other Assessments

The ECOG PSwill be assessed at Screeningand at subsequent visits as indicated in the Schedule of Assessments (Appendix I) and documented in the eCRF. Body weight will be measured at Screening and at subsequent visits as indicated in the Schedule of Assessments (Appendix I) and documented in the eCRF. Body height will be measured at Screening only.

A physical examination will be conducted at Screening and at subsequent visits as indicated in the Schedule of Assessments (Appendix I) and documented in the eCRF (detailed description in Section 7.1). Results of the physical examination, including any abnormalities, will be documented in the eCRF. Abnormal findings are to be reassessed at subsequent visits. Also a 12-lead ECG will be recorded as indicated in the Schedule of Assessments (Appendix I). All newly diagnosed or worsening conditions, signs, and symptoms observed from Screening, whether related to trial treatment or not, are to be reported as AEs.

For female subjects of childbearing potential, a serum β-HCG pregnancy test will be carried out during the Screening phase. A urine or serum β-HCG test will be performed before each administration of trial drugduring the treatment phase and at the End-of-Treatmentvisit. Results of the most recent pregnancy test should be available prior to the next dosing of trial drug. Subjects who are postmenopausal (age-related amenorrhea ≥ 12 consecutive months and FSH>40mIU/mL), or who have undergone hysterectomy or bilateral oophorectomy are exempt from pregnancy testing.

8Description of Statistical Analyses

8.1 Primary Endpoint

The primary endpoint of the trial is the OS, defined as the time (in months) starting treatment to the date of death, regardless of the actual cause of the subject's death.

The survival follow-up will continue until 2 years after the last subject receives the last dose of avelumab and cetuximab. For subjects who are still alive at the time of data analysis or who are

lost to follow- up, OS time will be censored at the last recorded date that the subject is known to be alive (date of last contact, last visit date, date of last trial treatment administration, or date of last scan, whichever is the latest) as of the data cut-off date for the analysis. If the date of last known status of alive or death date is after the data cut-off date, subjects will be censored at the data cut-off date.

8.2 Secondary Endpoints

8.2.1 Progression-Free Survival

The PFS will be determined according to RECIST 1.1. It is defined as the time from date of starting treatment until date of the first documentation of PD or death by any cause (whichever occurs first).

8.2.2Overall response rate (ORR)

The ORR will be determined according to RECIST 1.1. The ORR is defined as the proportion of all randomized subjects with a confirmed Best Overall Response (BOR) of PR or CR according to RECIST 1.1. The BOR is defined as the best response obtained among all tumor assessment visits after the date of starting treatment until documented disease progression.

8.2.3 Safety Endpoints

Safety endpoints include AEs, clinical laboratory assessments, vital signs, physical examination, ECG parameters, and ECOG PS

8.3 Description of Statistical Analyses

8.3.1 General Considerations

Baseline characteristics summary and the efficacy analysis will be performed on the total population enrolled in the study.

In order to provide overall estimates of treatment effects, data will be pooled across trial centers.

In general, descriptive summaries will be presented for the efficacy and safety variables collected. Continuous variables will be summarized using mean, standard deviation, minimum, median, and maximum. Categorical variables will be summarized using frequency counts and percentages.

8.3.2 Analysis of Primary Endpoint

The primary endpoint of this trial is OS. The primary analysis population will be the ITT population. We consider the results from phase 3 studies with standard third line treatment as comparator (CORRECT, RECOURSE). The current study aims to demonstrate a median OS of 11.0 months (alternative hypothesis) by experimental combination for comparison with historical median OS 8.0 (null hypothesis) with standard third line treatment, which correspond to an improvement of OS at 6 months from 35% to 46%. It was estimated that we would need to enroll 66 patients to achieve with a 1-sided 5% level test in this single stage, single arm trial. The accrual period will be of 18 months and the total duration of the stud will be of 36 months. Considering a potential drop-out of approximately 15% of patients a total of 75 patients will be recruited.

8.3.3 Analysis of Secondary Endpoints

Secondary efficacy analyses will be performed on the ITT analysis set.
For the secondary endpoint analysis of PFS time according to RECIST 1.1 the statistical analysis will be the same as described for the primary analysis of OS time.

For the secondary endpoint analysis of ORR according to RECIST 1.1, the ORR in terms of having a confirmed BOR of CR or PR will be calculated along with corresponding two-sided exact Clopper-Pearson 95% CIs.

9 Ethical and Regulatory Aspects

9.1 Responsibilities of the Investigator

The Investigator is responsible for the conduct of the trial at his/her site. He/she will ensure that the trial is performed in accordance with the clinical trial protocol and with the ethical principles that have their origin in the Declaration of Helsinki, as well as with the ICH Note for Guidance on GCP (ICH Topic E6, 1996) and applicable regulatory requirements. In particular, the Investigator must ensure that only subjects who have given their informed consent are included in the trial.

In 1998, the USA FDA introduced a regulation (21 Code of Federal Regulations, Part 54) entitled "Financial Disclosure by Clinical Investigators." For trials conducted in any country that could result in a product submission to the FDA for marketing approval and could contribute significantly to the demonstration of efficacy and safety of the trial drug (named "covered trials" by the FDA), the Investigator and all sub-Investigators are obliged to disclose any financial interest that they, their spouses, or their dependent children may have in the Sponsor or the Sponsor's product under study. This information is required during the trial and for 12 months following completion of the trial.

9.2 Subject Information and Informed Consent

An unconditional prerequisite for a subject's participation in the trial is his/her written informed consent. The subject's written informed consent to participate in the trial must be given before any trial-related activities are carried out. A separate specific PGx ICF will be provided to subjects who are willing to participate in this optional procedure, which refers to the extraction and analysis of DNA from blood and/or tumor biopsy in order to better understand how gene(s) may affect the efficacy of avelumab.

Adequate information must therefore be given to the subject by the Investigator before informed consent is obtained (a person designated by the Investigator may give the information, if permitted by local regulations). A subject information sheet in the local language and prepared in accordance with the Note for Guidance on GCP (ICH Topic E6, 1996) will be provided by the Sponsor for the purpose of obtaining informed consent. In addition to providing this written information to a potential subject, the Investigator or his / her designate will inform the subject verbally of all pertinent aspects of the trial. The language used in doing so must be chosen so that the information can be fully and readily understood by lay persons.

Depending on national regulations, a person other than the Investigator may inform the subject and sign the ICF, as above.

Where the information is provided by the Investigator, the ICF must be signed and personally dated by the subject and the Investigator.

The signed and dated declaration of informed consent will remain at the Investigator's site, and must be safely archived by the Investigator so that the forms can be retrieved at any time for monitoring, auditing, and inspection purposes. A copy of the signed and dated information and ICF should be provided to the subject prior to participation.

Whenever important new information becomes available that may be relevant to the subject's consent, the written subject information sheet and any other written information provided to subjects will be revised by the Sponsor or designee and be submitted again to the IEC / IRB for review and favorable opinion. The agreed, revised information will be provided to each subject in the trial for signing and dating. The Investigator will explain the changes to the previous version.

9.3 Subject Identification and Privacy

A unique subject number will be assigned to each subject at inclusion immediately after informed consent has been obtained. This number will serve as the subject's identifier in the trial as well as in the clinical trial database.

The subject's data collected in the trial will be stored under this number. Only the Investigator will be able to link the subject's trial data to the subject via an identification list kept at the site. The subject's original medical data that are reviewed at the site during source data verification by the Clinical Trial Monitor, audits, and Health Authority inspections will be kept strictly confidential.

Data protection and privacy regulations will be observed in capturing, forwarding, processing, and storing subject data. Subjects will be informed accordingly and will be requested to give their consent on data handling procedures in accordance with national regulations.

9.4 Emergency Medical Support and Subject Card

Subjects enrolled in this clinical trial will be provided with Emergency Medical Support cards during their trial participation, which will be furnished by the Sponsor or designee. The Emergency Medical Support card is based on the need to provide clinical trial subjects with a way of identifying themselves as participating in a clinical trial, and subsequently to give health care providers access to the information about this participation that may be needed to determine the course of the subject's medical treatment.

This service is designed to provide information to health care providers who are not part of the clinical trial.

Clinical trial Investigators, who are already aware of the clinical trial protocol and treatment, have other means of accessing the necessary medical information for the management of emergencies occurring in their subjects.

The first point of contact for all emergencies will be the clinical trial Investigator caring for the affected subject. The Investigator agrees to provide his or her emergency contact information on the card for this purpose. If the Investigator is available when an event occurs, she or he will answer any questions. Any subsequent action will follow the standard processes established for the Investigators.

9.5 Clinical Trial Insurance and Compensation to Subjects

Insurance coverage shall be provided for each center participating in the trial. Insurance conditions shall meet good local standards, as applicable.

9.6 Independent Ethics Committee or Institutional Review Board

Prior to commencement of the trial at a given site, the clinical trial protocol will be submitted together with its associated documents (such as the ICF) to the responsible IEC/IRB for its favorable opinion / approval. The written favorable opinion/approval of the IEC/IRB will be filed in the Investigator Site File, and a copy will be filed with the CRO.

The trial must not start at a site before the Sponsor or designee has obtained written confirmation of favorable opinion / approval from the concerned IEC/IRB. The IEC/IRB will be asked to provide documentation of the date of the meeting at which the favorable opinion/approval was given, and of the members and voting members present at the meeting. Written evidence of favorable opinion / approval that clearly identifies the trial, the clinical trial protocol version, and the Subject Information and ICF version reviewed should be provided. Where possible, copies of the meeting minutes should be obtained.

Amendments to the clinical trial will also be submitted to the concerned IEC/IRB before implementation in case of substantial changes (see Section 10.5). Relevant safety information will be submitted to the IEC/IRB during the course of the trial in accordance with national regulations and requirements.

9.7 Health Authorities

The clinical trial protocol and any applicable documentation (for example, Investigational Medicinal Product Dossier, Subject Information, and ICF) will be submitted or notified to the Health Authorities in accordance with the regulations of the countries involved in the trial.

10 Trial Management

10.1 Case Report Form Handling

The Investigator or designee will be responsible for entering trial data in the eCRF provided by the CRO and follow the data entry guidelines. It is the Investigator’s responsibility to ensure the accuracy of the data entered in the eCRFs and to sign the case report forms.

The data will be entered into a validated database. The CRO will follow the standards of the Sponsor in the database design and data structure. The CRO will be responsible for data review and processing, in accordance with the CRO’s data management procedures. Database lock will occur once quality control procedures and quality assurance procedures (if applicable) have been completed. Copies of the eCRFs will be provided to the Investigators at the completion of the trial.

10.2 Source Data and Subject Files

The Investigator must keep a subject file (medical file, original medical records) on paper or electronically for every subject included in the trial. This file will contain the available demographic and medical information for the subject, and should be as complete as possible.

In particular, the following data should be available in this file:

- Subject's full name
- Date of birth
- Sex
- Race
- Height
- Weight
- Medical history and concomitant diseases
- Prior and concomitant therapies (including changes during the trial)
- Tumor disease information
- Trial identification
- Date of subject's inclusion into the trial (that is, date of giving informed consent)
- Subject number in the trial
- Dates of the subject's visits to the site
- Any medical examinations and clinical findings predefined in the clinical trial protocol
- All AEs observed in the subject
- Date of subject's end of trial
- Date of and reason for early withdrawal of the subject from the trial or from trial drug, if applicable

It must be possible to identify each subject by using this subject file.

Additionally, any other documents containing source data must be filed. This includes original printouts of data recorded or generated by automated instruments, photographic negatives, X-rays, CT or MRI scan images, ECG recordings, laboratory value listings, etc. Such documents must include at least the subject number and the date when the procedure was performed. Information should be printed by the instrument used to perform the assessment or measurement, if possible. Information that cannot be printed by an automated instrument will be entered manually. Medical evaluation of such records should be documented as necessary and the documentation signed and dated by the Investigator.

10.3 Investigator Site File and Archiving

The Investigator will be provided with an Investigator Site File upon initiation of the trial. This file will contain all documents necessary for the conduct of the trial and will be updated and completed throughout the trial. It must be available for review by the Monitor, and must be ready for Sponsor audit as well as for inspection by Health Authorities during and after the trial, and must be safely archived for at least 15 years (or per local requirements or as otherwise notified by the Sponsor) after the end of the trial. The documents to be archived include the Subject Identification List and the signed subject ICFs. If archiving of the Investigator Site File is no longer possible at the site, the Investigator must notify the Sponsor.

All original subject files (medical records) must be stored at the site (hospital, research institute, or practice) for the longest possible time permitted by the applicable regulations, and / or as per ICH GCP guidelines, whichever is longer. In any case, the Investigator should ensure that no destruction of medical records is performed without the written approval of the Sponsor.

10.4 Monitoring, Quality Assurance, and Inspection by Health Authorities

This trial will be monitored in accordance with the ICH Note for Guidance on GCP (ICH Topic E6, 1996). The Clinical Trial Monitor will perform visits to the trial site at regular intervals.

Representatives of the Sponsor's Quality Assurance unit or a designated organization, as well as Health Authorities, must be permitted to inspect all trial-related documents and other materials at the site, including the Investigator Site File, the completed eCRFs, the trial drug, and the subjects' original medical records/files.

The clinical trial protocol, each step of the data capture procedure, and the handling of the data, including the final clinical trial report, will be subject to independent quality assurance activities. Audits may be conducted at any time during or after the trial to ensure the validity and integrity of the trial data.

10.5 Changes to the Clinical Trial Protocol

Changes to the clinical trial protocol will be documented in written protocol amendments. Major (substantial, significant) amendments will usually require submission to the Health Authorities and to the relevant IEC/IRB for approval or favorable opinion. In such cases, the amendment will be implemented only after approval or favorable opinion has been obtained.

Minor (nonsubstantial) protocol amendments, including administrative changes, will be filed by the Sponsor and at the site. They will be submitted to the relevant IEC/IRB or to Health Authorities only where requested by pertinent regulations.

Any amendment that could have an impact on the subject's agreement to participate in the trial requires the subject's informed consent prior to implementation (see Section 9.2).

10.6 Clinical Trial Report and Publication Policy

10.6.1 Clinical Trial Report

After completion of the trial, a clinical trial report according to ICH Topic E3 will be written by the Sponsor in consultation with the Coordinating Investigator.

10.6.2 Publication

The first publication will be a publication of the results of the analysis of the primary endpoint(s) that will include data from all trial sites.

The Investigator will inform the Sponsor in advance about any plans to publish or present data from the trial. Any publications and presentations of the results (abstracts in journals or newspapers, oral presentations, etc), either in whole or in part, by Investigators or their representatives will require presubmission review by the Sponsor. The Sponsor will not suppress or veto publications, but maintains the right to delay publication in order to protect intellectual property rights.

12. References

References

.

1. Torre LA, Bray F, Siegel RL, Ferlay J, Lortet-Tieulent J, Jemal A. Global cancer statistics, 2012. *CA Cancer J Clin*. 2015;65:87-108
2. Schmoll HJ, Van Cutsem E, Stein A, Valentini V, Glimelius B, Haustermans K, Nordlinger B, van de Velde CJ, Balmana J, Regula J. ESMO Consensus Guidelines for management of patients with colon and rectal cancer. a personalized approach to clinical decision making. AnnOncol. 2012;23:2479-2516.
3. Van Cutsem E, Cervantes A, Nordlinger B, Arnold D. Metastatic colorectal cancer: ESMO Clinical Practice Guidelines for diagnosis, treatment and follow-up. *Ann Oncol*. 2014;25Suppl 3:iii1-iii9.
4. Mayer RJ, Van Cutsem E, Falcone A, Yoshino T, Garcia-Carbonero R, Mizunuma N, Yamazaki K, Shimada Y, Tabernero J, Komatsu Y. Randomized trial of TAS-102 for refractory metastatic colorectal cancer. *N Engl J Med*. 2015;372:1909-1919.
5. Wadlow RC et al. Panitumumab in patients with KRAS wild-type colorectal cancer after progression on cetuximab. Oncologist 2012; 17: 14
6. Saif MW et al. Efficacy of panitumumab therapy after progression with cetuximab: experience at two institutions. Clin Colorectal Cancer 2010; 9: 315-318
7. Santini D et al. Cetuximab rechallenge in metastatic colorectal cancer patients: how to come away from acquired resistance Ann Oncol. 2012 Sep;23(9):2313-8.
8. Hauschild A et al. Checkpoint inhibitors: a new standard of care for advanced Merkelcell carcinoma Lancet Oncol. 2016, 17(10): 1337–1339
9. P.Correale et al. Cetuximab ±chemotherapy enhances dendritic cell-mediated phagocytosis of colon cancer cells and ignites a highly efficient colon cancer antigen specific cytotoxic T-cell responce*in vitr. Int.J.cancer 2012. 130: 1577-1589*
10. Marecheal . R et al. Puttativecontribution of CD56 positive cells in cetuximab treatment efficacy in firts line metastatic colorectal cancer patients. BMC Cancer 2010.10,340.
11. Dechant.M et al. Complement-dependent tumor cell lysis triggered by combinations of epidermal growth factor receptros antibodies. Cancer Res.2008.68, 4998-5003.
12. . Baselga J. The EGFR as a target for anticancer therapy – focus on cetuximab. Eur J Cancer 2001; 37(Suppl 4):S16-22.
13. Fan Z, Baselga J, Masui H, Mendelsohn J. Antitumor effect of anti-epidermal growth factor receptor monoclonal antibodies plus cis-diamminedichloroplatinum on well established A431 cell xenografts. Cancer Res 1993; 53: 4637-4642.
14. Milas L, Mason K, Hunter N, et al. In vivo enhancement of tumor radioresponse by C225 antiepidermal growth factor receptor antibody. Clin Cancer Res 2000;6:701-708.
15. Yarden Y. The EGFR family and its ligands in human cancer. signalling mechanisms and therapeutic opportunities. Eur J Cancer 2001; 37 Suppl 4:S3-8

16. Folprecht G, Lutz MP, Schoffski P, et al. Cetuximab and irinotecan/5-fluorouracil/folinic acid is a safe combination for the first-line treatment of patients with epidermal growth factor receptor expressing metastatic colorectal carcinoma. Ann Oncol 2006;17:450-456
17. Wilke H, Glynne-Jones R, ThalerJ,et al. MABEL – A large multinational study of cetuximab plus irinotecan in irinotecan resistant metastatic colorectal cancer. J ClinOncol 2006;24(18S):Abstract 3549
18. . Salomon DS, Brandt R, Ciardiello F, Normanno N. Epidermal growth factor-related peptides and their receptors in human malignancies. Crit Rev OncolHematol 1995; 19:183-232
19. Erbitux SmPC (Feb 2007)
20. Bonner JA, Harari PM, Giralt J, et al. Radiotherapy plus Cetuximab for Squamous-Cell Carcinoma of the Head and Neck. New Engl J Med. 2006; 354:567-78
21. Siena S, Glynne-Jones R, Thaler J, et al. MABEL - A large multinational study of cetuximab plus irinotecan in irinotecan-resistant metastatic colorectal cancer: Update on infusion related reactions (IRR). ASCO Gastrointestinal Cancers Symposium 2007:Abstract 353
22. Howard SC, Jones DP, Pui CH. The tumor lysis syndrome. N Engl J Med 2011; 364(19):1844-54.

11. Appendices

Appendix I Schedules of Assessments

Table 7. Screening/Baseline, Treatment Phase and Follow-up procedures

Measure	Screening/ Baseline Assements	Treatment Phase[a]								Discontinuation(x)/ End-of-Treatment Visit (X)	Safety Follow-up Visit	Long-term Follow-up[b]
	Day -28 to starting treatment	V1 W1 D1	V2 W2 D8	V3 W3 D15	V4 W4 D21	V5 W5 D28	V6 W6 D35	V7 W7 D43	Until Progression	Up to 7/28 Days (±5 days) after Last Treatment[c,d]	12 Weeks (±2weeks) after Last Treatment	Every 3 moths (±1 week) [b]
Written Informed Consent	X											
Collection of tumor tissue (when available)	X											
Inclusion/exclusion criteria	X											
Medical history [e]	X											
Demographic data	X											
HBV and HCV testing	X											
Physical examination, including Height at Screening	X	X	X	X	X	X	X	X	1 week	x/X	X	
Vital Signs	X	X	X	X	X	X	X	X	1 week	x/X	X	
Weight	X	X	X	X	X	X	X	X	1week	x/X	X	
ECOG PS	X[f]	X	X	X	X	X	X	X		x/X	X	
Enrollment (if eligible)[g]	X											
Cardiac assessment[h]	X									x/X		
Ophtalmologicassessment[i]	X											
Hematology and hemostaseology	X	X		X		X		X	2 weeks	x/X	X	
Full serum chemistry[j]	X	X		X		X		X	2 weeks	x/X	X	
	Screening/ Baseline Assements	Treatment Phase								Discontinuation(x)/ End-of-Treatment Visit (X)	Safety Follow-up Visit	Long-term Follow-up[b]
	Day -28 to starting treatment	V1 W1 D1	V2 W2 D8	V3 W3 D15	V4 W4 D21	V5 W5 D28	V6 W6 D35	V7 W7 D43	Until Progression	Up to 7/28 Days (±5 days) after Last Treatment[c,d]	12 Weeks (±2weeks) after Last	Every 3 moths (±1 week) [b]

Measure											Treatment	
Urinalysis[k]	X	X		X		X		X	**2 weeks**	**x/X**	X	
β-HCG pregnancy test[l]	X					X			**28 days**	**-/X**	X	
Tumor evaluation by CT scan or MRI (a bone scan should be done at Screening as clinically indicated)[m,n]	X							X	**8 weeks for 40weeks and every 12 weeks thereafter**	**-/X**		X
Documentation of AEs and concomitant medications[o]	X	X	X	X	X	X	X	X	**1 week**	**x/X**	X	X
ACTH, ANA, ANCA, RF	X								**Week 13, week 25, as indicated**	**-/X**	X	
T4, and TSH	X								**8 weeks**	**-/X**	X	
Pretreatment and trial drug administration[p]		X	X	X	X	X	X	X	**1 week**			

ACTH=adrenocorticotropic hormone, ADR=adverse drug reaction; AE=adverse events,ALT=alanine aminotransferase, ANA=antinuclear antibody, ANCA=antineutrophil cytoplasmic antibody, AST=aspartate aminotransferase,β-HCG=β-human chorionic gonadotropin, BUN=blood urea nitrogen, CR= complete response, CT=computedtomography, D=Day, ECG=electrocardiogram, ECOG PS=Eastern Cooperative Oncology GroupPerformance Status, HAHA=human antihuman antibody, HBV=hepatitis B virus, HCV=hepatitis C virus, ICF=Informed Consent Form, IV=intravenous, MRI=magnetic resonance imaging, PR=partial response, RECIST=ResponseEvaluation Criteria in Solid Tumors version 1.1, RF=rheumatoid factor, T4=free thyroxine, TSH=thyroidstimulating hormone, V=visit, W=Week.

aA time window of up to 1 day before or 1 day after the scheduled visit day (-1 / +1 days) will be permitted for all trial procedures. The calculation of the dose of avelumab will be based on the weight of the subject determined on the day prior to or the day of each drug administration. Complete blood count and core chemistry samples must also be drawn and results reviewed within 48 hours prior to dose administration.

b Subjects with an SAE ongoing at the Safety follow-up must be monitored and followed up by the Investigator until stabilization or until the outcome is known, unless the subject is documented as “lost to follow-up.” Any SAE assessed as related to IMP must be reported whenever it occurs, irrespective of the time elapsed since the last administration of IMP. Subjects without progressive disease at End-of-Treatment visit will be followed up for disease progression (CT / MRI scans every 6 weeks [±5days]) for up to 1 year. In addition, survival information (including assessment of any further anticancer therapy) will be collected quarterly (that is, every 3 months ±1week). The survival follow-up will continue until 2 years after the last subject receives the last dose of avelumab (see Section 7.5. for details).

c Tumor evaluation at the End-of-Treatmentvisit should only be performed if no disease progression has beendocumented previously

d If another antineoplastic therapy is administered before the end of this 28-day period, the End-of-Treatmentvisit should be conducted,if possible,prior to the start of this new therapy.

e Medical history should includehistory of mCRC, previous and ongoing medications, , and Baselinemedical condition.

f If the ScreeningECOGPSwas performed within 3 days prior to Day 1,it does not have to be repeated at Visit 1.

g Enrollment will be done after the confirmation of fulfilling all screening inclusion criteria without matching any exclusion criterion (Section5.6.1).

hCardiac assessment includes: 12-lead ECG that should be assessed during screening and at the Discontinuation / End-of-Treatment visit andLeft-ventricular function evaluation (echocardiogram or multigated acquisition scan).

I Ophtalmologic assessment : visual acuity and slit-lamp test

jFull chemistry includes core serum chemistry and other laboratory studies are detailed in Table 6. Follicle-stimulating hormone at Screening, if applicable (Section 7.1).

k Full urinalysis (dipstick plus microscopic evaluation) at the Screening and End-of-Treatment visits and a basic urinalysis (dipstick only) at each visit indicated prior to administration oftrial drug. If the basic urinalysis is abnormal, then a full urinalysis should be performed.

l β-HCG should be determined from serum at Screening and from a urine or serum sample thereafter. Results of the most recent pregnancy test should be available prior to next dosing of trial drug.

m In general, the tumor visit time window is 5 days prior to the scheduled tumor assessment. In case a tumor response according to RECIST 1.1 is documented during the course of the trial, confirmation of the response should be performed according to RECIST 1.1, preferably at the regularly scheduled 6-week assessment interval, but no sooner than 5weeks after the initial documentation of CR or PR. Confirmation of PR can be confirmed at an assessment later than the next assessment after the initial documentation of PR. A CT scan or MRI should always be used (if MRI is used, CT of chest is mandatoryin all countries except Germany, in which case a MRI of the chest is allowed).

n A brain CT / MRI scan is required at Screening if not performed within 6 weeks prior to starting treatment, and beyond as clinically indicated. A bone scan should be done as clinically indicated at Screeningand beyond. Bone metastases detected at Screeningneed to be followed at the tumor evaluation visits.

o Adverse events and concomitant medications will be documented at each trial visit. The AE reporting period for safety surveillance begins when the subject is initially included in the trial (date of first signature of informed consent) and continues through the trial's End of Treatment visit, defined as 28 days (± 5 days) after last trial drug administration. After the End of Treatment visit only treatment related AEs have to be documented until the Safety Follow up visit, defined as 12 weeks (± 2 weeks) after the last trial treatment administration.

pPremedication: in order to mitigate infusion related reactions, a premedication with an antihistamine and with paracetamol (acetaminophen) 30 to 60 minutes prior to the infusions of avelumabplus cetuximab is mandatory (for example, 25 50 mg diphenhydramine and 500 650 mg paracetamol IV or oral). This may be modified based on local treatment standards and guidelines, as appropriate.

Table 8. Soluble factors, Gene Expression profiling and Immunogenicity sampling times

Measure	Screening/ Baseline Assements	Treatment Phase[a]				
	Day -28 to starting treatment	V1 W1 D1	V3 W3 D15	V5 W5 D28	V7 W7 D43	Until Progression
		Prior to infusion	Prior to infusion	Prior to infusion	Prior to infusion	
Blood and plasma sample[a]	X					X
Tumor Tissue[b]	X					

a Blood samples will be collected from all subjects prior to infusion on Day1 (Week 1; Baseline samples for soluble factors may also be collected at Screening, instead of on Day 1 prior to dosing) and at progression.

B Tumor Tissue when availble

Appendix II Eastern Cooperative Oncology Group Performance Status

**ECOG PS[a]**	
Grade	ECOG
0	Fully active, able to carry on all pre-disease performance without restriction
1	Restricted in physically strenuous activity but ambulatory and able to carry out work of a light or sedentary nature, for example, light house work, office work
2	Ambulatory and capable of all self-care,but unable to carry out any work activities; up andabout> 50% of waking hours
3	Capable of only limited self-care, confined to bed or chair > 50% of waking hours
4	Completely disabled; cannot carry on any self-care; totally confined to bed or chair
5	Dead

aOken MM, Creech RH, Tormey DC, Horton J, Davis TE, McFadden ET, Carbone PP. Toxicity and Response Criteria of the Eastern Cooperative Oncology Group. Am J ClinOncol 1982;5: 649-55 (40).

G.O.N.O.

GRUPPO ONCOLOGICO NORD-OVEST

A PHASE II SINGLE-ARM STUDY OF CETUXIMAB PLUS IRINOTECAN AS RECHALLENGE 3RD-LINE TREATMENT OF *KRAS*, *NRAS* AND *BRAF* WILD-TYPE IRINOTECAN-PRETREATED METASTATIC COLORECTAL CANCER PATIENTS PROGRESSING AFTER AN INITIAL RESPONSE TO A 1ST-LINE CETUXIMAB-CONTAINING THERAPY AND A STANDARD 2ND-LINE

THE CRICKET STUDY

EUDRACT 2014-001126-15

-- Protocol Vers. 2.0 – August 2014 --

STUDY STAFF

Principal Investigators:

Prof. Alfredo Falcone	U.O. Oncologia Medica 2 Universitaria<br>Università di Pisa – AOUP<br>Polo Oncologico Area Vasta Nord-Ovest<br>Istituto Toscano Tumori	mail: alfredo.falcone@med.unipi.it
Prof. Daniele Santini	U.O.C. Oncologia Medica<br>Policlinico Universitario<br>Campus Biomedico - Roma	mail: d.santini@unicampus.it

Protocol Contributors:

Dr. Fotios Loupakis	U.O. Oncologia Medica 2 Universitaria<br>Università di Pisa – AOUP<br>Polo Oncologico Area Vasta Nord-Ovest<br>Istituto Toscano Tumori	mail: fotiosloupakis@gmail.com
Dr. Lisa Salvatore	U.O. Oncologia Medica 2 Universitaria<br>Università di Pisa – AOUP<br>Polo Oncologico Area Vasta Nord-Ovest<br>Istituto Toscano Tumori	mail: salvatore.lisa82@gmail.com
Prof. Daniele Santini	U.O.C. Oncologia Medica<br>Policlinico Universitario<br>Campus Biomedico - Roma	mail: d.santini@unicampus.it

Clinical Trial Administration:

Dr. Fotios Loupakis	U.O. Oncologia Medica 2 Universitaria<br>Università di Pisa – AOUP<br>Polo Oncologico Area Vasta Nord-Ovest<br>Istituto Toscano Tumori	mail: fotiosloupakis@gmail.com
Dr.ssa Laura Delliponti	U.O. Oncologia Medica 2 Universitaria<br>Università di Pisa – AOUP<br>Polo Oncologico Area Vasta Nord-Ovest<br>Istituto Toscano Tumori	mail: laura.delliponti@gmail.com

Translational laboratory

Prof. Romano Danesi	U.O. Farmacologia Clinica, Dip. Medicina Clinica e sperimentale, Università di Pisa	mail: romano.danesi@unipi.it
Dr.ssa Marzia Del Re	U.O. Farmacologia Clinica, Dip. Medicina Clinica e sperimentale, Università di Pisa	mail: marzia.delre@for.unipi.it

PARTICIPATING CENTERS

1. U.O. Oncologia Medica 2 Universitaria, Azienda Ospedaliero-Universitaria Pisana, Pisa
2. U.O.C. Oncologia Medica, Policlinico Universitario, Campus Biomedico, Roma
3. Clinica Oncologia Medica, Azienda Ospedaliero-Universitaria, Ancona
4. U.O. Oncologia Medica, Policlinico S. Orsola-Malpighi, Bologna
5. Polo Oncologico Provinciale Frosinone, Azienda Sanitaria Locale Frosinone
6. Oncologia Falck, Ospedale Niguarda Ca' Granda, Milano
7. U.O. Oncologia Medica, Seconda Università degli Studi, Napoli
8. U.O. Oncologia Medica 1, Istituto Oncologico Veneto, Padova
9. U.O. Oncologia Medica, ASL 5, Ospedale "F. Lotti", Pontedera
10. U.O. Oncologia Medica, Azienda Policlinico Umberto I, Roma
11. U.O.C. Oncologia, Ospedale S. Giovanni Calibita Fatebenefratelli, Roma
12. U.O. Oncologia, Azienda Ospedaliero-Universitaria, S. Maria della Misericordia, Udine
13. U.O. Oncologia, Arcispedale Santa Maria Nuova, Reggio Emilia
14. U.O.C. Oncologia Medica, Policlinico Universitario, "A.Gemelli" , Roma

TABLE of CONTENTS

1. ACRONYM .... page 05

2. INTRODUCTION .... page 06

3. STUDY RATIONALE .... page 09

4. STUDY DESIGN .... page 11

5. STUDY OBJECTIVES .... page 12

6. STATISTICAL METHODS .... page 14

7. PATIENTS' SELECTION .... page 15

8. PARTICIPATING CENTERS, ENROLLMENT AND STUDY DURATION…… .... page 17

9. STUDY TREATMENT .... page 18

10. BASELINE AND ON TREATMENT EVALUATIONS .... page 19

11. BLOOD, PLASMA AND TUMOR SAMPLES COLLECTION AND ANALYSES .... page 21

12. SAFETY ISSUES .... page 23

13. ETHICAL ISSUES .... page 33

14. STUDY MONITORING .... page 36

15. ADVERS EVENTS…… .... page 37

16. REFERENCES .... page 41

APPENDICES .... page 43

APPENDIX 1 – SYNOPSIS (ENGLISH VERSION) .... page 44

APPENDIX 2 – SINOSSI (ITALIAN VERSION).......... page 50

APPENDIX 3 –DICHIARAZIONE DI HELSINKI .... page 55

APPENDIX 4 – SCALA PER LA VALUTAZIONE DEL PERFORMANCE STATUS.......... page 59

APPENDIX 5 – VALUTAZIONE RISCHIO/BENEFICIO .... page 60

1. ACRONYM

Cetuximab **R**echallenge in **I**rinotecan-pretreated m**C**RC, ***K****RAS, NRAS* and *BRAF* wild-type treated in 1st line with anti-**E**GFR **T**herapy **(CRICKET)**.

2. INTRODUCTION

Cetuximab is a chimeric IgG1 monoclonal antibody (moAb) that binds extracellular domain of epidermal growth factor receptor (EGFR) preventing its linkage with endogenous ligands such as transforming growth factor-*alfa* and epidermal growth factor.

EGFR is a transmembrane glycoprotein with an intracellular tyrosine kinase domain. EGFR activation leads to the activation of intracellular effectors involved in intracellular signaling pathways, such as the G protein KRAS. Moreover, oncogene *KRAS* mutations affect the clinical response to anti-EGFR therapy. Several phase II and phase III trials supported cetuximab combination in first-line treatment of metastatic colorectal cancer (mCRC). Van Cutsem et al. have conducted a randomized phase III trial to investigate the efficacy of cetuximab added to FOLFIRI as first-line treatment of metastatic colorectal cancer (mCRC) patients. A total of 1598 patients, with an immunohistochemical EGFR-positive tumor, were randomly assigned to receive FOLFIRI alone or in combination with cetuximab (at the initial dose of 400 mg/sqm, followed by a dose of 250 mg/sqm once weekly). The primary end-point of the study was progression-free survival (PFS): the addition of cetuximab significantly improved PFS (8.9 months vs 8.0 months; HR=0.85, 95% CI 0.72-0.99; p=0.048). Also in terms of response rate (RR), cetuximab plus FOLFIRI achieved a significant advantage in comparison to FOLFIRI alone (RR: 46.9% vs 38.7%; p=0.004) with a consequent improvement in the rate of radical surgery of metastases with curative intent (4.8% vs 1.7%; p=0.002). No significant difference in overall survival (OS) was found between the two treatment groups: 19.9 months vs 18.6 months in cetuximab-FOLFIRI and FOLFIRI group respectively (HR=0.93, 95% CI 0.81-1.07; p=0.31). The toxicity profile of the combination treatment, cetuximab plus FOLFIRI, was in line with that expected: the incidence of grade 3 skin reactions, and in particular acne-like rash, was significantly higher in patients receiving the anti-EGFR moAb in comparison with those receiving FOLFIRI alone (skin reactions: 19.7% vs 0.2%, p<0.001; acne-like rash: 16.2% vs 0.0%; p<0.001). None of the skin-related toxicities reported were grade 4 and, in the cetuximab-FOLFIRI group, grade of rash was shown to be associated with PFS. Also the incidence of grade 3-4 diarrhea (15.7% vs 10.5%, p=0.008) and infusion-related reactions (2.5% vs 0.0%, p<0.001) was significantly increased in cetuximab-FOLFIRI group. However, these toxicities were manageable and the combination treatment appeared feasible and well tolerated. [1, 2]

A randomized phase II study evaluated the activity of cetuximab combined with FOLFOX-4 versus FOLFOX-4 alone in the first-line treatment of EGFR-expressing mCRC. Three hundred and thirty-seven patients were enrolled: 169 patients received cetuximab (weekly schedule) plus FOLFOX-4 and 168 patients received FOLFOX-4 alone. The addition of cetuximab to chemotherapy showed an increase, even if not significant, in RR (46% vs 36%, p=0.064) and it was associated with an approximate doubling of R0 resection rate (4.7% vs 2.4%). In terms of PFS, no benefit was achieved by the combination of cetuximab with FOLFOX-4 in the intention-to-treat (ITT) population.

The combination treatment was well tolerated and the most frequent grade 3-4 adverse events reported were consistent with the well-known toxicity profile of cetuximab. Skin reactions (including xerosis, erythema, dermatitis acneiform, pruritus, skin exfoliation) were observed in 18% of patients treated with cetuximab plus FOLFOX-4 versus 0.6% reported in the FOLFOX-4-alone group. Infusion-related reactions occurred in 5% and 2% of patients receiving FOLFOX-4 with or without cetuximab respectively. Hypersensivity reactions and rash were the most common reasons for cetuximab discontinuation. The incidence of grade 3-4 neutropenia and grade 3 diarrhea was similar in the two groups of treatment (30% and 8%, respectively, in cetuximab plus FOLFOX-4; 34% and 7%, respectively, in FOLFOX-4 alone); no grade 4 diarrhea was reported. [3, 4]

The German phase III clinical trial FIRE-3 reports that first-line cetuximab plus FOLFIRI chemotherapy offers a roughly four-month survival advantage for patients with metastatic colorectal cancer, compared with bevacizumab plus FOLFIRI (28.8 vs 25.0 mo, HR 0.77, p=0.0164, 95% CI: 0.620-0.953), RR was comparable between arms in the ITT analysis (62% vs 57%, odds ratio 1.249). Median PFS of the ITT population was nearly identical (10.3 vs 10.4 mo, HR 1.04, p=0.69).[5]

Other studies support the use of cetuximab as a single agent or in combination with irinotecan for patients who have progressed on a previous chemotherapy. [6, 7]

Many efforts have been made to identify potential predictors of benefit from anti-EGFR moAbs.

Since the evaluation of EGFR expression by immunohistochemistry was not demonstrated as an useful tool to predict the efficacy of the treatment, attention has been focused on intracellular mediators, involved in the transduction of EGFR signal. Both KRAS/BRAF/MAPKs and PTEN/PI3K/pAKT pathways have been investigated.

Several retrospective experiences, subsequently corroborated by the results of *post-hoc* analyses of large phase III randomized studies, have demonstrated the role of *KRAS* activating mutations as predictors of resistance to anti-EGFR antibodies. Such mutations, that occur in about the 40% of CRCs, involve codons 12 and 13 in more than 90% of cases and lead to the constitutive activation of the RAS/RAF/MAPKs cascade. Signalling events are thus independent from EGFR control. The *post-hoc* analysis of the CRYSTAL trial according to *KRAS* mutational status showed that only patients with *KRAS* wild-type disease derived a significant advantage both in terms of PFS (9.9 months vs 8.7 months, HR=0.68, p=0.017) and RR (59.3% vs 43.2%, OR= 1.91), by the administration of cetuximab combined with chemotherapy. [2] Similarly, in the OPUS study, a phase II randomized trial assessing the efficacy of FOLFOX plus cetuximab (vs FOLFOX) as a first line regimen, the *post-hoc* analysis showed that among patients with *KRAS* wild-type disease, those treated with cetuximab experienced a better outcome both in terms of RR and PFS, in comparison with patients who had received only FOLFOX (RR: 60.7% vs 37.0%, p=0.011; PFS: 7.7 months vs 7.2 months, HR=0.57, p=0.016). [4, 8]

Such results were confirmed by the analysis of phase III trials that randomized heavily pretreated mCRC patients to anti-EGFR monotherapy vs best supportive care (BSC), whose results are thus

not affected by the potential confounding effect of the associated chemotherapy regimens. When compared to BSC, both cetuximab and panitumumab have demonstrated a survival benefit only for patients with *KRAS* wild-type tumors. No responders were identified among patients with *KRAS* mutated disease, treated with panitumumab, in comparison with the 17% of patients with *KRAS* wild-type tumors. Similar findings were reported in terms of PFS: the treatment effect in the *KRAS* wild-type group (HR, 0.45; 95% CI: 0.34 to 0.59) was significantly greater (P< .0001) than in the mutant group (HR, 0.99; 95% CI, 0.73 to 1.36). [9, 10]

As a result of the above reported results of *post-hoc* analyses and retrospectively collected series, demonstrating the negative predictive value of *KRAS* codon 12 and 13 mutations, the use of monoclonal antibodies is now restricted to patients with *KRAS* wild-type disease.

KRAS activating mutations, occurring in codons other than 12 and 13, have been described in mCRC. Codon 61 and 146 mutations, that have been detected with frequencies ranging from 1 to 4%, determine the constitutive activation of RAS protein. It has been reported that, among 87 patients with *KRAS* codon 12 and 13 wild-type disease, none of the patients bearing codon 61 or 146 mutations responded to cetuximab plus irinotecan, compared to 22 out of 68 wild type patients (p=0.096). *KRAS* mutations were also associated with shorter PFS (HR: 0.46, P=0.028). [11]

Analysis of a phase III panitumumab monotherapy study indicated that *KRAS* mutations beyond exon 2 and *NRAS* mutations may be predictive of panitumumab efficacy. [12] It has also been hypothesized that other mutations downstream of EGFR could affect its anti-EGFRs effectiveness such as *BRAF*. BRAF is a serine-threonine kinase and the principal effector of KRAS. *BRAF* V600E mutation in CRC occurs in 5%–12% of cases, and several retrospective studies have suggested that it is associated with a decreased response to anti-EGFR therapy. [13]

Douillard et al. recently published a retrospective analysis of the PRIME trial: the objective was to assess the effect of panitumumab + FOLFOX vs FOLFOX on OS in patients with mCRC based on *RAS* (*KRAS* or *NRAS*) or *BRAF* mutational status. A statistically significant OS benefit was observed in patients with wild-type *RAS* mCRC treated with panitumumab + FOLFOX vs FOLFOX (median gain of more than 7 months in the panitinumab arm), while patients with any RAS mutation derived a significantly detrimental effect from EGFR inhibition. On the basis of these results the use of panitumumab was restricted to *RAS (K-* and *N-RAS)* wt mCRC pts.. In this analysis, *BRAF* mutation showed an undisputed prognostic value, while its predictive value remains controversial even if the efficacy of EGFR inhibition in *BRAF* mutant patients is clearly of limited value. [14]

Results on extended *RAS* mutations as negative predictors were confirmed also by the retrospective analysis of the FIRE3 study, showing a lack of response to cetuximab in patients with any *RAS* mutation.[15]

3. STUDY RATIONALE

As just mentioned above, recent data emerging from literature clearly pointed out that activating mutations of *RAS* predict lack of response to cetuximab or panitumumab therapy. At the same time *BRAF* V600E mutant patients have an extremely poor prognosis[16 17] and limited chances to benefit from anti-EGFR moAbs.[18 11, 13]

The acquisition of secondary mutations in tumoral tissue, has not been shown yet to play a major role in resistance to anti-EGFRs in mCRC: the evaluation of *KRAS*/*BRAF* status before and after anti-EGFR antibody treatment carried out by Gattenlohner et al. resulted highly concordant (95% for *KRAS*, 100% for *BRAF*). However, 5% to 10% of mCRC show *KRAS* molecular heterogeneity between primary, lymph nodes, and distant metastases. [19]

Baldus et al. evaluated *KRAS*, *BRAF*, and *PI3K* gene status in the primary tumor, comparing the tumor center and the invasion fronts. The intratumoral heterogeneity of *KRAS*, *BRAF*, and PIK3CA mutations was observed in 8%, 1%, and 5% of primary tumors, respectively. [20] According to the evidence of intratumoral heterogeneity, the occurrence of disease progression after the initial response in a wild-type *KRAS* primary tumor could not be due to late acquisition of the mutation, but rather to the progressive prevalence of a mutated clone, caused by a sort of 'cetuximab-driven mutated genotype acquisition' occurring during therapy. The recourse to a cetuximab-based therapy in *KRAS* wild-type mCRC patients, even without modifing *KRAS* gene status, could lead to the destruction of wild-type cells and to the prevalence of mutated clones, which lead, after an initial tumor reduction, to the first progression of disease. A further line of therapy without cetuximab could restore *KRAS* wild-type clones, which may constitute the major part of the tumor mass at the time of a following progression of disease. At this point, a rescue through a cetuximab-based new line therapy may result in a further shrinkage of the disease. Moreover, the tumor cell entrance to epithelial-to-mesenchymal transition (EMT) or the reverse mesenchymal-to-epithelial transition may justify response or refractoriness, respectively, in patients retreated with cetuximab. EMT is characterized by the combined loss of epithelial cell junction proteins such as E-cadherin and the gain of mesenchymal markers such as vimentin. Therefore, it is likely that the epithelial cells are more susceptible to EGFR-targeted therapies due to their activation of AKT primarily through EGFR-ErbB3. Mesenchymal cells activate AKT through alternative pathways like integrin-linked kinase (ILK) and are largely resistant to EGFR inhibitors. Cetuximab-based therapy could lead during the time, after a first response, to activation of this alternative pathway, ILK-dependent, which favours EMT. [21] A further line without anti-EGFR therapy may down-regulate this process restoring cetuximab sensitivity. Santini et al. conducted a multicenter retrospective analysis that examined irinotecan-refractory patients who had a clinical benefit following a line of cetuximab-plus irinotecan-based therapy and then progression of disease, during cetuximab-based therapy, who underwent a new line chemotherapy and finally, after a clear new progression of disease, were retreated with the same or another cetuximab- plus irinotecan-based therapy. Thirty-nine

patients were enrolled, median age was 59 years: RR was 53.8 [95% confidence interval (CI) 39.1% to 63.7%] with 19 PRs (48.7%) and 2 CRs (5.1%). Stable disease (SD) was obtained in 35.9% of patients (95% CI 24.7% to 51.6%) for a clinical control rate of disease of 89.8%. Progression occurred in only four patients (10.2%). The median PFS was 6.6 months (95% CI 4.1% to 9.1%). Eighteen patients (46.1%) showed the same type of response (SD, PR or CR) during cetuximab retreatment when compared with the response obtained during the first cetuximab-based therapy, 2 patients (5.1%) had an increase in the quality of clinical result, transiting from PR to CR and from SD to PR, respectively. Both, SD lasting at least 6 months and PR during the first cetuximab-based therapy have been demonstrated to predict clinical benefit after cetuximab retreatment. [22]

Basing on these biological and clinical data, we designed a phase II prospective study with the aim of demonstrating that patients who responded and then progressed during a first-line irinotecan- and cetuximab-based therapy may benefit again, after a second-line of therapy, from a third-line treatment with cetuximab and irinotecan.

4. STUDY DESIGN

This is a multicentric, phase II single-arm study in which *KRAS*, *NRAS* and *BRAF* wild-type, irinotecan-resistant mCRC patients progressing after an initial response to a first-line cetuximab-containing therapy, receive a rechallenge third-line treatment with cetuximab plus irinotecan.

5. STUDY OBJECTIVES

PRIMARY OBJECTIVE

The main objective of this study is to evaluate, in terms of Overall Response Rate (ORR) the activity of cetuximab plus irinotecan as rechallenge third line treatment of *RAS (K*- and *N-RAS,* codons 12, 13, 59, 61, 117, 146*)* and *BRAF (V600E)* wild-type, irinotecan-resistant, mCRC patients progressing after an initial response to a first-line irinotecan- and cetuximab-containing therapy and a second-line with FOLFOXIRI/FOLFOX/XELOX plus bevacizumab.

SECONDARY OBJECTIVES

Secondary objectives of this study are:

- the duration of progression-free survival (PFS);
- the duration of overall survival (OS);
- the safety profile;
- the evaluation of potential predictive and/or prognostic biomarkers.

PRIMARY ENDPOINT

The primary endpoint of this study is **overall response rate (ORR)**

ORR is defined as the percentage of patients, relative to the total of enrolled subjects, achieving a complete (CR) or partial (PR) response, according to RECIST 1.1 criteria. The determination of clinical response will be based on investigator-reported measurements. Responses will be evaluated with a chest and abdominal computed tomography (CT) scan every 8 weeks. Patients who do not have an on-study assessment will be included in the analysis as non-responders.

SECONDARY ENDPOINTS

Secondary endpoints of this study are the following:

Progression-free survival (PFS) is defined as the time from the start of therapy until the first documentation of objective disease progression or death due to any cause, whichever occurs first. PFS will be censored on the date of the last evaluable on-study tumor assessment documenting absence of progressive disease for patients who are alive, on study and progression-free at the time of the analysis, or if lost to follow-up. Alive patients having no tumor assessments after baseline will have time to event endpoint censored on the date of treatment start .
The determination of disease progression will be based on investigator-reported measurements. Disease status will be evaluated according to RECIST 1.1 criteria.

Overall survival (OS) is defined as the time from the start of therapy until the date of death due to any cause. For patients still alive at the time of analysis, or if lost to follow up, the OS time will be censored on the last date the patients were known to be alive.

Toxicity rate is defined as the percentage of patients, relative to the total of enrolled subjects, experiencing a specific adverse event, according to National Cancer Institute Common Toxicity V 4.03

6. STATISTICAL METHODS

STATISTICAL CONSIDERATION AND SAMPLE SIZE

According to the Fleming single-stage design and selecting the design parameters p0 (RR in the null hypothesis) = 0.05*, and p1 (RR in the alternative hypothesis) = 0.20**, and considering alpha (one-sided) and beta errors of 0.05 and 0.20 respectively, a total of 27 patients will be required. Null hypothesis will be rejected if at least 4 patients have an objective response.

*A null hypothesis of 0.05 is based on the consideration that available therapies in later lines of treatment for mCRC lead to an extremely low RR. Second-line irinotecan based chemotherapy produces less than 5% RR. [23, 24]

The results of this study will also be subjected to a combined analysis with data from study 062202-264.

**The alternative hypothesis of 0.20 is what we consider as a potential target of interest for further studies in this setting with the experimental treatment.

ANALYSIS POPULATIONS

MODIFIED INTENTION TO TREAT POPULATION (mITT)

The mITT population will include all patients who receive at least one dose of study medication. The mITT population will be the population for evaluating all primary and secondary endpoints,

ANALYSES of ENDPOINTS

ANALYSIS OF PRIMARY ENDPOINT

Best overall response rate will be calculated as the number of patients with a best response of CR or PR divided by the total number of enrolled patients. The corresponding 95% confidence interval will be calculated using a method based on the binomial distribution.

ANALYSIS OF SECONDARY ENDPOINTS

PFS and OS will be described using the Kaplan Meier method for estimating time-to-events and will also be displayed graphically. The median event times and corresponding 95% CI for the median will be provided.

Toxicity rates and overall toxicity rate will be calculated as the number of patients experiencing a specific adverse event of grade 3/4 or any adverse event of grade 3/4 divided by the total number of enrolled patients. The corresponding 95% confidence interval will be calculated using a method based on the binomial distribution.

7. PATIENTS' SELECTION

INCLUSION CRITERIA

- Histologically proven diagnosis of colorectal adenocarcinoma;
- *RAS* and *BRAF* wild-type status of primary colorectal cancer and/or related metastasis;
- First-line irinotecan-based (FOLFIRI or FOLFOXIRI) cetuximab-containing therapy producing at least a partial response;
- First-line progression-free survival in response to cetuximab-containing therapy ≥ 6 months;
- Documentation of progression to first-line cetuximab within 4 weeks after last cetuximab administration;
- Time between the end of first-line therapy and the start of third-line treatment with cetuximab plus irinotecan ≥4 months;
- Second-line oxaliplatin-based (FOLFOXIRI, FOLFOX or XELOX) bevacizumab-containing therapy;
- Documentation of progression to second-line treatment;
- Measurable disease according to Response Evaluation Criteria in Solid Tumors (RECIST criteria, vers.1.1);
- Have tumor tissue (of primary tumor and metastases or at least one of the two) available for biomarker analysis;
- Male or female, aged > 18 years of age;
- ECOG Performance Status ≤ 2;
- Life expectancy of at least 3 months;
- Adequate bone marrow, liver and renal function assessed within 14 days before starting study treatment;
- Women of childbearing potential must have a negative blood pregnancy test at the baseline visit. For this trial, women of childbearing potential are defined as all women after puberty, unless they are postmenopausal for at least 12 months, are surgically sterile, or are sexually inactive;
- Subjects and their partners must be willing to avoid pregnancy during the trial and until 6 months after the last trial treatment. Male subjects with female partners of childbearing potential and female subjects of childbearing potential must, therefore, be willing to use adequate contraception as approved by the investigator, such as a two-barrier method or one-barrier method with spermicidal or intrauterine device. This requirement begins 2 weeks before receiving the first trial treatment and ends 6 months after receiving the last treatment;
- Signed informed consent obtained before any study specific procedures.

EXCLUSION CRITERIA

- Active uncontrolled infections or active disseminated intravascular coagulation;
- Past or current history of malignancies other than colorectal carcinoma, except for curatively treated basal or squamous cell carcinoma of the skin or in situ carcinoma of the cervix;
- Fertile women (<12 months after last menstruation) and men of childbearing potential not willing to use effective means of contraception;
- Women who are pregnant or are breastfeeding;
- Previous Grade 3/4 infusion related reaction to cetuximab.

8. PARTICIPATING CENTERS, ENROLLMENT AND STUDY DURATION

PARTICIPATING CENTERS

14 Italian Centers

ENROLLMENT AND DATA COLLECTION

Patient registration and data collection are centralized at Polo Oncologico Area Vasta Nord-Ovest – Azienda Ospedaliero-Universitaria Pisana (AOUP), Istituto Toscano Tumori (ITT).

STUDY DURATION

Planned accrual time is 18 months for a total study duration of 24 months.

WITHDRAWAL OF SUBJECTS FROM TREATMENT and FROM STUDY

The Investigator has the right to discontinue a patient from study treatment or withdraw a patient from the study at any time. In addition, patients have the right to voluntarily discontinue study treatment or withdraw from the study at any time for any reason. In instances where consent is withdrawn, the Investigator must clarify whether the patient is willing to continue to be followed (i.e. for survival).

Reasons for discontinuation of study treatment may include, but are not limited to, the following:

- Any medical condition that the Investigator or Sponsor determines may jeopardise the patient‘s safety if he or she continues study treatment;
- Major protocol violation (i.e. affecting the patients‘ safety);
- Investigator or Sponsor determines it is in the best interest of the patient;
- Patient non-compliance;
- Patient withdrawal of consent to receive further study treatment.

Reasons for withdrawal from the study may include, but are not limited to, the following:

- Patient withdrawal of consent to be followed up;
- Patient lost to follow-up;
- Death.

9. STUDY TREATMENT

Experimental Treatment: cetuximab plus irinotecan

- **Cetuximab** 500 mg/sqm iv over 1-h every 2 weeks

followed by

- **Irinotecan** 180 mg/sqm iv over 1-h every 2 weeks (or according to investigator's choice in the best interest of the patient at previously maximum tolerated dose, but no <130 mg/mq)

Treatment will be continued until:

- Disease progression;
- Death;
- Unacceptable toxicity*;
- Consent withdrawn.

*if irinotecan is stopped due to toxicity, cetuximab may be continued as monotherapy at physician's discretion.

If cetuximab is stopped due to toxicity, irinotecan may be continued as monotherapy at physician's discretion.

10. BASELINE AND ON-TREATMENT EVALUATIONS

AT BASELINE

- Complete medical history, ECOG PS, physical examination and vital signs;
- Complete blood chemistry: total bilirubin, AST, ALT, alkaline phosphatase, total proteins, albumin, LDH, creatinine, electrolytes (Na^+, K^+, Ca^{++}, Mg^{++}), aPTT, INR, CEA, CA19.9; pregnancy test (if clinically indicated);
- Complete blood count and differential;
- Chest and Abdominal computed tomography (CT) scan, or Abdomen MRI and Chest X-Ray if CT scan contraindicated;
- Written informed consent;
- Collection of formalin-fixed paraffin-embedded tumor blocks (or 10 slides of conventional thickness and polarity for IHC and 10 slides of 10 micron thickness for molecular biology) of primary and/or metastatic sites;
- Before the administration of the 1st cycle: collection of whole blood (two 6 ml K2-EDTA Vacutainer. Total volume withdrawn: 12 ml) and plasma samples (three 6 ml K2-EDTA Vacutainer. Total volume withdrawn: 18 ml) for pharmacogenetic, pharmacodynamic and circulating DNA analyses. Whole blood and plasma samples will be immediately maintained at 0-4°C (not more than 45 min) and then stored at -20°C and -80°C, respectively.

DURING TREATMENT - EVERY 2 WEEKS

- Before the administration of the 2nd cycle (i.e. cycle 1 day 14): collection of plasma samples (three 6 ml K2-EDTA Vacutainer. Total volume withdrawn: 18 ml) for pharmacodynamic analyses. Plasma samples will be immediately maintained at 0-4°C (not more than 45 min) and then stored at -80°C;
- Partial Blood chemistry: total bilirubin, AST, ALT, alkaline phosphatase, creatinine;
- Complete blood count and differential;
- Toxicity evaluation (CTCAE v4.03)ECOG PS, physical examination (including weight).

DURING AND AFTER TREATMENT UNTIL DISEASE PROGRESSION - EVERY 8 WEEKS

- At first CT-scan evaluation and at progression: collection of plasma samples (three 6 ml K2-EDTA Vacutainer. Total volume withdrawn: 18 ml) for pharmacodynamic and circulating DNA analyses. The obtained plasma samples will be immediately maintained at 0-4°C (not more than 45 min) and then stored at -80°C;
- Tumor evaluation (RECIST criteria v1.1) by using the same technique performed at baseline;

- Complete blood chemistry: total bilirubin, AST, ALT, alkaline phosphatase, total proteins, albumin, LDH, creatinine, electrolytes (Na^+, K^+, Ca^{++}, Mg^{++}), aPTT, INR, CEA, CA19.9.

FLOW CHART			
	Baseline	***Every 2 weeks***	***Every 8 weeks***
**Informed Consent**	X		
**Demographics and Medical History**	X		
**General Physical Examination**	X		X
**Vital Signs and Physical Measurements** Height, body weight, temperature, blood pressure, pulse, PS	X	X	X
**Blood pregnancy test** (if applicable)	X		
**Hematology** Hemoglobin, platelet count, RBC, WBC including differential	X	X	X
**Blood Chemistry** Creatinine, alkaline phosphatase, ALT, AST, total bilirubin		X	
**Complete Blood Chemistry and Markers** Creatinine, alkaline phosphatase, ALT, AST, GGT, LDH, total bilirubin, total proteins, albumin, Na^+, K^+, Ca^{++}, Mg^{++}, aPTT, INR, CEA, Ca19.9	X		X
**Tumoral Samples Collection**	X		
**Whole Blood and Plasma Sampling**	X	X (2nd cycle)	X (1st CT-scan and PD)
**Toxicities Evaluation** According to NCI CTCAEv4.03	X	X	X
**Tumor Measurements** According to RECIST Criteria 1.1	X		X

11. BLOOD, PLASMA AND TUMOR SAMPLES COLLECTION AND ANALYSES

In order to investigate the potential predictive and/or prognostic biomarkers, blood, plasma and tumor samples will be collected and analyzed as following:

Blood and plasma samples will be obtained for all study participants. Blood and plasma samples will usually be obtained at a time when the subject is having blood drawn for other trial purposes.

Preparation and storage of whole blood samples

- Venous blood will be obtained by any standard phlebotomy technique from a peripheral access point or from a central line by trained personnel (two 6 ml K2-EDTA Vacutainer) and stored at -20 °C as soon as possible (no later than 45 min). DNA will be extracted by means of standard commercial kits with the aim to run explorative pharmacogenetic analyses of candidate SNPs in selected genes directly or indirectly related to the EGFR pathway.

Preparation and storage of plasma samples

- Venous blood will be obtained by any standard phlebotomy technique from a peripheral access point or from a central line by trained personnel (three 6 ml K2-EDTA Vacutainer);
- K2-EDTA tubes will be centrifuged at room temperature for 10 min at 1600 (±150) g. Time between blood collection and plasma/blood cell processing should to be less than 4 hours;
- The supernatant of the three EDTA tubes will be transferred to one fresh 15 ml centrifuge tube without disturbing the cellular layer using a disposable 10 ml serological pipette or disposable bulb pipette (sufficient residual plasma should be left in the tubes after the centrifugation without disturbing the leukocyte layer when pipetting);
- The plasma will be centrifuged in the 15 ml centrifuge tube at room temperature for 10 min at 3000 (±150) g;
- The supernatant will be transferred to a fresh 15 ml centrifuge tube without disturbing the cellular layer using a disposable 5 ml or 10 ml serological pipette or disposable bulb pipette (a residual volume of about 0.3 ml (~7 mm) on the bottom of the 15 ml tube should be left in order to avoid contamination of the plasma with cells);
- Plasma tubes will be stored at -80°C.

Collection of tumor samples

Formalin-fixed paraffin-embedded tumour blocks (or 10 slides of conventional thickness and polarity for IHC and 10 slides of 10 micron thickness for molecular biology) of primary and/or metastatic sites will be collected according to standard guidelines. Tumor tissues will be sent to the Coordinating Center (U.O. Oncologia Medica 2 Universitaria-AOUP) for collection and anonymization. DNA will be extracted by means of standard commercial kits and analyzed as per

standard practice by means of Pyrosequencing or Sequenom MassARRAY.

Circulating cell-free tumor DNA analysis

Treatment with target-specific drugs may have the potential to select cells with acquired drug resistance due to the selective pressure on tumor growth. Therefore, treatment may require periodic monitoring finalized to the early identification of resistance at molecular level due to the occurrence of secondary oncogenic drivers mutations including *KRAS*, *NRAS*, *BRAF*, PIK3CA.

A plasma sample (5-10 ml, see previous indications) will be obtained at baseline, before the administration of the 2nd cycle, at first CT-scan evaluation and at progression. Circulating cell-free tumor DNA (cftDNA) will be extracted from plasma using the QIAamp Circulating Nucleic Acid Kit (Qiagen). DNA will be stored at -20°C until the cftDNA analysis, which will be performed by a digital droplet PCR (BioRad). This biomarker study will serve as an indication of the role of *RAS* mutations in treatment response and resistance.

12. SAFETY ISSUES

DOSE REDUCTIONS AND DELAYS

Toxicities should be evaluated according to CTCAEv4.03.

Once a dose has been reduced it should not be increased at a later time.

DOSE MODIFICATIONS FOR TOXICITIES ATTRIBUTABLE TO IRINOTECAN

EVENT AT THE START OF SUBSEQUENT CYCLES OF THERAPY	*GRADE*	*ADJUSTMENT*
WBC	<3.000/mm^3	Hold until resolution
Neutrophils	<1.000/mm^3	
Platelets	<100.000/mm^3	
Diarrhea	≥1	
Mucositis	≥1	
Any other non-hematological toxicity	≥2	

PREVIOUS EVENT	*GRADE*	*ADJUSTMENT*
Neutropenia >5 days	4	**75%**
Febrile Neutropenia	4	
Thrombocytopenia	≥3	
Diarrhea	3	**75%**
Diarrhea	4	**50%**

DOSE MODIFICATIONS FOR TOXICITIES ATTRIBUTABLE TO CETUXIMAB, ACCORDING TO INVESTIGATOR JUDGMENT

For subjects who experience toxicities while on study, one or more doses of cetuximab may need to be withheld, reduced or delayed (administered at >14 day intervals). Cetuximab dose reduction are listed in the table below.

EVENT	*GRADE*	*ADJUSTMENT*
Skin or nail toxicity – *First Occurrence*	3 or 4	Hold cetuximab until grade ≤ 2 and restart at 100% dose level
Skin or nail toxicity in patients treated at 100% or 80% dose level – *Recurring*	3 or 4	Restart cetuximab at 80% dose level or 60% dose level respectively
Symptomatic hypomagnesemia – *First Occurence*		Hold cetuximab until resolution and restart at 100% dose level<br>Mg^{++} supplementation
Symptomatic hypomagnesemia in patients treated at 100% or 80% dose level – *Recurring*		Restart cetuximab at 80% dose level or 60% dose level respectively<br>Mg^{++} supplementation
Diarrhea – *First Occurence*	3 or 4	Hold cetuximab until resolution and restart at 100% dose level
Diarrhea in patients treated at 100% or 80% dose level – *Recurring*	3 or 4	Restart cetuximab at 80% dose level or 60% dose level respectively
Any non-hematologic toxicity	4	Hold cetuximab until resolution

CETUXIMAB DOSE REDUCTION FOR SKIN REACTIONS SCHEME

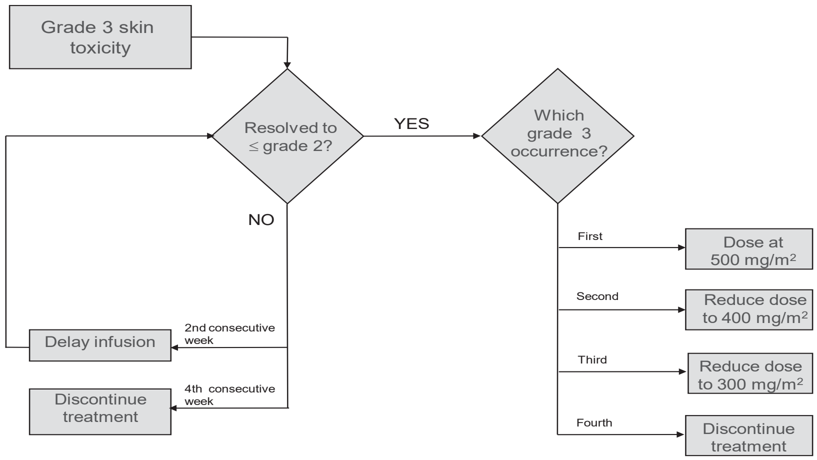

CRITERIA FOR WITHHOLDING A DOSE OF CETUXIMAB

For subjects who experience a toxicity that meets the criteria for withholding a dose of cetuximab:

- Subjects are allowed to have one subsequent dose withheld for toxicity, as per the scheme shown above. Even if the toxicity has resolved by the intervening week before the next cycle of chemotherapy is due, cetuximab will be restarted along with chemotherapy.

The cetuximab dose (100% or reduced) will be defined according to the scheme shown above and described below:

- Subjects treated at 100% dose level whose toxicity resolves after 1 dose of cetuximab is withheld should be restarted at 100% dose level (recommended but not required, reduction to 80% dose is allowed as an alternative to rechallenge with 100% dose);
- If toxicity recurs, subjects treated at 100% dose or 80% (400 mg/m^2) dose should be restarted at 80% dose or 60% (300 mg/m^2) dose, respectively, if the toxicity has resolved after withholding 1 dose of cetuximab;
- Subjects who experience grade 3 toxicity at the 60% dose level (300 mg/m^2) will not be retreated with cetuximab.

Patients, who must have a delay of cetuximab administration beyond 4 weeks from the previous dose of cetuximab (2 consecutive missed doses) due to toxicity, will be considered unable to tolerate cetuximab and will not be retreated with cetuximab.

Cetuximab should be given on the first day of each chemotherapy cycle. If a cycle of chemotherapy is delayed, cetuximab administration should be also delayed. If chemotherapy is

delayed greater than 4 weeks from the previous chemotherapy dose, and the patient does not have disease progression, cetuximab monotherapy should be administered as soon as possible.

Delay of cetuximab administration greater than 4 weeks from the previous dose of cetuximab are not allowed.

If cetuximab is delayed greater than 4 weeks from the previous administration, and the patient does not have disease progression, irinotecan monotherapy could be continued at physician discretion.

CONCOMITANT MEDICATIONS AND MANAGEMENT OF SPECIFIC TOXICITIES

ACUTE CHOLINERGIC SYNDROME

Atropine sulfate can be used, at the discretion of the investigator, as secondary prophylaxis or therapy of early onset cholinergic syndrome induced by irinotecan. Secondary prophylactic or therapeutic administration of 0.25-1 mg of intravenous or subcutaneous atropine can be considered (unless clinically contraindicated) in patients experiencing rhinitis, increased salivation, miosis, lacrimation, diaphoresis, flushing, abdominal cramping, or diarrhea (occurring during or shortly after infusion of irinotecan).

ANTIEMETIC PROPHYLAXIS

- Day 1 before chemotherapy: 5HT antagonist i.v. + dexamethasone 16 mg i.v.
- Day 2 in the morning: oral/i.m. 5HT antagonist or metoclopramide + dexamethasone 8 mg i.m.

INFUSION RELATED REACTION PROPHYLAXIS

- Day 1 before Cetuximab: Anti-histamine and Corticosteroid (8mg dexamethasone or equivalent). This must be given for the first 3 cycles and is strongly recommended for all subsequent cycles.

TREATMENT OF DIARRHOEA

Irinotecan can induce both early and late forms of diarrhea that appear to be mediated by different mechanisms. Early diarrhea (occurring during or shortly after infusion of irinotecan) is cholinergic in nature. It is usually transient and only infrequently is severe. It may be accompanied by symptoms of rhinitis, increased salivation, miosis, lacrimation, diaphoresis, flushing, and intestinal hyperperistalsis that can cause abdominal cramping. Early diarrhea and other cholinergic symptoms may be ameliorated by administration of atropine (0.25 mg SC). Atropine should not be given prophylactically during cycle 1. Late diarrhea (generally occurring more than 24 hours after administration of irinotecan) can be prolonged, may lead to dehydration and electrolyte imbalance, and can be life-threatening. Patients and patients' caregivers should be carefully informed of possible severe toxic effects such as diarrhea and abdominal cramps. Each patient should be instructed to have loperamide readily available and to begin treatment for late diarrhea (generally

occurring more than 24 hours after administration of irinotecan) at the first episode of poorly formed or loose stools or the earliest onset of bowel movements more frequent than normally expected for the patient. The patient should also be instructed to notify the Investigator if diarrhea or abdominal cramps occur. If diarrhea persists for more than 24 hours despite loperamide, the patient should be instructed to take a fluoroquinolone antibiotic and to re-contact the treating Investigator. The patient should be hospitalised for parenteral support and loperamide should be replaced by another anti-diarrheal treatment (e.g. octreotide). Patients should have a supply of fluoroquinolone antibiotic available at home. The recommended dosage regimen for loperamide previously used in irinotecan clinical trials consists of the following: 4 mg at the first onset of late diarrhea and then 2 mg every 2 hours until the patient is diarrhea-free for at least 12 hours. Note: This dosage regimen exceeds the usual dosage recommendations for loperamide. Premedication with loperamide is not recommended. If diarrhea occurs it is of vital importance that measures are taken to avoid dehydration and electrolyte imbalance. Patients should be supported as clinically indicated. The use of drugs with laxative properties should be avoided because of the potential for exacerbation of diarrhea. Patients should be advised to contact their Investigator to discuss any laxative use. Abdominal cramps should be treated the same as for diarrhea.

CETUXIMAB SPECIFIC INFUSION RELATED REACTIONS

Cetuximab must be administered under the supervision of a physician experienced in the use of antineoplastic medicinal products. Close monitoring is required during the infusion and for at least one hour after the end of the infusion. Availability of resuscitation equipment must be ensured.

Symptoms usually occur during the first infusion and up to one hour after the end of infusion, but may occur after several hours or with subsequent infusions. Occurrence of a severe infusion related reaction requires immediate and permanent discontinuation of cetuximab therapy and may necessitate emergency treatment. In each case of an infusion related reaction, the investigator should implement treatment measures according to the best available medical practice. Based on previous experience with cetuximab infusion related reactions, the treatment guidelines as described in the following table may be applicable.

CTCAE v4.03 grades/symptoms	Action
NCI CTCAE grade 1:<br>Mild transient reaction (transient flushing or rash, drug fever < 38°C)	Decrease cetuximab infusion rate by 50% and monitor closely for any worsening, decrease further if reactions persist as applicable:<br>- 1st dose: decrease infusion rate by 50%<br>- 2nd dose: decrease infusion rate by 50%, if infusion related reaction persists decrease infusion rate by another 25%<br>- Subsequent doses: decrease infusion rate by 50%, if infusion related reaction persists decrease infusion rate by another 50%<br>**The total infusion time for cetuximab should not exceed 4 hours.**

<table>
<tr><th>CTCAE v4.03 grades/symptoms</th><th>Action</th></tr>
<tr><td>NCI CTCAE grade 2:
Rash, flushing, urticaria, dyspnea, drug fever ≥ 38°C.
Promptly responsive to interruption of infusion and symptomatic treatment.</td><td>Stop cetuximab infusion.
Administer bronchodilators, oxygen, i.v. fluids, antihistamines, etc. as medically indicated.
Resume infusion at 50% of previous rate once infusion related reaction has resolved or decreased to grade 1 in severity, and monitor closely for any worsening.
Prolongation of infusion duration should be performed as described for grade 1 reactions, as applicable.
The total infusion time for cetuximab should not exceed 4 hours.
At second occurrence, cetuximab will be discontinued.</td></tr>
<tr><td>NCI CTCAE grade 3:
Symptomatic bronchospasm, allergy-related edema/angioedema, hypotension.
Not rapidly responsive to brief interruption of infusion and/or to symptomatic medication; recurrence of symptoms following initial improvement; hospitalization, indicated for clinical sequelae.</td><td rowspan="2">Stop cetuximab infusion immediately and disconnect infusion tubing from the patient.
Administer epinephrine, bronchodilators, antihistamines, glucocorticoids, intravenous fluids, vasopressor agents, oxygen, etc., as medically indicated.
The patient should not receive any further cetuximab treatment.</td></tr>
<tr><td>NCI CTCAE grade 4:
Anaphylaxis.
Life-threatening consequences; urgent intervention indicated.</td></tr>
</table>

Re-treatment following infusion related reactions: once a cetuximab infusion rate has been decreased due to an infusion related reaction, it will remain decreased for all subsequent infusions. If the patient has an infusion related reaction with the slowest infusion rate, the infusion should be stopped, and the patient must not receive any further cetuximab treatment. If a patient experiences a grade 3 or 4 infusion related reaction at any time, cetuximab should be discontinued.

If there is any question as to whether an observed reaction is an infusion related reaction of grades 1-4, one of the trial chairs should be contacted immediately to discuss and grade the reaction.

INTERSTITIAL PNEUMONITIS

Severe interstitial pneumonitis has been described in subjects treated with the EGFR-pathway targeting therapy gefitinib. To date, no increased risk of interstitial pneumonitis has been identified with cetuximab. Nevertheless, all subjects should have adequate chest imaging prior to commencing cetuximab therapy, as a safety precaution in order to document the baseline pulmonary condition. If there are respiratory symptoms at study entry, lung function tests and further diagnostic procedures should also be undertaken in order to diagnose pre-existing pulmonary fibrosis or interstitial pneumonitis. Furthermore, should pulmonary symptoms appear or worsen during or after cetuximab treatment, a detailed description is required and investigators should use their discretion in ordering such diagnostic procedures as are necessary to elicit an accurate diagnosis.

MANAGEMENT AND TREATMENT OF SKIN TOXICITY

Some educational and general interventions should be used in all patients:

- Sunscreen (avoid sun exposure, use protective products for the exposed areas)
- Avoid habits or products that cause dry skin (hot water, alcohol-based cosmetics)
- Try to maintain skin at maximum hydration (i.e using bath oils, etc.).
- Use warm water
- Frequent use of emollient creams alcohol free
- Use of Tocopherol acetate oil or gel
- Avoid tight shoes
- Do frequent checks
- Avoid the beard growth with regular shaving; use sharp razor multi-blade; use pre-shave creams, emollients and moisturizers after-shave, do not use alcoholic after-shave and electric shaver.

As general and prophylactic intervention maybe include the daily use of Vit K1 cream, beginning day-1 (one day before the administration of the first cetuximab dose) and continued through all the anti EGFR treatment period, applied to face, hands, feet, neck, back, and chest twice daily.

Vitamin K1:

Much evidence has been presented on the beneficial effect of vitamin K1 cream on patients experiencing severe anti-EGFr induced acne-like rash.

This evidence demonstrates that the twice a day use of Vitamin K1 cream in prophylactic or reactive approach improves cutaneous toxicity. The median improvement time in reactive K1 use is 8 - 18 days to observe down-staging in rash at least by 1 grade without reducing the cetuximab dose. No local or systemic toxicity from topical use of Vitamin K1 cream was observed.

The prophylactic approach showed that the twice daily use of Vitamin K1 cream from the beginning of anti EGFR treatment decreases the skin toxicity grade incidence (no grade 3 or 4 recorded in a group of 48 patients).

For these reasons, general and prophylactic intervention may include the daily use of Vit K1, beginning day-1 (one day before the administration of the first cetuximab dose) and continued throughout the anti EGFR treatment period, applied to face, hands, feet, neck, back, and chest twice daily.

Table 1: Management of skin rash grade 1

Skin lesions and symptoms	Papules, pustules, or symptom-free erythema
Cetuximab dose modifications	**No**
Topical treatment	**No**
Systemic treatment	**No**
Intervention	General educational and prophylactic measures

Table 2: Management of skin rash grade 2

Skin lesions and symptoms	Eruption with papules (Grade 2A) or pustules (Grade 2B) covering <50% of body surface, with moderate symptoms, and that does not interfere with daily activities
Cetuximab dose modifications	NO
Topical treatment	Antibiotics: clindamycin 1% gel, erythromycin 3% gel/cream, metronidazole 0.75-1% cream/gel, twice/day until regression to grade 1 (avoid benzoyl peroxide products).<br>Lesions of the scalp: erythromycin 2% lotion
Systemic treatment	*Prevalence of papules (Grade 2A)*<br>No<br>*Prevalence of pustules (Grade 2B)*<br>Antibiotics: minocycline 100 mg per os once/day, doxycycline 100 mg per os once/day for ≥ 4 weeks and until the rash is asymptomatic.

Table 3: Management of skin rash grade 3

Skin lesions and symptoms	Eruption with papules (Grade 3A) or pustules (Grade 3B) covering > 50% of body surface; severe symptoms that interfere with daily activities
Cetuximab dose modifications	*First occurrence*: delay cetuximab infusion for ≤ 14 days until the skin rash improves to grade ≤ 2. If there is an improvement, continue at 100% dose. If there is no improvement in 28 days since the previous infusion, discontinue therapy.<br>*Second occurrence*: delay cetuximab infusion for ≤ 14 days until the skin rash improves to grade ≤ 2. If there is an improvement, continue at reduced dose of 400 mg/m2. If there is no improvement, discontinue therapy.<br>*Third occurrence:* delay cetuximab infusion for ≤ 14 days until the skin rash improves to grade ≤ 2. If there is improvement, continue at reduced dose of 300 mg/m2. If there is no improvement, discontinue therapy.<br>*Fourth occurrence*: discontinue therapy definitively.
Topical treatment	Antibiotics: clindamycin 1% gel, erythromycin 3% gel/cream, metronidazole 0.75-1% cream/gel, twice/day until regression to grade 1 (avoid benzoyl peroxide products).<br>Lesions of the scalp: erythromycin 2% lotion
Systemic treatment	Antibiotics: minocycline 100 mg per os once/day, doxycycline 100 mg per os once/day for ≥ 4 weeks and until the rash is symptomatic.<br>Corticosteroids: according to investigator judjement, methylprednisolone 8 mg per os once or twice/day or prednisone 25 mg per os once/day , for up to 10 days can be administered.
Systemic treatment in highly symptomatic/non-responsive patients	Retinoids: isotretinoin 0.3-0.5 mg/kg per os<br>Corticosteroids: methylprednisolone, or dexamethasone iv<br>Antihistamines: clorfenamine im/iv<br>Antibiotics: amoxicillin/clavulanic acid, gentamicin iv<br>Intravenous hydration

Table 4: Management of skin rash grade 4

Skin lesions and symptoms	Generalized rash; severe symptoms that require emergency treatment
Cetuximab dose modifications	Discontinue therapy immediately and definitively
Topical treatment	Antibiotics: clindamycin 1% gel, erythromycin 3% gel/cream, metronidazole 0.75 to 1% cream/gel, 2 times daily until regression to grade 1 (avoid benzoyl peroxide products).<br>Lesions of the scalp: erythromycin 2% lotion
Systemic treatment	Retinoids: isotretinoin 0.3-0.5 mg/kg per os<br>Corticosteroids: methylprednisolone, dexamethasone iv<br>Antihistamines: clorfenamine im/iv<br>Antibiotics: amoxicillin/clavulanic acid, gentamicin iv<br>Intravenous hydration<br>Hopsitalization

EXTRAVASATION

No severe extravasation reactions have been observed so far with irinotecan. As a general recommendation, in the event of extravasation, the following advice should be observed (like for any drug):

1. stop the infusion immediately,
2. do not remove the needle or cannula,
3. aspirate as much infiltrated drug as possible from the subcutaneous site with the same needle,
4. apply ice to the area for 15 to 20 minutes every 4 to 6 hours for the first 72 hours,
5. watch the area closely during the following days in order to determine whether any further treatment is necessary.

HEMATOPOIETIC GROWTH FACTORS

May be used to treat symptomatic neutropenia but should not be used prophylactically before the 1st cycle. The prophylactic use could be considered in case of:

- Previous febrile neutropenia;
- Previous grade 4 neutropenia for 5 days or more;
- More than 2 delays due to neutropenia

ELECTROLYTE MANAGEMENT

Subjects should be evaluated as outlined in Section "*Baseline and on-treatment evaluations* " and managed as per local medical practice. If hypomagnesemia is present, replacement should be managed with either oral or parental replacement, or both, according to institutional practice and to

the degree of hypomagnesemia present. It is recommended that subject's serum magnesium level should be maintained within the normal range during study treatment.

It is important to assess and manage serum potassium and calcium (adjusted for albumin) in subjects who have concomitant hypomagnesemia. A subject's serum potassium and calcium parameters are recommended to be maintained, as per local medical practice, within the normal ranges during study treatment.

13. ETHICAL ISSUES

This protocol is in accordance with the principles laid down by the 18th World Medical Assembly (Helsinki, 1964) and amendments laid down by the 29th (Tokyo 1975), the 35th (Venice, 1983), the 41st (Hong Kong, 1989), the 48th (Somerset West, 1996) and the 52nd (Edinburgh, 2000) World Medical Assemblies (see appendices).

INFORMED CONSENT

The investigator must explain to each patient (or legally authorised representative) the nature of the study, its purpose, the procedures involved, the expected duration, the potential risks and benefits involved and any discomfort it may entail. Each patient must be informed that participation in the study is voluntary and that he/she may withdraw from the study at any time and that withdrawal of consent will not affect her subsequent medical treatment or relationship with physician. The informed consent will be given by means of standard written statement, written in non-technical language. The patient should read and consider the statement before signing and dating it, and should be given a copy of the signed document. If the subject cannot read or sign the document, oral presentation may be made or signature given by the subject’s legally appointed representative, if witnessed by a person not involved in the study, mentioning that the patient could not read or sign documents. No patient can enter the study before his/her informed consent has been obtained. The informed consent is part of the protocol and must be submitted by the investigator with to the local ethical committee.

A copy of the patient's signed written consent will be kept by the center in the proper section of the Investigator Site File.

PATIENT PROTECTION

The names of patients will not be recorded; a sequential identification number will be attributed to each patient registered in the trial. This number will identify the patient and must be included on all Case Report Forms.

In order to avoid identification errors, patients initials (maximum of 3 letters) and date of birth will also be reported on the Case Report Forms.

Investigators will guarantee that all persons involved in this study will respect the confidentiality of any information concerning the trial subject.

All parties involved in this clinical trial will maintain the strict confidentiality to assure that neither the person nor the family privacy of the patient participating in the trial is violated; appropriate measures shall be taken to avoid the access of non authorized persons to the trial data. The processing of the personal data of patients taking part in the trial, and in particular regarding data concerning consent, shall comply with local law on the privacy (Legge delega 127/2001) and with the European Directive on the Privacy of data (95/46/EC).

The patient can withdraw consent whenever he wants and further data will not be collected, even if the already collected data will be used for the study's analyses.

CONFIDENTIAL SUBJECT INFORMATION FOR GENETIC ANALYSIS

For the storage of biological samples, e.g. of tumor tissue samples in a bio bank, specific means will be taken to ensure the subject's right to privacy and the pertinent guidance documents and regulations will be considered.

Subjects may withdraw their consent to store the biological samples .

If the patient withdraws his consent from the study within 5 years, the biological samples will be destroyed.

After 5 years, biological samples will be anonymized completely. At that time the samples cannot be identified in any way.

The samples will be maintained for potential analysis for 15 years from the acquisition. Samples will be destroyed according to GONO policies and procedures.

Samples will be collected and sent to the laboratory designated for the trial where they will be processed.

Blood and plasma samples will be processed, analyzed and stored at U.O. Farmacologia Clinica of Azienda Ospedaliero-Universitaria Pisana under the responsibility of Laboratory's Director.

Tumor tissue samples will be processed and analyzed at U.O. Anatomia e Istologia Patologica III of Azienda Ospedaliero-Universitaria Pisana under the responsibility of Laboratory's Director.

The tumor tissue samples will be stored at Dipartimento di Ricerca Traslazionale e delle Nuove Tecnologie (U.O. Oncologia Medica 2 Universitaria of Azienda Ospedaliero-Universitaria Pisana) under the responsibility of Laboratory's Director.

To maintain privacy of information collected from samples obtained for storage and future analysis, GONO has developed secure policies and procedures to maintain subject privacy. At the clinical site, a unique Code will be placed on the blood sample for transfer to the storage facility. The Code is a random number used only to identify the biosample of each subject. No other personal identifiers will appear on the sample tube. The first Code will be replaced with a Sample Code at the Central Laboratory or at the GONO designated facility. This sample is now a single coded sample. The Sample Code is stored separately from all previous sample identifiers. A secure code, hereinafter referred to as a "first coding key", will be utilized to match the Sample Code to the original blood code and subject number to allow clinical information collected during the course of the trial to be associated with the biosample. This "first coding key" will be transferred by the central laboratory or GONO designated facility under secure procedures to the GONO designated as the entrusted keyholder to maintain confidentiality of the biosamples. The Sample Code will be logged into the primary biorepository database, and in this database this identifier will not have identifying demographic data or identifying clinical information (i.e., race, sex, age, diagnosis, lab

values) associated with it. The sample will be stored in a designated repository site with secure policies and procedures for sample storage and usage.

ETHICS COMMITTEE (EC)

The Investigator must submit this protocol to the local Ethics Committee and is required to forward a copy of the written approval to the CRP.

The EC approval must report, the identification of the trial (title, protocol number and version), the documents evaluated (protocol, informed consent material, advertisement when applicable) and the date of their version.

ADMINISTRATIVE RESPONSIBILITIES

The Coordinating Center (U.O. Oncologia 2 Universitaria – Polo Oncologico Azienda Ospedaliero-Universitaria Pisana, AOUP) and the Data Center (U.O. Oncologia 2 Universitaria – Polo Oncologico Azienda Ospedaliero-Universitaria Pisana, AOUP) will be responsible for:

- reviewing the protocol;
- centralizing databases;
- centralizing data validation;
- controlling the quality of the reported data;
- emitting Data Query Forms;
- generating study program reports;
- generating the Statistical Analysis Plan;
- perform statistical analysis.

TRIAL SPONSORSHIP AND FINANCING

The present study is an investigator-initiated trial**,** carried out by participating clinicians, who have the intellectual ownership of the results.

The study is sponsored by:

- Gruppo Oncologico Nord-Ovest (G.O.N.O.) Cooperative Group Via G. Mameli, 3 – Genoa (ITALY), who will provide the economic support for costs related to data management, statistical analysis and the other activities of central and group coordinating centers.
- MERCK.

No funds can be provided to ethical committees and single participating centers.

The study will be conducted according to the current regulations.

14. STUDY MONITORING

QUALITY ASSURANCE

Each participating Investigator will be responsible for ensuring data quality. Each reported information will be systematically checked for consistency, completeness and accuracy by the Coordinating Center that will issue Data Query Forms in case of inconsistent data. Local quality control will be provided by each participating group, which will be responsible for monitoring the centers.

RESPONSIBILITIES OF THE INVESTIGATORS

The Investigators will perform the study in accordance with ICH Good Clinical Practice and Good Clinical Practice for Trials on Medicinal Products in the European Community (ISBN 92 - 825-9563-3).

The Investigator is required to ensure his compliance to the procedures required by the protocol with respect to the investigational drug schedule and visit schedule. The Investigator agrees to provide all information requested in the Case Report Form in an accurate manner.

The Investigator has responsibilities to the Health Authorities to take all reasonable steps to ensure the proper conduct of the study as regards ethics, protocol adherence, integrity and validity of the data recorded on the case report forms.

At regular intervals during the study, the center will be contacted, through site visits, letters or telephone calls, to review the study progress, the investigators and subjects adherence to protocol requirements. The following points will be scrutinized:

- subject informed consent, recruitment and follow-up;
- subject compliance to the study treatment;
- study treatment accountability;
- Adverse Event documentation and reporting.

SOURCE DOCUMENT REQUIREMENTS

According to the guidelines on ICH Good Clinical Practice, monitors from the Coordinating Center will check the case report form entries against the source documents. These personnel, bound by professional secrecy, will not disclose any personal identity or personal medical information.

USE AND COMPLETION OF CASE REPORT FORMS (CRFs)

It is the responsibility of the Investigator to prepare and maintain adequate and accurate CRFs for each patient enrolled in the study. All CRFs should be completed to ensure accurate interpretation of data.

15. ADVERSE EVENTS

DEFINITION OF AN ADVERSE EVENT

An adverse event is defined in the International Conference on Harmonisation (ICH) Guideline for Good Clinical Practice as “any untoward medical occurrence in a patient or clinical investigation subject administered a pharmaceutical product and that does not necessarily have a causal relationship with this treatment.” (ICH E6:1.2).

The investigator is responsible for reviewing laboratory test results and determining whether an abnormal value in an individual study subject represents a change from values before the study. Abnormal laboratory findings without clinical significance (based on the investigator's judgment) should not be recorded as adverse events; however, laboratory value changes requiring therapy or adjustment in prior therapy are considered adverse events.

Patients will be instructed by the Investigator to report the occurrence of any adverse event.

ADVERSE DRUG REACTIONS (ADR)

All untoward and unintended responses to a medicinal product related to any dose administered.

The phrase “responses to a medicinal product” means that a causal relationship between the medicinal product and the adverse event is at least a reasonable possibility, i.e. the relationship cannot be ruled out. A serious ADR (SADR) is an ADR that meets the definition of serious.

DEFINITION OF SERIOUS ADVERSE EVENT

A serious adverse event (SAE) is defined as an adverse event that:

• is fatal;

• is life-threatening (places the subject at immediate risk of death);

• requires in-patient hospitalization or prolongation of existing hospitalization;

• results in persistent or significant disability/incapacity;

• is a congenital anomaly/birth defect;

• other significant medical hazard.

’Hospitalization’ meeting the regulatory definition of “serious” is any in-patient hospital admission that includes a minimum of an overnight stay in a health care facility. Any adverse event that does not meet one of the definitions of serious (i.e., emergency room visit, outpatient surgery, or requires urgent investigation) may be considered by the investigator to meet the “other significant medical hazard” criterion for classification as a serious adverse event. Examples include allergic bronchospasm, convulsions, and blood dyscrasias.

Hospitalization for the performing of protocol-required procedures or administration of study treatment is not classified as a SAE.

All adverse events which do not meet any of the criteria for serious should be regarded as non-serious adverse events.

All serious adverse events occurring during the study treatment period must be reported according to the procedure described below. Any late SAE (occurring within 30 days after the last treatment administration) possibly or probably related to the study treatment should follow the same reporting procedure.

Progression of colorectal cancer leading to one of the above should not be reported as a serious adverse event.

DEATH ON STUDY

Any death occurring between the *registration* and 30 days following the *treatment* must be reported to the Sponsor within 24 hours, as a Serious Adverse Event, regardless of the relation to study treatment. The Sponsor must notify this SAE to *CE coordinating center* by fax within 1 working day. Deaths occurring during the study follow-up period (i.e. later than 30 days after the last infusion) need only to be reported as serious adverse event if it is thought that there is a possible relation to the study treatment (possible, probable). All deaths should be reported on the death report form section of the CRF regardless of cause.

RELATIONSHIP WITH TRIAL MEDICATION

Relationship	Description
UNRELATED	There is no evidence of any causal relationship
UNLIKELY	There is little evidence to suggest there is a causal relationship (e.g. the event did not occur within a reasonable time after administration of the trial medication). There is another reasonable explanation for the event (e.g. the patient’s clinical condition, other concomitant treatments).
POSSIBLE	There is some evidence to suggest a causal relationship (e.g. because the event occurs within a reasonable time after administration of the trial medication). However, the influence of other factors may have contributed to the event (e.g. the patient’s clinical condition, other concomitant treatments).
PROBABLE	There is evidence to suggest a causal relationship and the influence of other factors is unlikely.
DEFINITELY	There is clear evidence to suggest a causal relationship and other possible contributing factors can be ruled out.
NOT ASSESSABLE	There is insufficient or incomplete evidence to make a clinical judgement of the causal relationship.

REPORTING PROCEDURE

REPORTING PROCEDURES FOR ALL ADVERSE EVENTS

The investigator is responsible for ensuring that all adverse events are properly captured in the subjects' medical records.

The following adverse event attributes must be assigned by the investigator: adverse event diagnosis or syndrome (if known), or signs/symptoms (if not known); appropriate event description; dates of onset and resolution; severity; assessment of relatedness to study treatment; actions taken.

Medically significant adverse events considered related to the study treatment by the investigator or the sponsor will be followed until resolved or considered stable.

It will be left to the investigator's clinical judgment to determine whether an adverse event is related and of sufficient severity to require the subject's removal from treatment or from the study. A subject may also voluntarily withdraw from treatment due to what he or she perceives as an intolerable adverse event. If either of these situations arises, the subject should be strongly encouraged to undergo an end-of-study assessment and be under medical supervision until symptoms cease or the condition becomes stable.

SERIOUS ADVERSE EVENTS REPORTING PROCEDURES

Serious adverse events will be collected and recorded throughout the study period, defined as through to 30 days after the last dose of study treatment or the end of the study (including the follow-up period), whichever is longer.

The investigator should notify the Sponsor of all serious adverse events occurring at the site in accordance with local procedures, statutes and the European Clinical Trial Directive. The Sponsor will medically review all SAEs.

The Sponsor will ensure the notification of the appropriate Ethics Committees, Competent Authorities and participating Investigators of all serious adverse events occurring at the site in accordance with local legal requirements, statutes and the European Clinical Trial Directive.

Details should be documented on the specified Serious Adverse Event Form.

Mail a .pdf scan version to:

trials.office.pisa@gmail.com

Mail subject should report: SAE Cricket - "Name of Participating Center"

The Sponsor will also send the report to National Authorities, Ethic Committees (EC) and investigators as appropriate, according to local regulations.

FOLLOW-UP

Patients withdrawn from the study treatment due to any adverse event will be followed at least until the outcome is determined, even if it implies that the follow-up continues after the patients has left the trial, and where appropriate, until the end of the planned period of follow-up.

In case of serious adverse event, the patient must be followed until complete clinical recovery and laboratory results have returned to normal, or until symptoms have stabilized. This may imply that the follow-up will continue after the patient has left the trial.

Further information will be noted on the SAE form, by ticking the box marked “follow-up” and will be sent to the Coordinating Center as information becomes available.

The Sponsor shall supply Merck Serono with a copy of any serious individual case safety report regardless of the causality assessment concerning the Pharmaceutical Product administration. Serious individual case safety reports shall include reports whether or not associated with a technical complaint, reports on drug interaction, reports on suspected transmission of an infectious agent by the product. Further, Sponsor shall provide reports of the following special situations whether associated or not with an Adverse Event (“Adverse Events” or “AEs”): medication errors, overdose, abuse, misuse, off- label use, occupational exposure, pregnancy and breast feeding notification and lack of efficacy reports.

In addition, Merck Serono will perform a medical assessment for the purpose of signal detection and cumulative reporting of safety information and will also request follow-up information from the Sponsor as needed.

The Sponsor shall provide Merck Serono Global Drug Safety on a quarterly basis with information on Adverse Events (AEs), safety laboratory data, and any efficacy data necessary to assess the safety of the Pharmaceutical Product.

The Sponsor shall inform Merck Serono of any pregnancy occurring in a subject treated with the Pharmaceutical Product during the course of the Study. The Sponsor shall ensure that the case is followed up to the end of the pregnancy and provide all relevant documentation and a final report on the outcome to Merck Serono.

Merck Serono will perform regular signal detection on the Pharmaceutical Product in its global safety database. In case of any action arising from such signal detection activities which is relevant for the conduct of the Study, Merck Serono will inform the Sponsor in a timely manner.

16. REFERENCES

1. Van Cutsem E, Kohne CH, Hitre E, et al. Cetuximab and chemotherapy as initial treatment for metastatic colorectal cancer. N Engl J Med. 2009;360: 1408-1417.
2. Van Cutsem E, Kohne CH, Lang I, et al. Cetuximab plus irinotecan, fluorouracil, and leucovorin as first-line treatment for metastatic colorectal cancer: updated analysis of overall survival according to tumor KRAS and BRAF mutation status. J Clin Oncol.29: 2011-2019.
3. Bokemeyer C, Bondarenko I, Makhson A, et al. Fluorouracil, leucovorin, and oxaliplatin with and without cetuximab in the first-line treatment of metastatic colorectal cancer. J Clin Oncol. 2009;27: 663-671.
4. Bokemeyer C, Bondarenko I, Hartmann JT, et al. Efficacy according to biomarker status of cetuximab plus FOLFOX-4 as first-line treatment for metastatic colorectal cancer: the OPUS study. Ann Oncol.22: 1535-1546.
5. V. Heinemann, LF Von Weikersthal, T Decker, et al. Randomized comparison of FOLFIRI plus cetuximab versus FOLFIRI plus bevacizumab as first-line treatment of KRAS wild-type metastatic colorectal cancer: German AIO study KRK-0306 (FIRE-3). J Clin Oncol 31, 2013 (suppl; abstr LBA3506) ASCO 2013. 2013.
6. Cunningham D, Humblet Y, Siena S, et al. Cetuximab monotherapy and cetuximab plus irinotecan in irinotecan-refractory metastatic colorectal cancer. N Engl J Med. 2004;351: 337-345.
7. Jonker DJ, O'Callaghan CJ, Karapetis CS, et al. Cetuximab for the treatment of colorectal cancer. N Engl J Med. 2007;357: 2040-2048.
8. Bokemeyer C, Van Cutsem E, Rougier P, et al. Addition of cetuximab to chemotherapy as first-line treatment for KRAS wild-type metastatic colorectal cancer: pooled analysis of the CRYSTAL and OPUS randomised clinical trials. Eur J Cancer.48: 1466-1475.
9. Karapetis CS, Khambata-Ford S, Jonker DJ, et al. K-ras mutations and benefit from cetuximab in advanced colorectal cancer. N Engl J Med. 2008;359: 1757-1765.
10. Amado RG, Wolf M, Peeters M, et al. Wild-type KRAS is required for panitumumab efficacy in patients with metastatic colorectal cancer. J Clin Oncol. 2008;26: 1626-1634.
11. Loupakis F, Ruzzo A, Cremolini C, et al. KRAS codon 61, 146 and BRAF mutations predict resistance to cetuximab plus irinotecan in KRAS codon 12 and 13 wild-type metastatic colorectal cancer. Br J Cancer. 2009;101: 715-721.
12. Peeters M, Oliner KS, Parker A, et al. Massively parallel tumor multigene sequencing to evaluate response to panitumumab in a randomized phase III study of metastatic colorectal cancer. Clin Cancer Res.19: 1902-1912.
13. Di Nicolantonio F, Martini M, Molinari F, et al. Wild-type BRAF is required for response to panitumumab or cetuximab in metastatic colorectal cancer. J Clin Oncol. 2008;26: 5705-5712.
14. Douillard JY, Oliner KS, Siena S, et al. Panitumumab-FOLFOX4 treatment and RAS mutations in colorectal cancer. N Engl J Med. 2013;369: 1023-1034.

15. Stintzing S, Jung A, Rossius L. Analysis of KRAS/NRAS and BRAF mutations in FIRE-3: A randomized phase III study of FOLFIRI plus cetuximab or bevacizumab as first-line treatment for wild-type (WT) KRAS (exon 2) metastatic colorectal cancer (mCRC) patients. Eur J Cancer. 2013;LBA 7.
16. Yokota T, Ura T, Shibata N, et al. BRAF mutation is a powerful prognostic factor in advanced and recurrent colorectal cancer. Br J Cancer. 2011;104: 856-862.
17. Tran B, Kopetz S, Tie J, et al. Impact of BRAF mutation and microsatellite instability on the pattern of metastatic spread and prognosis in metastatic colorectal cancer. Cancer. 2011;117: 4623-4632.
18. De Roock W, Claes B, Bernasconi D, et al. Effects of KRAS, BRAF, NRAS, and PIK3CA mutations on the efficacy of cetuximab plus chemotherapy in chemotherapy-refractory metastatic colorectal cancer: a retrospective consortium analysis. Lancet Oncol.11: 753-762.
19. Gattenlohner S, Etschmann B, Kunzmann V, et al. Concordance of KRAS/BRAF Mutation Status in Metastatic Colorectal Cancer before and after Anti-EGFR Therapy. J Oncol. 2009;2009: 831626.
20. Baldus SE, Schaefer KL, Engers R, Hartleb D, Stoecklein NH, Gabbert HE. Prevalence and heterogeneity of KRAS, BRAF, and PIK3CA mutations in primary colorectal adenocarcinomas and their corresponding metastases. Clin Cancer Res.16: 790-799.
21. Larue L, Bellacosa A. Epithelial-mesenchymal transition in development and cancer: role of phosphatidylinositol 3' kinase/AKT pathways. Oncogene. 2005;24: 7443-7454.
22. Santini D, Vincenzi B, Addeo R, et al. Cetuximab rechallenge in metastatic colorectal cancer patients: how to come away from acquired resistance? Ann Oncol. 2012;23: 2313-2318.
23. Sobrero AF, Maurel J, Fehrenbacher L, et al. EPIC: phase III trial of cetuximab plus irinotecan after fluoropyrimidine and oxaliplatin failure in patients with metastatic colorectal cancer. J Clin Oncol. 2008;26: 2311-2319.
24. Tournigand C, Andre T, Achille E, et al. FOLFIRI followed by FOLFOX6 or the reverse sequence in advanced colorectal cancer: a randomized GERCOR study. J Clin Oncol. 2004;22: 229-237.

G.O.N.O.

GRUPPO ONCOLOGICO NORD-OVEST

A PHASE II SINGLE-ARM STUDY OF CETUXIMAB PLUS IRINOTECAN AS RECHALLENGE 3RD-LINE TREATMENT OF *KRAS*, *NRAS* AND *BRAF* WILD-TYPE IRINOTECAN-PRETREATED METASTATIC COLORECTAL CANCER PATIENTS PROGRESSING AFTER AN INITIAL RESPONSE TO A 1ST-LINE CETUXIMAB-CONTAINING THERAPY AND A STANDARD 2ND-LINE

THE CRICKET STUDY

EUDRACT 2014-001126-15

APPENDICES

APPENDIX I: SYNOPSIS (ENGLISH VERSION)

A PHASE II SINGLE-ARM STUDY OF CETUXIMAB PLUS IRINOTECAN AS RECHALLENGE 3RD-LINE TREATMENT OF *KRAS*, *NRAS* AND *BRAF* WILD-TYPE IRINOTECAN-PRETREATED METASTATIC COLORECTAL CANCER PATIENTS PROGRESSING AFTER AN INITIAL RESPONSE TO A 1ST-LINE CETUXIMAB-CONTAINING THERAPY AND A STANDARD 2ND-LINE

(CRICKET) - EUDRACT 2014-001126-15

**Version**	2.0 – August, 2014
**Sponsor**	Gruppo Oncologico Nord-Ovest (G.O.N.O.)
**Setting**	Third line
**Protocol Phase**	Phase II single arm
**Indication**	Patients with unresectable, *KRAS, NRAS* and *BRAF* wild-type and irinotecan-pretreated metastatic colorectal cancer candidates for a 3rd-line treatment and having progressed on a 1st-line cetuximab-containing therapy.
**Study Rationale**	▪ Several phase II and III studies demonstrated that the combination of cetuximab with 1st-line chemotherapy improves response rate and progression-free survival in *KRAS* wild-type metastatic colorectal cancer patients.<br>▪ Cetuximab in combination with irinotecan is approved for the treatment of *RAS* wild-type, irinotecan-refractory metastatic colorectal cancer patients.<br>▪ A recent phase II study demonstrated a potential benefit from cetuximab rechallenge in irinotecan-refractory *KRAS* wild-type metastatic colorectal patients already progressed to a previous cetuximab-containing therapy.<br>▪ A recent retrospective analysis demonstrated the role of *RAS* mutations in the prediction of resistance to anti-EGFR treatment. Considering *BRAF* mutation, while its prognostic value is undisputed, its predictive role remains unclear.
**Endpoints**	**Primary:**<br>• To evaluate the activity in terms of response rate (RR)<br>**Secondary:**<br>• Progression-free survival (PFS)<br>• Overall survival (OS)<br>• Safety profile<br>• Evaluation of potential predictive and/or prognostic biomarkers
**Inclusion Criteria**	• Histologically proven diagnosis of colorectal adenocarcinoma;<br>• *RAS* and *BRAF* wild-type status;<br>• First-line irinotecan-based (FOLFIRI or FOLFOXIRI) cetuximab-containing therapy producing at least a partial response;<br>• First-line progression-free survival in response to cetuximab-containing therapy ≥6 months;<br>• Documentation of progression to first-line cetuximab within 4 weeks after last cetuximab administration;<br>• Time between the end of first-line therapy and the start of third-line treatment with cetuximab plus irinotecan ≥4 months;<br>• Second-line oxaliplatin-based (FOLFOXIRI, FOLFOX or XELOX)

**Inclusion Criteria (Cont.)**	bevacizumab-containing therapy;<br>• Documentation of progression to second-line treatment;<br>• Measurable disease according to RECIST criteria v1.1;<br>• Have tumor tissue (of primary tumor and metastases or at least one of the two) available for biomarker analysis;<br>• Male or female patients > 18 years of age;<br>• ECOG Performance Status ≤ 2;<br>• Life expectancy of at least 3 months;<br>• Adequate bone marrow, liver and renal function assessed within 14 days before starting study treatment;<br>• Women of childbearing potential must have a negative blood pregnancy test at the baseline visit. For this trial, women of childbearing potential are defined as all women after puberty, unless they are postmenopausal for at least 12 months, are surgically sterile or are sexually inactive;<br>• Subjects and their partners must be willing to avoid pregnancy during the trial and until 6 months after the last trial treatment. Male subjects with female partners of childbearing potential and female subjects of childbearing potential must, therefore, be willing to use adequate contraception as approved by the investigator, such as a two-barrier method or one-barrier method with spermicidal or intrauterine device. This requirement begins 2 weeks before receiving the first trial treatment and ends 6 months after receiving the last treatment;<br>• Signed informed consent obtained before any study specific procedure.
**Exclusion Criteria**	• Active uncontrolled infections or active disseminated intravascular coagulation;<br>• Past or current history of malignancies other than colorectal carcinoma, except for curatively treated basal and squamous cell carcinoma of the skin cancer or in situ carcinoma of the cervix;<br>• Fertile women (< 12 months after last menstruation) and men of childbearing potential not willing to use effective means of contraception;<br>• Women who are pregnant or are breastfeeding;<br>• Previous grade 3/4 infusion related reaction to cetuximab.
**Main parameters of activity**	• Response rate, evaluated according to RECIST 1.1 criteria
**Main parameters of efficacy**	• Progression-free survival (PFS) will be measured from the start of therapy until the first observation of disease progression or death due to any cause<br>The determination of disease progression will be based on investigator-reported measurements. Disease status will be evaluated according to RECIST 1.1 criteria<br>• Overall survival (OS) will be measured from the start of therapy until death due to any cause
**Main parameters of safety**	• Adverse events, laboratory parameters.<br>• All toxicity will be graded using the NCI Common Toxicity Criteria CTCAE version 4.03
**Study Treatment**	*Experimental Treatment*: cetuximab plus irinotecan<br>• Cetuximab 500 mg/sqm iv over 1-h every 2 weeks<br>*followed by*<br>• Irinotecan 180 mg/sqm iv over 1-h every 2 weeks (or according to investigator's choice in the best interest of the patient at previously maximum tolerated dose, but no <130 mg/mq)

**Study Treatment (Cont.)**	Treatment will be continued until:<br>• Disease progression<br>• Death<br>• Unacceptable toxicity*<br>• Consent withdrawn<br>*if irinotecan is stopped due to toxicity, cetuximab may be continued as monotherapy at physician's discretion<br>*if cetuximab will be stopped, irinotecan may be continued as monotherapy at physician's discretion
**Study procedures**	*At Baseline*<br>• Complete medical history, ECOG PS, physical examination and vital signs<br>• Complete blood chemistry and complete blood count and differential<br>• Chest and Abdominal computed tomography (CT) scan<br>• Written informed consent<br>• Collection of formalin-fixed paraffin-embedded tumour blocks (or 10 slides of conventional thickness and polarity for IHC and 10 slides of 10 micron thickness for molecular biology) of primary and/or metastatic sites<br>• Before the administration of the 1st cycle: collection of whole blood and plasma samples for pharmacogenetic, pharmacodynamic and circulating DNA analyses. Whole blood and plasma samples will be immediately maintained at 0-4°C (not more than 45 min) and then stored at -20°C and -80°C, respectively.<br><br>*During Treatment - Every 2 weeks*<br>• Before the administration of the 2nd cycle (i.e. cycle 1 day 14): collection of plasma samples for pharmacodynamic analyses. Plasma samples will be immediately maintained at 0-4°C (not more than 45 min) and then stored at -80°C<br>• Partial Blood chemistry (i.e. total bilirubin, AST, ALT, alkaline phosphatase, creatinine) and complete blood count and differential<br>• Toxicity evaluation (CTCAE v4.03)<br>ECOG PS, physical examination (including weight).<br><br>*During and After Treatment Until Disease Progression - Every 8 weeks*<br>• At first CT-scan evaluation and at progression: collection of plasma samples for pharmacodynamic and circulating DNA analyses. The obtained plasma samples will be immediately maintained at 0-4°C (not more than 45 min) and then stored at -80°C<br>• Tumor evaluation (RECIST criteria v1.1)<br>• Complete blood chemistry, CEA, Ca19.9
**Statistical Considerations**	According to the Fleming single-stage design and selecting the design parameters p0 (RR in the null hypothesis) = 0.05, and p1 (RR in the alternative hypothesis) = 0.20, and considering alpha (one-sided) and beta errors of 0.05 and 0.20 respectively, a total of 27 patients will be required.<br>Null hypothesis will be rejected if at least 4 patients will have an objective response.
**Total Number of Centers**	14 Italian Centers
**Duration of the Study**	Planned accrual time is 18 months for total study duration of 24 months

**Enrollment and Data Management**	Patients registration and data collection are centralized at Polo Oncologico Area Vasta Nord-Ovest – Azienda Ospedaliero-Universitaria Pisana (AOUP), Istituto Toscano Tumori (ITT)
**Contacts Scientific Issues**	Dr. Fotios Loupakis; Prof. Daniele Santini; Dr. Lisa Salvatore
**Contact Administrative Issues**	Dr. Laura Delliponti

FLOW CHART			
	Baseline	***Every 2 weeks***	***Every 8 weeks***
**Informed Consent**	X		
**Demographics and Medical History**	X		
**General Physical Examination**	X		X
**Vital Signs and Physical Measurements**<br>Height, body weight, temperature, blood pressure, pulse, PS	X	X	X
**Blood pregnancy test** (if applicable)	X		
**Hematology**<br>Hemoglobin, platelet count, RBC, WBC including differential	X	X	X
**Blood Chemistry**<br>Creatinine, alkaline phosphatase, ALT, AST, total bilirubin		X	
**Complete Blood Chemistry and Markers**<br>Creatinine, alkaline phosphatase, ALT, AST, GGT, LDH, total bilirubin, total proteins, albumin, Na^+, K^+, Ca^{++}, Mg^{++}, aPTT, INR, CEA, Ca19.9	X		X
**Tumoral Samples Collection**	X		
**Whole Blood and Plasma Sampling**	X	X<br>(2nd cycle)	X<br>(1st CT-scan and PD)
**Toxicities Evaluation**<br>According to NCI CTCAEv4.03	X	X	X
**Tumor Measurements**<br>According to RECIST Criteria 1.1	X		X

DOSE MODIFICATIONS FOR TOXICITIES ATTRIBUTABLE TO IRINOTECAN		
EVENT AT THE START OF SUBSEQUENT CYCLES OF THERAPY	***GRADE***	***ADJUSTMENT***
WBC	<3.000/mm^3	Hold until resolution
Neutrophils	<1.000/mm^3	
Platelets	<100.000/mm^3	
Diarrhea	≥1	
Mucositis	≥1	
Any other non-hematological toxicity	≥2	
**PREVIOUS EVENT**	**GRADE**	***ADJUSTMENT***
Neutropenia >5 days	4	**75%**
Febrile Neutropenia	4	
Thrombocytopenia	≥3	
Diarrhea	3	**75%**
Diarrhea	4	**50%**

DOSE MODIFICATIONS FOR TOXICITIES ATTRIBUTABLE TO CETUXIMAB		
EVENT	***GRADE***	***ADJUSTMENT***
Skin or nail toxicity – *First Occurrence*	3 or 4	Hold cetuximab until grade ≤ 2 and restart at 100% dose level
Skin or nail toxicity in patients treated at 100% or 80% dose level – *Recurring*	3 or 4	Restart cetuximab at 80% dose level or 60% dose level respectively
Symptomatic hypomagnesemia – *First Occurence*		Hold cetuximab until resolution and restart at 100% dose level<br>Mg^{++} supplementation
Symptomatic hypomagnesemia in patients treated at 100% or 80% dose level – *Recurring*		Restart cetuximab at 80% dose level or 60% dose level respectively<br>Mg^{++} supplementation
Diarrhea – *First Occurence*	3 or 4	Hold cetuximab until resolution and restart at 100% dose level
Diarrhea in patients treated at 100% or 80% dose level – *Recurring*	3 or 4	Restart cetuximab at 80% dose level or 60% dose level respectively
Any non-hematologic toxicity	4	Hold cetuximab until resolution

APPENDIX II: SINOSSI (ITALIAN VERSION)

STUDIO DI FASE II, A SINGOLO BRACCIO, DI TERAPIA DI III LINEA CON RECHALLENGE DI CETUXIMAB ED IRINOTECANO IN PAZIENTI CON CARCINOMA COLORETTALE METASTATICO *KRAS*, *NRAS* E *BRAF* WILD-TYPE E IRINOTECANO-PRETRATTATI PROGREDITI, DOPO AVER OTTENUTO UN' INIZIALE RISPOSTA, AD UNA TERAPIA DI PRIMA LINEA CONTENENTE CETUXIMAB

(CRICKET) - EUDRACT 2014-001126-15

**Versione**	2.0 – August 2014
**Sponsor**	Gruppo Oncologico Nord-Ovest (G.O.N.O.)
**Setting**	Terza linea
**Fase**	Fase II a singolo braccio
**Indicazioni**	Pazienti con tumore del colon-retto metastatico non resecabile, *KRAS, NRAS* e *BRAF* wild-type e irinotecano-pretrattati, candidati ad una terza linea di trattamento e progrediti ad una prima linea contenente cetuximab.
**Razionale**	▪ Diversi studi di fase II e III hanno dimostrato che la combinazione di cetuximab con una chemioterapia di prima linea migliora il tasso di risposte e la sopravvivenza libera da progressione nei pazienti con tumore del colon-retto metastatico *KRAS* wild-type.<br>▪ Cetuximab in combinazione con irinotecano è approvato per il trattamento dei pazienti con tumore del colon-retto metastatico *RAS* wild-type e irinotecano-refrattari.<br>▪ Un recente studio di fase II ha dimostrato il potenziale beneficio dal rechallenge con cetuximab in pazienti con tumore del colon-retto metastatico irinotecano-refrattari *KRAS* wild-type, già progrediti ad una precedente terapia contenente cetuximab.<br>▪ Una recente analisi retrospettiva ha dimostarto il ruolo delle mutazioni di *RAS* nel predire la resistenza agli anti-EGFR. Per quanto riguarda *BRAF* invece, mentre il suo valore prognostico negativo è indiscusso, il suo ruolo predittivo resta poco chiaro.
**Endpoints**	**Primario:**<br>• Valutare l'attività in termini di tasso di risposta (RR)<br>**Secondari:**<br>• Sopravvivenza libera da progressione (PFS)<br>• Sopravvivenza globale (OS)<br>• Profilo di tossicità<br>• Valutazione di potenziali biomarcatori predittivi e/o prognostici
**Criteri di Inclusione**	• Diagnosi istologica di adenocarcinoma del colon-retto;<br>• *RAS* e *BRAF* wild-type;<br>• Risposta ad una prima linea di terapia a base di irinotecan (FOLFIRI o FOLFOXIRI) e contenente cetuximab;<br>• PFS alla prima linea di terapia contenente cetuximab ≥6 mesi;<br>• Documentata progressione alla prima linea di terapia entro 4 settimane dall'ultima somministrazione di cetuximab;<br>• Tempo tra la fine della terapia di prima linea e l'inizio della terza linea con cetuximab e irinotecano ≥4 mesi;

**Criteri di Inclusione (Cont.)**	• Seconda linea di trattamento a base di oxaliplatino (FOLFOXIRI, FOLFOX, XELOX) in associazione a bevacizumab;<br>• Documentata progressione alla seconda linea;<br>• Malattia misurabile secondo i criteri RECIST v1.1Avere tessuto tumorale (di tumore primario e metastasi o almeno uno dei due) disponibile per l'analisi dei biomarcatori;<br>• Uomo o donna di età > 18 anni;<br>• ECOG Performance Status ≤ 2;<br>• Aspettativa di vita di almeno 3 mesi;<br>• Adeguata funzionalità ematologica, epatica e renale valutata entro 14 giorni dall'inizio del trattamento in studio;<br>• Donne fertili devono avere test di gravidanza negativo alla visita basale. Per questo studio sono considerate fertili tutte le donne dopo la pubertà, eccetto quelle che sono in menopausa da almeno 12 mesi, quelle chirurgicamente sterili o sessualmente inattive;<br>• I pazienti e i loro partner devono evitare la gravidanza durante il trattamento e fino a 6 mesi dopo l'ultima somministrazione. I soggetti maschili con partner fertili e i soggetti femminili fertili devono quindi accettare l'uso di un adeguato metodo contraccettivo approvato dallo sperimentatore (es. doppia barriera o singola barriera con spermicida o dispositivo intrauterino). La contraccezione è richiesta 2 settimane prima dell'inizio del trattamento e fino a sei medi dopo l'ultima somministrazione;<br>• Consenso informato scritto.
**Criteri di Esclusione**	• Infezioni in atto non controllate o coagulazione intravascolare disseminata in atto;<br>• Storia passata o corrente di altri tumori oltre a quello del colon-retto, con l'eccezione di basaliomi o carcinomi squamocellulari della cute o carcinoma in situ della cervice trattati in modo curativo;<br>• Donna (<12 mesi dall'ultima mestruazione) e uomo in età fertile che non utilizzano adeguati metodi contraccettivi;<br>• Donne in gravidanza o allattamento;<br>• Precedente reazione allergica di grado 3/4 correlata all'infusione di cetuximab.
**Principali parametri di attività**	• Tasso di risposta, valutato secondo i criteri RECIST 1.1
**Principali parametri di efficacia**	• Sopravvivenza libera da progressione (PFS): verrà misurata dall'inizio della terapia fino a progressione di malattia o morte dovuta a qualsiasi causa.<br>La valutazione della progressione di malattia si baserà sulle misurazioni riportate dagli investigatori sulla base dei criteri RECIST 1.1<br>• Sopravvivenza globale (OS): verrà misurata dall'inizio della terapia fino a morte dovuta a qualsiasi causa
**Principali parametri di safety**	• Eventi avversi, parametri di laboratorio.<br>• Tutte le tossicità verranno graduate secondo l' NCI Common Toxicity Criteria CTCAE versione 4.03.
**Trattamento**	*Trattamento sperimentale*: Cetuximab più irinotecano<br>• Cetuximab 500 mg/mq ev in 1-h ogni 2 settimane<br>*seguito da*<br>• Irinotecano 180 mg/mq ev in 1-h ogni 2 settimane (o in accordo alla scelta dell'investigatore nel miglior interesse del paziente alla precedente massima dose tollerata, ma <130 mg/mq)<br>Il trattamento verrà continuato fino a:<br>• Progressione di malattia

**Trattamento (Cont)**	• Morte<br>• Tossicità inaccettabile*<br>• Ritiro del consenso<br>*Se irinotecano viene sospeso per tossicità, cetuximab può essere continuato in monoterapia a discrezione dell'investigatore;<br>Se cetuximab viene sospeso per tossicità, irinotecano può essere continuato in monoterapia a discrezione dell'investigatore.
**Procedure dello studio**	*Basale*<br>• Anamnesi, ECOG PS, esame obiettivo e segni vitali<br>• Emocromo e chimica completa<br>• TC torace/addome<br>• Consenso informato scritto<br>• Raccolta di blocchetto in paraffina (o 10 vetrini con spessore e polarità convenzionali per IHC e 10 vetrini da 10 micron per biologia molecolare) del tumore primitivo e/o delle sedi metastatiche<br>• Prima della somministrazione del primo ciclo: raccolta di campioni di sangue intero e di plasma per le analisi di farmacogenetica, farmacodinamica e DNA circolante. I campioni di sangue intero e di plasma devono essere immediatamente mantenuti a 0-4°C (per non più di 45 minuti) e poi conservati a -20°C e -80°C, rispettivamente<br>*Durante il trattamento – Ogni 2 settimane:*<br>• Prima della somministrazione del secondo ciclo (i.e. ciclo 1 giorno 14): raccolta di campioni di plasma per le analisi di farmacodinamica. I campioni di plasma devono essere immediatamente mantenuti a 0-4°C (per non più di 45 minuti) e poi conservati a -80°C<br>• Chimica parziale (bilirubina totale, AST, ALT, fosfatasi alcalina, creatinina) ed emocromo<br>• Valutazione delle tossicità<br>• ECOG PS, esame obiettivo (incluso il peso)<br>*Durante e dopo il trattamento fino a progressione – Ogni 8 settimane:*<br>• Alla prima valutazione TC e alla progressione: raccolta di campioni di plasma per le analisi di farmacodinamica e DNA circolante. I campioni di plasma devono essere immediatamente mantenuti a 0-4°C (per non più di 45 minuti) e poi conservati a -80°C<br>• Valutazione di malattia (RECIST v1.1)<br>• Chimica completa, CEA, Ca19.9
**Considerazioni statistiche**	In accordo al disegno di Fleming single-stage, selezionando p0 (RR nell'ipotesi nulla) = 0.05, and p1 (RR nell'ipotesi alternativa) = 0.20, e considerando l'errore alpha (a 1 coda) e quello beta uguali a 0.05 e 0.20 rispettivamente, saranno necessari 27 pazienti.<br>L'ipotesi nulla verrà rifiutata se almeno 4 pazienti avranno una risposta obiettiva.
**Numero centri**	14 centri Italiani
**Durata dello studio**	Accrual pianificato: 18 mesi; durata totale dello studio: 24 mesi
**Arruolamento e gestione dati**	La registrazione dei pazienti e la raccolta dei dati sono centralizzate presso il Polo Oncologico Area Vasta Nord-Ovest – Azienda Ospedaliero-Universitaria Pisana (AOUP), Istituto Toscano Tumori (ITT)
**Contatti medici**	Dr. Fotios Loupakis; Prof. Daniele Santini; Dr.ssa Lisa Salvatore
**Contatti amminis.**	Dr.ssa Laura Delliponti

FLOW CHART			
	Basale	***Ogni 2 settimane***	***Ogni 8 settimane***
**Consenso informato**	X		
**Anamnesi**	X		
**Esame obiettivo**	X		X
**Segni vitali e parametri fisici**<br>Altezza, peso, temperatura, pressione, polso, PS	X	X	X
**test gravidanza su sangue** (se applicabile)	X		
**Emocromo**<br>Emoglobina, piastrine, eritrociti, globuli bianchi e formula	X	X	X
**Chimica**<br>Creatinina, fosfatasi alcalina, ALT, AST, bilirubina totale		X	
**Chimica completa e Markers**<br>Creatinina, fosfatasi alcalina, ALT, AST, GGT, LDH, bilirubina totale, proteine, albumin, Na^{+}, K^{+}, Ca^{++}, Mg^{++}, aPTT, INR, CEA, Ca19.9	X		X
**Raccolta campioni tumorali**	X		
**Raccolta sangue intero e plasma**	X	X<br>(ciclo 2)	X<br>(prima TC e PD)
**Valutazione delle tossicità**<br>Secondo NCI CTCAEv4.0	X	X	X
**Valutazione di malattia**<br>Secondo i criteri RECIST v1.1	X		X

MODIFICAZIONI DI DOSE PER TOSSICITA' DOVUTE AD IRINOTECANO		
EVENTO ALL'INIZIO DI OGNI CICLO	***GRADO***	***MODIFICA***
Globuli bianchi	<3.000/mm^3	Sospendere fino a risoluzione
Neutrofili	<1.000/mm^3	
Piastrine	<100.000/mm^3	
Diarrea	≥1	
Mucosite	≥1	
Altre tossicità non ematologiche	≥2	
EVENTI PRECEDENTI	***GRADO***	***MODIFICA***
Neutropenia >5 giorni	4	**75%**
Neutropenia febbrile	4	
Piastrinopenia	≥3	
Diarrea	3	**75%**
Diarrea	4	**50%**

MODIFICAZIONI DI DOSE PER TOSSICITA' DOVUTE A CETUXIMAB		
EVENTO	***GRADO***	***MODIFICA***
Tossicità cutanea o ungueale – *Prima occorrenza*	3 o 4	Sospendere cetuximab fino a grado ≤ 2 e riprendere al 100% della dose
Tossicità cutanea o ungueale in pazienti trattati al 100% o 80% della dose – *Ricorrenza*	3 o 4	Riprendere cetuximab all' 80% o al 60% della dose rispettivamente
Ipomagnesemia sintomatica – *Prima occorrenza*		Sospendere cetuximab fino a risoluzione e riprendere al 100% della dose<br>Supplementazione di Mg^{++}
Ipomagnesemia sintomatica in pazienti trattati al 100% o 80% della dose – *Ricorrenza*		Riprendere cetuximab all' 80% o al 60% della dose rispettivamente<br>Supplementazione di Mg^{++}
Diarrea – *Prima occorrenza*	3 o 4	Sospendere cetuximab fino a risoluzione e riprendere al 100% della dose
Diarrea in pazienti trattati al 100% o 80% della dose – *Ricorrenza*	3 o 4	Riprendere cetuximab all' 80% o al 60% della dose rispettivamente
Qualsiasi tossicità non ematologica	4	Sospendere cetuximab fino a risoluzione

APPENDIX III: Dichiarazione di Helsinki della Associazione Medica Mondiale (AMM)

(Traduzione di Antonio G. Spagnolo)

PRINCIPI ETICI PER LA RICERCA MEDICA CHE COINVOLGE SOGGETTI UMANI

Adottata dalla 18° Assemblea Generale dall'AMM a Helsinki, Finlandia, nel giugno 1964 ed emendata dalla 29° Assemblea Generale a Tokyo, Giappone, nell'ottobre 1975, dalla 35° Assemblea Generale a Venezia. Italia, nell'ottobre 1983, dalla - 41ª Assemblea Generale a Hong Kong, nel settembre 1989, dalla 48° Assemblea Generale a Somerset West, Repubblica del Sud Africa, nell'ottobre 1996 e dalla 52° Assemblea Generale a Edimburgo, Scozia, nell'ottobre 2000.

a. Introduzione

1. L'AMM ha elaborato la Dichiarazione di Helsinki come dichiarazione di principi etici che forniscano una guida per i medici e per gli altri partecipanti ad una ricerca medica che coinvolge soggetti umani. La ricerca medica che coinvolge soggetti umani include la ricerca su materiale umano identificabile o su altri dati identificabili.
2. È dovere del medico promuovere e salvaguardare la salute delle persone. Le sue conoscenze e la sua coscienza sono finalizzate al compimento di questo dovere.
3. La Dichiarazione di Ginevra[*] dell'AMM impegna il medico con le parole «La salute del mio paziente sarà la mia preoccupazione principale», e il Codice Internazionale di Etica Medica[**] dichiara che «Un medico dovrà agire solo nell'interesse del paziente quando fornisca una cura medica che possa avere l'effetto di indebolire lo stato fisico e mentale del paziente».
4. Il progresso medico è fondato sulla ricerca la quale a sua volta si deve basare in qualche misura su una sperimentazione che coinvolga soggetti umani.
5. Nella ricerca su soggetti umani, le considerazioni correlate con il benessere del soggetto umano devono avere la precedenza sugli interessi della scienza e della società.
6. Lo scopo primario della ricerca medica che coinvolga soggetti umani è quello di migliorare le procedure preventive, diagnostiche e terapeutiche e di comprendere l'eziologia e la patogenesi della malattia. Anche i più comprovati metodi preventivi, diagnostici e terapeutici devono continuamente essere messi in discussione mediante la ricerca sulla loro efficacia, efficienza, accessibilità e qualità.
7. Nella pratica medica corrente e nella ricerca medica, la maggior parte delle procedure preventive, diagnostiche e terapeutiche implicano rischi ed aggravi.
8. La ricerca medica è sottoposta agli standard etici che promuovono il rispetto per tutti gli esseri umani e proteggono la loro salute e i loro diritti. Alcuni soggetti di ricerca sono vulnerabili e richiedono una speciale protezione. Devono essere riconosciuti le particolari necessità di coloro che sono economicamente e medicalmente svantaggiati. Una speciale attenzione e

[*] *Approvata a Ginevra nel 1948 e rivista a Sidney nel 1968 dalla XXII Assemblea Medica Mondiale (N.d.T.)*

[**] *Adottato a Londra dalla III Assemblea Generale dell'Associazione Medica Mondiale (N.d.T.)*

pure richiesta per coloro che non possono dare o che rifiutano il consenso personale, per coloro che possono essere esposti a dare il consenso sotto costrizione, per coloro che non beneficeranno personalmente dalla ricerca e per coloro per i quali la ricerca è associata alla cura.

9. I ricercatori devono essere al corrente dei requisiti etici, giuridici e regolatori della ricerca sui soggetti umani, sia i requisiti nazionali sia quelli internazionali, ove applicabili. Nessun requisito nazionale di natura etica, giuridica o regolatoria deve poter ridurre o eliminare alcuna delle protezioni per i soggetti umani esposte in questa Dichiarazione.

b. Principi basilari per tutta la ricerca medica

10. Nella ricerca medica è dovere del medico proteggere la vita, la salute, la riservatezza e la dignità del soggetto umano.

11. La ricerca medica che coinvolge soggetti umani deve essere conforme ai principi scientifici universalmente accettati e deve essere basata su una approfondita conoscenza della letteratura scientifica, di altre rilevanti fonti di informazione, e su un'adeguata sperimentazione in laboratorio e, ove appropriato, sull'animale.

12. Un'appropriata cautela deve essere posta nella conduzione di ricerche che possano incidere sull'ambiente, e deve essere rispettato il benessere degli animali utilizzati per la ricerca.

13. Il disegno e l'esecuzione di ogni procedura sperimentale che coinvolga soggetti umani devono essere chiaramente descritti in un protocollo di sperimentazione. Tale protocollo deve essere sottoposto ad esame, commenti, orientamenti e, dove previsto, all'approvazione da parte di un comitato etico di revisione appositamente istituito; che deve essere indipendente dal ricercatore, dallo sponsor e da qualsiasi altro tipo di indebita influenza. Questo comitato indipendente deve essere conforme alle leggi ed ai regolamenti della nazione in cui la sperimentazione è condotta. Il comitato ha titolo per monitorare i trial in corso. Il ricercatore ha l'obbligo di fornire le informazioni di monitoraggio al comitato, specialmente quelle relative agli eventi avversi seri. Il ricercatore deve anche sottoporre al comitato, per la revisione, le informazioni relative a finanziamento, sponsor, appartenenze a istituzione, altri potenziali conflitti di interesse e incentivi per i soggetti di sperimentazione.

14. Il protocollo di ricerca deve sempre contenere una esposizione delle considerazioni etiche implicate e deve recare l'indicazione di conformità con i principi, enunciati nella presente Dichiarazione.

15. La ricerca biomedica che coinvolge soggetti umani deve essere condotta solo da persone scientificamente qualificate e sotto la supervisione di un medico competente sul piano clinico. La responsabilità nei confronti del soggetto umano deve sempre ricadere sul personale medico qualificato e mai sul soggetto della ricerca, anche se questi ha dato il proprio consenso

16. Ogni progetto di ricerca medica che coinvolga soggetti umani deve essere preceduto dà un'attenta valutazione dei rischi e degli aggravi prevedibili in rapporto ai benefici attesi per il soggetto stesso o per altri. Ciò non preclude la partecipazione di volontarisani ad una ricerca medica. Il disegno di tutti gli studi deve essere pubblicamente disponibile.

17. I medici devono astenersi dall'intraprendere progetti di ricerca che coinvolgano soggetti umani a meno che non siano sicuri che i rischi implicati siano stati adeguatamente valutati e possano essere controllati in modo

soddisfacente. 1 medici devono interrompere ogni ricerca se i rischi si presentano superiori ai potenziali benefici o se si è raggiunta già una prova definitiva di risultati positivi e benefici.

18. La ricerca medica che coinvolga soggetti umani deve essere condotta solo se l'importanza dell'obiettivo prevalga sui i rischi e gli aggravi connessi per il soggetto. Ciò è particolarmente importante quando i soggetti umani siano volontari sani.

19. La ricerca medica è giustificata solo se vi è una ragionevole probabilità che le popolazioni in cui la ricerca è condotta possano beneficiare dei risultati della ricerca.

20. I soggetti devono essere volontari e partecipare informati al progetto di ricerca.

21. Il diritto dei soggetti di sperimentazione alla salvaguardia della loro integrità deve essere sempre rispettato. Deve essere adottata ogni precauzione per rispettare la privacy del soggetto, la riservatezza sulle informazioni relative al paziente e per minimizzare l'impatto dello studio sulla integrità fisica e mentale del soggetto e sulla sua personalità.

22. In ogni ricerca su esseri umani ciascun potenziale soggetto deve essere adeguatamente informato degli scopi, dei metodi, delle fonti di finanziamento, di ogni possibile conflitto di interessi, della appartenenza istituzionale del ricercatore, dei benefici previsti e dei rischi potenziali connessi allo studio, nonché dei fastidi che esso potrebbe comportare. Il soggetto deve essere informato del diritto di astenersi dal partecipare allo studio o della possibilità di ritirare il consenso alla partecipazione in qualsiasi momento senza ritorsioni. Solo dopo essersi assicurato che il soggetto abbia compreso le informazioni, il medico deve ottenere dal soggetto il consenso informato, liberamente espresso, pre feribilmente in forma scritta. Se il consenso non può essere ottenuto per iscritto, deve essere formalmente documentato e testimoniato un consenso non scritto.

23. Nell'ottenere il consenso informato al progetto di ricerca, il medico deve essere particolarmente attento quando il soggetto si trovi in una condizione di dipendenza nei suoi confronti o possa sentirsi costretto a dare il consenso. In questo caso il consenso informato deve essere ottenuto da un altro medico che conosca bene la ricerca ma non sia coinvolto in essa e che sia completamente indipendente nella relazione col soggetto.,

24. Per un soggetto di ricerca che sia legalmente, fisicamente o mentalmente incapace di dare il consenso, o per un minore legalmente incapace, il ricercatore deve ottenere il consenso informato dal tutore legale, in accordo con la legislazione specifica. Questi gruppi di soggetti non devono essere inclusi in una ricerca a meno che la ricerca stessa non sia necessaria per promuovere la salute della popolazione rappresentata e tale ricerca non possa essere invece attuata su persone legalmente capaci.

25. Quando un soggetto giudicato legalmente incapace, come un minore, sia capace di dare un assenso alla decisione di partecipare in una ricerca, lo sperimentatore deve ottenere tale assenso in aggiunta a quello del tutore legale.

26. La ricerca su individui dai quali non sia possibile ottenere un consenso, incluso quello rappresentato o anticipato, deve essere attuata solo se la condizione fisica o mentale che impedisce di ottenere il consenso è una caratteristica necessaria della popolazione in studio. Le ragioni specifiche per coinvolgere soggetti di ricerca che si trovino in condizioni tali da renderli incapaci di dare un consenso informato devono essere dichiarate nel

protocollo di sperimentazione per l'esame e l'approvazione da parte del comitato di revisione. Il protocollo deve dichiarare che il consenso a rimanere nella ricerca sarà ottenuto non appena possibile da parte dello stesso soggetto o da un rappresentante legalmente autorizzato.

27. Sia gli autori sia gli editori hanno obbligazioni etiche. Nella pubblicazione dei risultati della ricerca gli sperimentatori sono obbligati, a salvaguardare l'accuratezza dei risultati. Sia i risultati negativi sia quelli positivi devono essere pubblicati o resi in qualche modo pubblicamente disponibili. Le fonti del si devono essere dichiarati nella pubblicazione. Relazioni di sperimentazioni non conformi con i principi fissati in questa Dichiarazione non devono essere accettati per la pubblicazione.

c. Principi aggiuntivi per la ricerca medica associata alle cure mediche

28. Il medico può associare la ricerca medica con le cure mediche solo con il limite che la ricerca sia giustificata da un potenziale valore preventivo, diagnostico o terapeutico. Quando la ricerca medica è associata con le cure mediche si applicano degli standard addizionali per proteggere i pazienti che sono soggetti di ricerca.

29. I benefici, i rischi, gli aggravi e l'efficacia di un nuovo metodo devono essere valutati in confronto con quelli dei migliori metodi preventivi, diagnostici e terapeutici attualmente in uso. Ciò non esclude l'impiego di placebo, o l'assenza di trattamento, negli studi dove non esistono metodi comprovati di prevenzione, diagnosi o terapia.

30. A conclusione _dello studio, ad ogni paziente entrato nello studio deve essere assicurato l'accesso ai migliori metodi preventivi, diagnostici e terapeutici di comprovata efficacia identificati dallo studio.

31. Il medico deve informare pienamente il paziente di quali aspetti della cura sono correlati con la ricerca. Il rifiuto di un paziente a partecipare in uno studio non deve mai interferire con la relazione medico-paziente.

32. Nel trattamento di un paziente, laddove non esistano comprovati metodi preventivi, diagnostici e terapeutici o questi siano stati inefficaci, il medico, con il consenso informato del paziente, deve essere libero di usare mezzi preventivi, diagnostici e terapeutici non provati o nuovi, se a giudizio del medico essi offrono speranza di salvare la vita, ristabilire la salute o alleviare la sofferenza. Laddove possibile, tali mezzi dovrebbero essere fatti oggetto di una ricerca disegnata per valutare la loro sicurezza ed efficacia. In tutti i casi, le nuove informazioni devono essere registrate e, dove opportuno, pubblicate. Tutte le altre linee-guida di questa Dichiarazione devono essere seguite.

APPENDIX IV: SCALA PER LA VALUTAZIONE DEL PERFORMANCE STATUS

Scale di valutazione delle condizioni generali (performance status) (Karnofsky ed ECOG)

ECOG	Karnofsky
**0** In grado di svolgere le attività normali senza restrizioni	**100%** Normale nessun disturbo né evidenza di malattia
	**90%** In grado di svolgere le attività normali; modesti segni o sintomi di malattia
**1** Presenta restrizioni alle attività fisiche strenue, ma deambula ed è in grado di svolgere attività lievi o sedentarie,quali lavori domestici	**80%** Attività normale con sforzo; alcuni segni o sintomi di malattia
**2** Deambula, è autosufficiente, ma non può svolgere attività lavorative; in piedi per più del 50% del tempo	**60%** Richiede assistenza saltuaria, ma può soddisfare la maggior parte delle sue esigenze
	**50%** Richiede notevole assistenza e frequenti cure mediche
**3** Appena autosufficiente, allettato o seduto per più del 50% del tempo	**40%** Disabile; richiede particolari cure e assistenza
	**30%** Gravemente disabile, sono opportuni il ricovero ospedaliero e un trattamento di sostegno efficace; il decesso non è imminente
**4** Completamento disabile; non autosufficiente; sempre allettato o seduto	**20%** molto ammalato; sono necessari il ricovero ospedaliero e un trattamento di sostegno efficace
	**10%** Moribondo, i processi fatali progrediscono rapidamente
**5** Deceduto	**0%** Deceduto

Karnofsky et al. The use of the nitrogen mustards in the palliation treatment of carcinoma with particular reference to bronchogenic carcinoma, Cancer 1:634-656, 1948, e di Oken MM et al: Toxicity and response criteria of the Eastern Cooperative Oncology Group, Am J Clin Oncol 5:649-655, 1982.

APPENDIX V: VALUTAZIONE RISCHIO/BENEFICIO

(STUDIO CRICKET) - EUDRACT 2014-001126-15

Versione 1.6 Aprile 2014

**STUDIO DI FASE II, A SINGOLO BRACCIO, DI TERAPIA DI III LINEA CON RECHALLENGE DI CETUXIMAB ED IRINOTECANO IN PAZIENTI CON CARCINOMA COLORETTALE METASTATICO KRAS, NRAS E BRAF WILD-TYPE E IRINOTECANO-PRETRATTATI PROGREDITI, DOPO AVER OTTENUTO UN' INIZIALE RISPOSTA, AD UNA TERAPIA DI PRIMA LINEA CONTENENTE CETUXIMAB.**

Il presente studio ha come obiettivo quello di valutare un ritrattamento con cetuximab e irinotecano in pazienti con tumore del colon-retto metastatico *KRAS, NRAS* e *BRAF* wild-type, irinotecano resistenti, progrediti ad una prima linea a base di irinotecano (FOLFIRI o FOLFOXIRI) contenente cetuximab dalla quale abbiano ricevuto un iniziale beneficio. Diversi studi hanno dimostrato che l'utilizzo di cetuximab in monoterapia o in combinazione con irinotecano migliora l'outcome di pazienti con tumore del colon-retto metastatico *KRAS* wild-type in cui gli altri trattamenti chemioterapici hanno fallito e che non hanno mai ricevuto un trattamento con anticorpi diretti contro il recettore per il fattore di crescita epidermico (EGFR). Un recente studio di fase II ha dimostrato un potenziale beneficio derivante da un ritrattamento con cetuximab in pazienti irinotecano-pretrattati che hanno già ricevuto una terapia contenente cetuximab. Inoltre recenti analisi hanno dimostrato che anche le mutazioni di *NRAS* hanno un ruolo nel predire la resistenza agli anticorpi anti-EGFR. Per quanto riguarda *BRAF*, benchè il suo ruolo predittivo non sia stato ancora definitivamente chiarito, è possibile affermare che la presenza di tale mutazione sia associata a ben poche possibilità di beneficiare di un trattamento con cetuximab o panitumumab, mentre il suo valore prognostico negativo resta indiscusso.

Dal punto di vista della safety, l'associazione di cetuximab e irinotecano presenta un profilo di tossicità maneggevole ed è ormai utilizzata routinariamente nella pratica clinica.

Questo studio di fase II ha lo scopo di gettare le basi per valutazioni future sul rechallenge di cetuximab e irinotecano in pazienti con tumore del colon-retto metastatico *KRAS, NRAS* e *BRAF* wild-type, che hanno beneficiato di un trattamento di prima linea a base di irinotecano contenente cetuximab.

Di seguito i riferimenti scientifici:

- Cunningham D, et al. NEJM 2004;351:337-45
- Karapetis CS, et al. NEJM 2008;359:1757-65
- Jonker DJ, et al. NEJM 2007;357:2040-8
- Wilke H, et al. JCO 2008;33:5335-43
- Santini D, et al. Ann Oncol 2012;23:2313-8
- Douillard JY, et al. NEJM 2013; 369: 1023-1034.
- Stintzing S, et al. EJC 2013; ESMO-ECCO 2013; LBA 7.

A PHASE II TRIAL OF RE**CH**ALLENGE WITH PANITUMUMAB D**R**IVEN BY RAS CL**ON**AL-MEDIATED DYNAMIC **O**F RE**S**ISTANCE.

THE **CHRONOS** TRIAL

CHRONOS
Franz Ignaz GÜNTHER
1765-70

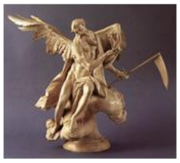

Limewood, white painted
Bayerisches Nationalmuseum
Munich

Study Type:	Phase II
Protocol Number:	013-IRCC-10IIS-16
EudraCT Number:	2016-002597-12
Protocol Version (Date):	v. 3.0 -22.03.2019
Sponsor:	Fondazione del Piemonte per l'Oncologia - Istituto di Candiolo IRCCS

This document contains confidential information belonging to Sponsor. Except as may be otherwise agreed to in writing, by accepting or reviewing these materials, you agree to hold such information in confidence and not to disclose it to others (except where required by applicable law), nor to use it for unauthorized purposes. In the event of actual or suspected breach of this obligation Sponsor should be promptly notified.

Contents

Contents 2
1 SIGNATURE PAGE 9
2 STUDY PERSONNEL 10
3 SYNOPSIS 11
4 SCHEDULE OF EVENTS 17
5 BACKGROUND AND STUDY RATIONALE 18
5.1 Rationale 18
6 OBJECTIVES AND ENDPOINTS OF THE TRIAL 20
6.1 Objectives 20
6.1.1 Primary Objective 20
6.1.2 Secondary Objectives 20
6.1.3 Translational Objectives 20
6.2 End-Points 20
6.2.1 Primary End-point 20
6.2.2 Secondary End-point 21
6.2.3 Translational End-point 21
7 TRIAL DESIGN 21
7.1 Patient Population 21
7.2 Patients' selection process 22
7.2.1 Screening Phase 22
7.2.2 Trial Phase 23
7.3 Enrolment 23
7.4 Study logistical plan 23
8 STUDY POPULATION 24
8.1 Inclusion Criteria 24
8.2 Exclusion Criteria 25
8.3 Patients substitution criteria 26
8.3.1 Severe Infusion reaction (Trial Phase only) 26
9 TREATMENT (TRIAL PHASE ONLY) 26
9.1 Description of investigational product 26
9.2 Pregnancy and contraception 28
9.3 Treatment Plan 29

9.3.1 Drug Administration ... 29
9.3.2 Premedication and patient monitoring during infusion ... 30
9.3.3 Duration of Therapy ... 30
9.4 Concomitant Therapy ... 30
9.5 Dose Adjustments ... 31
9.5.1 Panitumumab Dose Reductions ... 31
9.5.2 Criteria for Withholding a Dose of Panitumumab ... 31
9.5.3 Criteria for Re-treatment with Panitumumab ... 32
9.5.4 Dose Modification Schedule for Panitumumab ... 32
9.5.5 Delayed or Missed Doses ... 33
9.5.6 Discontinuation ... 34
9.5.7 Management of dermatologic toxicity and recommended concomitant treatment ... 34
10 CLINICAL EVALUATION, LABORATORY TESTS, FOLLOW-UP ... 34
10.1 Before Treatment Start ... 34
10.2 During Treatment ... 36
10.3 After the End of Treatment (Follow-Up) ... 36
11 EFFICACY ASSESSMENT ... 37
11.1 Definitions ... 37
11.2 Tumour assessments ... 38
11.2.1 Measurability of Tumour Lesions at Baseline ... 38
11.2.2 Tumour Response Evaluation ... 40
11.2.3 Date of Progression ... 42
11.2.4 Reporting of Tumour Response ... 42
11.3 Progression Free Survival ... 43
11.4 Overall Survival ... 43
12 SAFETY ASSESSMENT ... 43
12.1 Definitions for Adverse Event ... 43
12.2 Reporting procedures for Adverse Event ... 44
12.3 Reporting requirements for adverse event ... 45
12.4 Reporting requirements for complaints related to panitumumab ... 47
12.5 Recording Adverse Events in the Case Report Forms ... 47
12.5.1 Pre-existing Conditions ... 47
12.5.2 Procedures ... 48

12.5.3 Symptoms of Targeted Disease ... 48
12.5.4 Causality Assessment and Grading of Adverse Event Severity ... 48
12.5.5 Pregnancy Reporting ... 49
12.5.6 Overdose ... 50
12.5.7 Follow-up of Unresolved Adverse Events ... 50
13 TRANSLATIONAL ASSESSMENT ... 50
14 STATISTICAL CONSIDERATIONS ... 51
14.1 Sample Size ... 51
14.2 Statistical Analysis ... 51
14.2.1 Analysis Populations ... 51
14.2.2 Statistical Methods ... 52
15 QUALITY CONTROL AND QUALITY ASSURANCE ... 54
15.1 Monitoring ... 54
15.2 Auditing ... 54
16 DATA HANDLING AND RECORD KEEPING ... 54
16.1 Case Report Form (CRF) ... 54
16.2 Data Handling ... 55
16.3 Record Retention ... 55
17 ETHICAL CONSIDERATIONS ... 56
17.1 Institutional Review Board (IRB)/ Independent Ethics Committee (IEC) and Competent Authority (CA) ... 56
17.2 Ethical conduct of the trial ... 56
17.3 Informed Consent ... 56
18 LIABILITY AND INSURANCE ... 57
19 CONFIDENTIALITY OF INFORMATION AND PUBLICATION OF RESULTS ... 57
20 FIGURES ... 58
20.1 Figure 1 Mutated KRAS alleles emerge in circulating DNA during anti-EGFR therapy and decline when treatment is suspended ... 58
20.2 Figure 2 Re-challenge with EGFR specific antibodies in CRC cells and patients ... 59
20.3 Figure 3 Study Flow Chart ... 61
21 APPENDIX ... 62
21.1 Appendix A: References ... 62
21.2 Appendix B: minimum set of genes to be tested on tissue FFPE ... 63

21.3 Appendix C: ddPCR Panel for Molecular Screening 64
21.4 Appendix D: Liquid Biopsy Guidelines for Molecular Screening Phase 65
21.5 Appendix E: Liquid Biopsy Guidelines for Trial Phase 65

List of Abbreviations

5-FU	5-fluorouracil
ADR	Adverse Drug Reaction
AE	Adverse Event
ALT	Alanine Aminotransferase (Serum Glutamic-pyruvic Transaminase)
ANC	Absolute Neutrophil Count
aPTT	Activated Partial Thromboplastin Time
AST	Aspartate Aminotransferase (Serum Glutamic-oxaloacetic Transaminase)
AUC	Area Under the Curve
BML	Baseline Mutational Load
CEA	Carcinoembryonic Antigen
CI	Confidence Interval
CL	Clearance
CONSORT	Consolidated Standards of Reporting Trials
CR	Complete Response
CRC	Colorectal Cancer
CRF	Case Report Form
CRO	Clinical Research Organization
CT	Computerized Tomography
CTC	Common Terminology Criteria
CTCAE	Common Terminology Criteria for Adverse Events
Ct-DNA	Circulating tumor DNA
CTM	Clinical Trial Manager
DCL	Data Clarification List
dd-PCR	Digital Droplet PCR
DSUR	Development Safety Update Report
eCRF	Electronic Case Report Form
ECD	Extracellular Ectodomain
ECG	Electrocardiogram
ECOG	Eastern Cooperative Oncology Group
EE	Efficacy Evaluable
EGF	Epidermal Growth Factor
EGFR	Epidermal Growth Factor Receptor
FFPE	Formalin Fixed Paraffin Embedded
FPI	First Patient In
GCP	Good Clinical Practice
G-CSF	Granulocyte Colony Stimulating Factor

HR	Hazard Ratio
IC	Informed Consent
IEC	Independent Ethics Committee
IgG2	Immunoglobulin G2
ICH	International Conference on Harmonization
IML	Intermediate Mutational Load
INR	International Normalized Ratio
IRB	Institutional Review Board
ITT	Intention-to-treat analysis
IV	Intravenous
LDH	Lactate Dehydrogenase
LLN	Lower Limit of Normal
LPI	Last Patient In
LPLV	Last Patient Last Visit
LVEF	Left Ventricular Ejection Fraction
mAb	Monoclonal Antibody
mCRC	Metastatic Colorectal Carcinoma
MRI	Magnetic Resonance Imaging
MS	Molecular Screening
Msec	Milliseconds
MUGA	Multigated Acquisition
NCI	National Cancer Institute
NGS	Next Generation Sequencing
NYHA	New York Heart Association
ORR	Objective Response Rate
OS	Overall Survival
PET	Positron Emission Tomography
PD	Progressive Disease
PFS	Progression-Free Survival
PR	Partial Response
QW, Q2W, Q3W, Q8W, Q9W	Every Week, Every 2 Weeks, Every 3 Weeks, Every 8 Weeks, Every 9 Weeks
RBC	Red Blood Cell
RECIST	Response Evaluation Criteria In Solid Tumors
RML	Rechallenge Mutational Load
ROC	Receiver Operator Characteristic
SADR	Serious Adverse Drug Reaction

SAE	Serious Adverse Event
SAS	Statistical Analysis System
SD	Stable Disease
SE	Safety Evaluable
SPF	Sun Protection Factor
SUSAR	Suspect Unexpected Serious Adverse Reaction
TMF	Trial Master File
ULN	Upper Limit of Normal
WBC	White Blood Count / White Blood Cells
WKS	Weeks
WT	Wild Type

1 SIGNATURE PAGE

Sponsor Signature

Prof. Anna Sapino

Signature 22/03/2019
Date

Clinical Study Chair Signature

Prof. Salvatore Siena

Signature ASST Grande Ospedale Metropolitano Niguarda S.C. ONCOLOGIA FALCK NIGUARDA CANCER CENTER Dr. SALVATORE SIENA 22/03/2019
Date

Translational Study Chair Signature

Prof. Alberto Bardelli

Signature 22/03/2019
Date

Principal Investigator Agreement

I have read the Protocol entitled "A PHASE II TRIAL OF RECHALLENGE WITH PANITUMUMAB DRIVEN BY RAS CLONAL-MEDIATED DYNAMIC OF RESISTANCE" and I agree to conduct the study as detailed herein and in compliance with ICH Guidelines for Good Clinical Practice and applicable regulatory requirements. I will provide all study personnel under my supervision with all information provided by the Sponsor and I will inform them about their responsibilities and obligations.

Principal Investigator
(printed name, Institution, Department and location)

Signature Date

2 STUDY PERSONNEL

STUDY CHAIRS
**OPERATIONAL**<br>**Prof. Anna Sapino**<br>Fondazione del Piemonte per l'Oncologia – Istituto di Candiolo IRCCS<br>Telefono: 011 993 3887; Email: anna.sapino@ircc.it<br><br>**CLINICAL**<br>**Prof. Salvatore Siena**<br>Niguarda Cancer Center, Grande Ospedale Metropolitano – MILANO e Dipartimento di Oncologia ed Onco-Ematologia Università degli Studi di Milano<br>Telefono: 02 6444 2291; Email: salvatore.siena@ospedaleniguarda.it<br><br>**TRANSLATIONAL**<br>**Prof. Alberto Bardelli**<br>Fondazione Piemontese per la Ricerca sul Cancro (FPRC) – CANDIOLO (TO) e Dipartimento di Oncologia Università degli Studi di Torino<br>Telefono; 011 993 3548; Email: alberto.bardelli@ircc.it

PROJECT MANAGER
**Dr. Francesco Rua**<br>Fondazione del Piemonte per l'Oncologia, Istituto di Candiolo IRCCS **–** CANDIOLO (TO)<br>Telefono: 011 993 3203; Email: francesco.rua@ircc.it

PHARMACOVIGILANCE
**Dr.ssa Fiorenza Enrico**<br>Fondazione del Piemonte per l'Oncologia, Istituto di Candiolo IRCCS **–** CANDIOLO (TO)<br>Telefono: 011 993 3261; Email: farmacia@ircc.it

LAB CENTRALIZED LIQUID BIOPSY
**Dr.ssa Giulia Siravegna and Dr.ssa Benedetta Mussolin (Centralized Liquid Biopsy Unit)**<br>Fondazione del Piemonte per l'Oncologia, Istituto di Candiolo IRCCS **–** CANDIOLO (TO)<br>Telefono: 011 993 3812; Email: giulia.siravegna@ircc.it; benedetta.mussolin@ircc.it<br><br>**Dr.ssaTiziana Venesio**, **(Molecular Pathology Unit)**<br>Fondazione del Piemonte per l'Oncologia, Istituto di Candiolo IRCCS **–** CANDIOLO (TO)<br>Telefono: 011 9933547; Email: tiziana.venesio@ircc.it

BIOSTATICIAN
**Dr. Valter Torri**<br>Istituto Mario Negri – MILANO<br>Telefono: 02 390 141; Email: valter.torri@marionegri.it

3 SYNOPSIS

Protocol Number	013-IRCC-10IIS-16
Title	**A PHASE II TRIAL OF RECHALLENGE WITH PANITUMUMAB DRIVEN BY RAS CLONAL-MEDIATED DYNAMIC OF RESISTANCE**
Brief title	The **CHRONOS** Trial
Sponsor	Fondazione del Piemonte per l'Oncologia FPO-IRCCS
Clinical Phase	Phase II
Background and Study Rationale	Colorectal cancer (CRC) is the third most common cancer in the world and the second leading cause of cancer death in the United States and the European Union. In the last decade, substantial advances in the treatment of the metastatic disease (mCRC) have more than doubled overall survival (OS) from 12 months to 30 months due to the refinement of fluoropirimidine-based chemotherapy and the introduction of antiangiogenics and targeted therapies.<br>Pharmacologic blockade of the epithelial growth factor receptor (EGFR) with specific monoclonal antibodies, namely, cetuximab and panitumumab, represents the mainstay of tumour targeted therapy for mCRC in patients with tumors not harboring extended RAS pathway mutations (KRAS, NRAS, or BRAF). Such alterations, which constitutively activate typical EGFR downstream transducers, have been shown to trigger substitute survival pathways that bypass therapeutic blockade of EGFR signalling, thus abating the efficacy of anti-EGFR antibodies ("primary resistance"). Even when response to anti-EGFR therapy occurs in the context of appropriate molecular selection, acquired ("secondary") resistance inevitably arises in all cases. Our group has extensively studied this phenomenon and has shown that extended-RAS alterations are the principal culprit of anti EGFR acquired resistance, and that altered RAS clones decay upon anti-EGFR treatment withdrawal, while tumor cells regain sensitivity to anti EGFR treatment. We have also documented that ctDNA profiles of individuals who benefit from multiple challenges with anti-EGFR antibodies, exhibit pulsatile levels of mutant KRAS. Collectively, these results indicate that the CRC genome adapts dynamically to intermittent anti-EGFR drug schedules, and provide a molecular explanation for the efficacy of re-challenge therapies based on EGFR blockade. Our results also give experimental support to the empirical-based clinical benefit observed,

	following cetuximab or panitumumab rechallenge in two small series of originally KRAS exon 2 wild type mCRC patients.<br>We propose to assess the efficacy and safety of re-challenging with panitumumab RAS-extend wild type mCRC patients with ctDNA-confirmed secondary resistance to anti EGFR treatment, after progression on second or further lines chemotherapy. As proof-of-concept, patients will be blood monitored throughout their therapeutic itinerary for the presence of extended-RAS alterations and EGFR-ectodomain mutations by ctDNA determination (liquid biopsy). We also include in our ddPCR panel 7 different EGFR extracellular domain (ECD) mutations as they occur in 15-20% of patients who acquired resistance to anti-EGFR drugs.
Primary Objective	To evaluate the efficacy of Panitumumab re-challenge in RAS/RAF wild type metastatic colorectal cancer (mCRC), without plasmatic evidence of potentially resistant clones harbouring RAS or EGFR-ectodomain mutations.
Secondary Objective(s)	• To assess Progression Free Survival (PFS) and Overall Survival (OS) after panitumumab re-challenge.<br>• To determine the safety and tolerability of panitumumab re-challenge.
Translational Objective(s)	• To link the blood-presence of RAS-extended and EGFR extracellular (ECD) clones (if any) during panitumumab treatment with response and response duration.<br>• To describe by NGS the tumour ctDNA plasma landscape before and after panitumumab re-challenge.<br>• To determine potential associations, if existing, between different mutated ctDNA clones RAS-extended and EGFR extracellular (ECD) clones (if any) and response to panitumumab re-challenge.<br>• To retrospectively correlate (whenever possible) the decay kinetics of RAS-extended and EGFR extracellular (ECD) clones during anti-EGFR treatment holiday with response to panitumumab rechallenge.
Primary Endpoint	Overall response rate (ORR) to panitumumab according to RECIST v1.1.
Secondary Endpoint(s)	PFS; OS and Toxicity according to CTCAE version 4.03.

Translational endpoint(s)	Longitudinal extended RASmut ctDNA levels in plasma by ddPCR; plasma ctDNA landscapes by NGS Candiolo Panel at panitumumab baseline and progression.
Design	Open label, single-arm, multiple centers, Phase II trial. The trial has been designed to prove or disprove whether a rechallenge with panitumumab can achieve an objective response rate (ORR= CR+PR) of 30% or more in a population of RAS wild type mCRC patients selected on the basis of RAS extended clonal evolution in their plasma.
Population	Patients with metastatic colorectal cancer (mCRC), originally responsive to anti EGFR therapy, selected on the basis of RAS extended and EGFR ECD domain mutations absence in plasma.
Main Inclusion Criteria	1. Histologically confirmed diagnosis of metastatic colorectal cancer;<br>2. Age ≥ 18 years;<br>3. Written informed consent;<br>4. Documented WT RAS exons 2, 3 and 4 (KRas and NRas) and WT BRAF V600E for anti-EGFR treatment.<br>5. Complete or partial response to anti EGFR antibodies in any line-either received as monotherapy or in combination with chemotherapy;<br>6. Imaging documented progression while on therapy with a therapeutic regimen including anti-EGFR mAb;<br>7. Imaging documented progression at the last treatment regimen that must be anti-EGFR free;<br>8. Patient must be RAS and EGFR ectodomain wild type in a liquid biopsy performed no longer that 4 weeks after progression to the last anti-EGFR free treatment<br>9. FFPE sample used for eligibility to anti-EGFR prescription (see criteria 4) must be available for custom gene panel profiling (as described in appendix B). Otherwise if sample is not available, center must have already perfomed a genotyping on this tissue sample according to appendix B.<br>10. ECOG performance status ≤ 2;<br>11. At least one measurable tumor lesion as per RECIST v1.1. Lesions in previously irradiated areas or those that have received other loco-regional therapies (i.e. percutaneous ablation) should not be considered measurable unless there is clear documented evidence of progression of the lesion since therapy. Imaging must be performed maximum within 28 days prior to registration;<br>12. Normal organ functions;

	13. Negative serum pregnancy test within 1 week prior to the first study dose in all women of childbearing potential;<br>14. Subjects and their partners must be willing to avoid pregnancy during the trial. Male subjects with female partners of childbearing potential and female subjects of childbearing potential must, therefore, be willing to use adequate contraception;<br>15. Absence of any psychological, familial, sociological or geographical condition potentially hampering compliance with the study protocol and follow-up schedule; those conditions should be discussed with the patient before registration in the trial.
Main Exclusion Criteria	1. History of severe infusion reactions to monoclonal antibodies cetuximab or panitumumab;<br>2. Symptomatic or untreated leptomeningeal disease and symptomatic brain metastasis;<br>3. Clinically significant cardiac disease including:<br>a. congestive heart failure requiring treatment (NYHA grade ≥ 2), Left ventricular ejection fraction (LVEF) < 45% as determined by Multigated acquisition (MUGA) scan or echocardiogram;<br>b. history or presence of clinically significant ventricular arrhythmias or atrial fibrillation;<br>c. clinically significant resting bradycardia;<br>d. unstable angina pectoris ≤ 3 months prior to starting study drug;<br>e. acute myocardial infarction ≤ 3 months prior to starting study drug;<br>f. QTcF > 480 msec;<br>4. History of thromboembolic or cerebrovascular events within the last 6 months, including transient ischemic attack, cerebrovascular accident, deep vein thrombosis, or pulmonary embolism;<br>5. Patients with interstitial pneumonitis or pulmonary fibrosis;<br>6. Abnormal organ or bone marrow functions defined as:<br>a. Absolute neutrophil count < 1.5 x 10/L;<br>b. hemoglobin < 9 g/dL;

	c. alkaline phosphatase > 2.5 x upper normal limit (ULN), if liver metastases > 5 x ULN;<br>d. aspartate aminotransferase (AST)/ alanine aminotransferase (ALT) > 2.5 x ULN, if liver metastases > 5 x ULN;<br>e. bilirubin > 1.5 x ULN, if liver metastases > 2 x ULN;<br>f. serum creatinine > 1.5 x ULN and/or creatinine clearance ≤ 50 mL/min calculated according to Cockroft-Gault;<br>g. Patients with platelet count <100 x 10^9/L<br>7. Previous or concurrent second malignancy. Exceptions: adequately treated basal cell or squamous cell skin cancer; in situ carcinoma of the cervix, treated curatively and without evidence of recurrence for at least 3 years prior to study entry; or other solid tumor treated curatively and without evidence of recurrence for at least 3 years prior to study entry.<br>8.Patients with positive serology for HIV, HBV, HCV.<br>9. Patients with a history of severe or life threatening hypersensitivity to the active substance or to any of the excipients.
Treatments	Panitumumab 6 mg/kg in 100 cc 0.9% NaCl solution on Day 1 every two weeks by IV administration over 1 hour.
Sample Size	We used the A'Hern one-stage approach to calculate the sample size. For the primary objective of the amended protocol v.3.0, we will need to enroll 27 patients in order to achieve a power of at least 85% to test the null hypothesis that the rate of response to panitumumab would be 10% or less, versus the alternative hypothesis that the response rate would be 30% or more, at a one-sided alpha level of 0.05. Six objective responses are necessary to declare the study positive.<br>Protocol amendment number 2 restricts, while simplifying the eligibility criteria to patients with a negative liquid biopsy for extended RAS/RAF and EGFR ectodomain mutations, while eligibility in the prior version required a reduction of at least 50% of extended RAS/RAF positive clones. The expected Panitunumab rechallenge ORR remains however the same i.e. 30%, requiring under the same alfa and beta assumption a sample size of 27 patients. Therefore 27 patients need to be recruited in the amended protocol v3.0. Patients resulting positive to the Panel B will be analyzed by the intention-to-treat (ITT) approach

	and will be included in the efficacy analysis population (see statistical analysis paragraph 14.2).<br>In the previous protocol v. 2.1 dated 30.10.2017, four patients were enrolled with the previous criteria. All these patients left the trial for progression to panitumumab rechallenge at the first tumor assessment. These patients will be included in the Safety Evaluation Population.
Study Timeline	Expected Timeline Amendment #2 12 months<br>Planned FPI Amendment # 2 March 1 2019<br>Planned LPI Amendment # 2 March 1 2020<br>Planned LPLV Amendment # 2 March 1 2021<br>Total duration of the trial after its activation 42 months<br>Expected final report between May-June 2021.

4 SCHEDULE OF EVENTS

Assessment/Event	Pre study(1)	Cyle Q2wks Treatment day	End of treatment Visit (2)	FUP Visit(3)
	≤ 28 days	d 1 (day 15 = day 1 > cy 2)	> 4 wks from last TX	Q8 WKS
**Informed consent signed**	X			
**Blood Samples to determine the molecular eligibility for the trial**	X			
**History Demographics**	X			
**Panitumumab Administration**		X		
**FFPE availability (only if not already perfomed tissue genotyping according to appendix B)**	X			
**Performance Status**	X	X	X	X
**Physical exam**	X	X	X	X
**Concomitant meds**	X	Throughout the study		
**Adverse event evaluation**		Throughout the study		
**Hematology(4)**	X	X	X	
**Serum chemistry(5)**	X	X	X	
**Serology for HIV, HBV, HCV(6)**	X			
**Urinalysis**	X		X	
**CEA**		Q6 weeks		
**Serum or urine pregnancy test (7)**	X			
**ECG(8)**	X	Only if clinically indicated	X	
**LVEF**	X	Only if clinically indicated	X	
**Tumor assessment(9)**	X	Q8 weeks (+/- 3 days)	X	X
**Blood sample for molecular analysis during panitumumab rechallenge(10)**		X	X	X

1. Within 4 weeks of starting therapy. In case of anomalous values for Hematology, Serum chemistry, ECG and LVEF, urinalysis tests, the analyses must be repeated within 7 days before the date of treatment. Laboratory assessments performed within 72 hrs of study treatment will be considered good also for D1.
2. End of Treatment visit to be performed approximately 4 weeks after last treatment dose.
3. Follow-up visit for patients without documented PD at the time of treatment withdrawal: to be performed every approximately 8 weeks until PD or another anti-cancer therapy is initiated, whichever comes first. Evaluations to be performed according to clinical practice.
4. Hematology: hemoglobin, hematocrit, platelet count, total white blood cell count (WBC) and differential (neutrophil count, lymphocyte, monocyte, eosinophil, and basophil counts), red blood cell count (RBC). To be performed within 72 hours from the day reported on the schedule of assessment.
5. Serum Chemistry: sodium, potassium, chloride, bicarbonate, creatinine, calcium, albumin, total bilirubin, total protein, glucose, alkaline phosphatase, AST, ALT urea or BUN, INR, aPTT. To be performed within 72 hours from the day reported on the schedule of assessment.
6. Serology testing before treatment start for: Hepatitis B virus: HBsAg, antibodies against HBsAg, anti-hepatitis B core antibody (anti-HBcAb); Hepatiatis C virus: HCV antibody (anti-HCV);HIV testing: patients will be tested for HIV prior to the inclusion into the study. To Be done only if molecularly elegible to the rechallenge.
7. Within 1 week prior to the first study dose
8.12 lead electrocardiogram (ECG).
9.Tumor assessment will be performed every 8 weeks thereafter during treatment until PD; however, in order to fulfil RECIST, the FIRST occurrence of response MUST be reconfirmed after 28 days. Patients without documented PD at the time of treatment withdrawal should continue to have disease assessments done every approximately 8 weeks until PD or another anti-cancer therapy is initiated, whichever comes first.
10. Patients will be sampled at baseline (Day -7 to 1, prior to first panitumumab administration), every two weeks (d1 of each 2 weeks cycle) and at disease progression.

5 BACKGROUND AND STUDY RATIONALE

5.1 Rationale

Colorectal cancer (CRC) is the third most common cancer in the world and the second leading cause of cancer death in the United States and the European Union[1]. In the last decade, substantial advances in the treatment of the metastatic disease (mCRC) have more than doubled overall survival (OS) from 12 months to 30 months due to the refinement of fluoropirimidine-based chemotherapy and the introduction of antiangiogenics and targeted therapies[2].

Pharmacologic blockade of the epithelial growth factor receptor (EGFR) with specific monoclonal antibodies, namely, cetuximab and panitumumab, represents the mainstay of tumour targeted therapy for mCRC[3]. However, treatment with cetuximab or panitumumab, in front or second line combinations with a chemotherapy back-bone or as monotherapy in further lines, has resulted in limited clinical benefit when applied to molecularly unselected mCRC patients[4]. More substantial response and survival improvements have been obtained by excluding patients with tumours harboring extended RAS pathway mutations (KRAS, NRAS, or BRAF)[3]. Such alterations, which constitutively activate typical EGFR downstream transducers, have been shown to trigger substitute survival pathways that bypass therapeutic blockade of EGFR signaling, thus abating the efficacy of anti-EGFR antibodies ("primary resistance")[5-7]. Unfortunately even when response to anti-EGFR therapy occurs in the context of appropriate molecular selection, acquired ("secondary") resistance inevitably arises in all cases[5]. Several mechanisms of secondary resistance to anti EGFR targeted therapy have been unveiled by our and other, but the most prevalent by far is again associated with the emergence of RAS axis alterations in patients originally classified as RAS wild type[7,8]. Starting from that discovery, we exploited circulating tumor DNA (ctDNA) to longitudinally track EGFR wild type clonal evolution in patients while on treatment with EGFR-specific antibodies followed, at progression, by additional lines of chemotherapy administered alone or in combination with anti-angiogenic drugs, or monotherapy with the multikinase inhibitor regorafenib. By doing so we confirmed extended RAS genes alterations in the ctDNA of these patients as the prevalent mechanism of acquired resistance to EGFR blockade[9]. Most importantly, we also observed that emerging altered RAS clones declined upon withdrawal of EGFR-specific antibodies and remained below the limit of detection across subsequent lines of treatment (Fig. 1).

These findings led us to postulate that clonal evolution of tumor cell populations that survive treatment with EGFR-specific antibodies continues beyond the point of clinically established resistance.
To mechanistically evaluate this possibility, we studied in vitro CRC cells (DiFi) in which acquisition of resistance to cetuximab is accompanied by amplification of the KRAS gene[7,10]. To parallel withdrawal of EGFR blockade, which occurs when patients develop cetuximab resistance, two populations of resistant DiFi cells were cultured for 160 days in the absence of anti-EGFR antibody (Fig. 2a). Analogously to what we had observed in the blood of patients, KRAS copies declined significantly in both cell models (Student's t-test, $P \leq 0.01$) when the EGFR-specific antibody was suspended (Figs. 1 and 2). The cell populations that experienced antibody withdrawal regained partial sensitivity to cetuximab (Student's t-test $P \leq 0.001$), as compared to the population in which the drug pressure was maintained (Fig. 2a).

In summary, three important findings were revealed by the integrated body of our research. First, that extended-RAS alterations are the principal culprit of anti EGFR acquired resistance. Secondly, that altered RAS clones decay upon anti-EGFR treatment withdrawal, and cells regain sensitivity to anti EGFR treatment. Finally, that ctDNA profiles of individuals who benefit from multiple challenges with anti-EGFR antibodies exhibit pulsatile levels of mutant KRAS. Collectively, these results indicate that the CRC genome adapts dynamically to intermittent anti-EGFR drug schedules, and provide a molecular explanation for the efficacy of re-challenge therapies based on EGFR blockade. Our results also give experimental support to the empirical-based clinical benefit observed, following cetuximab[9] or panitumumab rechallenge[11,12] in two small series of originally KRAS exon 2 wild type mCRC patients.

We propose to assess the efficacy and safety of re-challenging with panitumumab RAS-extend wild type mCRC patients with ctDNA-confirmed secondary resistance to anti EGFR treatment, after progression on second line chemotherapy. As proof-of-concept, patients will be blood monitored throughout their therapeutic itinerary for the presence of extended-RAS alterations and EGFR-ectodomain mutations by ctDNA determination (liquid biopsy). We also include in our ddPCR panel

7 different EGFR extracellular domain (ECD) mutations as they occur in 15-20% of patients who acquired resistance to anti-EGFR drugs[13,14].

6 OBJECTIVES AND ENDPOINTS OF THE TRIAL

6.1 Objectives

6.1.1 Primary Objective

- To evaluate the efficacy of Panitumumab re-challenge in RAS/RAF wild type metastatic colorectal cancer (mCRC), without plasmatic evidence of potentially resistant clones[14] harbouring RAS or EGFR-ectodomain mutations.

6.1.2 Secondary Objectives

- To assess Progression Free Survival (PFS) and Overall Survival (OS) after panitumumab re-challenge.
- To determine the safety and tolerability of panitumumab re-challenge.

6.1.3 Translational Objectives

- To link the blood-presence of RAS-extended and EGFR extracellular (ECD) clones (if any) during panitumumab treatment with response and response duration.
- To describe by NGS the tumour ctDNA plasma landscape before and after panitumumab re-challenge.
- To determine potential associations, if existing, between different mutated ctDNA clones RAS-extended and EGFR extracellular (ECD) clones (if any) and response to panitumumab re-challenge.
- To retrospectively correlate (whenever possible) the decay kinetics of RAS-extended and EGFR extracellular (ECD) clones during anti-EGFR treatment holiday with response to panitumumab rechallenge.

6.2 End-Points

6.2.1 Primary End-point

- Overall response rate (ORR) to panitumumab according to RECIST v1.1.

6.2.2 Secondary End-point

- PFS; OS and Toxicity according to CTCAE version 4.03.

6.2.3 Translational End-point

- Longitudinal extended RAS mut ctDNA levels in plasma by ddPCR; plasma ctDNA landscapes by NGS Candiolo Panel at panitumumab baseline and progression.

7 TRIAL DESIGN

This is a hypothesis driven, open label, single-arm, multiple centers, Phase II trial. The trial has been designed to prove or disprove whether a rechallenge with panitumumab can achieve an objective response rate (ORR= CR+PR) of 30% or more in a population of RAS wild type mCRC patients selected on the basis of RAS extended clonal evolution in their plasma.

7.1 Patient Population

The study has been designed on the basis of three translational findings, as described in the background. Namely that: i) no more than 20% of newly metastatic CRC patients have a tumor "addicted" to EGFR that respond and then become resistant to anti EGFR therapy; ii) in a large majority of cases, resistance to anti-EGFR therapy is due to the emergence, under the anti EGFR treatment selective pressure, of a pre-existing RAS mutant cellular clone(s); iii) RAS-altered clones will spontaneously decay upon anti-EGFR treatment withdrawal (anti EGFR therapy 'holiday'), and cells will regain sensitivity to a second anti EGFR treatment.
Accordingly, to select the right population, we assumed the following

- Within an extended RAS wild type population of first metastatic CRC patients, approximately 20% of cases will have EGFR addicted tumors.
- EGFR addicted patients will achieve an Objective Response (CR o PR) to first therapy containing an anti EGFR monoclonal antibody, which can't, however, be dissected from the response to the primary chemotherapy.
- At least 70% of the addicted/responding patients will eventually develop a RAS and/or EGFR ECD mediated resistance.

- After a period of anti EGFR treatment holiday, the RAS mutational load (measured in the plasma ctDNA) in at least 60% of these patients should drop according to an exponential decay kinetic[15].
- Only patients showing absence of RAS and EGFR ectodomain mutations might benefit from a rechallenge with panitumumab.

7.2 Patients' selection process

Given the assumption, and due to the proof-of-concept nature of the trial, we adopted a patients' selection process consisting of i) a Screening Phase, and ii) a therapeutic Trial Phase. The correspondent study flow-chart is shown in Figure 3.

Only patients having received a previous regimen including anti EGFR therapy will be considered for the Screening Phase. It is expected that up to 75% of patients will have received panitumumab. Patients showing absence of RAS and EGFR ectodomain mutations at RML will be declared "molecularly eligible" for the Trial Phase.

7.2.1 Screening Phase

The purpose of the Screening Phase is to determine the "molecular eligibility" of the patients for panitumumab re-challenge. Once a patient has been identified "screenable", the treating physician will seek his/her Informed Consent.

During screening phase, patients will be liquid biopsied (LB) at one optional (BML) and one mandatory (RML) check-points and their ctDNA tested by ddPCR to monitor the emergency, and subsequent decay, of RAS altered clones or EGFR ectodomain mutated clones.

The RAS Baseline Mutational Load (BML) samples will be optionally collected after the last anti-EGFR and acknowledgment of the PD upon first anti-EGFR treatment. Patients progressing during or after subsequent lines of therapy will be retested at the Rechallenge Mutational Load (RML) checkpoint. Patients showing absence of RAS and EGFR ectodomain mutational load at RML will be declared "molecularly eligible" for the Trial Phase. At each checkpoint we will also collect the corresponding radio-imaging data.

7.2.2 Trial Phase

Patients resulting “molecularly eligible” at the RML checkpoint, having satisfied all other eligibility criteria, will be treated with panitumumab monotherapy at standard dose until documented radiological progression or unacceptable toxicity or any other reason (See Section 9.2.3) whichever comes first.

7.3 Enrolment

Only patients provided with a properly processed interventional liquid biopsy (RML) obtained at last chemotherapy (see 21.3) can be enrolled in CHRONOS. In addition, if not already performed, patients will assay within 3 months from the panitumumab rechallenge start, the negativity for a minimum set of gene mutations (defined in appendix B) assayed through a multiplex gene profiling platform (i.e. Foundation Medicine platform, Personal Genomic Diagnostic platform, MSK-IMPACT platform) at diagnosis of metastatic disease or at study screening.

7.4 Study logistical plan

Plasma ct-DNA determination by ddPCR and NGS will be undertaken by the Dept of Oncology, University of Torino, located at the Institute of Candiolo IRCCS. Tumor tissue that has not already been genotyped at participating center will be tested with the Panel of genes reported in Appendix B (performed through the following platform or equivalent: Foundation Medicine platform, Personal Genomic Diagnostic platform, MSK-IMPACT platform), including tumors of patients referred from non-participating centers; Radio-imaging will be collected, stored and centrally analyzed by the means of the mintLesions ® or equivalent software.

8 STUDY POPULATION

8.1 Inclusion Criteria

1 Histologically confirmed diagnosis of metastatic colorectal cancer;

2 Age ≥ 18 years;

3 Written informed consent;

4 Documented WT RAS exons 2, 3 and 4 (KRas and NRas) and WT BRAF V600E for anti-EGFR treatment.

5 Complete or partial response to anti EGFR antibodies in any line–either received as monotherapy or in combination with chemotherapy;

6 Imaging documented progression while on therapy with a therapeutic regimen including anti-EGFR mAb;

7 Imaging documented progression at the last treatment regimen that must be anti-EGFR free;

8 Patient must be RAS and EGFR ectodomain wild type in a liquid biopsy performed no longer that 4 weeks after progression to the last anti-EGFR free treatment

9 FFPE sample used for eligibility to anti-EGFR prescription (see criteria 4) must be available for custom gene panel profiling (as described in appendix B). Otherwise if sample is not available, center must have already perfomed a genotyping on this tissue sample according to appendix B.

10 ECOG performance status ≤ 2;

11 At least one measurable tumor lesion as per RECIST v1.1. Lesions in previously irradiated areas or those that have received other loco-regional therapies (i.e. percutaneous ablation) should not be considered measurable unless there is clear documented evidence of progression of the lesion since therapy. Imaging must be performed maximum within 28 days prior to registration;

12 Normal organ functions;

13 Negative serum pregnancy test within 1 week prior to the first study dose in all women of childbearing potential;

14 Subjects and their partners must be willing to avoid pregnancy during the trial. Male subjects with female partners of childbearing potential and female subjects of childbearing potential must, therefore, be willing to use adequate contraception;

15 Absence of any psychological, familial, sociological or geographical condition potentially hampering compliance with the study protocol and follow-up schedule; those conditions should be discussed with the patient before registration in the trial.

8.2 Exclusion Criteria

1. History of severe infusion reactions to monoclonal antibodies cetuximab or panitumumab;
2. Symptomatic or untreated leptomeningeal disease and symptomatic brain metastasis;
3. Clinically significant cardiac disease including:
    a. congestive heart failure requiring treatment (NYHA grade ≥ 2), Left ventricular ejection fraction (LVEF) < 45% as determined by Multigated acquisition (MUGA) scan or echocardiogram;
    b. history or presence of clinically significant ventricular arrhythmias or atrial fibrillation;
    c. clinically significant resting bradycardia;
    d. unstable angina pectoris ≤ 3 months prior to starting study drug;
    e. acute myocardial infarction ≤ 3 months prior to starting study drug;
    f. QTcF > 480 msec;
4. History of thromboembolic or cerebrovascular events within the last 6 months, including transient ischemic attack, cerebrovascular accident, deep vein thrombosis, or pulmonary embolism;
5. Patients with interstitial pneumonitis or pulmonary fibrosis;
6. Abnormal organ or bone marrow functions defined as:
    a. Absolute neutrophil count < 1.5 x 10/L;
    b. hemoglobin < 9 g/dL;

c. alkaline phosphatase > 2.5 x upper normal limit (ULN), if liver metastases > 5 x ULN;
d. aspartate aminotransferase (AST)/ alanine aminotransferase (ALT) > 2.5 x ULN, if liver metastases > 5 x ULN;
e. bilirubin > 1.5 x ULN, if liver metastases > 2 x ULN;
f. serum creatinine > 1.5 x ULN and/or creatinine clearance ≤ 50 mL/min calculated according to Cockroft-Gault;
g. Patients with platelet count <100 x 10^9/L

7. Previous or concurrent second malignancy. Exceptions: adequately treated basal cell or squamous cell skin cancer; in situ carcinoma of the cervix, treated curatively and without evidence of recurrence for at least 3 years prior to study entry; or other solid tumor treated curatively and without evidence of recurrence for at least 3 years prior to study entry.
8. Patients with positive serology for HIV, HBV, HCV.
9. Patients with a history of severe or life threatening hypersensitivity to the active substance or to any of the excipients.

8.3 Patients substitution criteria

8.3.1 Severe Infusion reaction (Trial Phase only)

Patient experiencing **severe infusion reaction to panitumumab** requiring permanent drug discontinuation will be substituted.

9 TREATMENT (TRIAL PHASE ONLY)

9.1 Description of investigational product

Panitumumab is a recombinant, fully human IgG2 monoclonal antibody that binds with high affinity and specificity to the ligand-binding domain of human EGFR and inhibits receptor autophosphorylation induced by all known EGFR ligands. Binding of panitumumab to EGFR results

in internalization of the receptor, inhibition of cell growth, induction of apoptosis and decreased interleukin 8 and vascular endothelial growth factor production.

Panitumumab exhibits clinical activity as monotherapy or in combination with chemotherapy in wild type RAS mCRC. In Italy panitumumab is indicated for the treatment of adult patients with exon 2,3,4 K and N RAS wild type mCRC in first-line in combination with FOLFOX or FOLFIRI, in second-line in combination with FOLFIRI for patients who have received first-line fluoropyrimidine-based chemotherapy (excluding irinotecan) and as monotherapy after failure of fluoropyrimidine-, oxaliplatin-, and irinotecan-containing chemotherapy regimens.

The most common AE of panitumumab is skin toxicity occurring in 90% of patients (grade 3 NCI-CTC in 25%; grade 4 NCI-CTC < 1%) including rash (45%), dermatitis acneiform (39%), pruritus (35%), erythema (30%) and dry skin (22%); AEs present in ≥ 20% of patients are: gastrointestinal disorders [diarrhea (50%), nausea (41%), vomiting (27%), constipation (23%) and abdominal pain (23%)]; general disorders [fatigue (37%), pyrexia (20%)]; anorexia (27%); infections and infestations [paronychia (20%)]. Panitumumab is mainly distributed into the vascular space and exhibits nonlinear pharmacokinetics that are consistent with target-mediated drug disposition, involving saturable binding to EGFR and subsequent internalization and degradation inside the cells. Following the recommended dose regimen (6 mg/kg given once every 2 weeks as a 1-hour infusion), panitumumab concentrations reach steady-state levels by the third infusion with mean (± Standard Deviation [SD]) peak and trough concentrations of 213 ± 59 and 39 ± 14 mcg/ml, respectively. The mean (± SD) AUC0-tau and CL are 1306 ± 374 mcg•day/ml and 4.9 ± 1.4 ml/kg/day, respectively. The elimination half-life is approximately 7.5 days (range: 3.6 to 10.9 days). Panitumumab is also cleared in a linear fashion by the reticuloendothelial system, similarly to other endogenous immunoglobulins. A population pharmacokinetic analysis was performed to explore the potential effects of selected covariates on panitumumab pharmacokinetics. Results suggest that age (21-88), gender, race, hepatic function, renal function, chemotherapeutic agents, and EGFR membrane staining intensity (1+, 2+, 3+) in tumour cells had no apparent impact on the pharmacokinetics of panitumumab.

Panitumumab is supplied as 20 mg/ml concentrate for solution for infusion. It must be stored in a refrigerator (2°C – 8°C), protected from light.

The recommended dose of panitumumab is 6 mg/kg of body weight given once every two weeks. Prior to infusion, panitumumab should be diluted in sodium chloride 9 mg/ml (0.9%) solution for injection to a final concentration not to exceed 10 mg/ml.

Panitumumab must be administered as an intravenous infusion via an infusion pump, using a low protein binding 0.2 or 0.22 micrometer in-line filter, through a peripheral line or indwelling catheter. The recommended infusion time is approximately 60 minutes. If the first infusion is tolerated, then subsequent infusions may be administered over 30 to 60 minutes. Doses higher than 1000 mg should be infused over approximately 90 minutes.

9.2 Pregnancy and contraception

ICH M3 guidance requires precautions to be taken to minimize risk to fetus or embryo when including women of childbearing potential in clinical studies.

These precautions include the use of highly effective contraceptive measures, excluding pregnancy at baseline (serum test), continued pregnancy monitoring, and continued pregnancy testing for up to 7 months following last dose of study drug (follow-up period based on PK considerations).

Women of childbearing potential (who have not undergone surgical sterilization with a hysterectomy and/or bilateral oophorectomy) and men with partners of childbearing potential must agree to use a highly effective nonhormonal form of contraception or two effective forms of non-hormonal contraception by the patient and/or partner.

Methods that can achieve a failure rate of less than 1% per year when used consistently and correctly are considered as highly effective birth control methods. Such methods include:

- combined (estrogen and progestogen containing) hormonal contraception associated with inhibition of ovulation
  - oral
  - intravaginal
  - transdermal
- progestogen-only hormonal contraception associated with inhibition of ovulation:
  - oral
  - injectable
  - implantable
- intrauterine device (IUD)
- intrauterine hormone-releasing system (IUS)
- bilateral tubal occlusion

- vasectomised partner[1]
- sexual abstinence[2]

9.3 Treatment Plan

9.3.1 Drug Administration

Treatment will be administered on an outpatient basis. No investigational or commercial agents or therapies other than those described below may be administered with the intent to treat the patient's malignancy.

**Regimen Description**				
Agent	Precautions	Dose	Route	Schedule
Panitumumab	Avoid exposure to sunlight	6 mg/kg in 100 cc 0.9% NaCl solution	IV over 1 hour	Day 1 every 2 weeks

The panitumumab dose will be calculated based on the subject's actual body weight at baseline and re-calculated at subsequent doses per institutional guidelines. At a minimum, the dose will be re-calculated if the actual body weight changes by at least 10%. Panitumumab will be administered IV by an infusion pump through a peripheral line or indwelling catheter using a non-pyrogenic, low protein binding filter with a 0.2 or 0.22-micron in-line filter infusion set-up over 1 hour ± 15 minutes by a trained healthcare professional.

If the first infusion of panitumumab is well tolerated (ie without any serious infusion-related reactions) all subsequent infusions may be administered over 30 ± 10 minutes. In the event a subject's actual weight requires greater than 150 mL volume infusion, panitumumab will be administered over 60 to

1

Vasectomised partner is a highly effective birth control method provided that partner is the sole sexual partner of the WOCBP trial participant and that the vasectomised partner has received medical assessment of the surgical success.

2

Sexual abstinence is considered a highly effective method only if defined as refraining from heterosexual intercourse during the entire period of risk associated with the study treatments. The reliability of sexual abstinence needs to be evaluated in relation to the duration of the clinical trial and the preferred and usual lifestyle of the subject.

90 minutes ± 15 minutes, as tolerated. Doses higher than 1000 mg should be diluted to 150 mL in 0.9% sodium chloride solution, USP (saline solution) and infused over 60 to 90 ± 15 minutes.

9.3.2 Premedication and patient monitoring during infusion

Pre-medications are not required for panitumumab but medications such as corticosteroids and anti-histamines may be administered to treat an existing infusion reaction or as pre-medication for a subject who has previously experienced an infusion reaction at the discretion of the investigator.
Patients will be monitored for any signs of AEs after their first dose of panitumumab for at least 60 minutes.

9.3.3 Duration of Therapy

Treatment may continue until one of the following criteria applies:

- Disease progression;
- Intercurrent illness that prevents further administration of treatment/death;
- Unacceptable adverse event(s);
- Patient decides to withdraw from the study, or
- General or specific changes in the patient's condition render the patient unacceptable for further treatment in the judgment of the investigator.

9.4 Concomitant Therapy

Throughout the study, investigators may prescribe any concomitant medications or treatments deemed necessary to provide adequate supportive care.
Anticancer therapy (chemotherapy, biologic or radiation therapy, palliative radiotherapy covering > 25% of the bone marrow reserve, and surgery) must not be given to patients during the study treatment.
Radiotherapy for palliation in non-target lesions with antalgic aim is allowed.
Recombinant human granulocyte colony stimulating factor (G-CSF) or erythropoiesis stimulating agent use according to the current approved label or institutional guidelines is permitted.

9.5 Dose Adjustments

Doses will be reduced for hematological and other adverse events. Dose adjustments are to be made according to the greatest degree of toxicity. Adverse events will be graded using the NCI Common Terminology Criteria for Adverse Events (CTCAE) Version 4.03 (download from the CTEP web site http://ctep.cancer.gov/protocolDevelopment/electronic_applications/ctc.htm).

The major adverse effects of panitumumab which limit dose is dermatologic reactions and soft tissue toxicity. Are also common anemia, gastrointestinal disorders, hypokalemia, hypomagnesaemia, anorexia, conjunctivitis, dyspnea and cough.

The guidelines that follow outline dose adjustments for several of these toxic effects. If a patient experiences several adverse events and there are conflicting recommendations, please use the recommended dose adjustment that reduces the dose to the lowest level.

9.5.1 Panitumumab Dose Reductions

Dose Level	Percentage (%)	Panitumumab
Starting Dose	100	6 mg/kg Q2W
-1	80	4.8 mg/kg Q2W
-2	60	3.6 mg/kg Q2W

9.5.2 Criteria for Withholding a Dose of Panitumumab

Panitumumab will be withheld if any of the following related toxicities occur:

- Skin- or nail-related toxicities:
  - Any grade ≥ 3 toxicity;
  - Any skin- or nail-related SAE;
- Non-skin- or non-nail-related toxicities:
  - Any grade 3 or 4 toxicity with the following exceptions:
    - ♦ Panitumumab will only be withheld for grade ≥ 3 hypomagnesemia and/or hypocalcemia that persists despite maximal (ie, IV replacement) magnesium and/or calcium replacement;
    - ♦ Panitumumab will only be withheld for grade ≥ 3 nausea, diarrhea, or vomiting that persists despite optimal supportive care;

♦ Panitumumab will only be withheld for grade ≥ 3 anemia or grade 4 thrombocytopenia that can't be managed by transfusion(s).

9.5.3 Criteria for Re-treatment with Panitumumab

Skin- or nail-related toxicities:

Panitumumab administration may recommence once the reason for withholding the dose of panitumumab has resolved:

- Systemic steroids are no longer required, or
- The skin- or nail-related toxicity is no longer intolerable to the subject, or
- IV antibiotic or IV antifungal treatment is no longer required, or
- The AE has improved to grade ≤ 2 or returned to baseline, and is no longer considered serious.

Non-skin- or nail-related toxicities:

Panitumumab administration may recommence at the next planned visit once the AE has improved to ≤ grade 1 or returned to baseline, unless otherwise indicated (see Section 9.4.5 and 9.4.6).

9.5.4 Dose Modification Schedule for Panitumumab

Subjects who have had their dose of panitumumab withheld for one or more reasons listed in Section 9.4.2 and then meet the criteria for re-treatment as listed in section 9.4.3 will recommence panitumumab at the next planned visit according to the following schedule:

**Occurrence of skin- or nail-related toxicities: ≥ grade 3**	**Administration of panitumumab**	**Outcome**	**Dose regulation**
Initial occurrence	Withhold 1 or 2 doses	Improved (≤ grade2)	Continuing infusion at 100% of original dose
		Not recovered	Discontinue
At the second occurrence	Withhold 1 or 2 doses	Improved (≤ grade2)	Continuing infusion at 80% of original dose
		Not recovered	Discontinue
At the third occurrence	Withhold 1 or 2 doses	Improved (≤ grade2)	Continuing infusion at 60% of original dose
		Not recovered	Discontinue
At the fourth occurrence	Discontinue	-	-

**Occurrence of Non-skin- or Non-nail-related toxicities:** ≥ grade 3 (for the exceptions see above)	**Administration of panitumumab**	**Outcome**	**Dose regulation**
Initial occurrence	Withhold 1 or 2 doses	Improved (≤ grade1)	Continuing infusion at 100% of original dose
		Not recovered	Discontinue
At the second occurrence	Withhold 1 or 2 doses	Improved (≤ grade1)	Continuing infusion at 80% of original dose
		Not recovered	Discontinue
At the third occurrence	Withhold 1 or 2 doses	Improved (≤ grade1)	Continuing infusion at 60% of original dose
		Not recovered	Discontinue
At the fourth occurrence	Discontinue	-	-

It is recommended that panitumumab doses will be escalated in subjects whose toxicity resolves to the degree that meets the criteria for re-treatment with panitumumab. Dose escalations are recommended but not required.

Dose escalations should occur in the following manner:

- Subjects treated at the 80% dose level whose toxicity does not recur should receive the 100% dose level at the next dose unless a previous attempt to re-escalate to the 100% dose level was not tolerated (re-initiation of the 80% dose is allowed as an alternative to dose escalation).
- Subjects treated at the 60% dose level whose toxicity does not recur should receive the 80% dose at the next dose unless a previous attempt to re-escalate to the 80% dose level was not tolerated (re-initiation of the 60% dose is allowed as an alternative to dose escalation).

9.5.5 Delayed or Missed Doses

If panitumumab is not given within ± 3 days of the planned visit, then the dose will be considered missed. The next dose should be given at the next planned visit. Missed doses will not be made up.

9.5.6 Discontinuation

Subjects for whom a delay of panitumumab administration is due to a related toxicity and is of > 6 weeks (from the planned administration date, ie, ≥ 3 consecutively missed doses) will be considered intolerant and be permanently discontinued.

9.5.7 Management of dermatologic toxicity and recommended concomitant treatment

Pre-emptive treatment of anticipated skin toxicity is strongly recommended since it can halve the incidence rate of grade 2 or higher skin toxicity from approximately 60% to 30%, including a reduction from 21% to 6% of grade 3 toxicity.
Subjects treated with panitumumab should receive the pre-emptive regimen 24 hours before study Day 1 and continue for at least 6 weeks, preferably for the duration of treatment. Proactive skin treatment includes:

- skin moisturiser and sun screen (SPF > 15 UVA and UVB), to be applied to face, hands, feet, neck, back and chest every morning during treatment
- topical steroid cream (not stronger than 1% hydrocortisone) to be applied face, hands, feet, neck, back and chest every night during treatment.

Oral antibiotic (e.g. doxycycline) may be useful in the management of dermatologic reactions.
Subjects who experience dermatological toxicities that meet the criteria defined in Section 9.4.2 should have panitumumab dose withheld as described in section 9.4.4.

10 CLINICAL EVALUATION, LABORATORY TESTS, FOLLOW-UP

10.1 Before Treatment Start

Given the progressively restrictive selection only a fraction of the patients undergoing the molecular screening will proceed to the Trial Phase.
They following information must be present:

- Dated IEC/IRB approved informed consent form;
- An imaging documented progression at last treatment with anti-EGFR
- An imaging documented progression at last line therapy not including anti-EGFR mAb;

- Documented absence of RAS extended, RAF and EGFR ectodomain mutations clones at liquid biopsy performed no longer than 4 weeks before trial entry;
- Documentation of demographics, relevant medical history, concomitant medication;
- Detailed information on prior cancer treatment (previous front-line chemotherapy and anti-EGFR mAb treatment, start date, stop date, best response and best response date, date of PD, reason for stopping; previous line chemotherapies, start date, stop date, best response and best response date, date of PD, reason for stopping.
- Tumor histology, location and extent of disease, tumor tissue RAS and BRAF genotyping assayed with the Panel of genes reported in Appendix B, or RAS and BRAF status at initial diagnosis and the FFPE block or at least 10 unstained slides of the diagnostic paraffin tumor;
- Safety laboratory (Blood count with WBC count and differential, sodium, potassium, chloride, urea, magnesium, creatinine, albumin, total protein, total bilirubin, alkaline phosphatase, ALT, AST, calcium, phosphorous, uric acid, INR, aPTT, pregnancy test [if applicable]);

- Baseline Safety (symptoms and persisting toxicities from prior therapy to check compliance with inclusion criteria);
- Serology testing for :
  - Hepatitis B virus: HBsAg, antibodies against HBsAg, anti-hepatitis B core antibody (anti-HBcAb);
  - Hepatiatis C virus: HCV antibody (anti-HCV);.
  - HIV testing: patients will be tested for HIV prior to the inclusion into the study
- Physical examination including assessment of vital signs (blood pressure, heart rate, body temperature), body weight, height, ethnicity, dermatological examination (CTCAE v4 scaling);
- ECOG performance status;
- 12-lead ECG;
- Tumor assessment. Computed tomography (CT) or magnetic resonance imaging (MRI) of the chest and abdomen must have been performed no longer than 28 days prior to first treatment. Imaging must be performed with contrast and baseline status of the tumor disease

using RECIST v1.1. (note it could coincide with the radioimaging of the second line therapy progression). Additional imaging (such as bone scan) must be performed when other areas of disease are suspected. In addition, all imaging evaluation performed during the pre-trial screening phase must be available.

Refer to the Summary table (Section 4.2) for an outline of procedures required at each visit.

10.2 During Treatment

The following assessments are to be performed according to the following schedule during the treatment phase in all subjects:

Every two weeks:

- Physical examination (normal/abnormal, weight);
- Vital signs (body temperature, heart rate, and blood);
- ECOG performance status;
- Laboratory tests local (hematology, coagulation, and serum chemistry);
- AEs (signs and symptoms);
- Concomitant medications (all concomitant medications including prophylactic use of antibiotics);
- Blood sampling for molecular analysis.

Every 8 weeks:

Tumor assessment. CT or MRI of the chest and abdomen. Additional imaging must be performed when other areas of disease are known or suspected. Tumor burden evaluation per RECIST v1.1. (+/- 3 days).

Refer to the Schedule of Events (See Section 4.2) for an outline of the frequency of the required procedures.

10.3 After the End of Treatment (Follow-Up)

Patients who discontinue study treatment should be scheduled for a safety follow-up visit within 28 days after the last dose of study or after the decision to discontinue study treatment.

This visit includes:

- Physical examination, weight, vital signs;
- ECOG PS;
- ECG;
- Laboratory tests local (hematology, coagulation, and serum chemistry);
- Tumor assessment. CT or MRI of the chest and abdomen. Additional imaging must be performed when other areas of disease are known or suspected. Tumor burden evaluation per RECIST v1.1;
- AEs;
- Concomitant medications/procedures;
- Blood sample for NGS molecular analysis;

An End of Treatment eCRF page should be completed, giving the date and reason for stopping the study treatment.

Patients who discontinue study treatment for any reason prior to disease progression (except informed consent withdrawn) should return for disease follow-up and blood sampling for the plasma longitudinal ct-DNA study every 8 weeks until progression, with a last sampling on day 28 post progression, at which time they will be considered withdrawn from the study.

If the subject ends treatment for reasons other than documented disease progression, then CT or MRI scans will be performed at end-of treatment visit and then every 8 weeks (+/- 3 days) until radiographically-confirmed disease progression, start of new cancer treatment, death, withdrawal of consent or at the end of the study, whichever is earlier. Assessments will be made by the Investigator using RECIST v1.1.

11 EFFICACY ASSESSMENT

11.1 Definitions

All eligible patients will be included in the response rate calculation (primary end-point). The subsets that will be assigned a response category (CR, PR, SD or PD; see definitions below) are all patients who have received at least one treatment and have their disease re-evaluated. Patients on will have their response classified according to the definitions set out below.

11.2 Tumour assessments

Tumour assessments will be performed at each center by local radiologists according to RECIST version 1.1. Screening/baseline imaging assessments may be performed up to 28 days prior of treatment start on-study tumor assessments every 8 weeks with a ± 3 day window of variance. In case of discontinuation of treatment for any reason other than disease progression, patients will still be re-evaluated every 8 weeks until disease progression or start of a new treatment or death or patients refusal, whichever comes first. At trial end, all in-Trial tumor assessments, and the radioimaging data at each of the Trial checkpoints (BML and RML) will be reviewed centrally by two radiologists who will read the CT/MRI scans blinded using the mintLesion™ software to collect, store, and guide the revision of the imaging results. The imaging review protocol and tumour assessment re-conciliation report will be included in the final study report and or in the publication of the study.

The complete criteria are included in the published RECIST document also available at http://www.eortc.be/RECIST.

11.2.1 Measurability of Tumour Lesions at Baseline

11.2.1.1 Definitions

- **Measurable disease** - the presence of at least one measurable lesion. If the measurable disease is restricted to a solitary lesion, its neoplastic nature should be confirmed by cytology/histology.
- **Measurable lesions** - *tumour lesions* that can be accurately measured in at least one dimension (longest diameter to be recorded) as ≥ 20 mm with chest x-ray, and as ≥ 10 mm with CT scan or clinical examination [using calipers]. Bone lesions are considered measurable only if assessed by CT scan and have an identifiable soft tissue component that meets these requirements (soft tissue component ≥ 10 mm by CT scan). *Malignant lymph nodes* must be ≥ 15 mm in the short axis to be considered measurable; only the short axis will be measured and followed. All tumour measurements must be recorded in millimeters (or decimal fractions of centimeters) by use of a ruler or calipers. Tumour lesions situated in a previously irradiated area, or in an area subjected to other loco-regional therapy, are usually not considered measurable unless there has been demonstrated progression in the lesion. Provide detail on the conditions under which such lesions would be considered measurable.

- **Non-measurable lesions** - All other lesions (or sites of disease), including small lesions are considered non-measurable disease. Bone lesions without a measurable soft tissue component, leptomeningeal disease, ascites, pleural/pericardial effusions, lymphangitis cutis/pulmonis, inflammatory breast disease, lymphangitic involvement of lung or skin and abdominal masses followed by clinical examination are all non-measurable. Nodes that have a short axis <10 mm at baseline are considered non-pathological and should not be recorded or followed.
- **Target Lesions.** When more than one measurable tumour lesion or malignant lymph node is present at baseline all lesions up to *a maximum of 5 lesions total* (and a maximum of *2 lesions per organ*) representative of all involved organs should be identified as target lesions and will be recorded and measured at baseline. Target lesions should be selected on the basis of their size (lesions with the longest diameter), be representative of all involved organs, but in addition should be those that lend themselves to *reproducible repeated measurements*. Note that pathological nodes must meet the criterion of a short axis of ≥ 15 mm by CT scan and only the *short* axis of these nodes will contribute to the baseline sum. At baseline, the sum of the target lesions (longest diameter of tumour lesions plus short axis of lymph nodes: overall maximum of 5) is to be calculated and recorded.
- **Non-target Lesions**. All non-measurable lesions (or sites of disease) including pathological nodes (those with short axis ≥ 10 mm but < 15 mm), plus any measurable lesions over and above those listed as target lesions are considered *non-target lesions*. Measurements are not required but these lesions should be noted at baseline and should be followed as “present” or “absent”.

All baseline evaluations should be performed as closely as possible to the beginning of treatment and never more than 4 weeks before the beginning of the treatment.

11.2.1.2 Methods of Measurements

The same method of assessment and the same technique should be used to characterize each identified and reported lesion at baseline and during follow-up. Assessments should be identified on a calendar schedule and should not be affected by delays in therapy. While on study, all target lesions recorded at baseline should have their actual measurements recorded on the CRF at each subsequent evaluation, even when very small (e.g. 2 mm). If it is the opinion of the radiologist that

the lesion has likely disappeared, the measurement should be recorded as 0 mm. If the lesion is believed to be present and is faintly seen but too small to measure, a default value of 5 mm should be assigned. For lesions which fragment/split add together the longest diameters of the fragmented portions; for lesions which coalesce, measure the maximal longest diameter for the "merged lesion".

- Clinical Lesions. Clinical lesions will only be considered measurable when they are superficial and ≥ 10 mm as assessed using calipers (e.g. skin nodules). For the case of skin lesions, documentation by color photography including a ruler to estimate the size of the lesion is recommended. If feasible, imaging is preferred.
- CT, MRI. CT is the best currently available and reproducible method to measure lesions selected for response assessment. This guideline has defined measurability of lesions on CT scan based on the assumption that CT slice thickness is 5 mm or less. When CT scans have slice thickness greater than 5 mm, the minimum size for a measurable lesion should be twice the slice thickness. MRI is also acceptable in for abdomen imaging.
- All measurements should be taken and recorded in metric notation using a ruler or calipers.

11.2.2 Tumour Response Evaluation

The activity of panitumumab will be assessed by the investigator according to the RECIST v1.1 (refer to the table below).

Complete Response (CR): disappearance of all *target* and *non-target* lesions and normalization of tumour markers. Pathological lymph nodes must have short axis measures < 10 mm (Note: continue to record the measurement even if < 10 mm and considered CR). Tumour markers must have normalized. Residual lesions (other than nodes < 10 mm) thought to be non-malignant should be further investigated (by cytology or PET scans) before CR can be accepted.

Partial Response (PR): at least a 30% decrease in the diameter of the target lesion or in the sum of measures (longest diameter for tumour lesions and short axis measure for nodes) of target lesions, taking as reference the baseline measurement or sum of diameters. Non target lesions must be non-PD.

Objective Response Rate: the sum of partial responses plus complete responses.

Stable Disease (SD): Neither sufficient shrinkage to qualify for PR nor sufficient increase to qualify for PD taking as reference the smallest sum of diameters on study.

Progressive Disease (PD): at least a 20% increase in the sum of diameters of measured lesions taking as references the smallest sum of diameters recorded on study (including baseline) AND an absolute increase of ≥ 5 mm. Appearance of new lesions will also constitute PD (including lesions in previously unassessed areas). In exceptional circumstances, unequivocal progression of non-target disease may be accepted as evidence of disease progression, where the overall tumour burden has increased sufficiently to merit discontinuation of treatment, for example where the tumour burden appears to have increased by at least 73% in volume (which is the increase in volume when all dimensions of a single lesion increase by 20%). Modest increases in the size of one or more non-target lesions are NOT considered unequivocal progression. If the evidence of PD is equivocal (target or non-target), treatment may continue until the next assessment, but on further documentation, the earlier date must be used.

Integration of target, non-target and new lesions into response assessment.

Target Lesions	Non-Target Lesions	New Lesions	Overall Response
Patients with Target lesions ± non target lesions			
CR	CR	No	CR
CR	Non-CR/Non-PD	No	PR
CR	Not all evaluated	No	PR
PR	Non-PD/ not all evaluated	No	PR
SD	Non-PD/ not all evaluated	No	SD
Not all evaluated	Non-PD	No	NE
PD	Any	Any	PD
Any	PD	Any	PD
Any	Any	Yes	PD
Patients with Non target lesions ONLY			
No Target	CR	No	CR
No Target	Non-CR/non-PD	No	Non-CR/ non-PD
No Target	Not all evaluated	No	NE
No Target	Unequivocal PD	Any	PD

No Target	Any	Yes	PD
Note: Patients with a global deterioration of health status requiring discontinuation of treatment without objective evidence of disease progression [or evidence of unequivocal disease progression] at that time should be reported as "*symptomatic deterioration*". This is a reason for stopping therapy, but is NOT objective PD. Every effort should be made to document the objective progression even after discontinuation of treatment.			

11.2.2.1 Frequency of Tumour Re-Evaluation

Tumor evaluation will be performed at baseline and every 8 weeks during the study treatment until disease progression. However in order to fulfil RECIST, the FIRST occurrence of response MUST be reconfirmed not earlier than 4 and not later than 5 weeks later.

Screening/baseline imaging assessments may be performed within 28 days of treatment start, on-study tumor assessments have a +/- 3 days window.

In case of discontinuation of treatment for any reason other than disease progression, patients will still be re-evaluated every 8 weeks.

11.2.3 Date of Progression

Date of progression is defined as the first day when RECIST (version 1.1) PD is observed.

11.2.4 Reporting of Tumour Response

All patients included in the study must be assessed for response to treatment, even if there is a major protocol treatment deviation or if they are ineligible, or not followed/re-evaluated. Each patient will be assigned one of the following categories: complete response, partial response, stable disease, progressive disease, early death from malignant disease, early death from toxicity, early death from other cause or unknown (not assessable, insufficient data).

Early death is defined as any death occurring before the first per protocol time point of tumour re-evaluation. The responsible investigator will decide if the cause of death is malignant disease, toxicity or other cause.

Patients for whom response is not confirmed will be classified as "unknown", unless they meet the criteria for stable disease (or the criteria for partial response in case of an unconfirmed complete

response). Patients' response will also be classified as "unknown" if insufficient data were collected to allow evaluation per these criteria.

11.3 Progression Free Survival

PFS is defined as the time from start of treatment to the first date of documented progression or death, whichever occurs first. Patients who are progression-free at the time of analysis will be censored at the date of last disease assessment.

11.4 Overall Survival

OS is defined as the time from start of treatment until the date of death due to any cause, patients still alive at the time of analysis will be censored at the date of the last visit.

12 SAFETY ASSESSMENT

All patients that received at least one dose of panitumumab will be evaluable for safety and toxicity analysis. Toxicity will be assessed using the Common Toxicity Criteria for Adverse Events version 4.03 (CTCAE). All adverse events (see section 11), up to 14 days after the last administration of study treatment, will be recorded on the case report forms; the investigator will decide if those events are drug related (not related, not likely, possibly, probably, certainly) and this decision will be recorded on the forms for all adverse events. Serious adverse events are defined by the Good Clinical Practice Guideline. Serious adverse events must be immediately reported according to the procedure detailed below.

12.1 Definitions for Adverse Event

Adverse Event (AE) is defined as any untoward medical occurrence or experience in a patient or clinical investigation subject which occurs following the administration of the trial medication regardless of the dose or causal relationship. This can include any unfavorable and unintended signs (such as rash or enlarged liver), or symptoms (such as nausea or chest pain), an abnormal laboratory finding (including blood tests, x-rays or scans) or a disease temporarily associated with the use of the protocol treatment *(ICH-GCP).*

An **Adverse Drug Reaction (ADR)** is defined as any response to a medical product, that is noxious and/or unexpected, related to any dose *(ICH-GCP).*

Response to a medicinal product (used in the above definition) means that a causal relationship between the medicinal product and the adverse event is at least a reasonable possibility, i.e. the relationship cannot be ruled out.

An **Unexpected Adverse Drug Reaction** is any adverse reaction for which the nature or severity is not consistent with the applicable product information, like the Investigators' Brochure *(ICH-GCP).*

A **Serious Adverse Event (SAE)** is defined as any undesirable experience occurring to a patient, whether or not considered related to the protocol treatment. A Serious Adverse Event (SAE) which is considered related to the protocol treatment is defined as a **Serious Adverse Drug Reaction (SADR)**.

Adverse events and adverse drug reactions which are considered as **serious** are those which result in:

- death;
- a life threatening event (i.e. the patient was at immediate risk of death at the time the reaction was observed);
- hospitalization or prolongation of hospitalization;
- persistent or significant disability/incapacity;
- a congenital anomaly/birth defect;
- any other medically important condition (i.e. important adverse reactions that are not immediately life threatening or do not result in death or hospitalization but may jeopardize the patient or may require intervention to prevent one of the other outcomes listed above).

12.2 Reporting procedures for Adverse Event

Toxicity will be scored using NCI Clinical Trials Criteria for Adverse Events (CTCAE) Version 4.03. AEs will be collected from the time the first dose of study medication is administered until 14 days following discontinuation of study medication regardless of initiation of a new cancer therapy or transfer to hospice. SAEs will be collected over the same time period as stated above for AEs. In addition, any SAE assessed as related to study participation, study medication or concomitant medication must be recorded from the time a subject consents to participate in the study up to and including any follow-up contact.

After discontinuation of study medication, the investigator will monitor all AEs/SAEs that are ongoing until resolution or stabilization of the event or until the subject is lost to follow-up.

Disease progression is not to be reported as an AE since time to progression (TTP) after Panitumumab Rechallenge is an endpoint of the study (See Section 6.2).

A death on study requires reporting regardless of causality and attribution to treatment or other cause must be provided. Death due to disease progression is not to be reported as an AE. Deaths that occur beyond 30 days after the end of study drug administration/initiation of an alternate therapy, do not qualify as SAEs.

Each adverse event is to be classified by the investigator as SERIOUS or NON-SERIOUS. This classification of the seriousness of the event determines the reporting procedures to be followed. If a serious adverse event occurs, the Istituto di Candiolo Pharmacovigilance (Fax Number: + 011 993 3261r mailbox: farmacia@ircc.it) is to be notified, using the SAE report form, within 24 hours of awareness of the event by the investigator. If the initial report is incomplete or the event is still ongoing at the time of reporting or if new significant information becomes available, this report is to be followed by submission of follow-up information within 5 calendar days after the initial notification. Reporting requirements for adverse events are summarized in the following table.

12.3 Reporting requirements for adverse event

Gravity	Reporting Time	Type of Report
**SERIOUS**	Within 24 hours from awareness by the investigator	Initial report on SAE report form + case report form
	Within 5 calendar days from initial report	Follow-up/Final report on SAE report form
**NON SERIOUS**	Per case report form submission procedure	Case report form

If for any reason the SAE form transmission is not possible, Istituto di Candiolo Pharmacovigilance should be informed by phone 011 993 3261) of the occurrence of the event. In this exceptional case, Istituto di Candiolo Pharmacovigilance will complete a SAE form with information received, which will be sent to the investigator for confirmation, and in the meanwhile Pharmacovigilance procedures will be initiated.

Istituto di Candiolo Pharmacovigilance will submit to the concerned drug company (Amgen) all SAEs occurring in this trial, regardless of whether the investigator suspects causality with the study treatment.

Serious adverse events should also be reported on the adverse event case report form. The form to be used for serious adverse event expedited reporting is not the same as the adverse event case report form, but where the same data are collected, the forms must be completed in a consistent manner. For example, the same adverse event term should be used on both forms.

Istituto di Candiolo assesses each SAE reported by the study Investigators to identify any suspected unexpected serious adverse reactions (SUSAR), i.e. serious adverse events considered at least possible associated to the study treatment by the Investigator and not listed in the IMP reference document(s) and provide this information to the sponsor for the final evaluation.

If a SAE is assessed as a possible SUSAR, the sponsor, through Istituto di Candiolo Pharmacovigilance, may urgently require further information to the Investigator. Istituto di Candiolo Pharmacovigilance will issue a SUSAR (Suspect Unexpected Serious Adverse Reaction) notification whenever appropriate, and submit it to all concerned recipients according to current law and following sponsor's indication.

Follow-up information is to be reported on a new serious adverse event form and transmitted to the same fax number as the initial report. A follow-up report is to be filled in, not only to complete the information provided on the initial report but also to modify any incorrect data.

The SAE fax delivery confirmation sheets must be retained at the study sites.

At the end of the study all original SAE report forms are to be collected by the CRO personnel and delivered to Istituto di Candiolo for archiving in the TMF, while the corresponding copies must be retained in the Investigator File.

The Sponsor shall provide Amgen with a copy of any SAE report received by the Investigators for patients treated with panitumumab. The Sponsor will send the SAE forms to Amgen Safety to the following fax number: 800916570 (in case of any issues with the fax number or for any other question the following e-mail address: *eu-it-farmacovigilanza@amgen.com* can be used).

All SAEs must be promptly forwarded to Amgen (in accordance with the local law requirements); a copy of SUSARs must be sent to Amgen at time of regulatory submission. Pregnancy and Lactation reports must be sent within 10 days of Sponsor awareness.

The Sponsor will also provide Amgen with a Development Safety Update Report (DSUR) and with a copy of any other communication or aggregate report, containing safety data generated during the course of the study, sent to the Regulatory Authorities by the Sponsor. Furthermore, the Sponsor will provide Amgen with a line listing/report of all SAEs occurred in subjects exposed to Amgen Product on a periodic basis (but no less frequently than every 6 months) in order to fulfill the reconciliation process.
The final study report should be sent to Amgen no later than 1 calendar year of study completion.

12.4 Reporting requirements for complaints related to panitumumab

All product complaints relating to Panitumumab will be reported directly to Amgen at the time of discovering the complaint. Such complaints include:

- packaging (eg, broken container or cracked container)
- function (eg, subject or healthcare provider cannot appropriately use the product despite training, [eg, due to malfunction of the auto-injector (AI)/Pen or Personal Injector])
- labeling (eg, missing labels, illegible labels, incorrect labels, and/or suspect labels)
- change in IP appearance (eg, color change or visible presence of foreign material)
- unexpected quantity or volume (eg, number of tablets or amount of fluid in bottle/vial)
- evidence of tampering or stolen material'.

Reports must be made to AMGEN using the "Product Complaint Form".

12.5 Recording Adverse Events in the Case Report Forms

AEs can be assessed directly by the Investigator during a clinical visit or based on laboratory/Instrumental examinations or can be referred by the patient.

12.5.1 Pre-existing Conditions

A pre-existing condition (i.e., a disorder starting before the adverse event reporting period) should not be reported as an adverse event unless the condition worsens during the adverse event reporting period.

12.5.2 Procedures

Diagnostic and therapeutic procedures, such as surgery, should not be reported as adverse events, while the medical condition for which the procedure was performed should be reported if it meets the definition of an adverse event. For example, an appendectomy performed for an acute appendicitis occurring during the adverse event reporting period should not be reported as adverse event; while "acute appendicitis" is to be reported as adverse event. If a patient undergoes a surgical procedure that was planned prior to entry into the trial, and surgery is not performed due to a worsening of a baseline condition, this baseline condition should not be reported as an adverse event.

12.5.3 Symptoms of Targeted Disease

Tumor-related signs and symptoms will be followed at each visit. Although a measure of efficacy, these will always be reported as pre-existing conditions at baseline and during treatment only if they meet the definition of adverse event.

For all adverse events the Investigator will be asked to assess its relationship with the study treatment.

12.5.4 Causality Assessment and Grading of Adverse Event Severity

The assessment of relationship to study drug will be done according to the following causality scale based on the WHO definitions:

- Certain: A clinical event, including laboratory test abnormality, occurring in a plausible time relationship to drug administration, and which cannot be explained by concurrent disease or other drugs or chemicals. The response to withdrawal of the drug (de-challenge) should be clinically plausible. The event must be definitive pharmacologically or phenomenologically, using a satisfactory re-challenge procedure if necessary
- Probable: A clinical event, including laboratory test abnormality, with a reasonable time sequence to administration of the drug, unlikely to be attributed to concurrent disease or other drugs or chemicals, and which follows a clinically reasonable response on withdrawal (rechallenge). Rechallenge information is not required to fulfil this definition.
- Possible: A clinical event, laboratory test abnormality, with a reasonable time sequence to administration of the drug, but which could also be explained by concurrent disease or other drugs or chemicals. Information on drug withdrawal may be lacking or unclear

- Unlikely: A clinical event, laboratory test abnormality, with a temporal relationship to drug administration which makes a causal relationship improbable, and in which other drugs, chemicals or underlying disease provide plausible explanations Severity grading of adverse events and pre-existing conditions will be done according to the National Cancer Institute (NCI) Common Toxicity Criteria (CTCAE) V. 4.03.

AEs that are not defined in the NCI CTCAE should be evaluated for severity according to the following scale:

- Grade 1 = Mild – transient or mild discomfort; no limitation in activity; no medical intervention/therapy required;
- Grade 2 = Moderate – mild to moderate limitation in activity, some assistance may be needed; no or minimal medical intervention/therapy required;
- Grade 3 = Severe – marked limitation in activity, some assistance usually required; medical intervention/therapy required, hospitalization is possible;
- Grade 4 = Life threatening – extreme limitation in activity, significant assistance required; significant medical intervention/therapy required, hospitalization or hospice care probable;
- Grade 5 = Death - the event results in death.

Note the distinction between the gravity and the severity of an adverse event. Severe is a measure of intensity; thus, a severe reaction is not necessarily a serious reaction. For example, a headache may be severe in intensity but would not be classified as serious unless it meets one of the criteria for serious events listed above.

12.5.5 Pregnancy Reporting

Any pregnancy that occurs during study participation must be reported using the “exposure in utero” form. To ensure subject safety, each pregnancy must be reported to Istituto di Candiolo Pharmacovigilance within 2 weeks of learning of its occurrence. The pregnancy must be followed-up to determine outcome (including premature termination) and status of mother and child. Pregnancy complications and elective terminations for medical reasons must be reported as an AE or SAE. Spontaneous abortions must be reported as a SAE.

Any SAE occurring in association with a pregnancy brought to the investigator’s attention after the subject has completed the study and considered by the investigator as **possibly related** to study treatment, must be promptly reported to Istituto di Candiolo Pharmacovigilance.

In addition, the investigator must attempt to collect pregnancy information on any female partners of male study subjects who become pregnant while the subject is enrolled in the study. Pregnancy information must be reported to Istituto di Candiolo Pharmacovigilance as described above.

12.5.6 Overdose

Reporting if any overdose (accidental or intentional) which results in serious adverse reactions is to be handled following the SAE procedures. This includes reports related to drug intake with suicidal intentions and consequent drug overdose.

Overdose reporting even not associated with adverse reactions shall be anyhow reported immediately to Istituto di Candiolo Pharmacovigilance using the most rapid type of communication (phone, e-mail).

12.5.7 Follow-up of Unresolved Adverse Events

All adverse events should be followed at least until 28 days following the last dose of IMP. Drug-related and serious adverse events ongoing at the end of this observation period must be recorded until they are resolved or the investigator assesses them as chronic or the subject is lost to follow-up or starts a new anti-cancer treatment, whichever occurs earlier.

13 TRANSLATIONAL ASSESSMENT

Patients are liquid biopsied at two checkpoints: BML (at end of first anti-EGFR PD, optional), and RML (mandatory), and during Trial Phase on day 1 and 15 of each cycle until progression.

Specific Standard Operating Procedures will be used according to our Liquid Biopsy Guidelines (See Appendix C).

Isolated circulating free DNA is analyzed using ddPCR™(Bio-Rad) or by Next Generation Sequencing using Illumina platforms as previously described. Next generation DNA sequencing based on Illumina reagents is widely used and considered highly reliable. The NGS LB approaches we developed are based on Illumina reagents.

The collection information must be captured on the Biomarker Assessment eCRF pages and Central Lab Requisition forms.

The expanded RAS molecular status will be analyzed by plasma ddPCR to investigate how the RAS profile is affected (if any) by panitumumab rechallenge. The mutation load and the duration of mutation free plasma (if any), will be measured.

The molecular landscape of plasma ctDNA will be defined by NGS at RML and in the LB at progression of all patients participating to the Trial Phase.

14 STATISTICAL CONSIDERATIONS

14.1 Sample Size

We used the A'Hern one-stage approach to calculate the sample size. For the primary objective, we will need to enroll 27 patients in order to achieve a power of at least 85% to test the null hypothesis that the rate of response to panitumumab would be 10% or less, versus the alternative hypothesis that the response rate would be 30% or more, at a one-sided alpha level of 0.05. Six objective responses are necessary to declare the study positive.

Protocol amendment number 2 restricts, while simplifying the eligibility criteria to patients with a negative liquid biopsy for extended RAS/RAF and EGFR ectodomain mutations, while eligibility in the prior version required a reduction of at least 50% of extended RAS/RAF positive clones. The expected Panitunumab rechallenge ORR remains however the same i.e. 30%, requiring under the same alfa and beta assumption a sample size of 27 patients.

27 patients need to be recruited in the amended protocol v3.0. Patients resulting positive to the Panel B genotypization will be analyzed by the intention-to-treat (ITT) approach and will be included in the efficacy analysis population (see statistical analysis paragraph 14.2).

In the previous protocol v. 2.1 dated 30.10.2017, four patients were enrolled with the previous criteria. All these patients left the trial for progression to panitumumab rechallenge at the first tumor assessment. These patients will be included in the Safety Evaluation Population. Therefore, at the end of the study 31 patients will have received the rechallenge with panitumumab.

14.2 Statistical Analysis

14.2.1 Analysis Populations

Screening Phase. All patients signing the informed consent will be registered. The number of patients who will not be eligible will be recorded together with the reasons for non-eligibility.

The number of patients who died or withdrew before trial entry, will be specified.

Two populations will be considered for the analysis, as follows:

- The Safety Evaluable (SE) population defined as all treated patients (i.e. eligible as decided at the time of registration that receives at least 1 dose of study treatment). An incorrect treatment schedule or drug administration or an early termination of treatment does not result in exclusion of patients from this population. This population will be the object of the Safety and Tolerability analysis. Patients with major deviations from the eligibility criteria affecting safety or from the treatment schedule at cycle 1 for reasons other than toxicity may be presented in separate tables/listings. In this population are included the four patients already enrolled with protocol criteria v.2.1 and the 27 patients to be enrolled with protocol v.3.0 criteria.
- The Efficacy Evaluable (EE) population defined as all treated patients, with no major deviations from the eligibility criteria affecting efficacy evaluation, for which the tumor response could be evaluated at least once while on treatment. These patients should have received at least 2 cycles after treatment starts, unless disease progression occurs within this period. Patient with tissue DNA alteration (see also section 8.3.2 and appendix B) will be analyzed by the intention-to-treat (ITT) approach and will be included in the Efficacy Evaluable Population. A patient will be considered to be Trial eligible if he/she did not have any deviation from the patient entry criteria listed in sections 8.1 and 8.2 Potential eligibility problems will be assessed by the Study Coordinator at time of medical review. The final efficacy analysis will occur after 27 patients are evaluable for response according to RECIST 1.1. The distribution of follow-up time will be described and the number of patients lost to follow-up will be reported.

Conclusions will be based on all eligible patients. Further analysis may be performed excluding those patients for whom major protocol deviations have been identified (e.g., early death due to other reasons, early discontinuation of treatment, major protocol violations, etc.). However, these sub-analyses may not serve as the basis for drawing conclusions concerning treatment efficacy, and the reasons for excluding patients from the analysis should be clearly reported.

14.2.2 Statistical Methods

Binary variable such as ORR will be reported as proportions with 95% confidence interval (from the exact binomial distribution). Adverse events will be reported as proportions.

Time-to-event variables such as PFS and OS will be estimated using the Kaplan-Meier product-limit method. The full curve will be included in the report. Estimates at weekly intervals will be tabulated with their standard error; medians will be provided with 95% confidence interval. In case of explorative subgroup analysis to determine a potential association between the different (if any) RAS-extended mutant clones in plasma and response to panitumumab, we will use the log-rank test to estimate the significance of the comparison. Results will be considered significant when P values are 0.05 or less; Fisher's exact test will be used for subgroup comparisons of categorical variables. For the translational exploratory objectives, receiver operating characteristic (ROC) curve analysis might be used to assess whether fractional abundance of the mutational load is a potential classifier. In case, to quantify the discriminatory accuracy of the mutational as a predictor of efficacy outcomes, we will use Harrell C statistics Area under the curve analysis and a recursive partitioning method for detecting the best cut-off points[17]. Analyses will be performed with the use of SAS statistical software, version 9.2 (SAS Institute) and Stata version 12.

15 QUALITY CONTROL AND QUALITY ASSURANCE

15.1 Monitoring

Monitoring visits to the trial site will be made periodically during the trial by a qualified monitor to verify that the trial is conducted according to study protocol, GCP principles and regulatory requirements. The monitor will verify the accurate and complete recording of data on CRFs, source documents, Investigators File and drug accountability records.

The investigator/institution guarantees direct access to source documents of the study patients and to any other trial related documentation.

It is important that the investigator(s) and/or their relevant personnel are available during the monitoring visits.

15.2 Auditing

Representative members of Sponsor Quality Assurance may conduct an on-site audit. The investigator will be informed if an audit is to take place.

Representative of Regulatory Agencies may also conduct an inspection of the study. If informed of such an inspection, the Investigator should notify Sponsor immediately. The investigator will ensure that the auditors/ inspectors have access to the clinical supply, study site facilities, source documents and all study files.

16 DATA HANDLING AND RECORD KEEPING

16.1 Case Report Form (CRF)

An electronic Case Report Form will be completed for each enrolled subject. The language used must be English. The completed original Case Report Forms are the sole property of Sponsor and should not be made available in any form to third parties, except for authorized representatives of appropriate regulatory authorities, without written permission from Sponsor.

The Investigator or an authorized staff member (medically qualified) has the responsibility to ensure completion and to review and sign all Case Report Forms.

However, the Investigator has final personal responsibility for the accuracy and authenticity of all clinical and laboratory data entered on the Case Report Form.

Subject source documents are the hospital subject records maintained at the study site. In case where the source documents are the hospital chart, the information collected on the Case Report Form must match with those charts. In some case a portion of the source documents are not the hospital subject records. The investigator and Sponsor must agree which items will be recorded in the source documents and for which items the Case Report Form will stand as the source document. This must be stated in the "Data Location List" (filed in the Investigator File). One copy of this document should be remitted to the sponsor or CRO delegated by the sponsor (CD Pharma, Milano) for filing into the Trial Master File.

16.2 Data Handling

Data Management will be carried out by external CRO (CD Pharma, Milano). Medical terms are coded according to the MedDRA dictionary. Data will be analyzed using SAS® System currently used at CRO (TBD). Data cleaning will include both visual and computer-driven procedures in order to minimize logical inconsistencies and errors within the collected data. The data are checked for completeness, accuracy and consistency. The errors detected will be rectified by means of Data Clarification List (DCL) that will be used by the monitor for resolution of queries. The original DCL must be kept together with the patient CRF.

16.3 Record Retention

To enable evaluation and/or audits and/or regulatory authorities inspections, the Investigator agrees to keep records, including the identity of all participating subjects ("Subject identification Log"), all original signed informed consent forms, copies of all case report forms, source documents, detailed records of treatment disposition as well as the documentation included in the Investigator File according to local regulations or as specified in the Clinical Trial Agreement.

If the Investigator relocates, retires, or for any reason withdraws from the study, Sponsor should be prospectively notified. The study records must be transferred to an acceptable designee, such as another investigator, another institution, or to CRO (CD Pharma, Milano). The investigator must obtain Sponsor's written permission before disposing of any records.

17 ETHICAL CONSIDERATIONS

17.1 Institutional Review Board (IRB)/ Independent Ethics Committee (IEC) and Competent Authority (CA)

Before initiating the trial, the Investigator should have written favourable opinion from the IRB/IEC and CA for the trial conduction. All the correspondence with the IRB/IEC and CA should be retained in the Investigator File.

Before implementing any protocol amendment, the IRB/IRC/CA written approval must be obtained. The only circumstance in which an amendment may be initiated prior to IRB/IEC approval is where the change is necessary to eliminate apparent immediate hazards to the subjects. In that event, the IRB/IEC/CA must be notified in writing *asap*.

It is responsibility of Sponsor to provide the Investigator with the Health Authority approval where needed to implement a trial.

17.2 Ethical conduct of the trial

The trial will be performed in accordance with International Conference on Harmonization Good Clinical Practice guidelines, the Declaration of Helsinki and applicable local regulatory requirements and laws.

17.3 Informed Consent

It is the responsibility of the investigator to give each patient full and adequate verbal and written information regarding the objective and procedures of the trial and the possible risks involved. The patient must be informed about his/her right to withdraw from trial at any time. The patient should have time and opportunity to enquire about details of the trial and to decide whether or not to participate in the trial.

Written subject information must be approved by IRB/IEC and CA and must be given to each patient before any trial-related procedure is undertaken.

It is responsibility of the investigator to obtain informed consent signed and dated by the patient and by the medical person conducting the informed consent discussion, prior to undertaken any trial-related procedure. One copy of the signed and dated Informed Consent Form should be given to the

patient. The originally signed document should be archived in the confidential section of the Investigator File.

The approved patient information sheet must not be changed without prior approval by Sponsor and by the IRB/IEC and CA.

When new study information arises during the study, the patients still on treatment must be informed and a new Informed Consent form or an addendum to the already signed Informed Consent form must be signed and dated by the patients.

If a patient becomes incompetent during the course of a trial where it was not anticipated, legally acceptable representative authorization should be obtained for a subject's continued participation.

18 LIABILITY AND INSURANCE

The involved parties will be insured in accordance with the applicable laws and regulation for injuries and/or damages that may arise as a consequence of this trial.

19 CONFIDENTIALITY OF INFORMATION AND PUBLICATION OF RESULTS

Sponsor assures that the key design element of this protocol will be posted in a publicly accessible database such as clinicaltrial.gov; in addition, upon study completion, the results of this study will be submitted for publication and posted in publicly accessible database for clinical trial studies.

All information regarding study drug supplied by Sponsor to the investigator is privileged and confidential information. The investigator agrees to use this information to accomplish the study and will not use it for other purposes without consent from Sponsor.

It is understood that there is an obligation to provide Sponsor with complete data obtained during the study. The investigator agrees to keep in confidence all the results obtained from the study. Such information shall not be disclosed to third parties without prior written permission from Sponsor, except to regulatory authority(ies), when requested.

Individual investigators may present results of the study at scientific meetings. However prior to the submission, the Sponsor will have the opportunity to review and comment the abstracts for a period of up to 15 calendar days prior to the submission.

20 FIGURES

20.1 Figure 1 Mutated KRAS alleles emerge in circulating DNA during anti-EGFR therapy and decline when treatment is suspended

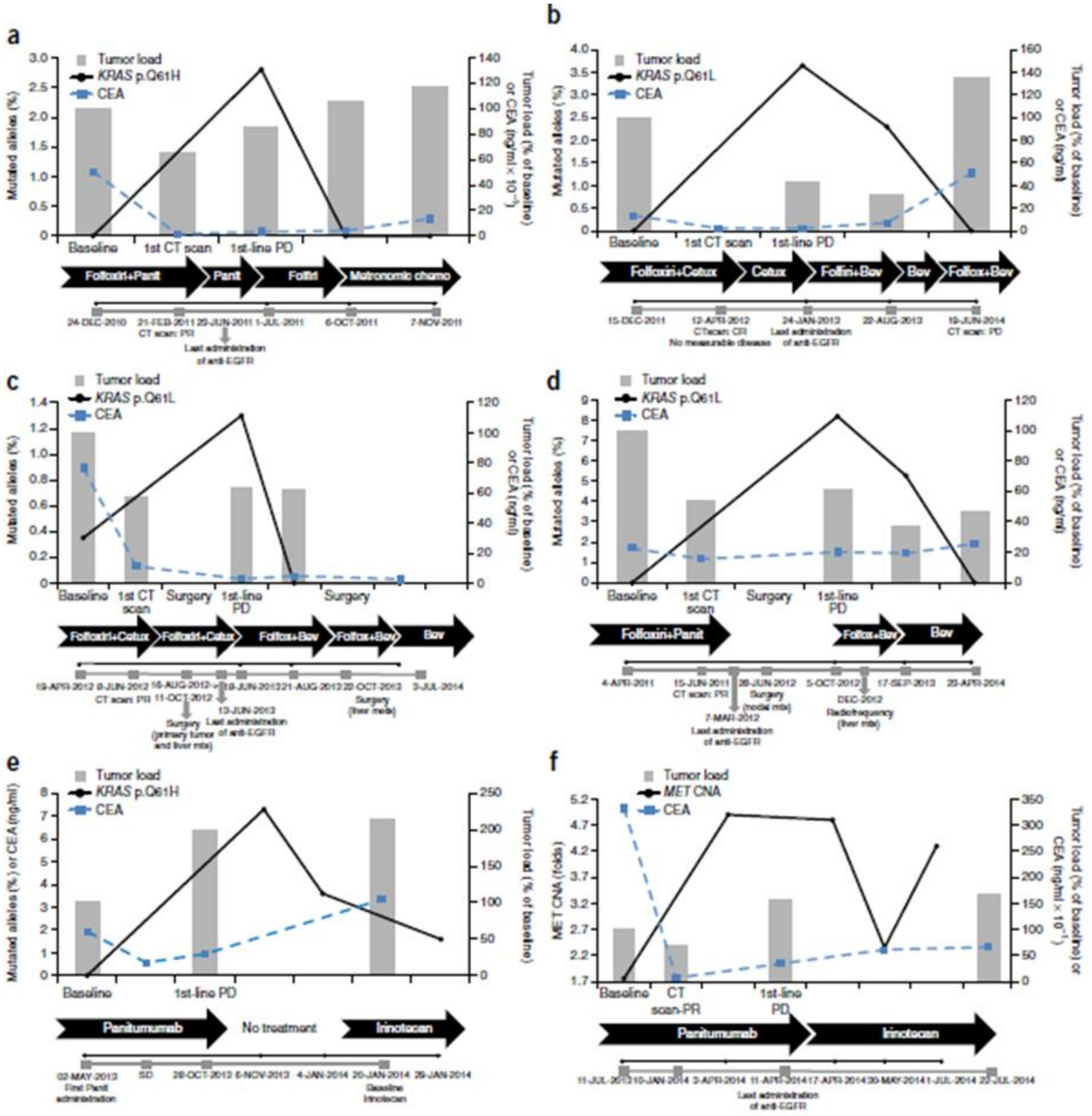

(a–f) Detection of KRAS mutations in mCRC patients AOUP-CRC04 (a), AOUP-CRC01 (b), AOUP-CRC06 (c), AOUP-CRC03 (d), ONCG-CRC71 (e) and MET amplification in patient ONCG-CRC72 (f) in circulating DNA of patients who developed acquired resistance to first-line chemotherapy plus anti-EGFR treatment and then received other lines of treatment. Gray bars represent the variation of tumor load, compared to baseline, during systemic treatments specified in arrows below the graphs. Tumor load is calculated as follows: measurable disease at the initiation of treatment (baseline) is assumed as 100%; responses or progression are calculated as the percentage of tumor load compared to baseline, as per Response Evaluation Criteria in Solid Tumors (RECIST) criteria. Relevant clinical events are indicated in gray boxes below the graphs. Black lines indicate the frequency of

KRAS mutation (percentage of alleles) or MET copy number alteration, detected in circulating DNA at the time points indicated below the graphs. Dotted blue line indicates CEA (carcinoembryonic antigen) values. PD, progressive disease; Cetux, cetuximab; Panit, panitumumab; Bev, bevacizumab; Irino, irinotecan; Folfoxiri, folinic acid, 5-fluorouracil, oxaliplatin and irinotecan; Folfiri, folinic acid, 5-fluorouracil and irinotecan; Folfox, folinic acid, fluorouracil and oxaliplatin.

20.2 Figure 2 Re-challenge with EGFR specific antibodies in CRC cells and patients

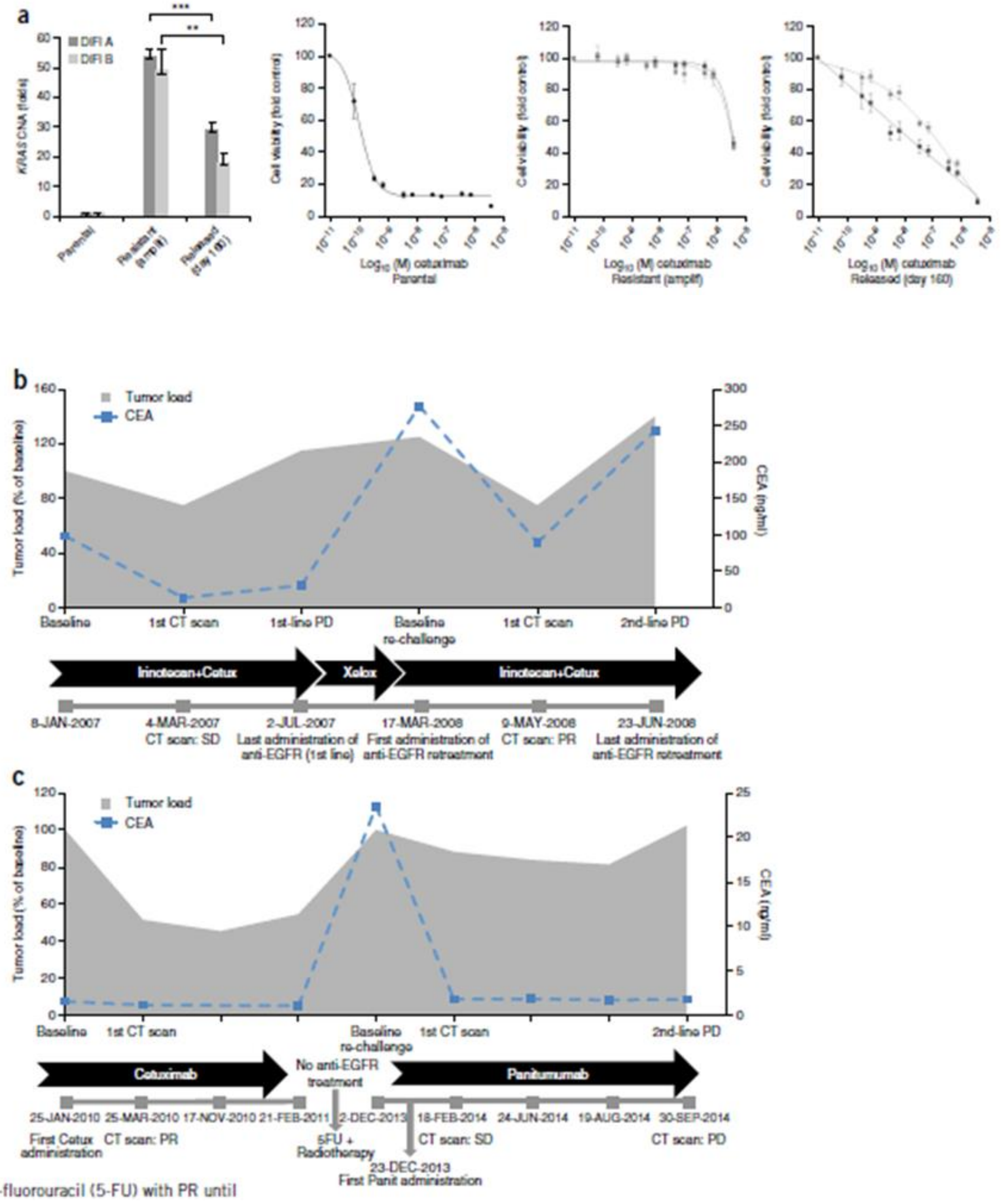

(a) Two CRC cell populations (DiFi A and DiFi B) that developed KRAS amplification as a resistance mechanism to cetuximab were allowed to replicate in the absence of the antibody for 160 d. Top, KRAS amplification assessed by qPCR in the indicated cell models (parental/sensitive, resistant derivatives and resistant cells after 160 d of antibody withdrawal). Gray bars indicate KRAS gene copy number alterations. Statistical differences were

calculated by Student's t-test. Data are expressed as means ± s.d. of three independent experiments. ***P ≤ 0.001; **P ≤ 0.01. Bottom, cetuximab sensitivity assay. Data points represent means ± s.d. of three independent experiments.
(b) Clinical synopsis of mCRC patient HMAR-CRC07 treated with irinotecan plus cetuximab achieving stable disease (SD) for approximately 6 months. At progression, the patient received capecitabine plus oxaliplatin (Xelox) with further progression of the disease after 3 months. The patient was subsequently re-treated with irinotecan plus cetuximab, achieving a partial response (PR). Gray area represents tumor load (percentage of baseline, calculated as described in Fig. 1 legend); dotted blue line indicates CEA (carcinoembryonic antigen) values.
(c) Clinical synopsis of mCRC patient ONCG-CRC74, who was treated with cetuximab as a third-line therapy, achieving a partial response that lasted 13 months; the patient then refused further therapy because of skin toxicity. At disease progression, the subject underwent radiotherapy and treatment with 5-fluorouracil (5-FU) with PR until progression occurred after 6 months. The patient was re-challenged with anti-EGFR treatment, achieving long-lasting stable disease (7 months). Gray area represents tumor load (percentage of baseline, calculated as described in Fig 1 legend); dotted blue line indicates CEA values. Cetux, cetuximab.

20.3 Figure 3 Study Flow Chart

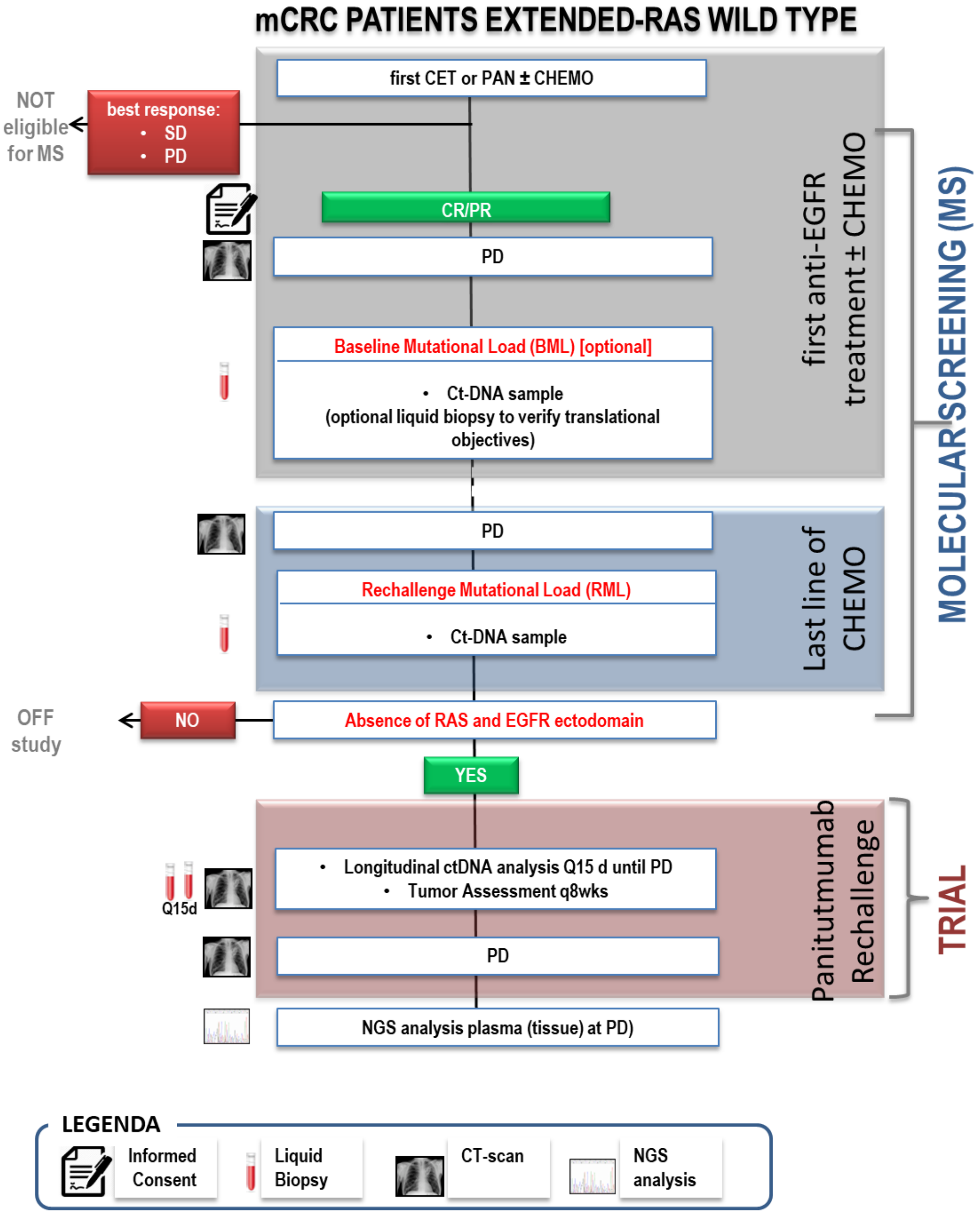

21 APPENDIX

21.1 Appendix A: References

1 Siegel, R., Naishadham, D. & Jemal, A. Cancer statistics, 2013. *CA Cancer J Clin* **63**, 11-30, doi:10.3322/caac.21166 (2013).

2 Heinemann, V. *et al.* FOLFIRI plus cetuximab versus FOLFIRI plus bevacizumab as first-line treatment for patients with metastatic colorectal cancer (FIRE-3): a randomised, open-label, phase 3 trial. *Lancet Oncol* **15**, 1065-1075, doi:10.1016/S1470-2045(14)70330-4 (2014).

3 Fakih, M. G. Metastatic colorectal cancer: current state and future directions. *J Clin Oncol* **33**, 1809-1824, doi:10.1200/JCO.2014.59.7633 (2015).

4 Siena, S., Sartore-Bianchi, A., Di Nicolantonio, F., Balfour, J. & Bardelli, A. Biomarkers predicting clinical outcome of epidermal growth factor receptor-targeted therapy in metastatic colorectal cancer. *J Natl Cancer Inst* **101**, 1308-1324, doi:10.1093/jnci/djp280 (2009).

5 Misale, S., Di Nicolantonio, F., Sartore-Bianchi, A., Siena, S. & Bardelli, A. Resistance to anti-EGFR therapy in colorectal cancer: from heterogeneity to convergent evolution. *Cancer Discov* **4**, 1269-1280, doi:10.1158/2159-8290.CD-14-0462 (2014).

6 Arena, S. *et al.* Aurora kinase A gene copy number is associated with the malignant transformation of colorectal adenomas but not with the serrated neoplasia progression. *Clin Cancer Res.* **21**, 2157-2166. doi: 2110.1158/1078-0432.CCR-2114-2821. Epub 2015 Jan 2126. (2015).

7 Misale, S. *et al.* Emergence of KRAS mutations and acquired resistance to anti-EGFR therapy in colorectal cancer. *Nature* **486**, 532-536, doi:10.1038/nature11156 (2012).

8 Diaz, L. A. *et al.* The molecular evolution of acquired resistance to targeted EGFR blockade in colorectal cancers. *Nature* **486**, 537-540, doi:10.1038/nature11219 (2012).

9 Siravegna, G. *et al.* in *Nat Med* Vol. 21 827 (2015).

10 Arena, S. *et al.* Emergence of Multiple EGFR Extracellular Mutations during Cetuximab Treatment in Colorectal Cancer. *Clin Cancer Res* **21**, 2157-2166, doi:10.1158/1078-0432.ccr-14-2821 (2015).

11 Santini, D. *et al.* Cetuximab rechallenge in metastatic colorectal cancer patients: how to come away from acquired resistance? *Ann Oncol*, doi:10.1093/annonc/mdw551 (2017).

12 Pietrantonio, F. *et al.* Single agent panitumumab in KRAS wild-type metastatic colorectal cancer patients following cetuximab-based regimens: Clinical outcome and biomarkers of efficacy. *Cancer Biol Ther* **14**, 1098-1103, doi:10.4161/cbt.26343 (2013).

13 Arena, S. *et al.* Emergence of Multiple EGFR Extracellular Mutations during Cetuximab Treatment in Colorectal Cancer. *Clin Cancer Res* **21**, 2157-2166, doi:10.1158/1078-0432.CCR-14-2821 (2015).

14 Van Emburgh, B. O. *et al.* Acquired RAS or EGFR mutations and duration of response to EGFR blockade in colorectal cancer. *Nat Commun* **7**, 13665, doi:10.1038/ncomms13665 (2016).

15 Parseghian, C. M. *et al.* Anti-EGFR Resistant Clones Decay Exponentially After Progression: Implications for Anti-EGFR Re-challenge. *Ann Oncol*, doi:10.1093/annonc/mdy509 (2018).

16 A'Hern, R. P. Sample size tables for exact single-stage phase II designs. *Stat Med* **20**, 859-866, doi:10.1002/sim.721 (2001).

17 Harrell, F. E., Lee, K. L. & Mark, D. B. Multivariable prognostic models: issues in developing models, evaluating assumptions and adequacy, and measuring and reducing errors. *Stat Med* **15**, 361-387, doi:10.1002/(SICI)1097-0258(19960229)15:4<361::AID-SIM168>3.0.CO;2-4 (1996).

21.2 Appendix B: minimum set of genes to be tested on tissue FFPE

If not already performed, patients will be tested for negativity to the following minimum set of gene mutations and CNV within 3 months from the treatment C1D1. Genotyping can be performed trough any multigene platform (i.e. Foundation Medicine platform, Personal Genomic Diagnostic platform, MSK-IMPACT platform) at diagnosis of metastatic disease, during study screening or within 3 months from treatment C1D1.

Negativity to this panel is defined as the absence of any DNA mutation and no CNV ≥ 10 in the following loci/genes.

Genotyping can be perfomed on archival tumour samples (not older than 1 year) or on a freshly obtained tumor biopsy. Participating center that cannot perform Genotyping will send FFPE to Istituto di Candiolo-IRCCS for centralized testing.

CHRONOS minimum panel of genes for multiplex screening

GENE	MUTATION FOR SCREENING (SEQUENOM PLATFORM)
BRAF	G464R, G464V/E, G466R, F468C, G469S, G469E, G469A, G469V, G469R, D594G, D594V, V600E (1799T>A), V600K, V600R (1798_1799GT>AG), V600M, V600Q, V600E (1799_1800TG>AA) / V600D (1799_1800TG>AC/1799_1800TG>AT), V600A, V600G, V600L (1798G>C), V600R (1797_1799AGT>GAG), V600>YM, V600_K601>E, K601E,
EGFR	S492R, G465E/R R108K, T263P, A289V, G598V, E709K/H, E709A/G/V, G719S/C, G719A, M766_A767insAI, S768I, V769_D770insASV, V769_D770insCV, D770_N771>AGG/V769_D770insASV, D770_N771insG, N771_P772>SVDNR, P772_H773insV, H773>NPY, H773_V774insNPH/PH/H, V774_C775insHV, T790M, L858R, L861Q, E746_T751del, E746_A750del, S752D, L747_E749del, L747_T750del, L747_T751del, L747_S752del, P753S, A750P, T751A, T751P, T751I, S752I/F, S752_I759del, L747_Q ins, E746_T751del, I ins (combined), E746_A750del, T751A (combined), L747_E749del, A750P (combined), L747_T750del, P ins (combined), L747_S752del, Q ins (combined)
HER2	L866M, V777L, S310Y
IDH1	R132C
KRAS	G12D (35G>A), G12V (35G>T), G12C (34G>T) , G12A , G12S , G12R (34G>C), G12F, G12L, G12I, G12fs*3, G12E (35_36GT>AA/35_36GT>AG), G12Y, G12N, G13D (38G>A), G13C (37G>T), G13S, G13R, G13A, G13V (38G>T), A59E, A59G, A59T, A59S, A59P, A59V, Q61H (183A>C), Q61H (183A>T), Q61L, Q61R (182A>G), Q61K (181C>A), Q61P, Q61E, K117N (351A>C), K117N (351A>T), K117Q, K117E, K117R, K117T, K117I, A146T, A146P, A146V, A146G, A146S, A146E,
MEK1 (MAP2K1)	K57N
NRAS	G12V/A/D, G12C/R/S, G13V/A/D, G13C/R/S, A18T, Q61L/R/P, Q61H, Q61E/K
PIK3A	R88Q, N345K, C420R, P539R, E542K, E545K, Q546K, H701P, H1047R/L, H1047Y, R38H, C901F, M1043I

GENES FOR CNV (NANOSTRING PLATFORM)
CRAF(RAF1)
EGFR
FGFR1
FGFR2
FGFR3
HER2
IGF1
IGF1R
IGF2
KRAS
MET
NF1 (deletion)

21.3 Appendix C: ddPCR Panel for Molecular Screening

ddPCR Panel for Molecular Screening

Gene	***KRAS***													
Mutation	G12A	G12C	G12D	G12R	G12S	G12V	G13D	Q61K	Q61L	Q61R	Q61H (A>T)	Q61H (A>C)	Q61P	Q61E
Gene	***NRAS***													**BRAF**
Mutation	G12A	G12C	G12D	G12R	G12S	G12V	G13D	Q61K	Q61L	Q61R	Q61H (A>T)	Q61H (A>C)		V600E
Gene	***EGFR***													
Mutation	S492R (C>A)	G465R (G>A)	G465R (G>C)	G465E (G>A)	S464L (C>T)	V441G (T>G)	V441D (T>A)							

21.4 Appendix D: Liquid Biopsy Guidelines for Molecular Screening Phase

Health and safety

In accordance with the site's policies and guidelines, use personal protective equipment to prevent exposure to blood borne pathogens or other potentially infectious materials, and dispose of all clinical waste appropriately. Before starting to work under this protocol, all staff should review the guidelines for working with blood borne pathogens and have been vaccinated.

Materials and Equipment

Cell-Free DNA BCT® Streck Tubes (Streck, catalogue number 218997) will be used to collect blood samples. Streck Tubes must be stored at controlled room temperature (6°-30°C).

21.5 Appendix E: Liquid Biopsy Guidelines for Trial Phase

These instructions describe how to collect whole blood samples and prepare plasma for the isolation of cell-free circulating tumor DNA (step 1)

PBMC isolation guidelines from the same blood samples are described below (step 2).

Health and safety

In accordance with the site's policies and guidelines, use personal protective equipment to prevent exposure to blood borne pathogens or other potentially infectious materials, and dispose of all clinical waste appropriately. Before starting to work under this protocol, all staff should review the guidelines for working with blood borne pathogens and have been vaccinated.

All steps to be carried out under a laminar flow sterile hood.

Materials and Equipment for step 1

- 10 ml K2-EDTA Vacutainer (Becton Dickinson, # 366643 or equivalent)
- Vacutainer needles or butterfly needles, 20G/21G (Becton Dickinson, #367344/364815 or equivalent)
- 1.5ml APEX Screw-Cap Microcentrifuge Tube, Conical, Standard Cap, Sterile #APCP5931
- 10 ml and 5 ml serological disposable pipettes (Corning, # 4487 or #4488 or equivalent)
- 15 ml polypropylene centrifuge tubes (Fisher, # 3208303 or equivalent)

- Freezer storage boxes for 2 ml cryogenic vials (Fisher, # 3468196 or equivalent)
- Centrifuge, capable of ~3000 g with a swing bucket rotor (e.g. Eppendorf, 5702; # 5702 000.019 or equivalent)
- Pipetting aid (e.g. Eppendorf, Easypet; #4421 000.013 or equivalent)
- Ultra-Low Temperature Freezer (e.g. Thermo Electron, Revco Ultima PLUS; ULT1786-10 or equivalent)

Materials and Equipment for step 2

- Lysis buffer 10X: (NH4Cl 1,55M, KHCO3 100mM, EDTA 10mM)
- H2O infusion grade.
- Freezing mix: 80% FCS + 20% DMSO
- Turk Staining
- Centrifuge tube 15 and 50mL
- Serological pipette, micropipette and pipetting aid.
- Burker chamber

Lysis Buffer recipe

NH_4Cl 1,55M	= 41,45gr
$KHCO_3$ 100mM	= 5gr
EDTA 10mM	= 1,85gr
H_20	to 500ml

Procedure

Blood Draw (in Clinic)

1. Confirm subject's ID and write subject's name or subject ID and DOB on the sample sheet and EDTA tube.
2. Prepare subject for blood draw.
3. Obtain venous blood (~1 to 2 times10 ml) by any standard phlebotomy technique from a peripheral access point or from a central line by trained personnel into EDTA tubes.
4. For special instructions, see EDTA tube product information.
5. Gently invert tubes about 10 times immediately after collection.
6. Record date and time of blood draw on samples sheet and EDTA tube.
7. Prepare sample for the transportation to the laboratory or processing side.

Time between blood collection and plasma/blood cell processing is recommended to be <4-5 h. Experiments have shown that extended storage at room temperature ≥5h can affect the detection of cell-free circulating tumor DNA in plasma.

STEP 1

Plasma Processing (in Laboratory)

8. Upon arrival in the laboratory, centrifuge EDTA tubes at room temperature for 10 min at 1600 (±150) g. If centrifuge uses rpm (revolutions per minute), see centrifuge instructions for the conversion.
9. Ensure that brake switch is off in order to prevent disruption of the cell layer.
10. Record plasma processing start time at start of first centrifugation.
11. After centrifugation remove tubes from centrifuge.
12. Transfer supernatant of the two EDTA tubes to one fresh 15 ml centrifuge tube without disturbing the cellular layer using a disposable 10 ml serological pipette or disposable bulb pipette. Do not discard the EDTA tubes, as they will be used for PBMC extraction described in step 2.

Centrifugation separates plasma from leukocytes and erythrocytes as shown in the figure below (left). Leaving sufficient residual plasma in the tubes after the centrifugation and not disturbing the leukocyte layer (see image) when pipetting is a critical step in the sample preparation process (right). Be careful not to disturb leukocyte layer in the tubes.

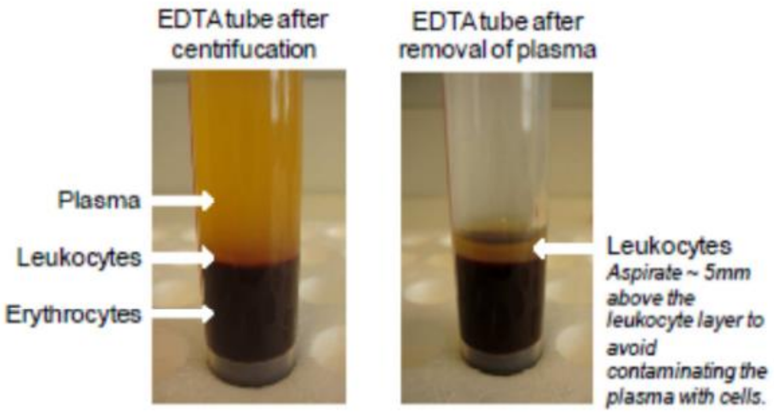

13. Centrifuge the plasma in the 15 ml centrifuge tube at room temperature for 10 min at 3000 (±150) g.

14. After centrifugation remove tubes from centrifuge.
15. Transfer supernatant to a fresh 15 ml centrifuge tube without disturbing the cellular layer using a disposable 5 ml or 10 ml serological pipette or disposable bulb pipette.

The 2nd centrifugation is intended to remove any residual intact blood cells carried over from the 1st centrifugation step. Tests have shown that speeds slower than 3000g do not completely remove blood cells from the supernatant. Centrifugation speeds higher than 3000 g are preferred.

16. Leave a residual volume of about 0.3 ml (~7 mm) on the bottom of the 15 ml tube to avoid contaminating the plasma with cells (see image).

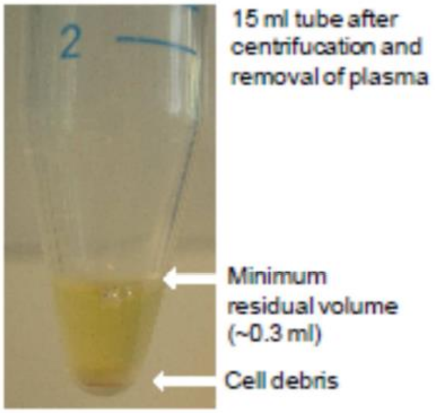

17. After transferring the plasma to a new 15 ml centrifuge tube as described, gently mix plasma and record total plasma volume (~8-10 ml plasma per 20 ml blood).
18. Transfer 1 ml plasma aliquots with a pipette to 1.5ml APEX Screw-Cap Microcentrifuge Tube
19. Place plasma tubes into storage box and freeze plasma in freezer upright in storage box at -70°C or colder.
20. Short time storage at -20°C is possible.

STEP 2

PBMC extraction

**Start from EDTA tubes of step 1 (point 12) after having removed the surnatant (plasma)**

21. With a micropipette transfer all the buffy coat (the leukocyte layer above the erythrocytes) to a new 1,5mL Eppendorf.
22. Resuspend the cell in ≈1,2mL of lysis buffer 1X (dilute lysis buffer 10X in water) and mix thoroughly (vortex).
23. Wait 7 minutes (room temperature) or until the mix looks clear (the mix becomes clear when the erythrocyte are lysed).
24. Centrifuge 5 minutes at 1500rpm.
25. Remove supernatant with a pipette without disturbing the pellet and resuspend it in ≈1,2mL of lysis buffer (wash).
26. Centrifuge again for 5 minutes at 1500rpm.
27. Remove supernatant with a pipette without disturbing the pellet and resuspend it in 500µL of FCS.
28. Count the cells in a Burker Chamber with a vital stain (Turk) and take note of the results. (approx. $15x10^6$ cells will be harvested from each 10 mL blood sample)
29. Add 500µL of Freezing mix
30. Transfer the Eppendorf in a criobox and let it freeze to -80°C overnight.

Specimen Storage Instructions

- Once frozen, maintain samples continuously at -70°C or colder.
- When outside the freezer, such as when transferring to a different freezer in another location or preparing for shipment, boxes containing tubes should be covered with dry ice.
- Freezer or dry ice specimen storage container temperature must be checked and documented at least once each workday. Document any deviation from protocol. The freezer or dry ice storage box containing the specimens should either be locked or in a secure area accessible only to authorized site staff.
- A backup storage plan should be in place in the event of freezer failure. Ship frozen samples according to Inostics' Specimen Shipment Instructions.

Supplementary Material Appendix 2.

Recruiting centers, principal investigators, and enrolled patients.

**Recruiting Center**	**Patients enrolled**
Niguarda Cancer Center, Grande Ospedale Metropolitano Niguarda, Milano, Italy - PI Dr. Andrea Sartore-Bianchi -	11
Fondazione IRCCS Istituto Nazionale dei Tumori, Milan, Italy - PI Prof. Filippo de Braud-	8
Veneto Institute of Oncology (IOV)-IRCCS Padua, Italy - PI Dr. Sara Lonardi -	7
Istituto di Candiolo, Fondazione del Piemonte per l'Oncologia, IRCCS, Candiolo, Italy - PI Prof. Massimo Aglietta -	1
POLICLINICO UNIVERSITARIO CAMPUS BIOMEDICO - PI Prof. Giuseppe Tonini -	0
HUMANITAS Research Hospital, Milano - Dr. Lorenza Rimassa -	0
**TOTAL**	**27**

Supplementary Material Appendix 3.

Inclusion and exclusion criteria Protocol version 3.0.

Inclusion Criteria

1 Histologically confirmed diagnosis of metastatic colorectal cancer;
2 Age ≥ 18 years;
3 Written informed consent;
4 Documented WT RAS exons 2, 3 and 4 (KRas and NRas) and WT BRAF V600E for anti-EGFR treatment.
5 Complete or partial response to anti EGFR antibodies in any line–either received as monotherapy or in combination with chemotherapy;
6 Imaging documented progression while on therapy with a therapeutic regimen including anti-EGFR mAb;
7 Imaging documented progression at the last treatment regimen that must be anti-EGFR free;
8 Patient must be RAS and EGFR ectodomain wild type in a liquid biopsy performed no longer that 4 weeks after progression to the last anti-EGFR free treatment

9 FFPE sample used for eligibility to anti-EGFR prescription (see criteria 4) must be available for custom gene panel profiling (as described in appendix B). Otherwise, if sample is not available, center must have already perfomed a genotyping on this tissue sample according to appendix B.

10 ECOG performance status ≤ 2;
11 At least one measurable tumor lesion as per RECIST v1.1. Lesions in previously irradiated areas or those that have received other loco-regional therapies (i.e. percutaneous ablation) should not be considered measurable unless there is clear documented evidence of progression of the lesion since therapy. Imaging must be performed maximum within 28 days prior to registration;
12 Normal organ functions.

13 Negative serum pregnancy test within 1 week prior to the first study dose in all women of childbearing potential;

14 Subjects and their partners must be willing to avoid pregnancy during the trial. Male subjects with female partners of childbearing potential and female subjects of childbearing potential must, therefore, be willing to use adequate contraception;

15 Absence of any psychological, familial, sociological or geographical condition potentially hampering compliance with the study protocol and follow-up schedule; those conditions should be discussed with the patient before registration in the trial.

Exclusion Criteria

1. History of severe infusion reactions to monoclonal antibodies cetuximab or panitumumab;
2. Symptomatic or untreated leptomeningeal disease and symptomatic brain metastasis;
3. Clinically significant cardiac disease including:

    a. congestive heart failure requiring treatment (NYHA grade ≥ 2), Left ventricular ejection fraction (LVEF) < 45% as determined by Multigated acquisition (MUGA) scan or echocardiogram;

    b. history or presence of clinically significant ventricular arrhythmias or atrial fibrillation;

    c. clinically significant resting bradycardia;

    d. unstable angina pectoris ≤ 3 months prior to starting study drug;

    e. acute myocardial infarction ≤ 3 months prior to starting study drug;

    f. QTcF > 480 msec;

4. History of thromboembolic or cerebrovascular events within the last 6 months, including transient ischemic attack, cerebrovascular accident, deep vein thrombosis, or pulmonary embolism;
5. Patients with interstitial pneumonitis or pulmonary fibrosis;
6. Abnormal organ or bone marrow functions defined as:

a. Absolute neutrophil count < 1.5 x 10/L;

b. hemoglobin < 9 g/dL;

c. alkaline phosphatase > 2.5 x upper normal limit (ULN), if liver metastases > 5 x ULN;

d. aspartate aminotransferase (AST)/ alanine aminotransferase (ALT) > 2.5 x ULN, if liver metastases > 5 x ULN;

e. bilirubin > 1.5 x ULN, if liver metastases > 2 x ULN;

f. serum creatinine > 1.5 x ULN and/or creatinine clearance ≤ 50 mL/min calculated according to Cockroft-Gault;

g. Patients with platelet count <100 x 10^9/L

7. Previous or concurrent second malignancy. Exceptions: adequately treated basal cell or squamous cell skin cancer; in situ carcinoma of the cervix, treated curatively and without evidence of recurrence for at least 3 years prior to study entry; or other solid tumor treated curatively and without evidence of recurrence for at least 3 years prior to study entry.
8. Patients with positive serology for HIV, HBV, HCV.
9. Patients with a history of severe or life-threatening hypersensitivity to the active substance or to any of the excipients.

Supplementary Material Appendix 4.

Protocol deviations.

Patient ID	Protocol deviation
CAN-002	Exclusion criteria n. 8. (HBV, no active infection but Ab positive)
IOV-002	Procedures deviation (Baseline TC > 28 d. Protocol schedule)
IOV-007	Procedures deviation (Baseline TC > 28 d. Protocol schedule)

Supplementary Material Appendix 5.

Report of the independent review of radiologic responses of CHRONOS trial.

Report of the Independent Review of radiologic responses of CHRONOS trial

Product Code:	Vectibix®(panitumumab)
Report of the peer review for study:	CHRONOS 013-IRCC-10IIS-16
EudraCT:	2016-002597-12
ClinicalTrials.gov:	NCT03227926
Date of First Subject Enrolled:	August 19, 2019
Date of Last Subject Enrolled:	November 6, 2020
Date of Data Base Lock:	July 31, 2021
Principal Investigator:	Salvatore Siena, MD<br>Niguarda Cancer Center<br>Milano - Italy
Development Phase of Study:	2

APPROVAL SIGNATURES

I have read the report and confirm that to the best of my knowledge it accurately describes the conduct and results of the study.

15/09/2021

Daniele Regge, MD — Date
Diagnostic, Radio-diagnostic Department
Fondazione del Piemonte per l'Oncologia-IRCCS
Candiolo (TO) - Italy

15/09/2021

Angelo Vanzulli, MD — Date
Director, Struttura Complessa Radiologia
Niguarda Cancer Center, Grande Ospedale Metropolitano Niguarda
Milano, Italy

TABLE OF CONTENTS

1. ABBREVIATIONS AND DEFINITION OF TERMS 2
2. OVERALL REVIEW CONDUCT 3
3. REVIEW PROCESS 3
3.1. Blind independent revision 3
3.2. Reconciliation process 3
3.3. Blind independent revision 3
4. REVIEW REULTS 4
4.1. Summary of the reconciliation results by patient 8
Patient INT-013 8
Patient INT-015 8
Patient IOV-003 8
Patient IOV-006 8
Patient IOV-007 8
Patient IOV-009 8
Patient IOV-010 8
Patient NIG-001 9
Patient NIG-002 9
Patient NIG-003 9
Patient NIG-004 9
Patient NIG-006 9
Patient NIG-015 9

1. ABBREVIATIONS AND DEFINITION OF TERMS

CR	Complete Response
CRF	Case Report Form
DOR	Duration of Response
FPI	First patient in
LD	Longest Diameter
NE	Not Evaluable
ORR	Objective Response Rate
PR	Partial Response
SD	Stable Disease
PFS	Progression free survival

2. OVERALL REVIEW CONDUCT

The review was conducted by two independent reviewers with the support of Telemis version 4.9 (Telemis SA, Louvain la Neuve, Belgium). Due to the pandemic restrictions, the final reconciliation was performed via teleconference on August, 31, 2021.

The reviewers were:

- Prof. Daniele Regge, Director of the FPO Department of Radio-diagnostics, Candiolo (TO), Italy

-Dr. Angelo Vanzulli, Head of the Struttura complessa Radiologia, Ospedale Niguarda Ca'Granda-Milan, Italy

Copies of CT-Scan of all evaluable patients were collected from each participating center and uploaded on Telemis version 4.9 (Telemis SA, Louvain la Neuve, Belgium) for evaluation.

Tumor assessments by protocol were required at baseline (within 4 weeks from study drug administration) and planned every 8 weeks thereafter until progressive disease was noted. The RECIST 1.1 criteria were used for tumor response evaluation by the local Investigators and by both Reviewers.

3. REVIEW PROCESS

3.1. Blind independent revision

All CT-scans for all patients in trial were assessed by RECIST 1.1 independently. Each reviewer, blinded to both the investigator and the other independent reviewer, recorded RECIST 1.1 assessment on an electronic form.

3.2. Reconciliation process

All the cases were uploaded on Telemis version 4.9 (Telemis SA, Louvain la Neuve, Belgium). The study Project Manager flagged all discordant in either assessment of response, and or differences in the length of time dependent variables (time to response; progression free survival; duration of response). Cases requiring reconciliation were discussed collegially via teleconference by the two reviewers.

3.3. Blind independent revision

Discrepancies and decision whether reconciliation was required are reported in Table 1 (best response) and table 2 (time dependent variables).

Five of 27 patients (INT-011, NIG-009, NIG-012, NIG-013 and NIG-014) were not included in the independent revision as the baseline examinations were not available. All the above-mentioned patients were in progression at the first re-assessment (8 weeks).

Overall, 13 (48%) patients were selected for the reconciliation process due to discrepancies in the assessment of best response (n=7; 26%), or length of time dependent variable (n=9; 33%); three patients (IOV-003, IOV-006, NIG-001) are included in both series.

4. REVIEW REULTS

The results of the reconciliation process are summarized Table 3 and 4.

Best responses assigned by the local investigators was modified by reviewers according to RECIST 1.1 in three cases: two cases were downgraded from PR to SD and one was upgraded from SD to PR.

Date of progression - leading to the shortening of the individual patients' time dependent variables - was anticipated in 7 (26%) cases for TTP and 4 (15%) cases for DOR.

Table 1: Discrepancies in the definition of best response

	Assessment			Type of discrepancy	Patient ID #	ACTION
	INV	REV A	REV B			
RESPONSE (RECIST 1.1)	PD	NE	NE	Baseline images not available for revision	INT-011, NIG-009, NIG-012, NIG-013, NIG-014	Not revised
	PD	PD	PD	NONE (full concordance)	INT-001, INT-009, IOV-004	None
	SD	SD	SD	NONE (full concordance)	CAN-002, INT-015, INT-021, INT-024, IOV-002, IOV-007, NIG-002, NIG-005	None
	PR	PR	PR	NONE (full concordance)	INT-005, INT-013, NIG-006, NIG-015	None
	SD	SD	PD	Reviewer B discordant with Investigator and A	IOV-006	To be reconciled
	SD	PR	PR	Reviewer A and B discordant with Investigator	NIG-004	To be reconciled
	PR	SD	SD	Reviewer A and B discordant with Investigator	IOV-010, NIG-003	To be reconciled
	PR	PR	SD	Reviewer B discordant with Investigator and A	IOV-003, NIG-001	To be reconciled
	PR	SD	PR	Reviewer A discordant with Investigator and B	IOV-009	To be reconciled

Table 2: Discrepancies in the evaluation of dates for time-dependent variables calculation

	INV	REV A	REV B	LEVEL OF DISCREPANCY	Patient ID #	ACTION
TIME DEPENDENT VARIABLES	Time to best response	longer	longer	Reviewer A and B discordant with Investigator	NIG-004	To be reconciled

	INV	REV A	REV B	CONCORDANCE	Patient ID #	ACTION
**TIME DEPENDENT VARIABLES**	Progression free survival	NE	NE	Baseline images not available for revision	INT-011, NIG-009, NIG-012, NIG-013, NIG-014	Not revised
		NE	NE	Clinical PD, progression images not available for revision	IOV-010	Not revised
		same	same	NONE (full concordance)	CAN-002, INT-001, INT-005, INT-009, INT-021, INT-024, IOV-002, IOV-004, IOV-009, NIG-003, NIG-004, NIG-005	None
		shorter	shorter	Reviewer A and B discordant with Investigator	INT-015, IOV-003, IOV-007, NIG-002, NIG-006, NIG-015	To be reconciled
		shorter	same	Reviewer A discordant with Investigator and B	NIG-001	To be reconciled
		same	shorter	Reviewer B discordant with investigator and A	IOV-006	To be reconciled
		same	longer	Reviewer B discordant with Investigator and A	INT-013	To be reconciled

Table 3: Reconciliation results – BEST response by RECIST

Revised Patients N 22		OBJECTIVE RESPONSE POST- REVISION			
		PD	SD	PR	CR
OBJECTIVE RESPONSE PRE- REVISION	PD	N=3			
	SD		N=9	N=1	
	PR		N=2	N=7	
	CR				N=0

Table 4: Reconciliation results – Time-dependent variables

TTP after review			
**Patients N 22**	**shorter**	**same**	**longer**
**TTP before review**	N=7	N=15	None
**DOR after review**			
**Patients N 8**	**shorter**	**same**	**longer**
**DOR before review**	N=4	N=4	None

4.1. Summary of the reconciliation results by patient

Patient INT-013

Discrepancy: Date of progression

INV: PD on 25/09/20.
REV A: PD on 25/09/20.
REV B: PR on 25/09/20.

Reconciliation: PD on 25/09/20.

Patient INT-015

Discrepancy: Date of progression

INV: PD on 16/06/20.
REV A: PD on 09/06/20.
REV B: no PD on 09/06/20.

Reconciliation: PD on 09/06/20.

Patient IOV-003

Discrepancy: Best Response and Date of progression

INV: PR on 30/05/20. PD on 23/09/2020.
REV A: PR on 30/05/20. PD on 03/08/2020.
REV B: SD on 30/05/20. PD on 03/08/2020.

Reconciliation: PR on 30/05/20. PD on 03/08/2020.

Patient IOV-006

Discrepancy: Best response and Date of progression

INV: SD on 13/08/20. PD on 09/10/2020.
REV A: SD on 13/08/20. PD on 09/10/2020.
REV B: PD on 13/08/20. PD on 13/08/20.

Reconciliation: SD on 13/08/20. PD on 09/10/2020.

Patient IOV-007

Discrepancy: Date of progression

INV: PD on 11/12/2020.
REV A: PD on 06/10/2020.
REV B: PD on 06/10/20.

Reconciliation: PD on 06/10/2020.

Patient IOV-009

Discrepancy: Best response

INV: PR on 27/10/20.
REV A: SD on 27/10/20.
REV B: PR on 27/10/20.

Reconciliation: PR on 27/10/20.

Patient IOV-010

Discrepancy: Best response

INV: PR on 23/11/20. Clinical progression on 24/12/20.

REV A: SD on 23/11/20. PD not evaluated, no images available at progression.
REV B: SD on PR on 23/11/20. PD not evaluated, no images available at progression.

Reconciliation: SD on 23/11/20. PD on 24/12/20 (clinical progression)

Patient NIG-001

Discrepancy: Best response and Date of progression.

INV: PR on 24/10/19. PD on 23/04/20
REV A: PR on 24/10/19. PD on 27/02/20
REV B: SD on 24/10/19. PD on 27/02/20

Reconciliation: PR on 24/10/19. PD on 27/02/20.

Patient NIG-002

Discrepancy: Date of progression.

INV: PD on 21/09/20.
REV A: PD on 20/05/20.
REV B: PD on 20/05/20.

Reconciliation: PD on 20/05/20.

Patient NIG-003

Discrepancy: Best response

INV: PR on 16/12/19.
REV A: SD on 16/12/19.
REV B: SD on 16/12/19.

Reconciliation: SD on 16/12/19.

Patient NIG-004

Discrepancy: Best response

INV: SD on 09/01/20.
REV A: PR on 19/02/20.
REV B: PR on 19/02/20.

Reconciliation: PR on 19/02/20.

Patient NIG-006

Discrepancy: Date of progression.

INV: PD on 12/08/20.
REV A: PD on 18/06/20.
REV B: PD on 18/06/20.

Reconciliation: PD on 18/06/20.

Patient NIG-015

Discrepancy: Date of progression.

INV: PD on 28/06/21.
REV A: PD on 28/04/21.
REV B: PD on 28/04/21.

Reconciliation: PD on 28/04/21.

CLINICAL STUDY PROTOCOL

VELO

Phase II randomized study evaluating the efficacy of panitumumab (VEctibix) and Trifluridine-Tipiracil (LOnsurf) in pretreated *RAS* wild type metastatic colorectal cancer patients: the VELO trial

Protocol code: **VELO**

Protocol version: 2.0

Protocol date: 10/Apr/2019

EudraCT number:

Sponsor: Dipartimento di Medicina di Precisione, Università degli studi della Campania Luigi Vanvitelli.

TABLE OF CONTENTS

1 SYNOPSIS 8
1.1 BACKGROUND 11
1.2 STUDY RATIONALE 12
2 AIMS OF THE STUDY 14
2.1 Study Hypothesis 14
2.2 Objectives of the study 14
2.2.1 Primary objective 14
2.2.2 Secondary objectives 14
2.2.3 Exploratory secondary objectives 14
2.3 Endpoints of the study 14
2.3.1 Primary Endpoint 14
2.3.2 Secondary Endpoints 15
2.3.3 Exploratory secondary Endpoints 15
3 SELECTION OF PATIENTS 15
3.1 Inclusion Criteria 15
3.2 Exclusion Criteria 17
4 STUDY DESIGN AND PROCEDURES 18
4.1 Sample size 19
4.2 Study duration 19
4.3 Treatment plan 19
4.3.1 Treatment dosage and schedule 20
4.3.2 Dose modification, delays and interruptions criteria 23
4.3.3 Concomitant treatments 29
4.4 Schedule of assessments and procedures 31
4.4.1 Procedures for enrolment of eligible patients 31
4.4.2 Screening and Baseline Assessments 32
4.4.3 Assessment during treatment 33
4.4.4 Assessments at End of Treatment 34
4.4.5 Follow-Up Assessments 34
4.4.6 Collection of CT scans 35
5. RESPONSE EVALUATION 39
5.1 Measurability of tumor lesions 39
5.2. Identification of "target" and "non-target" lesions 39
5.3 Evaluation of target lesion response 40
5.4. Evaluation of non-target lesion response 40
5.5. Evaluation of best overall response 40
5.6. Reporting of results 41
6. BIOMARKERS 41
6.1 Blood Sample Collection and analysis 41
6.2 Tumor Tissue Samples Collection 41
7. SAFETY INSTRUCTIONS AND GUIDANCE 42
7.1 Warning and management of specific toxicities 42
7.2 Adverse Events and Laboratory Abnormalities 44
7.2.1 Clinical Adverse Events (AEs) 44
7.2.2 Adverse Drug Reaction (ADR) 46

7.2.3 Serious Adverse Events (SAE) 46
7.2.4 Serious adverse drug reaction (SADR)………………………………………...47
7.2.5 Special situations…………………………………………………………………48
7.3 Treatment and Follow-up of AEs 49
7.4 Laboratory Test abnormalities 49
7.4.1 Follow-up of Abnormal Laboratory Test Values 50
7.5 Handling of Safety Parameters 50
7.5.1 Reporting of AEs 50
7.5.2 Reporting of SAEs (immediately reportable) 50
7.5.3 Pregnancy 51
7.5.4 Safety data exchange requirement 52
8. STATISTICAL CONSIDERATIONS 52
8.1 Sample size calculation 52
8.2 Analysis Populations 53
8.3 Statistical analysis 53
9. DATA COLLECTION AND MANAGEMENT 55
9.1 Data confidentiality 55
9.2 Site monitoring 55
9.3 Data collection 55
9.4 Database management and quality control 56
10. ETHICAL CONSIDERATION 56
10.1 Regulatory and ethical compliance 56
10.2 Responsibilities of the investigator and IEC 56
10.3 Informed consent procedures 56
10.4 Publication of study protocol and results 56
10.5 Study documentation, record keeping and retention of documents 57
10.6 Confidentiality of study documents and patient records 57
10.7 Audits and inspections 57
10.8 Financial disclosures 58
11. PROTOCOL ADHERENCE 58
11.1 Amendments to the protocol 58
12. ADMINISTRATIVE ASPECTS 58
12. 1 Investigational medical product 58
12.1.1 Formulation, Packaging and Labelling 58
12.1.2 Accountability, assessment of compliance and destruction of the drugs…………..59
13. References 60
14 APPENDICES 63
....

LIST OF ABBREVIATIONS

AE Adverse Event
ANC Absolute neutrophil count
CRF Case Report Form
CI Confidence interval
CNS Central nervous system
CRC Colorectal cancer
CTC Common toxicity criteria
cfDNA Circulating free tumor DNA
ECOG Eastern Cooperative Oncology Group
ECG Electrocardiogram
EGFR Epidermal Growth Factor Receptor
GCP Good Clinical Practice
HR Hazard ratio
ICH International Conference on Harmonization
IEC Independent Ethics Committee
IRB Institutional Review Board
ITT Intention To Treat
mCRC Metastatic colorectal cancer
MoAb Monoclonal antibody
NGS Next generation sequencing
OR Odds ratio
ORR Overall response rate
OS Overall survival
PD Disease progression
PFS Progression free survival
Pts Patients
PS Performance status
QoL Quality of life
RR Response rate
SAE Serious Adverse Event
SC Steering Committee
WBC white blood cell
WT Wild type

1 PROTOCOL SYNOPSIS

**Study Title**	Phase II randomized study evaluating the efficacy of panitumumab (VEctibix) and Trifluridine-Tipiracil (LOnsurf) in pretreated *RAS* wild type metastatic colorectal cancer patients: the VELO trial
**Study ID**	
**Study Acronyms**	VELO
**EudraCT Number**	2018-001600-12
**Trial Registration ClinicalTrials.Gov Number**	NCT XXXXX
**Sponsor**	Dipartimento di Medicina di Precisione,<br>Università degli studi della Campania "Luigi Vanvitelli"
**Setting**	Patients with *RAS* WT metastatic colorectal cancer, third line setting
**Phase**	II
**Number of patients**	112
**Background**	The Epidermal Growth Factor Receptor (EGFR) targeted therapy with the monoclonal antibodies cetuximab or panitumumab represents a major step forward in the treatment of *RAS* wild type (WT) metastatic colorectal cancer (mCRC), given the relevant efficacy in terms of progression-free survival (PFS), overall survival (OS), response rate (RR), as well as quality of life (QoL), observed in several phase III clinical trials among different lines of treatment. However, the clinical benefit observed with these agents is limited to only a subset of patients and responses are often transient due to the development of various mechanisms of resistance. Several studies have provided new insights into molecular basis of EGFR inhibitors resistance and have identified mutations in KRAS, NRAS, BRAF and EGFR extracellular domain (ECD) as well as the amplification of ERBB2 and MET, as biomarkers of both primary and/or acquired resistance to these drugs. Unraveling the biology underlying the complex mechanisms of resistance have been useful for developing rational combination therapies in order to revert or overcome resistance. Rechallenge with an alternative anti-EGFR monoclonal antibody (MoAb) after failure with an agent of the same family has been proposed as strategy to overcome drug resistance. However, panitumumab, as single agent, has demonstrated to provide minimal benefit in patients with KRAS WT mCRC who have experienced progression to cetuximab as prior therapy. The hypotheses that pre-existing sensitive subclones may emerge after treatment breaks with anti-EGFR MoAb has led the design of several clinical trials prospectively evaluating the rechallenge with anti-EGFR MoAbs in the third-line setting after a response to a first-line therapy with anti-EGFR drugs. Trifluridine-Tipiracil demonstrated to be effective in the treatment of refractory mCRC. Anti-EGFR agents in combination with Trifluridine-

	Tipiracil have shown preliminary activity in SW48 (KRAS WT) xenograft models. Panitumumab in combination with Trifluridine-Tipiracil could be a valid therapeutic option for mCRC *RAS* WT patients that achieved a major response in the first line of therapy to panitumumab or cetuximab as third line rechallenge treatment.
**Rationale**	The aim of this study is to investigate a rechallenge strategy of panitumumab in combination with Trifluridine-Tipiracil as compared to standard third line therapy (Trifluridine-Tipiracil) in patients with *RAS* WT mCRC treated in first line with chemotherapy in combination with an anti-EGFR drug that have had a clinical benefit (complete or partial response) from treatment.
**Primary Objectives**	To explore the efficacy of panitumumab in combination with Trifluridine-Tipiracil vs standard third line therapy (Trifluridine-Tipiracil) as measured by PFS
**Secondary Objectives**	To compare the activity of panitumumab in combination with Trifluridine-Tipiracil vs standard third line therapy (Trifluridine-Tipiracil) as measured by ORR<br>To compare the efficacy of panitumumab in combination with Trifluridine-Tipiracil vs standard third line therapy (Trifluridine-Tipiracil) as measured by OS<br>To explore the safety and tolerability of panitumumab in combination with Trifluridine-Tipiracil vs standard third line therapy (Trifluridine-Tipiracil)
**Study plan**	This is a randomized phase II, open label, two arm study, evaluating the efficacy of panitumumab in combination with Trifluridine-Tipiracil as third line therapy, after a first line containing an anti-EGFR agent panitumumab (at least 70% of study population) or cetuximab.<br><br>Arm A: Trifluridine-Tipiracil<br>Arm B: Panitumumab+ Trifluridine-Tipiracil
**Sample size**	The study is designed to have 80% power to detect a hazard ratio for progression of 0.56 (a 44% reduction in risk) in the Trifluridine-Tipiracil + panitumumab group as compared with the Trifluridine-Tipiracil group, with a two-sided type I error rate of 0.1. Given the treatment assignment ratio of 1:1 (Trifluridine-Tipiracil : panitumumab + Trifluridine-Tipiracil), we calculate that at least 74 events (progression) in 112 subjects (56 in the control group and 56 in the treatment group) would be required for the primary analysis.
**Inclusion criteria**	• Histologically proven diagnosis of colorectal adenocarcinoma<br>• Diagnosis of metastatic disease<br>• *RAS* (NRAS and KRAS exon 2,3 and 4) wild-type in tissue at initial diagnosis<br>• Efficacy of a first line therapy containing an anti-EGFR agent (panitumumab or cetuximab) with a major response achieved (complete or partial response)

	• Progression after a second line therapy<br>• Available and adequate baseline tumour tissue sample<br>• Measurable disease according to RECIST criteria v1.1<br>• Male or female patients > 18 years of age<br>• ECOG Performance Status 0-1<br>• Life expectancy of at least 3 months<br>• Adequate bone marrow, liver and renal function<br>• If female and of childbearing potential, have a negative result on a pregnancy test performed a maximum of 14 days before initiation of study treatment<br>• If female and of childbearing potential, or if male, agreement to use adequate contraception (eg, abstinence, intrauterine device, oral contraceptive, or double-barrier method)<br>• Signed informed consent
**Exclusion criteria**	• Any contraindication to use Trifluridine - Tipiracil or Panitumumab<br>• More than two previous lines of treatment<br>• Active uncontrolled infections<br>• Past or current history of malignancies other than colorectal carcinoma, except for curatively treated basal and squamous cell carcinoma of the skin or in situ carcinoma of the cervix<br>• Pregnancy<br>• Breastfeeding<br>• Interstitial lung disease or pulmonary fibrosis<br>• Grade III or IV heart failure (NYHA classification)
**Statistical methods**	The study is designed to have 80% power to detect a hazard ratio for progression of 0.56 (a 44% reduction in risk) in the Trifluridine-Tipiracil + panitumumab group as compared with the Trifluridine-Tipiracil group, with a two-sided type I error rate of 0.1. Given the treatment assignment ratio of 1:1 (Trifluridine - Tipiracil: Panitumumab + Trifluridine - Tipiracil), we calculate that at least 74 events (progression) in 112 subjects (56 in the control group and 56 in the treatment group) would be required for the primary analysis.<br><br>The median period of follow-up will be calculated for the entire study cohort according to the reverse Kaplan–Meier method. Distributions of time-to-event variables will be estimated with the use of the Kaplan–Meier product-limit method. The stratified log-rank test will be used as the primary analysis for comparison of treatment groups. Cox proportional-hazards modeling will be also performed as supportive analyses. Subgroup analyses of progression-free survival will be performed by means of an interaction test to determine the consistency of the treatment effect according to key baseline characteristics. Overall survival will be analyzed with the same methods used for the analysis of progression-free survival.<br>The objective response rate and the incidence of adverse events in the different groups will be compared with the use of the chi-square test for heterogeneity.<br>All statistical tests will be two-sided, and P values of 0.05 or less will be considered to indicate statistical significance. Odds ratios and 95%

	confidence intervals will be estimated with a logistic-regression model, and hazard ratios and 95% confidence intervals will be estimated with a Cox proportional hazards model.
**Duration of the study**	30 months
**Total Number of Centers**	13 Italian centers
**Data Management**	Mrs. Daniela Renato<br>Dr Vincenzo Famiglietti
**Contact for Scientific Issues**	Prof Fortunato Ciardiello<br>Dr Erika Martinelli<br>Dr Teresa Troiani<br>Dr Claudia Cardone<br>Dr Vincenzo De Falco
**Contact for Coordination and Administrative Issues**	Mrs Daniela Renato

1.1 BACKGROUND

Colorectal cancer (CRC) is the second most commonly diagnosed cancer in Europe and a leading cause of cancer death in the world, accounting for about 1.4 million new cases and almost 700.000 deaths in 2012 (Ferlay et al 2013 GLOBOCAN)

Five-year survival is highly dependent on the stage at diagnosis. Approximately one-fourth of CRC patients present metastatic disease at the time of diagnosis, and a further one-third will develop metastases during the course of the disease.

The introduction of oxaliplatin and irinotecan and the development of combination chemotherapy regimens FOLFOX (5-FU, folinic acid and oxaliplatin) (De Gramont et al 2000), FOLFIRI (5-FU, folinic acid, and irinotecan) (Douillard et al 2000) and FOLFOXIRI (5-FU, folinic acid, irinotecan and oxaliplatin) (Falcone et al 2007) has dramatically improved outcomes of patients with metastatic CRC (mCRC). Furthermore, recently, Trifluridine-Tipiracil, an oral cytotoxic agent, on the basis of RECOURSE trial, has been approved for the treatment of patients with refractory mCRC (Mayer et al 2015).

The recent advances made in the knowledge of tumor biology and molecular genetics have further influenced the treatment of mCRC patients, with the introduction of novel molecularly targeted agents: monoclonal antibodies (MoAbs) against vascular endothelial growth factor

(VEGF), bevacizumab (Hurwitz et al 2004); MoAbs against epidermal growth factor receptor (EGFR), cetuximab (Van Cutsem et al 2009) or panitumumab (Douillard et al 2010); the anti-angiogenic fusion decoy receptor blocking the activity of VEGFA, VEGFB, and placental growth factor, aflibercept (Van Cutsem et al 2012); and the multikinase receptor inhibitor regorafenib (Grothey et al 2013). Following the development of new treatment strategies and the increasing integration with surgery and local therapies, the outcome of patients with mCRC has progressively achieved a median survival of more than 30 months (Van Cutsem et al 2016). However, despite the recent developments, the efficacy of current treatment strategies, in particular for molecular targeted agents, is limited by the occurrence of drug-resistance mechanisms.

Whereas for bevacizumab, as well for other anti- angiogenic drugs, there is no predictive biomarker, it has been demonstrated that the efficacy of anti-EGFR monoclonal antibodies is limited to molecularly selected patients whose tumor is *RAS* and *BRAF* wild type. Several studies have provided new insights into molecular basis of EGFR inhibitors resistance and have identified mutations in *KRAS*, *NRAS*, *BRAF* and EGFR extracellular domain (ECD) as well as the amplification of *ERBB2* and *MET*, as biomarkers of both primary and/or acquired resistance (Sforza et al 2016, Schirripa et al 2016).

1.2 STUDY RATIONALE

Advances in the molecular understanding of the complex mechanisms of resistance to anti-EGFR MoAb, with the use of innovative technologies, such as liquid biopsies, are leading to the development of novel approach to revert or overcome resistance.

Rechallenge with an alternative anti-EGFR monoclonal antibody (MoAb) after failure with an agent of the same family has been proposed as strategy to overcome drug resistance. Recent studies have suggested a limited benefit from a retreatment strategy with anti-EGFR therapy in mCRC.

In a phase II study 39 patients with KRAS exon 2-wt mCRC who had previously progressed following an initial clinical benefit to cetuximab-based therapy, were retreated with cetuximab and irinotecan. Results demonstrated an overall response rate of 53.8 %, stable disease rate was 35.9 %, and the median progression-free survival was 6.6 months (Santini et al 2012). In PANERB trial 32 patients with KRAS wild-type mCRC were prospectively treated with cetuximab and irinotecan followed by panitumumab monotherapy after progression. In 11 patients who had previously responded to cetuximab and irinotecan, an objective response rate of 22 % to panitumumab, including a disease control rate (objective response plus stable

disease) of 73 % was observed (Wadlow *et al* 2012). In heavily pre-treated patients without acquired resistance to prior cetuximab-based regimens, panitumumab obtained 67 % disease control rate and 30 % objective response rate, with median PFS of 4.2 and median OS of 9.6 months (Pietrantonio et al 2013).

Trifluridine-Tipiracil, is an oral agent that combines trifluridine (TFD) and tipiracil hydrochloride (TP), that have provided benefit of 2 months in overall survival and have recently been approved for the treatment of refractory mCRC (Mayer et al 2015). Trifluridine-Tipiracil antitumor efficacy is mainly explained by the inhibition of thymidylate synthase (Santi et al 1971; Temmink et al., 2004), and the incorporation of FTD-TP into DNA with subsequent DNA damage (Tanaka et al., 2014; Suzuki et al, 2011; Matsuoka et al, 2015). Importantly, Trifluridine-Tipiracil exhibits antitumor activity against FU-resistant cell lines in preclinical xenograft models (Emura et al., 2004; Emura et al., 2004; van der Velden et al., 2016).

Panitumumab in combination with Trifluridine-Tipiracil have shown preliminary activity in KRAS WT tumor xenografts model (Tsukihara et al. 2015). Moreover, Baba et al have suggested that panitumumab interacts with TFD by targeting EGFR-mediated adaptive responses, enhancing anti-tumour effects when used in combination with Trifluridine-Tipiracil (Baba et al 2017).

The Phase I-II study (APOLLON) has recently evaluated panitumumab in combination with Trifluridine-Tipiracil in patients with *RAS* WT mCRC, refractory to standard chemotherapies (NCT02613221). The recommended phase II dose of this combination was reported as standard dose (panitumumab: 6 mg/kg on days 1 and 15, every 4 weeks; Trifluridine-Tipiracil: 35 mg/m2 BID on days 1-5 and 8-12, every 4 weeks) (Kato et al 2017).

Preliminary data have shown favorable antitumor activity with an acceptable safety profile: median PFS, RR and DCR were 5.8 months (95% CI: 4.5–6.5), 37.0% and 81.4%, respectively (n = 54); The most common grade (G) ≥3 treatment-emergent adverse events (n = 55) were neutropenia (G3: 30.9%, G4: 16.4%), febrile neutropenia (G3: 10.9%), stomatitis (G3: 9.1%), dermatitis acneiform (G3: 9.1%), fatigue (G3: 3.6%) and hypomagnesemia (G3: 3.6%), no treatment-related deaths or unexpected safety signals was reported.

2. AIMS OF THE STUDY

2.1 Study Hypothesis

The aim of this study is to investigate a rechallenge strategy of panitumumab in combination with Trifluridine-Tipiracil as compared to standard third line therapy (Trifluridine-Tipiracil) in

patients with *RAS* WT mCRC, treated in first line with chemotherapy in combination with an anti-EGFR drug and have obtained a clinical benefit (complete or partial response) from treatment.

2.2 Objectives of the study

2.2.1 Primary Objective

- To explore the efficacy of panitumumab in combination with Trifluridine-Tipiracil vs standard third line therapy (Trifluridine-Tipiracil) as measured by PFS

2.2.2Secondary Objectives

- Compare the activity of panitumumab in combination with Trifluridine-Tipiracil vs standard third line therapy (Trifluridine-Tipiracil) as measured by ORR
- Compare the efficacy of panitumumab in combination with Trifluridine-Tipiracil vs standard third line therapy (Trifluridine-Tipiracil) as measured by OS
- To explorethe safety and tolerability of panitumumab in combination with Trifluridine-Tipiracil vs standard third line therapy (Trifluridine-Tipiracil)

2.2.3 Exploratory secondary objectives

- To explore the mutational profile and evaluate acquired mutations in plasma circulating cell-free DNA (cfDNA) and to explore the relationship with panitumumab in combination with Trifluridine-Tipiracil activity

2.3 Endpoints of the study

2.3.1 Primary Endpoint

- PFS: defined as the time from randomization to the earliest documented disease progression or death due to any cause of panitumumab in combination with Trifluridine-Tipiracil vs Trifluridine-Tipiracil

2.3.2 Secondary Endpoints

- ORR: per the Response Evaluation Criteria in Solid Tumors (RECIST), version 1.1 (v1.1), defined as the number of patients achieving an overall best response of complete or partial response divided by the total number of patients
- OS: defined as the time from randomization to death due to any cause of panitumumab in combination with Trifluridine-Tipiracil vs Trifluridine-Tipiracil
- Safety analysis: defined as the evaluation of incidence and severity of Adverse Events (AEs), graded according to National Cancer Institute (NCI) Common Terminology Criteria for Adverse Events (CTCAE), version 5.0 (v. 5.0) of panitumumab in combination with Trifluridine-Tipiracil vs standard third line therapy (Trifluridine-Tipiracil)

2.3.3 Exploratory secondary endpoints

- Genomic analysis of blood samples collected baseline, during treatment period and at end of treatment

3. SELECTION OF PATIENTS

3.1 Inclusion Criteria

Patients must meet the following criteria for study entry:

- Signed informed consent to any study-specific procedure.
- Age ≥18 years
- Eastern Cooperative Oncology Group (ECOG) performance status of 0 or 1 (see Appendix 1)
- Histologically confirmed adenocarcinoma of the colon or rectum
- Stage IV American Joint Committee on Cancer [AJCC] 8th edition
- Measurable tumour lesions according to RECIST v1.1 (with at least one measurable lesion)
- Life expectancy of at least 3 months
- *RAS* (NRAS and KRAS exon 2,3 and 4) wild-type in tissue at initial diagnosis
- Experienced disease progression on two prior systemic chemotherapy regimens for mCRC, as follows:

1. Received a first line therapy containing an anti-EGFR agent (panitumumab or cetuximab) and obtained an objective response (complete or partial response) from treatment;

2. Progressed to a second line regimen of standard chemotherapy (including fluoropyrimidines, irinotecan, oxaliplatin, bevacizumab or aflibercept)

Note:

- Patient who is refractory to chemotherapy is defined as a patient who didn't respond (obtaining a PR or CR) from the beginning of the treatment (primary refractory) or, after initial response, experiences progression while on treatment (secondary refractory);
- Patient who is intolerant to chemotherapy is defined as a patient who has been withdrawn from standard treatment due to unacceptable toxicity warranting discontinuation of treatment and precluding retreatment with the same agent prior to progression of disease. This patient will also be eligible to enter the study;

- Able to take medications orally (*i.e*., no feeding tube)
- Available and adequate baseline tumour tissue sample
- Adequate organ function as defined by the following laboratory values:

- Hemoglobin value of $\geq$9.0 g/dL.
- Absolute neutrophil count of $\geq 1.5\times 10^9$/L by International Units (IU).
- Platelet count $\geq 100 \times 10^9$/L
- Total serum bilirubin of $\leq$1.5 mg/dL (except for Grade 1 hyperbilirubinemia due solely to a medical diagnosis of Gilbert's syndrome)
- Aspartate aminotransferase (AST) and alanine aminotransferase (ALT) $\leq 3.0\times$upper limit of normal (ULN); if liver function abnormalities are due to underlying liver metastasis, AST and ALT $\leq 5 \times$ ULN
- Creatinine clearance $\geq$ 60 mL/min (assessed with Cockcroft-Gault formula)

- Women of childbearing potential must have been tested negative in a serum pregnancy test within 14 days prior to first day of test drug administration

  Note:

- Female participants of childbearing potential and male participants with partners of childbearing potential must agree to use a highly effective method of birth control (*i.e*., pregnancy rate of less than 1% per year) during the study and for 6 months after the discontinuation of study medication

- A women is not considered to be of childbearing potential if she has become amenorrheic for > 12 months and has follicle-stimulating hormone level ≥40 IU/L

Contraceptive methods that result in a low failure rate when used consistently and correctly include methods such as combined hormonal contraception associated with inhibition of ovulation (oral, intravaginal, transdermal), progestogen-only hormonal contraception associated with inhibition of ovulation (oral, injectable, implantable), some intrauterine devices (IUDs), intrauterine hormone-releasing system (IUS), true sexual abstinence, bilateral tubal occlusion, or a female partner who is not of childbearing potential or a male partner who has had a vasectomy. Women and female partners using hormonal contraceptive must also use a barrier method *i.e.* condom or occlusive cap (diaphragm or cervical/vault caps).

3.2 Exclusion Criteria

A subject is excluded from the study if any of the following criteria is met:

- Unlikely to cooperate in the study
- Pregnancy, breastfeeding or possibility of becoming pregnant during the study
- Hypersensitivity to the active substances or to any of the excipients of Trifluridine-Tipiracil or panitumumab
- More than two previous lines of treatment
- Previously received Trifluridine-Tipiracil
- Other concurrently active malignancies, excluding skin basal cell and cervix in situ cancers cured by adequate treatment
- Active or untreated Central Nervous System (CNS) metastases are excluded. Patients with treated and asymptomatic CNS metastases are eligible if they meet all of the following:
- Evaluable disease outside the CNS
- No evidence of intracranial haemorrhage or spinal cord haemorrhage
- Corticosteroids ant anti-convulsivant at a stable dose
- Myocardial infarction within the last 12 months, severe/unstable angina, symptomatic congestive heart failure New York Heart Association (NYHA) class III or IV

- Uncontrolled tumor-related pain. Patients requiring pain medications must be on a stable regimen at study entry
- Symptomatic lesions (e.g. bone metastases) amenable to palliative radiotherapy should be treated prior to start of study treatment
- Active infection (e.g., body temperature ≥38°C due to infection)
- Pulmonary fibrosis or cerebrovascular disorder at the time of screening
- Uncontrolled diabetes
- Active gastrointestinal haemorrhage or intestinal obstruction at the time of screening
- In the investigator's opinion, significant malabsorption syndrome, significant chronic digestive or gastrointestinal inflammatory syndrome
- Known human immunodeficiency virus (HIV) or acquired immunodeficiency syndrome (AIDS)-related illness, or active hepatitis B or C.
- Autoimmune disorders or history of organ transplantation who require immunosuppressive therapy
- Psychiatric disease that may increase the risk associated with participation or drug administration, or may interfere with the interpretation of results
- Rare hereditary problems of galactose intolerance, the Lapp lactase deficiency, or glucose-galactose malabsorption
- Major surgery within 4 weeks prior to first day of study drug administration
  Note: indwelling catheter and Portacath® implantation are allowed
- Any anticancer therapy within 3 weeks prior to first day of study drug administration
- Radiation therapy within 3 weeks prior to first day of study drug administration
- Unresolved toxicity of ≥Grade 2 CTCAE attributed to any prior therapy (excluding anaemia, alopecia, skin pigmentation, and platinum-induced neurotoxicity)

4. STUDY DESIGN AND PROCEDURES

This is a randomized phase II, open label, two arm study, evaluating the efficacy of panitumumab in combination with Trifluridine-Tipiracilas third line therapy, after a first line containing an anti-EGFR agent panitumumab(at least 70% of study population) or cetuximab. The primary objective is to evaluate the efficacy of panitumumab in combination with Trifluridine-Tipiracil (as measured by PFS) in pretreated mCRC patients. The secondary objective is to evaluate the ORR, OS and safety. Patients will be randomized in a 1:1 ratio to receive:

Arm A: Trifluridine-Tipiracil

Arm B: Panitumumab + Trifluridine-Tipiracil

4.1 Sample size

A total of 112 patients, 56 for arm, will be enrolled.

4.2 Study Duration

It is planned that patient's enrolment will be completed in 18 months with an expected total study duration of approximately 30months considering an additional 12 months of follow up from last patient enrolled.

4.3 Treatment plan

Eligible Patients (see inclusion and exclusion criteria see sections 3.1 and 3.2) will be randomized to receive a third line treatment with panitumumab in combination with Trifluridine-Tipiracil or Trifluridine-Tipiracil as monotherapy (see Study Design, Figure 1).

Randomization will be stratified by baseline Eastern Cooperative Oncology Group (ECOG PS: 0 vs 1), prior anti-EGFR agent administered (panitumumab at least 70% of study population vs cetuximab), and tumor sidedness (right vs left colon). In particular, the tumors located from the caecum to a point approximately half the two-thirds of the way along the transverse colon will be considered as "right colon", whilst the tumors located from the distal third of the transverse colon to the rectum will be considered "left colon".

Treatment will be administered in 28-days cycles until disease progression, unacceptable toxicity, withdrawal of consent or death due to any cause.

All patients will be closely monitored for safety and tolerability during all cycles of therapy, at the treatment discontinuation visit, and during the follow-up period. The NCI v.5.0 will be used to characterize the toxicity profile of the study treatments on all patients.

Patients who discontinue treatment for reasons other than disease progression (e.g., toxicity) will continue scheduled tumor assessments until disease progression, withdrawal of consent,

study termination by Sponsor, or death, whichever occurs first. In the absence of disease progression, tumor assessments should continue regardless of whether patients start a new anti-cancer therapy, unless consent is withdrawn. All patients will be followed for survival unless consent is withdrawn (see section 4.4.5).

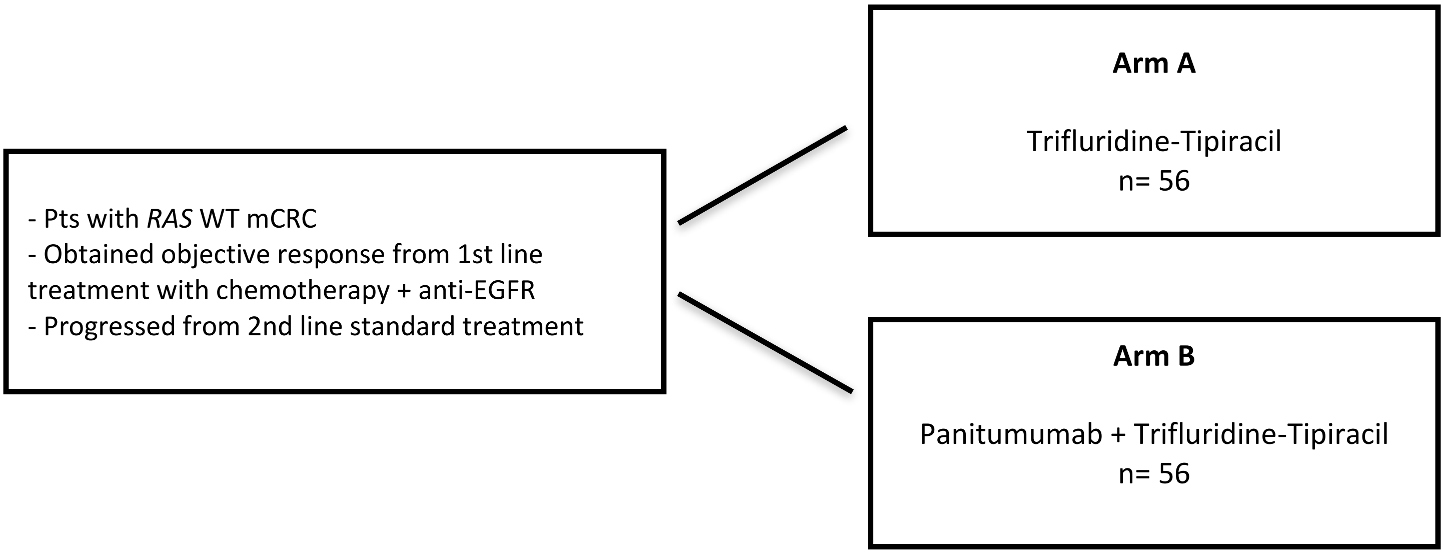

Figure 1. Study Design

4.3.1 Treatment dosage and schedule

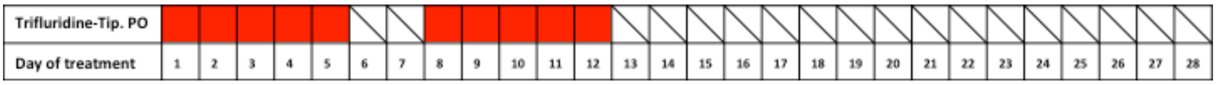

Figure 2.1. Treatment Schedule: Arm A (Trifluridine-Tipiracil)

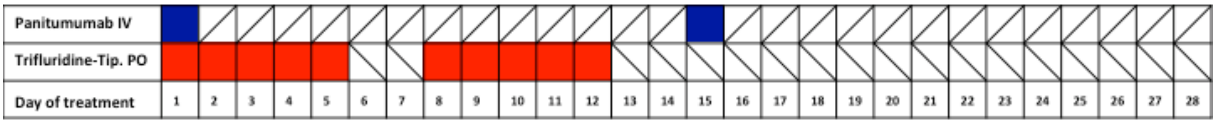

Figure 2.2. Treatment Schedule: Arm B (Panitumumab + Trifluridine-Tipiracil)

Panitumumab administration

Panitumumab will be administered as a 6 mg/kg intravenous infusion over 60 minutes every 2 weeks (q2w) of a 28-day cycle (Day 1 and Day 15) (see Figure 2.2)

Note: If the subject's weight changes by ≥ 10% during the course of the study, the dose of panitumumab will be recalculated. No ideal body weight should be used for the calculation of BSA.

Panitumumab must be diluted in pyrogen-free 0.9% sodium chloride for injection using aseptic technique to a recommended total volume of 100 mL. Doses higher than 1000 mg should be diluted to 150 mL. The maximum concentration of the final solution to be infused should not exceed 10 mg/mL.

The diluted products should be used within 6 hours of preparation if stored at room temperature, or within 24 hours of dilution if stored refrigerated at 2ºC and 8ºC. Recommended safety measures for preparation and handling of drugs include laboratory coats and gloves.

For details on prepared drug storage and use time of drugs under room temperature and refrigeration, please refer to pharmacy reference sheets.

Pre-emptive skin treatment regimen is mandatory, if there are not contraindications and it must be administered one day before the administration of the first Panitumumab dose and continued through weeks 1 to 6 (Please refer to paragraph 4.3.3 "Concomitant medication" and Permissible concomitant medications and treatments" for more details).

Trifluridine-Tipiracil administration

Trifluridine-Tipiracil will be administered at 35 mg per square meter orally twice daily, with a glass of water within 1 hour after completion of morning and evening meals, 5 days a week, with 2 days of rest, for 2 weeks, followed by a 14-day rest period (see Figure 2.1 and 2.2). One treatment cycle with Trifluridine-Tipiracil consists of the following:

Days 1-5: Trifluridine / Tipiracil (35 mg/m^2/dose) orally twice daily

Days 6-7: Rest

Days 8-12: Trifluridine / Tipiracil(35mg/m^2/dose) orally twice daily

Days 13-28: Rest

Note:

– If at the beginning of the next treatment cycle, a patient's body weight decreases by ≥10% from baseline, the body surface area (BSA) and the dosage of Trifluridine-Tipiracil has to be adjusted (See Table 1 and Table 8).
– No increase in Trifluridine-Tipiracil dose due to increase in BSA is permitted.
– If doses are missed or held, the patient should not make up for missed doses.
– Any missed doses reported by the patient should be recorded in the patient's diary.
– Extension of study treatment into Days 6 to 7 or into the rest period (Days 13 through 28) is not permitted.

For information on the formulation, packaging and handling of Trifluridine-Tipiracil, see Trifluridine-Tipiracil, Lonsurf ® prescribing information.

**Trifluridine-Tipiracil dose (2 x daily)**	**BSA (m^2)**	**Dosage in mg (2x daily)**	**Total daily dose (mg)**	**Tablets per dose**	
				**15 mg**	**20 mg**
**35 mg/m^2**	<1.07	35	70	1	1
	1.07 – 1.22	40	80	0	2
	1.23 – 1.37	45	90	3	0
	1.38 – 1.52	50	100	2	1
	1.53 – 1.68	55	110	1	2
	1.69 – 1.83	60	120	0	3
	1.84 – 1.98	65	130	3	1
	1.99 – 2.14	70	140	2	2
	2.15 – 2.29	75	150	1	3
	≥ 2.30	80	160	0	4

Table 1. Number of tablets of Trifluridine- Tipiracil per dose

4.3.2 Dose Modifications, delays ad interruptions criteria

<u>**General Recommendation**</u>

Subjects should be assessed for toxicity before each treatment cycle. Toxicity will be graded according to the NCI CTCAE Version 5.0. Reasons for dose modifications, delays or interruptions, the supportive measures taken, and the outcome, will be documented in the patient's chart and recorded in the eCRF.

<u>Guidelines to be followed in the case of AEare :</u>

- For toxicities considered by the investigator unlikely to develop into serious or life-threatening events (e.g. alopecia, altered taste etc.), treatment will be continued at the same dose without reduction or interruption.
- Where several toxicities with different grades or severity occur at the same time, the dose modifications applied should be the greatest reduction applicable.
- Once a dose has been reduced it should not be increased at a later time.
- If toxicity requires a dosing delay of all study drugs for more than four weeks, the patient will be withdrawn from the study for toxicity reasons.

Note:

For patients randomized to Arm B (Panitumumab+ Trifluridine-Tipiracil) (Figure 2.2):

– If, in the opinion of the Investigator, a toxicity is considered to be due solely to one drug, the dose of the other drug does not require modification.

– If a delay related to a treatment alone is required, the other study drug should be delayed as well.

Grade of Event	Management/Dose regulation for study drug
**Grade 1**	no change in dose
**Grade 2**	Tolerable: no change in dose<br>Non tolerable: hold until ≤ grade 1 – resume at same dose
**Grade 3**	Hold* until ≤ grade 2 – resume at 1 dose level lower if indicated
**Grade 4**	Hold* until ≤ grade 2 – resume at 1 dose level lower
* Patients requiring a delay of > 4 weeks should discontinue protocol therapy	

Table 2. Study drug dose modifications according to toxicity grade

Dose delay and modification criteria for panitumumab-related adverse events

In the event of panitumumab-related adverse events patients should follow the dose interruption, resumption and reduction criteria stated in Table 2.

Note: dose modifications levels for panitumumab are stated in Table 3.

**Starting dose**	100% of original dose
**Level 1 Dose reduction**	80% of original dose
**Level 2 Dose reduction**	60% of original dose

Table 3. Panitumumab dose modification levels

It is allowed, according to the Investigator's opinion, with holding panitumumab administration, up to a maximum of four weeks, for the following reasons:

- Symptomatic skin- or nail-related toxicity requiring narcotics, systemic steroids, or felt to be intolerable by the subject (see Table 4)
- Skin or nail infection requiring IV antibiotic or IV antifungal treatment
- Any skin- or nail-related serious adverse event
- Symptomatic hypomagnesaemia and/or hypocalcaemia that persists despite magnesium and/or calcium replacement (see Table 5)

Note: for detailed toxicities grading refer to NCI CTCAE v. 5.0

Occurrence of skin toxicity(s):≥ grade 3	Administration of Panitumumab	Outcome	Dose regulation
Initial occurrence	Withhold 1 or 2 doses	Improved (< grade 3)	Continuing infusion at 100% of original dose
		Not recovered	Discontinue
At the second occurrence	Withhold 1 or 2 doses	Improved (< grade 3)	Continuing infusion at 80% of original dose
		Not recovered	Discontinue
At the third occurrence	Withhold 1 or 2 doses	Improved (< grade 3)	Continuing infusion at 60% of original dose
		Not recovered	Discontinue
At the fourth occurrence	Discontinue	-	-

Table 4: Panitumumab dose modifications and delay according to skin toxicities

Event	Grade	Adjustment
Symptomatic hypomagnesemia *First occurrence*	any	Hold Panitumumab until resolution and restart at 100% dose level with Mg++ supplementation
Symptomatic hymomagnesemia in patients treated at 100% or 80% dose level *Recurring*	any	Restart Panitumumab at 80% dose level or 60% dose level respectively

Diarrhea<br>*First Occurrence*	3 or 4	Hold Panitumumab until resolution and restart at 100% dose level
Diarrhea in patients treated at 100% or 80% dose level<br>*Recurring*	3 or 4	Restart Panitumumab at 80% dose level or 60% dose level respectively
Any haematologic or non-haematologic toxicity	4	Hold Panitumumab until resolution

Table 5: Panitumumab dose modifications and delay according to other toxicities

Dose delay and modification criteria for Trifluridine-Tipiracil related adverse events

In the event of haematological and/or non-haematological toxicities patients should follow the dose interruption, resumption and reduction criteria stated in Table 6, Table 7, Table 8.

- If the toxicities that require trifluridine / tipiracil dose reductions recur after dose reduction to 20 mg/m², trifluridine / tipiracil should be discontinued.
- A maximum of three dose reduction(s) is permitted to a minimum dose of 20 mg/m² (total 40 mg/m²/day) in 5 mg/m² steps. Dose re-escalation is not permitted at any time.

Withhold Trifluridine-Tipiracil if any of these AE occurs:	Resumption criteria[a]
Neutrophils<0.5 x 10^9/L	Neutrophils≥1.5 x 10^9/L;
Febrile Neutropenia	Febrile neutropenia resolved
Platelets<50 x 10^9/L	Platelets≥75 x 10^9/L
Grade 3 or 4 non hematologicAEs	Grade ≤1 non hematologicAEs

[a]Resumption criteria applied to the start of the next cycle for all patients regardless of whether or not the interruption criteria were met

Table 6 - Dose interruption and resumption criteria for Trifluridine-Tipiracil related AEs

Adverse events that require dose modifications:	Recommended dose modifications:
· Febrile neutropenia<br>· Grade 4 neutropenia (<0.5 x 10^9/L) or thrombocytopenia (<25 x 10^9/L) that results in more than 1 week delay in start of next cycle<br>· Non-hematologic Grade 3 or Grade 4 adverse reaction (except for Grade 3 nausea and/or vomiting controlled by antiemetic therapy or diarrhea responsive to antidiarrheal medicinal products)	· Interrupt dosing until toxicity resolves Grade ≤1 .<br>· When resuming dosing, decrease the dose level by 5 mg/m²/dose from the previous dose level (Table 8: dose reduction).<br>· Dose reductions are permitted to aminimum dose of 20 mg/m²/dose twice daily.<br>· Do not increase dose after it has been reduced.

Table 7– Recommended dose modifications for Trifluridine/Tipiracil in case of hematological and non-hematological adverse reactions

**Trifluridine / Tipiracil dose (2x daily)**	**BSA (m^2)**	**Dosage in mg (2x daily)**	**Total daily dose (mg)**	**Tablets per dose**	
				**15 mg**	**20 mg**
**Level 1 Dose reduction: From 35 mg/m² to 30 mg/m²**					
**30 mg/m²**	**<1.09**	**30**	**60**	**2**	**0**
	**1.09 – 1.24**	**35**	**70**	**1**	**1**
	**1.25 – 1.39**	**40**	**80**	**0**	**2**
	**1.40 – 1.54**	**45**	**90**	**3**	**0**
	**1.55 – 1.69**	**50**	**100**	**2**	**1**
	**1.70 – 1.94**	**55**	**110**	**1**	**2**
	**1.95 – 2.09**	**60**	**120**	**0**	**3**
	**2.10 – 2.28**	**65**	**130**	**3**	**1**
	**≥ 2.29**	**70**	**140**	**2**	**2**
**Level 2 Dose reduction: From 30mg/m² to 25mg/m²**					
**25 mg/m²**	**<1.10**	**25**	**50**	**2 (PM)[a]**	**1 (AM)[a]**
	**1.10 – 1.29**	**30**	**60**	**2**	**0**
	**1.30 – 1.49**	**35**	**70**	**1**	**1**
	**1.50 – 1.69**	**40**	**80**	**0**	**2**
	**1.70 – 1.89**	**45**	**90**	**3**	**0**
	**1.90 – 2.09**	**50**	**100**	**2**	**1**
	**2.10 – 2.29**	**55**	**110**	**1**	**2**
	**≥2.30**	**60**	**120**	**0**	**3**
**Level 3 Dose reduction: From 25 mg/m² to 20 mg/m²**					
**20 mg/m²**	**<1.14**	**20**	**40**	**0**	**1**
	**1.14 – 1.34**	**25[a]**	**50[a]**	**2(PM)[a]**	**1(AM)[a]**
	**1.35 – 1.59**	**30**	**60**	**2**	**0**
	**1.60 – 1.94**	**35**	**70**	**1**	**1**
	**1.95 – 2.09**	**40**	**80**	**0**	**2**
	**2.10 – 2.34**	**45**	**90**	**3**	**0**
	**≥2.35**	**50**	**100**	**2**	**1**

[a]At a total daily dose of 50 mg, patient should take 1 x 20mg tablet in the morning and 2 x 15mg tablets in the evening.

Table 8 - Trifluridine/Tipiracil dose reduction levels and number of tablets per dose

Criteria to resume treatment

Subjects may resume treatment with study drug when the drug-related AE(s) resolve to Grade ≤ 2 or baseline value, as per Table 2, with the exception of Trifluridine-Tipiracil toxicities, as per Table 6, Table 7, Table8.

If the criteria to resume treatment are met, the subject should restart treatment at the next scheduled time-point per protocol.

If treatment is delayed >4 weeks, the subject must be permanently discontinued from study. However, the patient can continue any treatment as per local standard practice and must be followed up with the study procedures.

Discontinuation criteria

Treatment with panitumumab should be permanently discontinued for the following reasons:

- Progressive disease during treatment
- Patient's refusal
- Interstitial lung disease (ILD)
- Grade 3 or 4 allergic/hypersensitivity reactions
- Grade 3 or 4 diarrhea not recovered withholding treatment for 1 or 2 doses
- Unacceptable AEs, or change in underlying condition such that the patient can no longer tolerate therapy
- Study drug dose reduction by more than two dose levels
- Investigator's decision
- If female, a pregnancy test result consistent with pregnancy
- Development of any intercurrent illness or situation that may, in the judgment of the investigator affect assessments of clinical status and study endpoints to a relevant degree.

Treatment with Trifluridine-Tipiracil should be permanently discontinued for the following reasons:

- Progressive disease during treatment
- Patient's refusal
- Unacceptable AEs, or change in underlying condition such that the patient can no longer tolerate therapy.
- Study drug dose reduction until < 20 mg/m²

- Investigator's decision
- If female, a pregnancy test result consistent with pregnancy
- Development of any intercurrent illness or situation that may, in the judgment of the investigator affect assessments of clinical status and study endpoints to a relevant degree.

Patients have the right to withdraw from the treatment or from the study at any time and irrespective of the reason. When applicable, patients should be informed of circumstances under which their participation may be terminated by the Investigator without their consent. The Investigator may withdraw patients from the study in the event of intercurrent illness, AEs, treatment failure after a prescribed procedure, lack of compliance with the study and/or study procedures (e.g., dosing instructions, study visits), or any reason where it is felt by the Investigator that it is in the best interest of the patient to be terminated from the *study*. Any administrative or other reasons for withdrawal must be documented and explained to the patient.

Any subject who definitively interrupts study treatment will be evaluated at the End-of-treatment Visit conducted 21 days (±7 days) after permanently stopping study treatment (see section 4.4.4).

In all cases, the reason for definitive interruption of study treatment mustbe entered in the electronic Case Report Form (eCRF) and in the subject's medical records. Patients who withdraw from the study will not be replaced. Only patients included in the study that have not started treatment with the first dose of drugs will be replaced.

4.3.3Concomitant Treatments

The patient must notify the investigational site about any new medications he/she takes after the start of the study drug. All medications (other than study drug) and significant non-drug therapies (including physical therapy, herbal/natural medications and blood transfusions) administered during the study must be listed on the Concomitant Medications or the Surgical and Medical Procedures eCRF.

Patients taking concomitant medications chronically should maintain the same dose and dose schedule throughout the study if medically feasible. However, if a concomitant medication is used intermittently during the study, this medication should be avoided on these days, if medically feasible.

Permissible concomitant medications and treatments

- Standard therapies for concurrent medical conditions. Prophylactic anti-emetics may be administered according to standard practice
- G-CSF and other haematopoietic growth factors may be used during the study for the management of acute toxicity such as febrile neutropenia when clinically indicated or at the discretion of the investigator; however, they may not be substituted for a required dose reduction. The use of granulocyte colony stimulating factors (G-CSF) as secondary prophylaxis is allowed, as per standard treatment (J. Klastersky et al 2016, ESMO guidelines). Long-term administration of erythropoietin is permitted
- Bisphosphonates and denosumab
- Subjects taking narrow therapeutic index medications, such as warfarin, quinidine, cyclosporine, and digoxin, should be monitored proactively. A subject treated with warfarin or heparin will be allowed to participate provided that the anticoagulant dose and PT-INR and a-PTT values are stable. Close monitoring (evaluation - once weekly) is recommended. If either value is above the therapeutic range, the anti-coagulant dose should be modified and the assessments should be repeated once weekly until the value is stable.
- Palliative radiotherapy may be given for control of pain or for other reasons without curative intent.
- Pre-emptive skin treatment regimen is mandatory, if there are not contraindications and it must be administered one day before the administration of the first panitumumab dose and continued through weeks 1 to 6. It consists of:

  -skin moisturizer applied to face, hands, feet, neck, back, and chest daily in the morning on rising;

  - sunscreen (PABA free, SPF ≥ 15, UVA and UVB protection) applied to exposed skin areas before going outdoors;

  - topical steroid (1% hydrocortisone cream) applied to face, hands, feet, neck, back, and chest at bedtime

  - doxycycline 100 mg twice daily. During the assumption of doxycycline, the patient must be advised to drink plenty of liquids to reduce the risk of esophageal irritation and ulceration; he/she must take doxycycline in upright position and must not lie down for an hour after taking the drug. The patient should not take iron supplements, multivitamins, calcium supplements, antacids, or laxatives within 2 hours before or after taking

doxycycline. Please remember that the absorption of tetracyclines is reduced when taken with foods, especially those containing calcium.

Prohibited concomitant medications and treatments

- No other systemic anti-cancer therapy
- Caution is required when using medicinal products that are human thymidine kinase substrates, e.g., zidovudine. Such medicinal products, if used concomitantly with Trifluridine-Tipiracil, may compete with the effector, Trifluridine, for activation via thymidine kinases. Therefore, when using antiviral medicinal products that are human thymidine kinase substrates, monitor for possible decreased efficacy of the antiviral medicinal product, and consider switching to an alternative antiviral medicinal product that is not a human thymidine kinase substrate, such as lamivudine, zalcitabine, didanosine and abacavir.

4.4 Schedule of Assessments and Procedures

All screening/baseline assessments must be performed as outlined in schedule assessment flow chart (see Tables 9)
Eligibility for the study will be determined by the Investigator, from the mandatory screening/baseline assessments, performed during screening and according to the study inclusion/exclusion criteria.
First dosing of study drugs will be determined by the patient's eligibility and the laboratory assessments done prior to dosing.
Patients will be assessed for AEs at each clinical visit according to NCI CTC-AE v. 5.0 criteria and as necessary throughout the study.
Patients who discontinue study drug for any reason (e.g., AEs, etc.) other than disease progression will continue to be followed.
Progression of disease will be documented, as well as the initiation of another cancer therapy

4.4.1 Procedures for Enrolment of Eligible Patients

A patient who has fulfilled the entry criteria will be randomized and given an identifying number. Each identifying number will be unique to the patient for whom it is issued. A patient number will not be re-used if the patient leaves the study. Under no circumstances will patients

who enroll in this study and have completed treatment as specified be permitted to re-enroll in the study. A Patient Enrolment and Identification Code List must be maintained by the Investigator.

4.4.2 Screening and Baseline Assessments

All screening/baseline evaluations must be performed within 28 days prior to randomization. Patients who fulfill all the inclusion and none of the exclusion criteria will be accepted into the study. The following clinical assessments and procedures must be completed for all patients enrolled in this study at screening/baseline and during study visits. Screening procedures could be completed during multiple visits but must be completed within the specified timeframes.

Screening procedures to be completed or that must be available within 28 days prior to randomization

- Informed Consent Form. No screening procedures may be performed until written informed consent has been obtained. Informed Consent Forms for enrolled patients and for patients who are not subsequently enrolled will be maintained at the study site
- Histologically confirmed diagnosis of colorectal adenocarcinoma, *RAS* status.
- Medical history (including allergies, relevant medical history, previous and current diseases, all ongoing medications) and Demographic data (age, gender, race);
- Tumor assessment: CT or MRI eligibility scan (chest, abdomen, pelvis, and other suspected sites as applicable) meeting the standard of care for the imaging of the respective organ system(s). All measurable disease must be documented.
  Note: Tumor assessment performed prior to patient ICF signature may be used if the date of evaluation is within 28 days prior to randomization.
- 12-lead ECG
- ECOG Performance status
- Physical exam including height (screening only) and weight and skin examination. Changes from baseline abnormalities should be recorded at each subsequent physical examination. New or worsened abnormalities should be recorded as AEs if appropriate
- Vital signs (respiratory rate, pulse, blood pressure and temperature) will be obtained in the same position
- Haematology (including: haemoglobin, haematocrit, Platelet Count, white blood cell [WBC] count, absolute neutrophil count [ANC]); All laboratory tests performed during

patient's visit and inter visits laboratory tests (unscheduled tests) could be assayed in any certified laboratory (upon communication to the Sponsor of laboratory normal ranges).

- All samplings for biochemistry should be taken in fasting conditions.
- Biochemistry (including: glucose, blood urea nitrogen (BUN), creatinine or creatinine clearance, sodium, potassium, calcium, magnesium, chloride, total bilirubin with fractionation into direct and indirect (if total bilirubin elevated), alkaline phosphatase, aspartate aminotransferase [AST, or serum glutamic oxalo-acetic transaminase, SGOT], alanine aminotransferase [ALT, or serum glutamic-pyruvic transaminase, SGPT]) and lactate dehydrogenase (LDH); serum B-HCG test for women potentially fertile
  Note: serum B-HCG test should be obtained within 14 days prior to C1D1
- Urine samples for qualitative (dipstick) analysis, (protein, glucose, urobilinogen, RBC, and WBC)
- Basal tumor markers: CEA and CA19.9
- AEs (including SAEs) from time signed Informed Consent is obtained until first dose of study drugs
- Peripheral blood samples for plasma biomarker analyses

Treatment assignment

After written informed consent has been obtained and eligibility established, each patient will be randomized as per Study Design (see Figure 1). Randomization should be performed within 3 days to C1D1.

4.4.3Assessment during treatment

Patients should receive study drug according to treatment schedule (Figure 2.1 and 2.2)

- ECOG performance status (Day 1 and day 15 of each cycle)
- Physical exam and Vital signs (as indicated above) at every visit, prior to any treatment cycle. (day 1 and day 15 of each cycle)
- Haematology (including haemoglobin, haematocrit, platelet count, WBC, ANC) within 72 hours prior to day 1 of each treatment cycle. (day 1 of each cycle)
- Biochemistry (including glucose, BUN, creatinine or creatinine clearance, sodium, potassium, calcium, magnesium, chloride, total bilirubin with fractionation into direct and indirect (if total bilirubin elevated), alkaline phosphatase, AST [SGOT], ALT [SGPT] and lactate dehydrogenase (LDH), prior to day 1 of each treatment cycle.

Note: procedures (haematology, biochemistry) have not to be repeated if performed as baseline (screening) within 7 days prior to randomization.

- Evaluation of tumor markers response: CEA and CA19.9 every 8 weeks ± 7 days.
- Evaluation of tumor response by CT, or MRI eligibility scan (chest, abdomen, pelvis, and other having resulted positive during baseline staging) conforming to RECIST v1.1 must be performed every 8 weeks ± 7 days until PD on treatment. Tumor assessments should be performed on this schedule regardless of whether study treatment has been administered or held, and also if the patient is off treatment due to reason other than PD (i.e. refusal or unacceptable toxicity).
- Peripheral blood samples for exploratory biomarker studies every 8 weeks ± 7 days, the first day before drugs administration of a treatment cycle until PD.
- AEs (including SAEs) throughout the study.

4.4.4 Assessments at End of Treatment

The End-of-Treatment assessments should be performed no later than 21 days+/- 7 days after patient's last dose of study medication. If the patient will be starting new anticancer therapy within the 21 days +/- 7 days after the last dose of study medication, the withdrawal visit should be performed prior to the start of new anticancer therapy.

- ECOG performance status
- Haematology (including haemoglobin, haematocrit, platelet count, WBC, ANC)
- Biochemistry (including glucose, BUN, creatinine or creatinine clearance, sodium, potassium, calcium, magnesium, chloride, total bilirubin with fractionation into direct and indirect (if total bilirubin elevated), alkaline phosphatase, AST [SGOT], ALT [SGPT] and lactate dehydrogenase (LDH)
- Concomitant disease and medications
- Documentation of AEs (CTCAE v 5.0 grading)
- Peripheral blood samples for plasma biomarker analyses

4.4.5 Follow-Up Assessments

All subjects who finish treatment, whichever the reason, will enter the follow-up. All subjects will be followed until death and data on subsequent treatment will be collected. Follow-up information on tumor status and vital status will be updated every 3 months until death. For patients with no progressive disease after terminating study treatment (i.e. treatment refusal,

unacceptable toxicity), tumor assessments, blood samples for plasma biomarker analyses, will be performed every 8 weeks ± 7 days until PD

If lost to follow-up, the Investigator should make every effort to contact the patient by telephone or by sending a registered letter to establish as completely as possible the reason for the withdrawal. A complete final evaluation at the time of the patient's withdrawal should be made with an explanation of why the patient is withdrawing from the study.

4.4.6 Collection of CT scans

It is required that CT scans or other relevant radiologic examinations used for staging and restaging procedures are collected and made available for eventual revision of response and time-to-progression definition.

Procedure	Screening period/Inclusion	On-treatment period			Withdrawal visit
		Cycles (28 days)		Based on Patient's status	
	Within 28 days prior C1D1	Day 1	Day 15		
Sign ICF[1]	X				
Demography	X				
MedicalHistory	X				
Histologic/cytologic confirmation[2]	X				
Previous surgery, radiotherapy and treatments	X				
ECG	X				
Inclusion/Non-inclusion criteria	X				
Pregnancy Testing[3]	X				
Patient number[4]	X				
Dispense Trifluridine/Tipiracil[5]		X			
Dispense Panitumumab[5]		X	X		
Efficacy measurements					

**Tumor Measurements**	X[6]			X[7]	X[7]
**Survival Status**		→	→	→	→
**Safety Measurements**					
**Physical Examination**	X	X[9]	X[9]		X
**ECOG**	X[8]	X[9]	X[9]		X
**Height**	X				
**12-lead ECG**	X				
**Vital signs & Weight**	X	X[9]	X[9]		X
**Haematology[10]**	X	X[12]			X
**Biochemistry[11]**	X	X[12]			X
**Urinalysis**	X				
**CEA, CA19-9[13]**	X			X	X
**Plasma Exploratory Biomarkers[14]**	X			X	X
**Concomitant treatments**	X	→	→	→	X
**AE Assessment[15]**	X	→	→	→	X
**Other measurements**					

Table 9. Study Assessment

Notes:

1. Sign Informed Consent Form (ICF): Written informed consent must be obtained prior to the performance of any study procedure.
2. Histologic/Cytological confirmation: Histological or cytological confirmation of metastatic adenocarcinoma of the colon or rectum, *RAS* wt, prior to administration of study drugs.
3. Pregnancy Testing: performed with serum βHCG test within 14 days prior to C1D1. Note: More frequent pregnancy assessments should be performed if required by local law.
4. Once patient has signed ICF, connection to the IWRS for patient's registration and eCRF patient number.
5. Study Medication: Trifluridine / Tipiracil will be administered twice daily (BID) on Days 1 to 5 and 8 to 12 of each treatment cycle. Panitumumab on day 1 and day 15 of each cycle.
6. Tumour Measurement at Screening Period: Tumour evaluation performed prior to patient ICF signature may be used if the date of the evaluation is within 28 days prior to randomization.
7. Tumour Measurement at Treatment Period: Perform tumour assessment based on general practices and standard of care of the site every 8 week ± 7 days from C1D1.
8. ECOG Performance Status: Patient's performance status must remain 0 or 1 during the screening
9. Physical examination, ECOG Performance Status, Vital signs and Weight have to be performed at screening and on Day 1 and on Day 15 pre-dose study drugs administration of each cycle. Prior to administering study drug, verify that patients with toxicities have met resumption criteria.
10. Haematology (including: haemoglobin, haematocrit, Platelet Count, white blood cell [WBC] count, absolute neutrophil count [ANC]) has to be performed at screening and within 72 hours prior to Day 1 study drug administration of each cycle. Prior to starting subsequent cycles, verify that patients with toxicities have met resumption criteria prior to administering study drug.
11. Biochemistry including: glucose, blood urea nitrogen (BUN), creatinine or creatinine clearance, sodium, potassium, calcium, magnesium, chloride, total bilirubin with fractionation into direct and indirect (if total bilirubin elevated), alkaline phosphatase, aspartate aminotransferase [AST, or serum glutamic oxalo-acetic transaminase, SGOT], alanine aminotransferase [ALT, or serum glutamic-pyruvic transaminase, SGPT]) and lactate dehydrogenase (LDH) has to be performed at screening and within 72 hours prior to Day 1 study drug administration of each cycle.
12. Procedures (haematology and biochemistry) for the first cycle have not to be repeated if performed as baseline (screening) within 7 days prior to C1D1. Prior to starting subsequent cycles, verify that patients with toxicities have met resumption criteria prior to administering study drug.
13. CEA (Carcinoembryonic antigen) and CA19-9(Carbohydrate Antigen 19-9) have to be performed during the screening period, every 8 weeks ± 7 days and at the End of Treatment.
14. Blood samples for plasma biomarker analysis have to be collected during the screening period, every 8 weeks ± 7 days and at the end of treatment.
15. <u>AE Assessment</u>: AE will be recorded from the first dose of study medication until the withdrawal visit. Any AE that occur prior to the first dose of study medication should be recorded as Medical History except events associated with any procedure/condition required by the study protocol: procedure (exercise test, MRI, etc.), change or withdrawal of previous/concomitant treatment relating to the conditions of the protocol, or a product other than the study drug, taken as part of the protocol.

Note: <u>Assessment Windows</u>: A window of +/-3 days is allowable for study procedures, as long as the proper order is maintained.

5. RESPONSE EVALUATION

Response must be evaluated through repetition of the CT or MRI scan of chest, abdomen and pelvis every 8 weeks from treatment start. Response will be codified by the Investigator according to the RECIST (Response Evaluation Criteria In Solid Tumors) guidelines version 1.1, summarized below.

5.1. Measurability of tumor lesions

At the baseline evaluation, the lesions will be defined as follows:

**measurable**:	Lesions which can be accurately measured on at least one dimension (the longest diameter must be recorded) and found to be ≥10 mm with CT scan or ≥ 20 mm with conventional techniques Lymph node found to be ≥ 15 mm in short axis with CT scan.<br><br>Note: All tumor measurements must be recorded in millimeters (or decimal fractions of centimeters)
**non-measurable**:	Lesions that are measurable but small (largest Diameter<10 mm with CT scan or <20 mm with conventional techniques or pathological lymph nodes with ≥ 10 to <15 mm short axis).<br><br>Bone lesions, leptomeningeal disease, ascites, pleural/pericardial effusions, lymphangitis of skin or lung, and abdominal masses or organomegaly (that cannot be followed by CT or MRI).<br><br>Lesions that are located in previously irradiates areas

All measurements must be made using a centimeter rule or gauge. All the baseline evaluations must be performed as close to the treatment start date as possible, and in all cases no more than 4 weeks to randomization.

5.2. Identification of "target" and "non-target" lesions

All measurable lesions up to a maximum of 2 lesions per organ and 5 lesions in total, representative of all involved organs, should be identified as target lesions and recorded and measured at baseline assessment. The target lesions should be selected on the basis of their size (lesions with the longest diameter), trying to represent all the involved organs. In addition, target lesions should be those that lend themselves to reproducible repeated measurements. It may be the case that, on occasion, the largest lesion does not lend itself to reproducible measurement in which circumstance the next largest lesion which can be measured reproducibly should be selected. The sum of the baseline diameters (longest for

non-nodal lesions, short axis for nodal lesions) of all target lesions will be calculated, reported in the eCRF, and used as reference for defining the objective response. All other lesions are identified as non-target lesions and must also be recorded at baseline assessment. Measurement of these lesions is not required, but the presence, absence, or the eventual unequivocal progression of each of them must be noted during the follow-up, at each scheduled restaging

5.3 Evaluation of target lesion response

Complete response (CR):	disappearance of all the target lesions. Any pathological lymph nodes (whether target or non-target) must have reduction in short axis to <10mm.
Partial Response (PR):	reduction of at least 30% in the sum of diameters of the target lesions, compared to the baseline evaluation.
Progression (PD):	increase of at least 20% in the sum of diameters of target lesions and an absolute increase of at least 5 mm, compared to the lowest sum recorded since the start of the treatment, or the appearance of one or more new lesions.
Stable disease (SD):	neither a sufficient reduction to be defined as PR, nor a sufficient increase to be defined as PD

5.4. Evaluation of non-target lesion response

Complete Response (CR):	Disappearance of all non-target lesions. All lymph nodes must be non-pathological in size(<10 mm short axis)
Non Complete Response / Non Progression (CR/non-PD):	Persistence of one or more target lesions
Progression (PD):	Appearance of one or more new lesions or indisputable, clear progression of an existing non-target lesion.

5.5. Evaluation of best overall response

The best overall response is the best response recorded from the start of treatment until disease progression/recurrence (taking the lowest measurement recorded since the start of treatment as reference for the progression).

Target Lesions	Non-Target Lesions	New Lesions	RECIST Response
CR	CR	No	**CR**

CR	non-CR/non-	No	**PR**
CR	Not evaluated	No	**PR**
PR	CR o non-CR/non-PD or not evaluated	No	**PR**
SD	CR o non-CR/non-PD or not evaluated	No	**SD**
Not all evaluated	CR o non-CR/non-PD	No	**Not evaluable**
PD	Any	Yes or No	**PD**
Any	PD	Yes or No	**PD**
Any	Any	Yes	**PD**

An overall deterioration in the state of health leading to a suspension of the treatment without evidence of progression will be defined as a "symptomatic worsening". Patients who suspend treatment due to symptomatic worsening will be considered as non-responding

5.6. Reporting of results

All patients included in the study must be assessed for response to treatment. Each patient will be assigned one of the following categories: 1) complete response, 2) partial response, 3) stable disease, 4)progressive disease, 5) early death from malignant disease, 6) early death from toxicity, 7) early death because of other cause, or 8) unknown (not assessable, insufficient data).All patients will be included in the analysis of the response rate. Patients in response categories 4-8 will be considered as failing to respond to treatment.

6. BIOMARKERS

6.1 Blood Sample Collection and analysis

Patients should provide written informed consent to obtain plasma blood samples (at least 20 mL in EDTA tubes) at screening, tumor assessment time points and End of Treatment.

Plasma samples will be stored at -80 °C at local sites and subsequently will be shipped to the Sponsor.

6.2Tumor Tissue Sample Collection

Tumor Tissue Sample will be collected at baseline and subsequently will be shipped to the Sponsor

7. SAFETY INSTRUCTIONS AND GUIDANCE

7.1 Warning and management of specific toxicities

PANITUMUMAB

Summary of the Safety Profile

Based on an analysis of mCRC clinical trial patients receiving panitumumab monotherapy and in combination with chemotherapy (n = 2224), adverse reactions occurring in ≥ 20% of patients were gastrointestinal disorders (diarrhea [46%], nausea [39%], vomiting [26%], abdominal pain [23%], and constipation [23%]);

Hypersensitivity Reactions

Hypersensitivity reactions have been reported, including a fatal case of angioedema that occurred more than 24 hours after the infusion. Depending on the severity (eg, presence of bronchospasm, edema, angioedema, hypotension, need for parenteral medication, or anaphylaxis) and/or persistence of hypersensitivity reactions, permanently discontinue panitumumab.

Dermatologic and Soft Tissue Toxicity

Skin and subcutaneous tissue disorders, a pharmacologic effect observed with epidermal growth factor receptor (EGFR) inhibitors, were frequently reported (approximately 93% in patients across the monotherapy mCRC clinical trials [N = 1052]).It is recommended that patients wear sunscreen and hats and limit sun exposure while receiving panitumumab as sunlight can exacerbate any skin reactions that may occur. Patients who develop dermatologic or soft tissue toxicities while receiving panitumumab should be monitored for the development of inflammatory or infectious sequelae. Life-threatening and fatal infectious complications, including events of necrotizing fasciitis and/or sepsis, have been observed in patients treated with panitumumab. Rare cases of Stevens-Johnson syndrome and toxic epidermal necrolysis have been reported in patients treated with panitumumab in the post-marketing setting.
Stop or discontinue panitumumab for dermatologic or soft tissue toxicity associated with severe or life-threatening inflammatory or infectious complications.

Other Warnings and Precautions

Proactive skin treatment including skin moisturizer, sun screen (sun protection factor[SPF] > 15 ultraviolet A [UVA] and ultraviolet B [UVB]), topical steroid cream(not stronger than 1% hydrocortisone) and an oral antibiotic (eg, doxycycline), as prescribed by the physician, are useful in the management of skin toxicities. Subjects should be advised to apply moisturizer and sunscreen to face, hands, feet, neck, back, and chest every morning during treatment and, if any contraindications, to apply the topical steroid to face, hands, feet, neck, back, and chest every night. Treatment of skin

reactions should be based on severity and may include a moisturizer, sun screen (SPF >15 UVA and UVB), and topical steroid cream (not stronger than 1% hydrocortisone) applied to affected areas, and/or oral antibiotics, as prescribed by the physician.

Pulmonary Toxicity

Fatal and non-fatal cases of interstitial lung disease (ILD) have been observed in patients treated with EGFR inhibitors including panitumumab. In the event of acute onset or worsening of pulmonary symptoms, panitumumab therapy should be interrupted and a prompt investigation of these symptoms should occur. If ILD is confirmed, panitumumab should be permanently discontinued and the patient should be treated appropriately. In patients with a history of interstitial pneumonitis or pulmonary fibrosis or evidence of interstitial pneumonitis or pulmonary fibrosis, the benefits of therapy with panitumumab versus the risk of pulmonary complications must be carefully considered.

Laboratory Tests

Electrolyte Disturbances/Monitoring

Progressively decreasing serum magnesium levels leading to severe hypomagnesemia have been observed in some patients. Patients should be monitored for hypomagnesemia and accompanying hypocalcemia prior to initiating panitumumab treatment, and periodically during panitumumab treatment and for up to 8 weeks after the completion of treatment. Magnesium repletion is recommended, as appropriate.

Other electrolyte disturbances, including hypokalemia, have also been observed. Repletion of these electrolytes is also recommended, as appropriate.

Trifluridine-Tipiracil

Myelosuppressive toxicities

In the RECOURSE trial, the incidence of Grade ≥3 neutropenia was 38% in patients treated with Trifluridine/Tipiracil, the incidence of febrile neutropenia was 4%. In the event of Grade 4 neutropenia (absolute neutrophil count [ANC] <0.5 x 10^9/L) within a treatment cycle, the dose of Trifluridine/Tipiracil should be held until the ANC increases to ≥1.5 x 10^9/L(see section 4.3.2; Table 6-7). If the delay is longer than 1 week, the dose of Trifluridine/Tipiracil in the next cycle should be reduced by 5 mg/m2 per dose from the previous dose level. If febrile neutropenia occurs, the dose of Trifluridine/Tipiracil should be held until the episode of febrile neutropenia is completely resolved, and the drug dose in the next cycle should be reduced by 5 mg/m2 per dose from the previous dose level (see Table 8). Prophylactic administration of G-CSF is recommended to prevent the development of

febrile neutropenia, according to current guidelines (J. Klastersky et al 2016, ESMO guidelines). In the RECOURSE study G-CSF support was used in 9.4% of patients

Anemia

Red blood cell transfusion is the recommended treatment for patients with symptomatic anemia due to myelosuppressive chemotherapy, and the use of transfusions should also be considered for asymptomatic patients with anemia who have underlying comorbidities.

Thrombocytopenia

For platelet counts <0.5 x 10^9/L, the dose of Trifluridine/Tipiracil should be held until the platelet counts increase to ≥75 x 10^9/L (see section 4.3.2; Table 6- Table 7). If the delay is longer than 1 week, the dose of Trifluridine/Tipiracil in the next cycle should be reduced by 5 mg/m^2 per dose from the previous dose level. Prophylactic platelet transfusions are recommended at a threshold platelet count of 10 x 10^9/L for patients with solid tumors who have chemotherapy-induced thrombocytopenia.

Non-hematologic AEs

In the RECOURSE trial the Trifluridine/Tipiracil group had higher rates of Grade ≥3 nausea (2% vs. 1%), vomiting (2% vs. < 1%), and diarrhea (3% vs. < 1%). However, there were no clinically meaningful differences between the treatment groups in the percentage of patients with other non-hematologic AEs, including decreased appetite, stomatitis, and hand-foot syndrome. For Grade ≥3 non-hematologic AEs, the dose of Trifluridine/Tipiracil should be held until resolution of the AE to Grade 0 or 1 (see section 4.3.2; Table 6-7). With the exception of Grade 3 nausea, vomiting, or diarrhea that is controlled by medication, the dose of Trifluridine/Tipiracil in the next cycle should be reduced by 5 mg/m2 per dose from the previous dose level for Grade ≥3 non-hematologic AEs (Table 3).

7.2 Adverse Events and Laboratory Abnormalities

7.2.1 Clinical Adverse Events (AEs)

According to the International Conference of Harmonization (ICH), an AE is any untoward medical occurrence in a patient or clinical investigation subject administered a pharmaceutical product and which does not necessarily have a causal relationship with this treatment. An AE can therefore be any unfavorable and unintended sign [including an abnormal laboratory finding], symptom, or disease temporally associated with the use of a medicinal (investigational) product, whether or not considered related to the medicinal (investigational) product. Pre-existing conditions worsening during thestudy are to be reported as AEs.

Intensity

Intensity of all AEs will be graded according to the NCI CTCAE v.5.0 on a five-point scale (Grade 1 to 5) and reported in detail on the CRF.

AEs not listed on the CTCAE should be graded as follows:

- Grade 1: Mild Discomfort noticed but no disruption of normal daily activity
- Grade 2: Moderate Discomfort sufficient to reduce or affect daily activity; no treatment or medical intervention is indicated although this could improve the overall well-being or symptoms of the patient
- Grade 3: Severe Inability to work or perform normal daily activity; treatment or medical intervention is indicated in order to improve the overall wellbeing or symptoms; delaying the onset of treatment is not putting the survival of the patient at direct risk
- Grade 4: Life threatening/disabling. An immediate threat to life or leading to a permanent mental or physical conditions that prevents work or performing normal daily activities; treatment or medical intervention is required in order to maintain survival
- Grade 5: AE resulting in death

7.2.2Drug AE relationship Adverse (drug) reaction (ADR)

The causality relationship of study drug to the AE will be assessed by the Investigator as either: *Yes* or *No*

If there is a reasonable suspected causal relationship to the study medication, i.e. there are facts (evidence) or arguments to suggest a causal relationship, drug-event relationship should be assessed as Yes.

The following criteria should be considered in order to assess the relationship as Yes:

- Reasonable temporal association with drug administration
- It may or may not have been produced by the patient's clinical state, environmental or toxic factors, or other modes of therapy administered to the patient
- Known response pattern to suspected drug
- Disappears or decreases on cessation or reduction in dose
- Reappears on re-challenge
- An **adverse (drug) reaction** (ADR) is a response to a medicinal product which is noxious and unintended. Response in this context means that a causal relationship between a medicinal product and an adverse event is at least a reasonable possibility. Adverse reactions may arise from use of the product within or outside the terms of the marketing authorization or from occupational exposure. Conditions of use outside the marketing authorization include overdose, misuse, abuse, and medication errors.

The following criteria should be considered in order to assess the relationship as No:

- It does not follow a reasonable temporal sequence from administration of the drug
- It may readily have been produced by the patient's clinical state, environmental or toxic factors, or other modes of therapy administered to the patient
- It does not follow a known pattern of response to the suspected drug
- It does not reappear or worsen when the drug is re-administered

7.2.3 Serious Adverse Events (SAE)

A SAE is any experience that suggests a significant hazard, contraindication, side effect or precaution. It is any AE that at any dose fulfills at least one of the following criteria:

- is fatal (results in death; NOTE: death is an outcome, not an event)

- is Life-Threatening (NOTE: the term "Life-Threatening" refers to an event in which the patient was at immediate risk of death at the time of the event; it does not refer to an event which could hypothetically have caused a death if it had been more severe).
- requires in-patient hospitalization or prolongation of existing hospitalization;
- results in persistent or significant disability/incapacity;
- is a congenital anomaly/birth defect;
- is another Important Medical Event (any important adverse events/reactions that is not immediately life-threatening or do not result in death or hospitalization, but may jeopardize the subject or may require medically significant or requires intervention to prevent one or other of the outcomes listed above).
- Examples of such events include, but are not limited to, intensive treatment in an emergency room or at home for allergic bronchospasm, blood dyscrasias or convulsions that do not result in hospitalization).

Note: The term sudden death should be used only when the cause is of a cardiac origin as per standard definition. The terms death and sudden death are clearly distinct and must not be used interchangeably. The study will comply with all local regulatory requirements and adhere to the full requirements of the ICH Guideline for Clinical Safety Data Management, Definitions and Standards for Expedited Reporting.

7.2.4 Serious adverse drug reaction (SADR)

A **Serious adverse drug reaction** (SADR) is an adverse reaction which results in death, is life-threatening, requires in-patient hospitalization or prolongation of existing hospitalization, results in persistent or significant disability or incapacity, or is a congenital anomaly/birth defect.

Medical and scientific judgment should be exercised in deciding whether other situations should be considered serious adverse reactions, such as important medical events that might not be immediately life threatening or result in death or hospitalization but might jeopardize the patient or might require intervention to prevent one of the other outcomes listed above.

Examples of such events are intensive treatment in an emergency room or at home for allergic bronchospasm, blood dyscrasias, or convulsions that do not result in hospitalization, or development of dependency or abuse.

Any suspected transmission via a medicinal product of an infectious agent is also considered a serious adverse reaction.

7.2.5 Special situations:

Overdose

An overdose is a significant variation above the recommended/scheduled dosage for a product. In this current trial an overdose of the study drugs is any dose higher than the dose specified in the Sections 4.3.1.

There is no known antidote available in case of Trifluridine/Tipiracil overdose. Overdose should be managed aggressively with close monitoring and administration of prophylactic and symptomatic therapies to prevent or correct potential side effects.

Abuse, misuse, occupational exposure

Misuse refers to situations where the medicinal product is intentionally and inappropriately used not in accordance with the authorized product information.

Abuse corresponds to the persistent or sporadic, intentional excessive use of a medicinal product, which is accompanied by harmful physical or psychological effects.

Occupational exposure refers to the exposure to a medicinal product, as a result of one's professional or non-professional occupation.

Medication errors

A medication error for this study is defined as any accidental incorrect administration of a medicinal product. The error may be related to the administration of a wrong medication, nature of the medication, route of administration, dosage or frequency of the treatment as specified in this protocol (including omission of one or more administrations).

- Medication errors with concomitant medication treatment will not be recorded in the CRF unless they result in an AE.
- Medication errors with the study medication that result in an AE will be captured as an AE in the CRF.
- Medication errors with the study medication that result in the omission of an administration, an incorrect dose (relative to that specified in this protocol), or the administration of more than the prescribed dose will be identified through the recording of study drug accountability data in the CRF.
- Medication errors with the study medication resulting in an overdose, incorrect route of administration, or administration with the incorrect study drug will be reported

Planned Hospitalization

A hospitalization planned by the subject prior to signing the informed consent form (ICF) is considered a therapeutic intervention and not the result of a new SAE and should be recorded as medical history. If the planned hospitalization or procedure is executed as planned, the record in the subject's medical history is considered complete. However, if the event/condition worsens during the trial, it must be reported as an AE.

7.3 Treatment and Follow-up of AEs

Please see section 7.1. for general guidelines for management of specific toxicities.

A general principle is that differential diagnoses should be diligently evaluated according to standard medical practice. Non-inflammatory etiologies should be considered and appropriately treated.

The treatment and follow up of all the AEs should follow the indication provided below.

- For Related AEs, follow until one of the following occurs:
  - Resolved or improved to baseline;
  - Relationship is reassessed as unrelated;
  - Death;
  - Investigator confirms that no further improvement can be expected;
  - Clinical or safety data will no longer be collected, or final database closure.
- For Unrelated severe or life-threatening AEs, follow until one of the following occurs:
  - Resolved or improved to baseline;
  - Severity improved to Grade 2;
  - Death;
  - Investigator confirms that no further improvement can be expected;
  - Clinical or safety data will no longer be collected, or final database closure.

The final outcome of each AE must be recorded on the CRF.

7.4 Laboratory Test abnormalities

Laboratory test results will be recorded on the laboratory results form of the eCRF, or appear on electronically produced laboratory reports submitted directly from the central laboratory, if applicable. Any treatment-emergent abnormal laboratory result that is clinically significant, i.e., meeting one or more of the following conditions, should be recorded as a single diagnosis on the AE page in the eCRF:

- Accompanied by clinical symptoms

- Leading to a change in study medication (e.g. dose modification, interruption or permanent discontinuation)
- Requiring a change in concomitant therapy (e.g. addition of, interruption of, discontinuation of, or any other change in a concomitant medication, therapy or treatment)

This applies to any protocol and non-protocol specified safety and efficacy laboratory result from tests performed after the first dose of study medication, which falls outside the laboratory reference range and meets the clinical significance criteria.

7.4.1 Follow-up of Abnormal Laboratory Test Values

In the event of medically significant unexplained abnormal laboratory test values, the tests should be repeated and followed up until they have returned to the normal range and/or an adequate explanation of the abnormality is found. If a clear explanation is established it should be recorded on the eCRF.

7.5 Handling of Safety Parameters

7.5.1 Reporting of AEs

Information about all adverse events, whether volunteered by the patient, discovered by Investigator questioning, or detected through physical examination, laboratory test or other means, will be collected on the Adverse Event CRF page, documented in the patient's medical records, and followed as appropriate.

The NCI CTC-AE (Version 5.0) will be used to evaluate the clinical safety of the treatment in this study. Patients will be assessed for AEs at each clinical visit and as necessary throughout the study.

7.5.2 Reporting of SAEs (immediately reportable)

Any clinical AE, or abnormal laboratory test value assessed as serious (as defined above), pregnancy case, occurred during the course of this study from the enrolment visit (start of study screening procedures), including long term follow-up, must be reported to Sponsor and within *one* working day of the Investigator becoming aware of the event (expedited reporting).

The Investigator must complete the SAE form and forward it to the SAE Responsible person:

**Emergency contact**	Prof. Fortunato Ciardiello	0815666729

	Dr E. Martinelli, Dr T. Troiani	0815666628

In addition, report of Adverse Events will be done, according to current local law, to local Health Authorities.

From the first administration of study drugs, all SAEs must be reported. Related SAEs MUST be collected and reported regardless of the time elapsed from the last study drugs administration, even if the study has been closed. Suspected Unexpected Serious Adverse Reactions (SUSARs) are reported to Investigators at each site and associated IRB/IEC when the following conditions occur:

- The event must be a SAE
- There must be a certain probability that the event is an adverse reaction from the administered drugs
- The adverse reaction must be unexpected, that is to say, not foreseen in the Investigator's Brochure of study drugs. When all patients at a particular site are off treatment as defined by the protocol:
  - only individual SUSAR reports originating in that particular trial will be forwarded to the site and associated Insitutional Review Board (IRB) / Independent Ethics Committee (IEC) on an expedited basis
  - individual SUSARs considered to be a significant safety issue and/or which result in recommending a change to the ICF, will be reported in an expedited manner to all Investigators and IRBs/IECs
- SUSAR reports originating from other trials using the same study drugs will be provided as six monthly SUSAR Reports to Investigators and IRBs/IECs where long-term follow-up studies are carried out, unless they are considered significant

Unrelated SAEs must be collected and reported during the study and for up to five years after the last dose of study medication and every 3 months for long-term follow up.

This study adheres to the definition and reporting requirements of ICH Guideline for Clinical Safety Data Management, Definitions and Standards for Expedited Reporting.

7.5.3 Pregnancy

Female patients must be instructed immediately inform the investigator if they become pregnant during the study. The investigator should report all pregnancies within 24 hours to the Sponsor and, using the Clinical Trial Pregnancy Reporting Form. The investigator should counsel the patient, discuss the risks of continuing with the pregnancy and the possible effects on the fetus.

Pregnancies occurring up to 6 months after the completion of the study medication must also be reported to the Investigator. Pregnancies occurring in the partner of a male patient participating in the study

should be reported to the investigator and the sponsor. The partner should be counseled, the risks of continuing the pregnancy discussed, as well as the possible effects on the fetus. Monitoring of the patient should continue until conclusion of the pregnancy.

7.5.4 Safety data exchange requirement

Investigators should forward all SAEs and SUSARS to the Sponsor as soon as possible, but no later than 1 business day; pregnancy and lactation reports must be sent within 7days.
The Sponsor will provide Amgen with a copy of any SAE report received by the Investigators. Furthermore a copy of SUSARS must be sent to Amgen at the time of regulatory submission.
The Sponsor will also provide Amgen with a yearly Safety Report and with a copy of any other communication. Aggregate reports, containing safety data generated during the course of the study are to be sent to the Regulatory Authorities and to Amgen by the sponsor.
The final study report to be sent to Amgen no later than one calendar year from study completion.

The Sponsor will provide Servier with a copy of any SAE report received by the Investigators. Furthermore, a copy of SUSARS must be sent to Servier at the time of regulatory submission.
The Sponsor will also provide Servier with a yearly Safety Report and with a copy of any other communication. Aggregate reports, containing safety data generated during the course of the study are to be sent to the Regulatory Authorities and to Servier by the sponsor
The final study report to be sent to Servier no later than one calendar year from study completion

8. STATISTICAL CONSIDERATIONS

8.1 Sample size calculation

This is a randomized phase II, open label, two arm study, evaluating the efficacy of Trifluridine-Tipiracil in combination with panitumumab as third line therapy, after a first line containing an anti-EGFR agent panitumumab or cetuximab.
The study is designed to have 80% power to detect a hazard ratio for progression of 0.56 (a 44% reduction in risk) in the panitumumab+ Trifluridine-Tipiracil group as compared with the Trifluridine-Tipiracil group, with a two-sided type I error rate of 0.1. Given the treatment assignment ratio of 1:1 (Trifluridine-Tipiracil: panitumumab + Trifluridine-Tipiracil), we calculate that at least 74 events (progression) in 112 subjects (56 in the control group and 56 in the treatment group) would be required for the primary analysis.

8.2 Analysis Populations

Baseline is defined as the last valid visit or day before the treatment start. The following populations are defined for this study:

All Enrolled Set

All screened subjects who have been enrolled.

Intention To Treat (ITT) set

All enrolled patients that start the treatment will be considered the Intention-To-Treat population (ITT).

Safety Set

The subset of patient of the ITT population receiving at least one dose of the study medication will define the Safety Population (SP).

8.3 Statistical analysis

The median period of follow-up will be calculated for the entire study cohort according to the reverse Kaplan–Meier method. Distributions of time-to-event variables will be estimated with the use of the Kaplan–Meier product-limit method. The stratified log-rank test will be used as the primary analysis for comparison of treatment groups. Cox proportional-hazards modeling will be also performed as supportive analyses. Subgroup analyses of progression-free survival will be performed by means of an interaction test to determine the consistency of the treatment effect according to key baseline characteristics. Overall survival will be analyzed with the same methods used for the analysis of progression-free survival.

The objective response rate and the incidence of adverse events in the different groups will be compared with the use of the chi-square test for heterogeneity.

All statistical tests will be two-sided, and P values of 0.05 or less will be considered to indicate statistical significance. Odds ratios and 95% confidence intervals will be estimated with a logistic-regression model, and hazard ratios and 95% confidence intervals will be estimated with a Cox proportional hazards model.

Data handling

ORR and its association with prognostic factors will be investigated using the chi-square test and a logistic regression model will be used to study interrelationship among factors.

Safety and tolerability will be assessed in terms of AEs, laboratory data, ECG data, vital signs and weight, which will be collected for all patients. AEs (both in terms of MedDRA preferred terms and CTCAE grade), laboratory data, ECG data, vital signs data and weight will be listed individually by the patient and summarized by treatment received. Worst grade for each toxicity will be reported according to their relationship to study drugs. Treatment discontinuation due to AEs will also be described according to the treatment exposure.

Treatment duration, dose intensity, dose modification and discontinuation will be analyzed in each arm. Due to the small sample size, statistical analysis of **biomarkers data** will be conducted with the aim of hypothesis generation. A complete description of data will be done. For biomarkers that might change over time as a consequence of treatment, levels before and after treatment will be compared with appropriate statistical tests, based on the type of data. Repeated measurement ANOVA model will be used to test differences along time.

Correlation with Outcomes will be evaluated with univariate regression models. P values ≤ 0.05 will be considered significant, and no adjustment is planned for multiple comparisons due to the exploratory nature of the analysis of the secondary endpoints.

9. DATA COLLECTION AND MANAGEMENT

9.1 Data confidentiality

Information about study subjects will be kept confidential and managed under the applicable laws and regulations. The data collection system for this study uses built-in security features to encrypt all data for transmission in both directions, preventing unauthorized access to confidential participant information. Access to the system will be controlled by a sequence of individually assigned user identification codes and passwords, made available only to authorized personnel who have completed prerequisite training.

9.2 Site monitoring

Before study initiation, at a site initiation visit or at an investigator's meeting, Sponsor personnel will review the protocol and CRFs with the investigators and their staff. During the study, the field monitor will visit the site regularly to check the completeness of patient records, the accuracy of entries on the CRFs, the adherence to the protocol to Good Clinical Practice, the progress of enrolment, and to ensure that study treatment is being stored, dispensed, and accounted according to specifications. Key study personnel must be available to assist the field monitor during these visits. The investigator must maintain source documents for each patient in the study, consisting of case and visit notes (hospital or clinic medical records) containing demographic and medical information, laboratory data, electrocardiograms and the results of any other tests or assessments. All information recorded on CRFs must be traceable to source documents in the patient's file. The investigator must also keep the original signed informed consent form (a signed copy is given to the patient). The investigator must give the monitor access to all relevant source documents to confirm their consistency with the CRF entries. The Sponsor monitoring standards require full verification for the presence of informed consent, adherence to the inclusion/exclusion criteria and documentation of SAEs.

9.3 Data collection

This study will use an Electronic Data Capture (EDC) system (eClinical platform provided by Clinical Research Technology). The designated investigator staff will enter the data required by the protocol into the Electronic Case Report Forms (eCRF). The eCRFs have been built using eClinical platform provided by Clinical Research Technology, a fully validated secure web-enabled software that conforms to Food and Drug Administration (FDA) requirements. Investigator site staff will not be given access to the EDC system until they have been trained. Automatic validation programs check for data discrepancies in the eCRFs allow modification or verification of the entered data by the investigator staff. The Principal Investigator is responsible for assuring that the data entered into eCRF is complete, accurate, and that entry and updates are performed in a timely manner.

9.4 Database management and quality control

The Sponsor personnel (or designated contract research organization [CRO]) will review the data entered by investigational staff for completeness and accuracy. Electronic data queries stating the nature of the problem and requesting clarification will be created for discrepancies and missing values and sent to the investigational site via the EDC system. Designated investigator site staff is required to respond promptly to queries and to make any necessary changes to the data. Concomitant treatments entered into the database will be coded using the WHO Drug Reference List, which employs the Anatomical Therapeutic Chemical classification system. Medical history/current medical conditions and adverse events will be coded using the Medical dictionary for regulatory activities (MedDRA) terminology. The occurrence of any protocol violations will be determined. After the data have been verified to be complete and accurate, the database will be declared locked. Authorization is required prior to making any database changes to locked data, by joint written agreement between the Biostatistics and Data Management and the Sponsor.

10. ETHICAL CONSIDERATION

10.1 Regulatory and ethical compliance

This clinical study was designed, shall be implemented and reported in accordance with the ICH Harmonized Tripartite Guidelines for Good Clinical Practice, with applicable local regulations (including European Directive 2001/20/EC), and with the ethical principles laid down in the Declaration of Helsinki.

10.2 Responsibilities of the investigator and IEC

The protocol and the proposed informed consent form must be reviewed and approved by Independent Ethics Committee (IEC) of all participating centers before study start.

10.3 Informed consent procedures

Eligible patients may only be included in the study after providing written (witnessed, where required by law or regulation), IEC-approved informed. Informed consent must be obtained before conducting any study-specific procedures (i.e. all of the procedures described in the protocol). The process of obtaining informed consent should be documented in the patient source documents. The date when a subject’s Informed Consent was actually obtained will be captured in their CRFs.

10.4 Publication of study protocol and results

The key design elements of this protocol will be posted in the publicly accessible database clinicaltrialsregister.eu. The Investigators assure that results of this study will be submitted for publication and reported in scientific meetings.

10.5 Study documentation, record keeping and retention of documents

Each participating site will maintain appropriate medical and research records for this trial, in compliance with Section 4.9 of the ICH E6 GCP, and regulatory and institutional requirements for the protection of confidentiality of subjects. Each site will permit authorized representatives of the sponsor and regulatory agencies to examine (and when required by applicable law, to copy) clinical records for the purposes of quality assurance reviews, audits and evaluation of the study safety and progress. Source data are all information, original records of clinical findings, observations, or other activities in a clinical trial necessary for the reconstruction and evaluation of the trial. Examples of these original documents and data records include, but are not limited to, hospital records, clinical and office charts, laboratory notes, memoranda, subjects' diaries or evaluation checklists, pharmacy dispensing records, recorded data from automated instruments, copies or transcriptions certified after verification as being accurate and complete, microfiches, photographic negatives, microfilm or magnetic media, x-rays, and subject files and records kept at the pharmacy, at the laboratories, and medico-technical departments involved in the clinical trial. Data collection is the responsibility of the clinical trial staff at the site under the supervision of the site Principal Investigator. The study CRF is the primary data collection instrument for the study. The investigator should ensure the accuracy, completeness, legibility, and timeliness of the data reported in the CRFs and all other required reports. Data reported on the CRF, that are derived from source documents, should be consistent with the source documents or the discrepancies should be explained. All data requested on the CRF must be recorded. Any missing data must be explained. For electronic CRFs an audit trail will be maintained by the system. The investigator/institution should maintain the trial documents as specified in Essential Documents for the Conduct of a Clinical Trial (ICH E6 Section 8) and as required by applicable regulations and/or guidelines. The investigator/institution should take measures to prevent accidental or premature destruction of these documents. Essential documents (written and electronic) should be retained for a period of not less than seven (7) years from the completion of the Clinical Trial unless Sponsor provides written permission to dispose of them or, requires their retention for an additional period of time because of applicable laws, regulations and/or guidelines

10.6 Confidentiality of study documents and patient records

The investigator must ensure anonymity of the patients; patients must not be identified by names in any documents submitted to the Sponsor. However, the Sponsor may have access to source data. Signed informed consent forms and patient enrolment log must be kept strictly confidential to enable patient identification at the site.

10.7 Audits and inspections

Source data/documents must be available to inspections by the Sponsor or designee or Health Authorities.

10.8 Financial disclosures

Financial disclosures should be provided by study personnel who is directly involved in the treatment or evaluation of patients at the site - prior to study start.

11. PROTOCOL ADHERENCE

Investigators ascertain they will apply due diligence to avoid protocol deviations. Under no circumstances should the investigator contact the Sponsor or its delegates, if any, monitoring the study to request approval of a protocol deviation, as no authorized deviations are permitted. If the investigator feels a protocol deviation would improve the conduct of the study this must be considered a protocol amendment, and unless such an amendment is agreed upon by the Sponsor and approved by the IEC it cannot be implemented.

11.1 Amendments to the protocol

Any change or addition to the protocol can only be made in a written protocol amendment that must be approved by the Sponsor, Health Authorities where required, and the IEC. Only amendments that are required for patient safety may be implemented prior to IEC approval. Notwithstanding the need for approval of formal protocol amendments, the investigator is expected to take any immediate action required for the safety of any patient included in this study, even if this action represents a deviation from the protocol.

12. ADMINISTRATIVE ASPECTS

The study is a non-profit investigator initiated trial not sponsored by the pharmaceutical company which produce the drug used in the trial.
Panitumumab will be provided by AMGEN, Trifluridine-Tipiracil by ServierItaly.
The promoter will provide an insurance policy to cover possible damages caused to patients participating in the trial.
Study protocol, patient information and informed consent will be submitted to Independent Ethical Committees and will only be started after their approval. Independent Ethical Committees will be periodically informed of study progress, safety and planned or premature end of the study.

12. 1 Investigational medical product

12.1.1 Formulation, Packaging and Labelling

Study drug packaging will bear a label with the identification required by local law, the protocol number, drug identification and dosage.

Drugs will be administered open label. Drugs will be required to be labelled as per local SOPs and regulations.

Medication labels will be in the local language and comply with the legal requirements of each country in which the study will be conducted.

12.1.2 Accountability, assessment of compliance and destruction of the drugs

Study treatments must be received by designated personnel at the study site, handled and stored safely and properly, and kept in a secured location to which only the Investigator and designated site personnel have access.

Upon receipt, the study drugs should be stored according to the instructions specified on the drug labels and Investigator's Brochure for drugs (Panitumumab and Trifludine-Tipiracil). Study medication is to be stored in a secure locked area while under the responsibility of the Investigator. Receipt and dispensing of study medication must be recorded by an authorized person at the Investigator's site.

Any unused study drugs can only be destroyed after being inspected and reconciled by the responsible Study Monitor unless study drug containers must be immediately destroyed as required for safety, or to meet local regulations (e.g., cytotoxics or biologics).

On-site destruction is allowed provided the following minimal standards are met:

- On-site disposal practices must not expose humans to risks from the drug.
- On-site disposal practices and procedures are in agreement with applicable laws and regulations, including any special requirements for controlled or hazardous substances.
- Written procedures for on-site disposal are available and followed. The procedures must be filed with the site's SOPs and a copy provided to the study sponsor upon request.
- Records are maintained that allow for traceability of each container, including the date disposed of, quantity disposed, and identification of the person disposing the containers. The method of disposal, i.e., incinerator, licensed sanitary landfill, or licensed waste disposal vendor must be documented.
- Accountability and disposal records are complete, updated, and available for the Monitor to review throughout the clinical trial period.

If conditions for destruction cannot be met the responsible Study Monitor will make arrangements for return of study drug.

It is the investigator's responsibility to arrange for disposal of all empty containers, provided that procedures for proper disposal have been established according to applicable state, local, and institutional guidelines and procedures, and provided that appropriate records of disposal are kept.

REFERENCES

Arnold D, Lueza B, Douillard JY, Peeters M, Lenz HJ, Venook A, Heinemann V, Van Cutsem E, Pignon JP, Tabernero J, Cervantes A, Ciardiello F. Prognostic nad predeictive value of primary tumour side in

patients with RAS wild-type metastatic colorectal cancer treated with chemotherapy and GFR directed antibodies in six randomized trials. Ann Oncol. 2017 Aug 1;28(8):1713-1729. doi: 10.1093/annonc/mdx175

Baba Y, Tamura T, Satoh Y, Gotou M, Sawada H, Ebara S, Shibuya K, Soeda J, Nakamura K.Mol Panitumumab interaction with TAS-102 leads to combinational anticancer effects via blocking of EGFR-mediated tumor response to trifluridine. Oncol. 2017 Aug;11(8):1065-1077. doi: 10.1002/1878-0261.12074. Epub 2017 May 30.

de Gramont, A. et al. Leucovorin and fluorouracil with or without oxaliplatin as first- line treatment in advanced colorectal cancer. J. Clin. Oncol. 18, 2938–2947 (2000).

Douillard J.Y. et al. Irinotecan combined with fluorouracil compared with fluorouracil alone as first-line treatment for metastatic colorectal cancer: a multicentre randomised trial. Lancet 355, 1041–1047 (2000).

Douillard,J.Y.et al. Randomized,phaseIIItrial of panitumumab with infusional fluorouracil, leucovorin, and oxaliplatin (FOLFOX4) versus FOLFOX4 alone as first-line treatment in patients with previously untreated metastatic colorectal cancer: the PRIME study. J. Clin. Oncol. 28, 4697–4705 (2010).

Falcone, A. et al. Phase III trial of infusional fluorouracil, leucovorin, oxaliplatin, and irinotecan (FOLFOXIRI) compared with infusional fluorouracil, leucovorin, and irinotecan (FOLFIRI) as first-line treatment for metastatic colorectal cancer: the GruppoOncologico Nord Ovest. J. Clin. Oncol. 25, 1670–1676 (2007).

Ferlay J, Soerjomataram I, Ervik M, et al. GLOBOCAN 2012 v1.0, Cancer Incidence and Mortality Worldwide: IARC Cancer Base No. 11. Lyon, France: International Agency for Research on Cancer, 2013

Grothey A, Van Cutsem E, Sobrero A, Siena S, Falcone A, Ychou M, Humblet Y, Bouché O, Mineur L, Barone C, Adenis A, Tabernero J, Yoshino T, Lenz HJ, Goldberg RM, Sargent DJ, Cihon F, Cupit L, Wagner A, Laurent D; CORRECT Study Group. Regorafenibmonotherapy for previously treated metastatic colorectal cancer (CORRECT): an international, multicentre, randomised, placebo-controlled, phase 3 trial. Lancet. 2013 Jan 26;381(9863):303-12

Hurwitz, H. et al. Bevacizumab plus irinotecan, fluorouracil, and leucovorin for metastatic colorectal cancer. N. Engl. J. Med. 350, 2335–2342 (2004).

Jeevan M Puthiamadathil and Benjamin A Weinberg. Emerging combination therapies for metastatic colorectal cancer – impact of trifluridine/tipiracil Cancer Manag Res. 2017; 9: 461–469.

J. Klastersky, J. de Naurois, K. Rolston, B. Rapoport, G. Maschmeyer, M. Aapro and J. Herrstedt Management of Febrile Neutropaenia: ESMO Clinical Practice GuidelinesAnnOncol (2016) 27 (suppl 5): v111-v118 (J. Klastersky et al 2016, ESMO guidelines)

Mayer RJ, Van Cutsem E, Falcone A, Yoshino T, Garcia-Carbonero R, Mizunuma N, Yamazaki K, Shimada Y, Tabernero J, Komatsu Y, Sobrero A, Boucher E, Peeters M, Tran B, Lenz HJ, Zaniboni A, Hochster H, Cleary JM, Prenen H, Benedetti F, Mizuguchi H, Makris L, Ito M, Ohtsu A; RECOURSE Study Group. Randomized trial of TAS-102 for refractory metastatic colorectal cancer. N Engl J Med. 2015 May 14;372(20):1909-19.

Mayer RJ, Van Cutsem E, Falcone A, Yoshino T, Garcia-Carbonero R, Mizunuma N, Yamazaki K, Shimada Y, Tabernero J, Komatsu Y, Sobrero A, Boucher E, Peeters M, Tran B, Lenz HJ, Zaniboni A, Hochster H, Cleary JM, Prenen H, Benedetti F, Mizuguchi H, Makris L, Ito M, Ohtsu A; RECOURSE

Study Group. Randomized trial of TAS-102 for refractory metastatic colorectal cancer. N Engl J Med. 2015 May 14;372(20):1909-19. doi: 10.1056/NEJMoa1414325.

Pietrantonio F, Perrone F, Biondani P, Maggi C, Lampis A, Bertan C, et al. Single agent panitumumab in KRAS wild-type metastatic colorectal cancer patients following cetuximab-based regimens: Clinical outcome and biomarkers of efficacy. Cancer BiolTher. 2013;14(12):1098–1103.

Santini D, Vincenzi B, Addeo R, Garufi C, Masi G, Scartozzi M, et al. Cetuximab rechallenge in metastatic colorectal cancer patients: how to come away from acquired resistance? Ann Oncol. 2012;23(9):2313–2318.

Schirripa M, Lenz HJ. Colorectal cancer: Overcoming resistance to anti-EGFR therapy - where do we stand? NatRevGastroenterolHepatol. 2016 May;13(5):258-9. doi: 10.1038/nrgastro.2016.52. Epub 2016 Mar 23.

Sforza V, Martinelli E, Ciardiello F, Gambardella V, Napolitano S, Martini G, Della Corte C, Cardone C, Ferrara ML, Reginelli A, Liguori G, Belli G, Troiani T. Mechanisms of resistance to anti-epidermal growth factor receptor inhibitors in metastatic colorectal cancer.
World J Gastroenterol. 2016 Jul 28;22(28):6345-61.

Takeshi Kato, Yoshinori Kagawa, Yoshito Komatsu, Eiji Oki, Takayuki Yoshino, Kentaro Yamazaki, Hirofumi Yasui, HironagaSatake, Kazunori Shibuya, Koji Oba, and Kensei Yamaguchi. A phase I/II study for panitumumab combined with TAS-102 in patients with *RAS* wild-type metastatic colorectal cancer (APOLLON study): Phase I results.
Journal of Clinical Oncology 2017 35:4_suppl, 770-770

Takeshi Kato, Yoshinori Kagawa, Yoshito Komatsu, Eiji Oki, Takayuki Yoshino, Kentaro Yamazaki, Journal of Clinical Oncology 35, no. 4_suppl (February 2017) 770-770.

Van Cutsem E. et al. Cetuximab and chemotherapy as initial treatment for metastatic colorectal cancer. N. Engl. J. Med. 360, 1408–1417 (2009).

Van Cutsem E, Tabernero J, Lakomy R et al. Addition of aflibercept to fluorouracil, leucovorin, and irinotecan improves survival in a phase III randomized trial in patients with metastatic colorectal cancer previously treated with an oxaliplatin- based regimen. J ClinOncol 2012; 30: 3499–3506.

Van Cutsem E, Cervantes A, Adam R, Sobrero A, Van Krieken JH, Aderka D, Aranda Aguilar E, Bardelli A, Benson A, Bodoky G, Ciardiello F, D'Hoore A, Diaz-Rubio E, Douillard JY, Ducreux M, Falcone A, Grothey A, Gruenberger T, Haustermans K, Heinemann V, Hoff P, Köhne CH, Labianca R, Laurent-Puig P, Ma B, Maughan T, Muro K, Normanno N, Österlund P, Oyen W, Papamichael D, Pentheroudakis G, Pfeiffer P, Price TJ, Punt C, Ricke J, Roth A, Salazar R, Scheithauer W, Schmoll HJ, Tabernero J, Taïeb J, Tejpar S, Wasan H, Yoshino T, Zaanan A, Arnold D. ESMO consensus guidelines for the management of patients with metastatic colorectal cancer. Ann Oncol. 2016 Aug;27(8):1386-422.

Wadlow RC, Hezel AF, Abrams TA, Blaszkowsky LS, Fuchs CS, Kulke MH, et al. Panitumumab in patients with KRAS wild-type colorectal cancer after progression on cetuximab. Oncologist. 2012;17(1):14. doi: 10.1634/theoncologist.2011-0452.

Yasutoshi Kuboki, Takayuki Yoshino, Takeshi Kato, Yoshinori Kagawa, Makio Gamoh, Hirofumi Yasui, Kentaro Yamazaki, Yoshito Komatsu, Hironaga Satake, Masahiro Goto, Hiroaki Tanioka, Eiji Oki, Masahito Kotaka, Akitaka Makiyama, Tadamichi Denda, Junpei Soeda, Kazunori Shibuya, Masaru Iwata, Koji Oba, Kensei Yamaguchi. APOLLON: A phase I/II study of panitumumab combined with TAS-102 in patients (pts) with RAS wild-type (wt) metastatic colorectal cancer (mCRC). J Clin Oncol 36, 2018 (suppl; abstr 3523)

Appendix 1:

Performance Status Criteria

WHO Performance Status Scale		Karnofsky Performance Scale	
**Grade**	Descriptions	**Percent**	Description
0	Normal activity. Fully active, able to carry on all pre-disease performance without restriction.	100	Normal, no complaints, no evidence of disease.
		90	Able to carry on normal activity; minor signs or symptoms of disease.
1	Symptoms, but ambulatory. Restricted in physically strenuous activity, but ambulatory and able to carry out work of a light or sedentary nature (e.g., light housework, office work)	80	Normal activity with effort; some signs or symptoms of disease.
		70	Cares for self, unable to carry on normal activity or to do active work.
2	In bed <50% of the time. Ambulatory and capable of all self-care, but unable to carry out any work activities. Up and about more than 50% of waking hours.	60	Requires occasional assistance, but is able to care for most of his/her needs.
		50	Requires considerable assistance and frequent medical care.
3	In bed >50% of the time. Capable of only limited self-care, confined in bed or chair more than 50% of waking hours.	40	Disabled, requires special care and assistance.
		30	Severely disabled, hospitalization indicated. Death not imminent.
4	100% bedridden. Completely disabled. Cannot carry on any self-care. Totallyconfined to bed or chair	20	Very sick, hospitalization indicated. Death not imminent.
		10	Moribund, fatal processes progressing rapidly.
5	Deceased.	0	Deceased.
