# Supplementary material for: Anti-EGFR Rechallenge in Patients With Refractory ctDNA RAS/BRAF wt Metastatic Colorectal Cancer: A Nonrandomized Controlled Trial
Source: JAMA Netw Open. 2024 Apr 9;7(4):e245635. doi: 10.1001/jamanetworkopen.2024.5635 (PMC11004834; doi:10.1001/jamanetworkopen.2024.5635)
Supplement: Supplement 2. — eFigure 1. Forest Plot of Progression Free Survival in Different Subgroup eFigure 2. Forest Plot of Overall Survival (OS) in Different Subgroup eTable 1. Progression Free and Overall Survival Rate in the Study Population eTable 2. Progression Free and Overall Survival Rate According to the Presence of Liver Metastasis eTable 3. Adverse Events [file jamanetwopen-e245635-s002.pdf]

Supplemental Online Content

Ciardiello D, Martinelli E, Troiani T, et al. Anti-EGFR rechallenge in patients with refractory ctDNA *RAS/BRAF* wt metastatic colorectal cancer: a nonrandomized controlled trial. *JAMA Netw Open*. 2024;7(4):e245635. doi:10.1001/jamanetworkopen.2024.5635

eFigure 1. Forest Plot of Progression Free Survival in Different Subgroup
eFigure 2. Forest Plot of Overall Survival (OS) in Different Subgroup
eTable 1. Progression Free and Overall Survival Rate in the Study Population
eTable 2. Progression Free and Overall Survival Rate According to the Presence of Liver Metastasis
eTable 3. Adverse Events

This supplemental material has been provided by the authors to give readers additional information about their work.

© 2024 Ciardiello D et al. *JAMA Network Open.*

eFigure 1. Forest Plot of Progression Free Survival in Different Subgroup

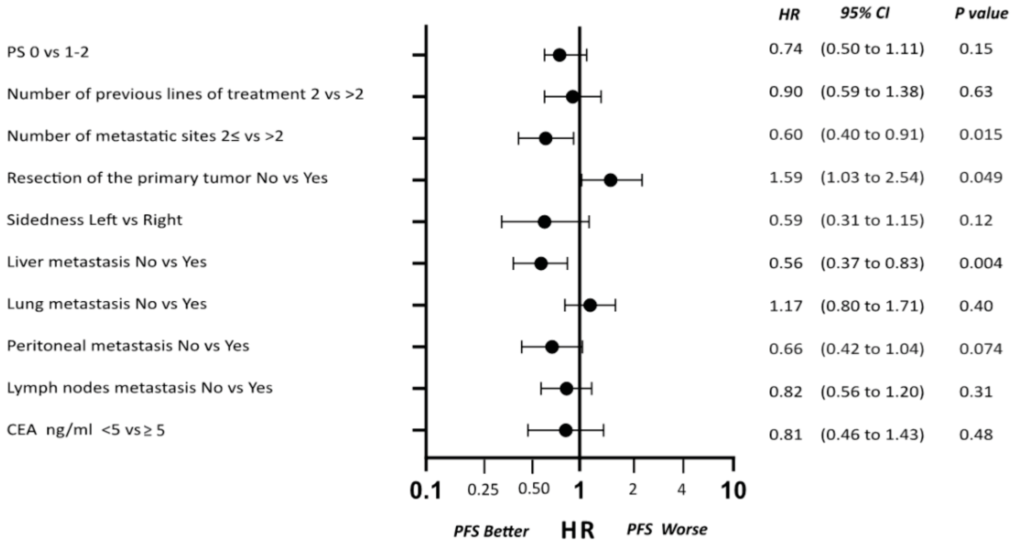

© 2024 Ciardiello D et al. *JAMA Network Open*.

eFigure 2. Forest Plot of Overall Survival (OS) in Different Subgroup

	HR	*95% CI*	*P value*
PS 0 vs 1-2	0.71	(0.46 to 1.08)	0.11
Number of previous lines of treatment 2 vs >2	1.07	(0.68 to 1.69)	0.76
Number of metastatic sites 2≤ vs >2	0.67	(0.43 to 1.02)	0.063
Resection of the primary tumor No vs Yes	1.45	(0.89 to 2.37)	0.13
Sidedness Left vs Right	1.00	(0.46 to 2.16)	1.00
Liver metastasis No vs Yes	0.63	(0.41 to 0.97)	0.036
Lung metastasis No vs Yes	1.02	(0.66 to 1.50)	0.99
Peritoneal metastasis No vs Yes	0.84	(0.51 to 1.35)	0.47
Lymph nodes metastasis No vs Yes	0.83	(0.54 to 1.24)	0.35
CEA ng/ml < 5 vs ≥ 5	0.65	(0.35 to 1.21)	0.17

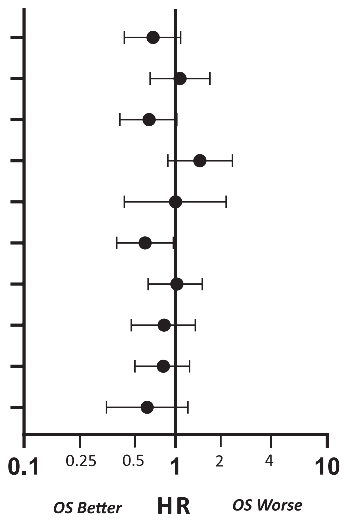

© 2024 Ciardiello D et al. *JAMA Network Open*.

eTable 1. Progression Free and Overall Survival Rate in the Study Population

PFS Rate		OS Rate	
6-months PFS rate (%)	32.5%	18-months OS rate (%)	36%
9-months PFS rate (%)	15%	24-months OS rate (%)	14.5%
12-months PFS rate (%)	10.5%	30-months OS rate (%)	13%

© 2024 Ciardiello D et al. *JAMA Network Open*.

eTable 2. Progression Free and Overall Survival Rate According to the Presence of Liver Metastasis

PFS Rate	Patients without liver metastasis	Patients with liver metastasis	OS Rate	Patients without liver metastasis	Patients with liver metastasis
6-months PFS rate (%)	45.2%	25.0%	18-months OS rate (%)	46.7%	29.8%
9-months PFS rate (%)	26.2%	9.7%	24-months OS rate (%)	24.7%	8.5%
12-months PFS rate (%)	21%	5.6%	30-months OS rate (%)	21.6%	8.5%

© 2024 Ciardiello D et al. *JAMA Network Open*.

eTable 3. Adverse Events

Adverse Events No. (%)	Grade 1-2	Grade 3-4
**Skin rash**	57 (62%)	24 (21%)
**Asthenia**	31 (27%)	0 (0%)
**Diarrhea**	18 (16%)	9 (8%)
**Dry skin**	21 (18%)	1 (1%)
**Nausea**	17 (15%)	1 (1%)
**Fever**	16 (14%)	0 (0%)
**Anemia**	15 (13%)	2 (2%)
**Conjunctivitis**	14 (12%)	0 (0%)
**Neutropenia**	13 (11%)	19 (17%)
**Mucositis**	13 (11%)	3 (3%)
**Vomiting**	13 (11%)	0 (0%)
**Pruritus**	11(10%)	0 (0%)
**Hypomagnesemia**	9 (8%)	2 (2%)
**Folliculitis**	8 (7%)	1 (1%)
**Blood bilirubin increase**	7 (4%)	2 (1%)
**Platelet count decreased**	6 (5%)	1 (1%)
**Anorexia**	6 (5%)	0 (0%)
**GOT/GPT increase**	6 (5%)	2 (2%)
**Stypsis**	5 (4%)	0 (0%)
**Palmar-plantar erythrodysesthesia syndrome**	3 (3%)	1 (1%)
**Nail ridging**	3 (3%)	0 (0%)
**Infusion related reaction**	2 (2%)	1 (1%)
**Nail changes**	2 (2%)	0 (0%)
**Hyperglycemia**	2 (2%)	0 (0%)
**Hypothyroidism**	2 (2%)	0 (0%)
**Hypertrichosis**	2 (2%)	0 (0%)
**Alopecia**	2 (2%)	0 (0%)
**Paronychia**	2 (2%)	0 (0%)
**Creatine increase**	2 (2%)	0 (0%)
**Hypomagnesemia**	1 (2%)	2 (2%)
**CPK increase**	1 (1%)	0 (0%)
**Constipation**	1 (1%)	0 (0%)
**Hyperthyroidism**	1 (1%)	0 (0%)
**Hypocalcemia**	1 (1%)	0 (0%)
**Hyperamylasemia**	0 (1%)	2 (2%)
**Myocardial infarction**	0 (1%)	1 (1%)

© 2024 Ciardiello D et al. *JAMA Network Open*.
