# Supplementary material for: Anti-EGFR Rechallenge in Patients With Refractory ctDNA RAS/BRAF wt Metastatic Colorectal Cancer: A Nonrandomized Controlled Trial
Source: JAMA Netw Open. 2024 Apr 9;7(4):e245635. doi: 10.1001/jamanetworkopen.2024.5635 (PMC11004834; doi:10.1001/jamanetworkopen.2024.5635)
Supplement: Supplement 3. — Data Sharing Statement [file jamanetwopen-e245635-s003.pdf]

Data Sharing Statement

Ciardiello. Anti-EGFR Rechallenge in Patients With Refractory ctDNA RAS/BRAF wt Metastatic Colorectal Cancer. *JAMA Netw Open*. Published April 09, 2024.
doi:10.1001/jamanetworkopen.2024.5635

Data
Data available: No

Additional Information
Explanation for why data not available: Researchers can request access to de-identified individual patient-level data from the corresponding author on a reasonable request.
